# Enantioselective Synthesis of α-Trifluoromethyl Amines via Biocatalytic N–H Bond Insertion with Acceptor-Acceptor Carbene Donors

**DOI:** 10.1021/jacs.1c10750

**Published:** 2022-02-02

**Authors:** Donggeon Nam, Antonio Tinoco, Zhuofan Shen, Ronald D. Adukure, Gopeekrishnan Sreenilayam, Sagar D. Khare, Rudi Fasan

**Affiliations:** † Department of Chemistry, University of Rochester, Rochester, New York 14627, United States; ‡ Department of Chemistry and Chemical Biology, Rutgers University, New Brunswick, New Jersey 08854, United States

## Abstract

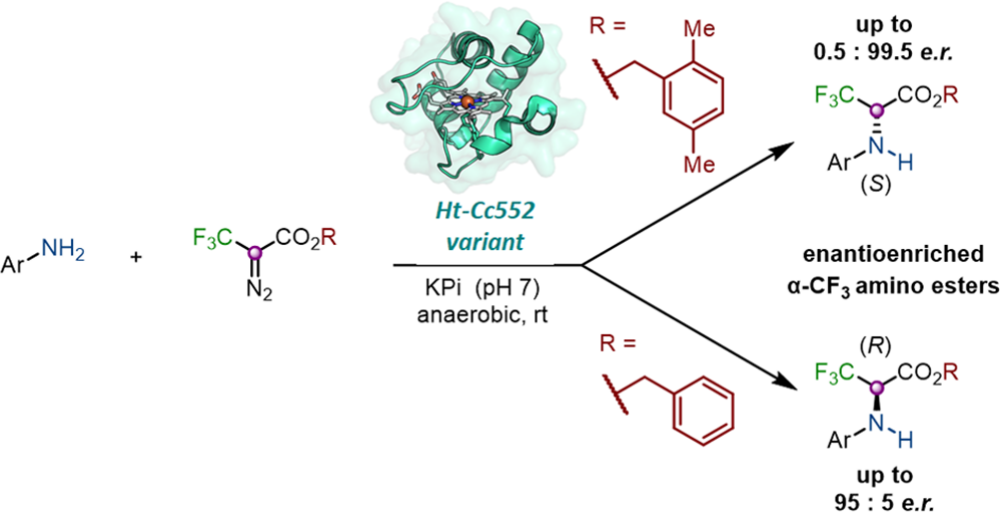

The biocatalytic toolbox has recently been expanded to include enzyme-catalyzed carbene transfer reactions not occurring in Nature. Herein, we report the development of a biocatalytic strategy for the synthesis of enantioenriched α-trifluoromethyl amines through an asymmetric N–H carbene insertion reaction catalyzed by engineered variants of cytochrome *c*_*552*_ from *Hydrogenobacter thermophilus*. Using a combination of protein and substrate engineering, this metalloprotein scaffold was redesigned to enable the synthesis of chiral α-trifluoromethyl amino esters with up to >99% yield and 95:5 er using benzyl 2-diazotrifluoropropanoate as the carbene donor. When the diazo reagent was varied, the enantioselectivity of the enzyme could be inverted to produce the opposite enantiomers of these products with up to 99.5:0.5 er. This methodology is applicable to a broad range of aryl amine substrates, and it can be leveraged to obtain chemoenzymatic access to enantioenriched β-trifluoromethyl-β-amino alcohols and halides. Computational analyses provide insights into the interplay of protein- and reagent-mediated control on the enantioselectivity of this reaction. This work introduces the first example of a biocatalytic N–H carbenoid insertion with an acceptor–acceptor carbene donor, and it offers a biocatalytic solution for the enantioselective synthesis of α-trifluoromethylated amines as valuable synthons for medicinal chemistry and the synthesis of bioactive molecules.

## Introduction

The incorporation of fluorine can favorably alter the physicochemical and biological properties of bioactive molecules.^1,2^ Fluorine-containing building blocks are increasingly used in medicinal chemistry, as the introduction of fluorine substituents can improve the pharmacokinetic and pharmacological properties of small-molecule drugs, including their potency, cell permeability, and metabolic stability.^3,4^

**Scheme 1:**
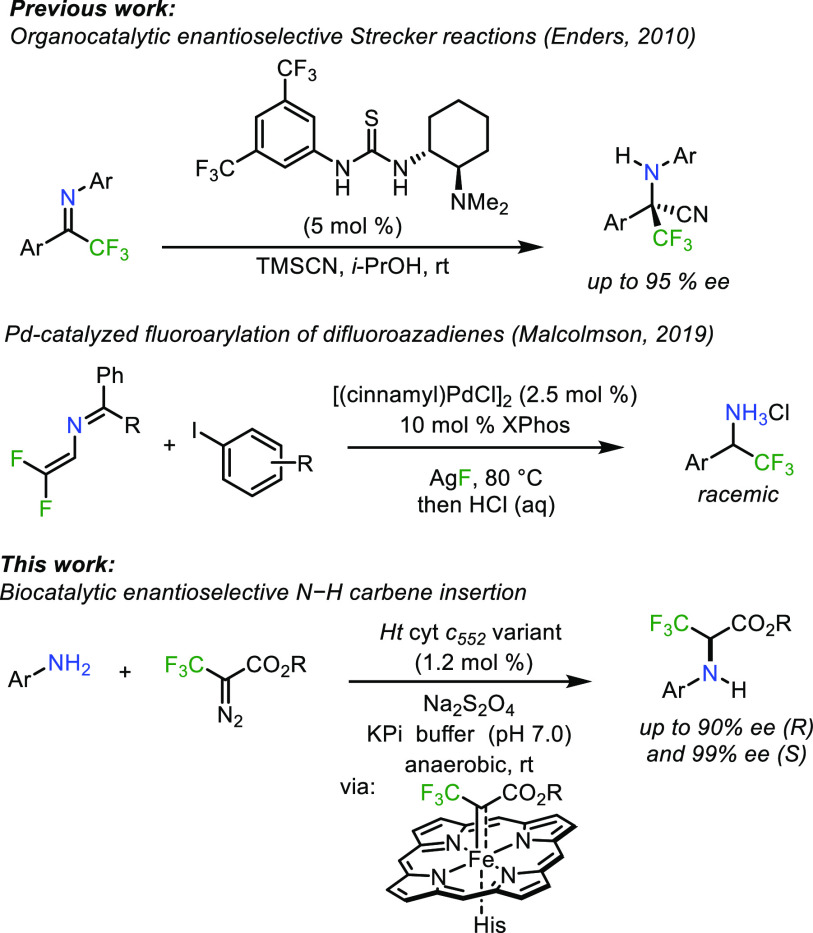
Representative Chemocatalytic Methods and Biocatalytic Strategy (This Work) For the (Asymmetric) Synthesis of α-Trifluoromethylated Amines

One group of organofluorines of great interest in drug discovery and development are chiral α-trifluoromethyl amine derivatives, such as substituted trifluoroethylamines^5,6^ and α-trifluoromethyl amino esters.^7^ These fluorinated building blocks can serve as unnatural amino acids useful for generating proteolytically stable peptides with increased lipophilic properties.^8,9^ Additionally, chiral α-trifluoromethyl amines and amino esters have been utilized as peptide mimics^5,10^ and as PLP-dependent enzyme suicide inhibitors,^11,12^ respectively, prompting considerable efforts toward the development of methodologies to afford these important fluorinated building blocks.^13^ Reported methods for the construction of chiral α-trifluoromethyl amines include the asymmetric reduction of *N*-arylimino trifluoropropanoic acid esters,^14^ asymmetric organocatalytic Strecker reactions,^15^ catalytic asymmetric umpolung reactions with trifluoromethylimines,^16,17^ and palladium-catalyzed vicinal fluoroarylation of *gem*-difluoro-2-azadienes,^18^ among others (Scheme 1).^19−21^ Despite this progress, these methods offer moderate levels of stereoselectivity and require the use of a preinstalled trifluoromethyl group, rare metals, or multiple steps to access the desired α-trifluoromethyl amino core. The transition-metal-catalyzed asymmetric insertion of carbenoids into N–H bonds represents an attractive strategy for the synthesis of optically active amines.^22,23^ We further appreciated that, while a carbene N–H insertion reaction involving fluoroalkyl-substituted α-diazo esters could provide a direct route to optically active α-trifluoromethyl amino esters, no methods have so far been reported to realize this transformation.

Previous efforts by our group and others have led to the development of hemoprotein-based biocatalysts for a growing number of abiological carbene transfer reactions,^24−26^ including carbene insertions into heteroatom–hydrogen bonds (N–H, S–H, Si–H, and B–H).^27−34^ In particular, engineered myoglobins, P450 enzymes, and artificial metalloenzymes have been reported for (nonasymmetric) N–H insertion reactions involving acceptor-only diazo esters.^27,28,32,35,36^ More recently, the first example of a biocatalytic asymmetric N–H insertion with donor–acceptor α-alkyl-substituted diazo esters was accomplished.^34^ On the other hand, laboratory-evolved variants of cytochrome *c* from *Rhodothermus marinus* have proven useful for stereoselective Si–H^30^ and B–H^31,33^ insertion reactions in the presence of donor–acceptor diazo compounds. Despite this progress, bio- and chemocatalytic methods for asymmetric carbene N–H insertions remain scarce, and none have yet been made available for the synthesis of α-trifluoromethyl amino esters. Using engineered variants of cytochrome *c*_*552*_ from *Hydrogenobacter thermophilus*, we report herein the first biocatalytic strategy for the enantioselective carbene N–H insertion of acceptor–acceptor alkyl 2-diazo-3,3,3-trifluoropropanoates (DTPs) (Scheme 1). Furthermore, the combination of protein engineering with substrate engineering achieved by tuning of the diazo compound is shown to provide an effective approach to achieve high enantioselectivity as well as enantiodivergence in this reaction. This biocatalytic strategy represents a sustainable and efficient approach to afford enantioenriched α-trifluoromethylated amines, which are important pharmacophores for medicinal chemistry as well as useful intermediates to obtain other valuable fluorinated building blocks such as β-trifluoromethyl-β-amino alcohols and halides.

## Results and Discussion

### Biocatalyst Screening for N–H Insertion with Ethyl α-Diazotrifluoropropanoate

In initial studies, we tested the activity of wild-type sperm whale Mb and variants thereof toward catalyzing the conversion of *p*-anisidine **1a** into α-trifluoromethyl amino ester **1b** in the presence of ethyl 2-diazo-3,3,3-trifluoropropanoate (EtDTP, **2a**), under anaerobic and reducing conditions using sodium dithionite as a reductant (Table 1, entries 2 and 4). Unfortunately, the formation of the desired product **3a** was not detected. In the presence of Mb(H64V,V68A), a highly active catalyst for N–H insertion with acceptor-only α-diazo esters,^28,35^ only trace amounts of the desired N–H insertion product were detected and no enantioselectivity was observed (Table 1, entry 3). On the basis of these results, we turned our attention to cytochrome *c*_*552*_ from *Hydrogenobacter thermophilus*,^37^ herein referred to as *Ht-Cc552*, a highly thermostable electron transfer protein (*T*_m_ > 110 °C)^38^ whose structure both in solution and in crystal form are known.^39,40^ Since the “distal” axial position of the heme *c* cofactor in this protein is occupied via coordination by a methionine residue (Met59), an M59G variant was initially designed to enhance its reactivity in the desired reaction. A similar strategy has previously proven useful for improving the carbene transfer activity of cytochrome *c* from *Rhodothermus marinus* toward silanes and borane.^30,31^ Gratifyingly, while *Ht-Cc552* exhibited no detectable activity in the N–H insertion reaction between *p*-anisidine and EtDTP, the *Ht-Cc552*(M59G) variant produced **3a** with significantly higher efficiency (33% yield), albeit with modest enantioselectivity (64:36 er) (Table 1, entry 5). Control experiments showed that the product formation was abolished when the reaction was carried out in the absence of the reductant (Na_2_S_2_O_4_) or under aerobic conditions (Table 1, entries 6 and 7), indicating that ferrous *Ht-Cc552* is the catalytically active species and that molecular oxygen inhibits the reaction, likely preventing or interfering with the formation of the iron porphyrin carbene intermediate.^41^ Importantly, the heme cofactor alone does not catalyze the N–H carbene insertion reaction (Table 1, entry 1), highlighting the critical role of the protein scaffold in promoting catalysis. Furthermore, the superior catalytic activity of *Ht-Cc552*(M59G) vs Mb(H64V,V68A) in the reaction suggested a beneficial effect of the heme *c* cofactor in *Ht-Cc552* in comparison to the heme *b* cofactor in myoglobin toward activation of the acceptor–acceptor diazo reagent EtDTP. Indeed, Mb(H64V,V68A) was previously found to catalyze the N–H functionalization of aniline with ethyl 2-diazopropanoate,^34^ which is sterically similar to but electronically different from EtDTP. This difference in reactivity with EtDTP may be ascribed, at least in part, to the more positive redox potential of the *Ht-Cc552* scaffold in comparison to the myoglobin scaffold (i.e., ∼+250 mV^42^ and +60 mV,^43,44^ respectively, vs SHE). Indeed, our group recently demonstrated that myoglobin-based carbene transferases featuring increased redox potentials as a result of structural alterations to the heme cofactor and the first metal-coordination sphere enhanced reactivity toward the cyclopropanation of electron-deficient substrates.^44^ Consistent with this hypothesis, we determined experimentally that wild-type *Ht-Cc552* features an Fe^3+^/Fe^2+^ reduction potential (*E*°(Fe^3+^/Fe^2+^)) of +245(±2) mV (Figure S8)―in excellent agreement with a prior literature value^42^―whereas the *Ht-Cc552*(M59G) variant features an even higher *E*°(Fe^3+^/Fe^2+^) value that exceeds +300 mV (Figure S8), as estimated on the basis of the upper limit for measurable *E*°(Fe^3+^/Fe^2+^) values using the spectrophotochemical method applied for these analyses.^45^ Using the same method, the reduction potential (*E*°(Fe^3+^/Fe^2+^)) of Mb(H64V,V68A) was previously determined to be +54 mV.^44^ Thus, in addition to facilitating access of the diazo reagent to the active iron center of the heme *c* cofactor, the beneficial M59G mutation could favor the reaction by shifting the redox potential of the metalloprotein toward a more positive value.

**Table 1:**
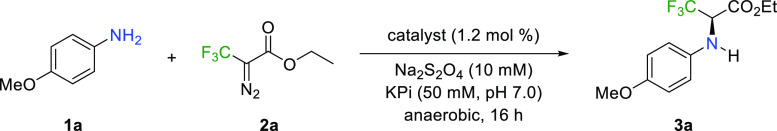
Catalytic Activity and Enantioselectivity of Mb, *Ht-Cc552* Variants, and Hemoproteins for N–H Carbene Insertion of *p*-Anisidine with EtDTP (**2a**)

entry	catalyst	conditions^a^	yield (%)^b^	TON^b^	*er*^b^
1	hemin^c^	std	0	nd	nd
2	wt Mb	std	0	nd	nd
3	Mb(H64V,V68A)	std	2	2	50:50
4	*Ht-Cc552*	std	0	nd	nd
5	*Ht-Cc552*(M59G)	std	33	27	64:36
6	*Ht-Cc552*(M59G)	no reductant	0	nd	nd
7	*Ht-Cc552*(M59G)	aerobic	1.4	1	nd

a Standard reaction conditions: 5 mM *p*-anisidine (**1a**), 10 mM EtDTP (**2a**), 60 μM (1.2 mol %) catalyst in KPi buffer (50 mM, pH 7.0), 10 mM Na_2_S_2_O_4_, rt, 16 h, under an argon atmosphere. nd = not determined.

b Yield, TON, and enantiomeric ratios based on chiral supercritical fluid chromatography (SFC) analysis using calibration curves generated from racemic analytical standards.

c 15% DMF.

### Protein Engineering of *Ht-Cc552* Variants for Enantioselective N–H Insertion

**Figure 1:**
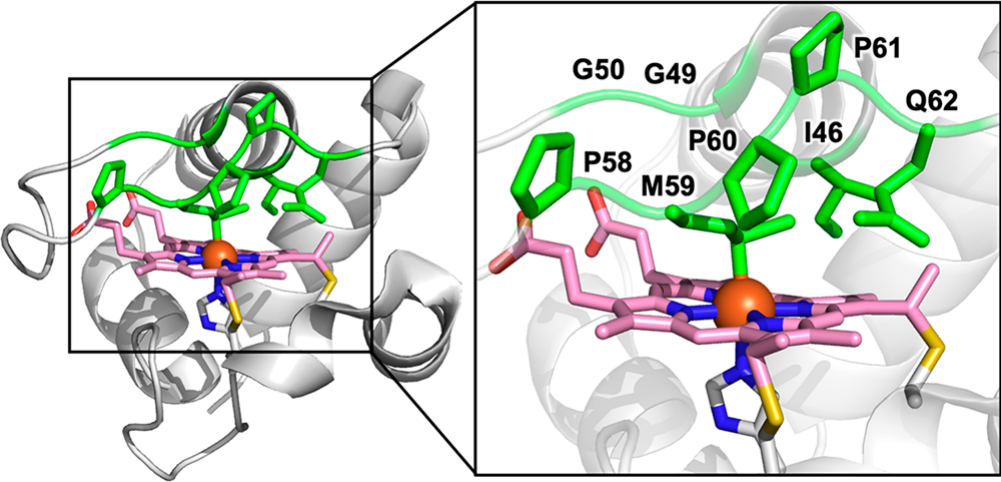
X-ray crystal structure of *Ht-Cc552* (PDB 1YNR) shown as a ribbon model. Amino acid residues targeted for site-saturation mutagenesis are highlighted in green, and the heme *c* cofactor is shown in pink.

**Figure 2:**
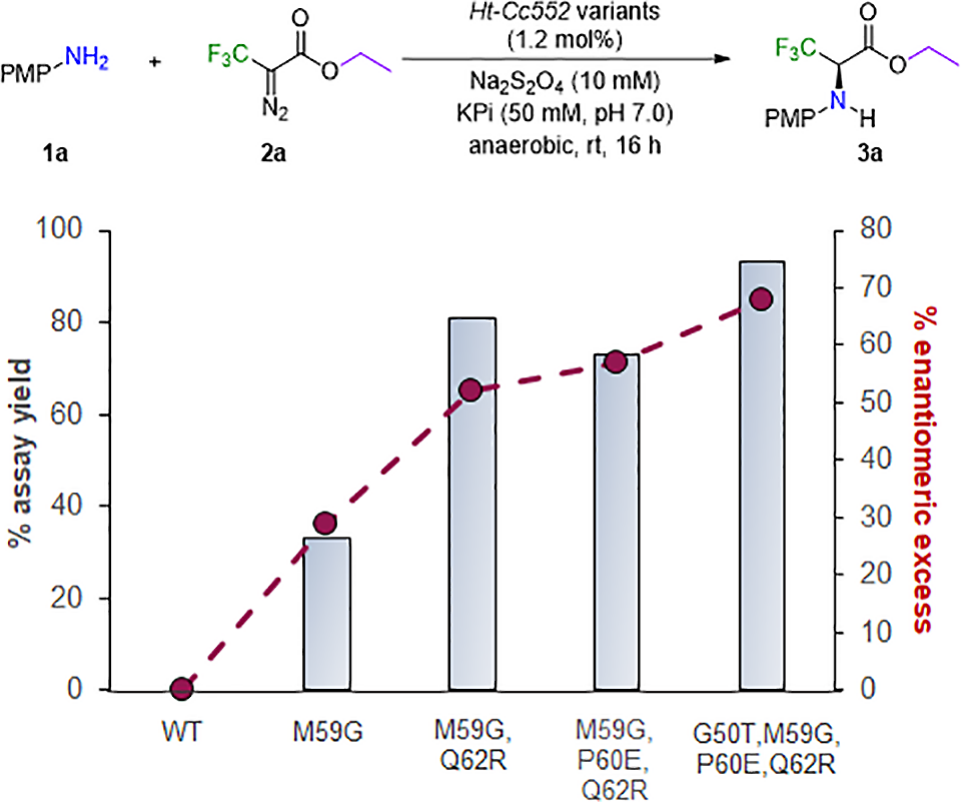
Directed evolution of *Ht-Cc552* for the enantioselective N–H insertion of *p*-anisidine with EtDTP. Reactions were carried out using 60 μM (1.2 mol %) purified *Ht-Cc552* variants, 5 mM **1a**, and 10 mM **2a**, in KPi buffer (50 mM, pH 7.0) at room temperature under anaerobic conditions (=standard reaction conditions or s.r.c.).

On the basis of these initial results, *Ht-Cc552*(M59G) was selected as a promising starting point for further development of a biocatalyst for this reaction via protein engineering. To this end, we created and screened an “active-site mutational landscape” library that sampled all 19 possible amino acid substitutions at positions Pro60, Pro61, and Gln62, which reside within a loop region above the heme pocket (Figure 1). From these libraries, a Q62R mutation (i.e., *Ht-Cc552*(M59G,Q62R)) was found to be particularly beneficial to improve both the efficiency (33% → 81% yield) and enantioselectivity (64:36 vs 76:24 er) of the metalloprotein over the parental sequence (Figure 2 and Figures S1–S3 in the Supporting Information). Using *Ht-Cc552*(M59G,Q62R) as the parent, the introduction of the P60E mutation induced a further improvement in enantioselectivity (78:22 er; Figure 2 and Figures S1–S3 in the Supporting Information), without affecting the catalytic activity, resulting in the identification of *Ht-Cc552*(M59G,P60E,Q62R) as a significantly improved biocatalyst for the N–H insertion reaction over *Ht-Cc552*(M59G). Next, the amino acid positions Ile46, Gly49, and Gly50, which reside within the inner side of the heme pocket (Figure 1), were targeted for site-saturation mutagenesis, due to their close proximity to the heme *c* cofactor. From these libraries, the improved variant *Ht-Cc552*(G50T,M59G,P60E,Q62R) was identified that is able to produce the desired α-trifluoromethyl amino ester **3a** in nearly quantitative yield (93%) and with further improved enantioselectivity (81:19 er) (Figure 2 and Figure S4 in the Supporting Information).

### Tuning of *Ht-Cc552* Variant Enantioselectivity via Diazo Reagent Engineering

**Scheme 2:**
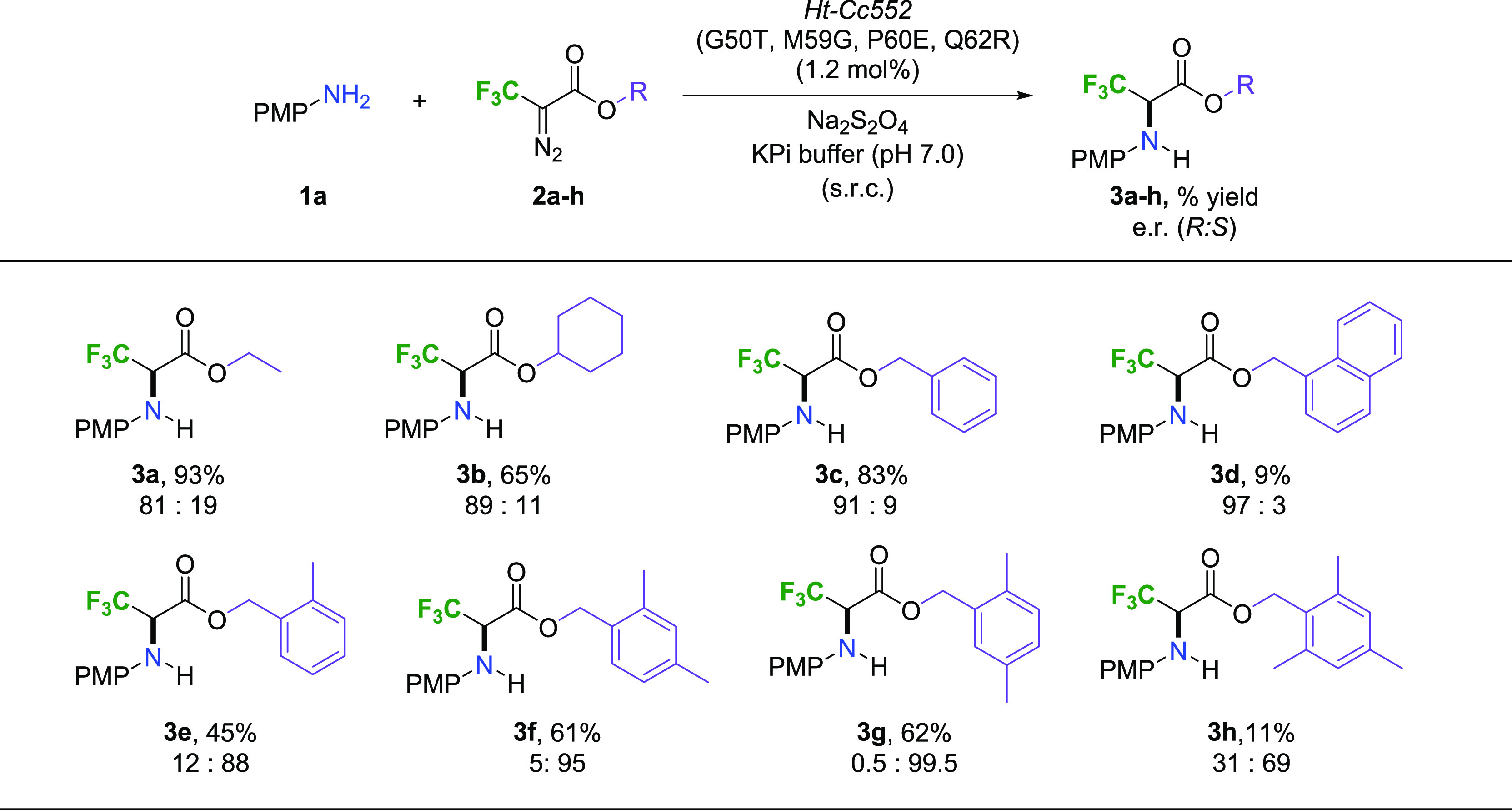
Tuning of *Ht-Cc552*(G50T,M59G,P60E,Q62R) Enantioselectivity via Diazo Reagent Engineering s.r.c. = Standard reaction conditions as in Figure 2. The product conversion and stereoselectivity were determined by chiral SFC using a calibration curve with authentic standards.

In previous studies, we found that re-engineering of the diazo reagent can furnish a valuable and complementary strategy (to protein engineering) for fine-tuning the enantioselectivity of carbene transfer biocatalysts.^34,46^ Armed with this knowledge, we investigated the possibility of increasing the enantioselectivity of the *Ht-Cc552*(G50T,M59G,P60E,Q62R)-catalyzed N–H insertion reaction by varying the alkyl ester group in the diazo reagent, with the goal of exploiting beneficial steric interactions between this group (e.g., in the heme-bound carbene intermediate) and the surrounding protein residues. To this end, we developed an efficient and versatile synthetic route to afford 2-diazo-3,3,3-trifluoropropanoate esters from inexpensive trifluoroacetic acid and *p*-anisidine (Scheme S1) and applied this protocol to produce a diverse set of DTP carbene donors (compounds **2b**–**h**, Scheme 2) bearing ester groups of varying size (e.g., **2d** vs **2c**), bulk (e.g., **2b** vs **2c**), or substitution patterns on the benzyl ring (e.g., **2e** vs **2g** vs **2h**).

To our delight, a notable improvement in the enantioselectivity of the reaction with *Ht-Cc552*(G50T,M59G,P60E,Q62R) was obtained upon substitution of the ethyl group in the EtDTP reagent with a larger group (i.e., a cyclohexyl (**2b**), benzyl (**2c**), or naphthyl group (**2d**)), resulting in the formation of the desired N–H insertion product in up to 97:3 enantiomeric ratio (**3b**–**d** vs **3a**, Scheme 2). Further analysis of **3b**–**d** via chiral chromatography and other control experiments (see the Supporting Information for details) showed that they share the *R* configuration of **3a**. Thus, across this compound series, the increase in *R* enantioselectivity was found to correlate largely with the increasing size of the ester group in the carbene donor reagent (**3d** > **3c** ≈ **3b** > **3a**). In consideration of the higher enantioselectivity but comparably high reactivity vs EtDTP (83% vs 93% yield), the benzyl ester derivative **3c** was chosen as the optimal reagent for the formation of the *R* enantiomer of the N–H insertion product. A variation of the reaction conditions did not lead to a further improvement in yield and/or enantioselectivity (Table S1). Additional experiments demonstrated that *Ht-Cc552*(G50T,M59G,P60E,Q62R) supports up to 752 total turnovers under catalyst-limited conditions (entry 11, Table S1) and it catalyzes the reaction with an initial product formation rate of 8 and 1.5 TON min^–1^ in the presence of EtDTP and BnDTP, respectively (Figure S5). Under the optimized reaction conditions, no significant loss in the Soret band of the protein (<10%) or protein precipitation was noted during the reaction (Figure S6), indicating that destruction of the heme cofactor does not play a major role in limiting the catalyst performance and that the biocatalyst may be recyclable, an aspect that will be investigated as part of future studies.

Intriguingly, substitution of the benzyl group in the diazo reagent with one or two methyl groups (**2e**–**h**) led to a complete switch of the biocatalyst’s enantioselectivity to give the corresponding *S*-configured N–H insertion products **3e**–**h** in up to 99% enantiomeric excess (**3g**, Scheme 2). On the basis of its superior performance in terms of both enantioinduction and yield, the 2,5-dimethylbenzyl-containing diazo compound **2g** was selected as the optimal carbene donor reagent for favoring *S* enantioselectivity in the *Ht-Cc552*(G50T,M59G,P60E,Q62R)-catalyzed N–H insertion reaction. Altogether, these studies highlighted the value of combining protein engineering of the metalloprotein scaffold with diazo substrate engineering for both tuning and inverting the enantioselectivity of a carbene transferase enzyme. Indeed, while it has been previously possible to obtain enantiocomplementary carbene transfer biocatalysts by re-engineering of the enzyme,^34,47,48^ to our knowledge this is the first example in which enantiodivergence has been achieved within a single enzyme through engineering of the diazo reagent.

### Substrate Scope of *R* and *S* Enantioselective N–H Insertion Reactions with *Ht-Cc552*(G50T,M59G,P60E,Q62R)

**Scheme 3:**
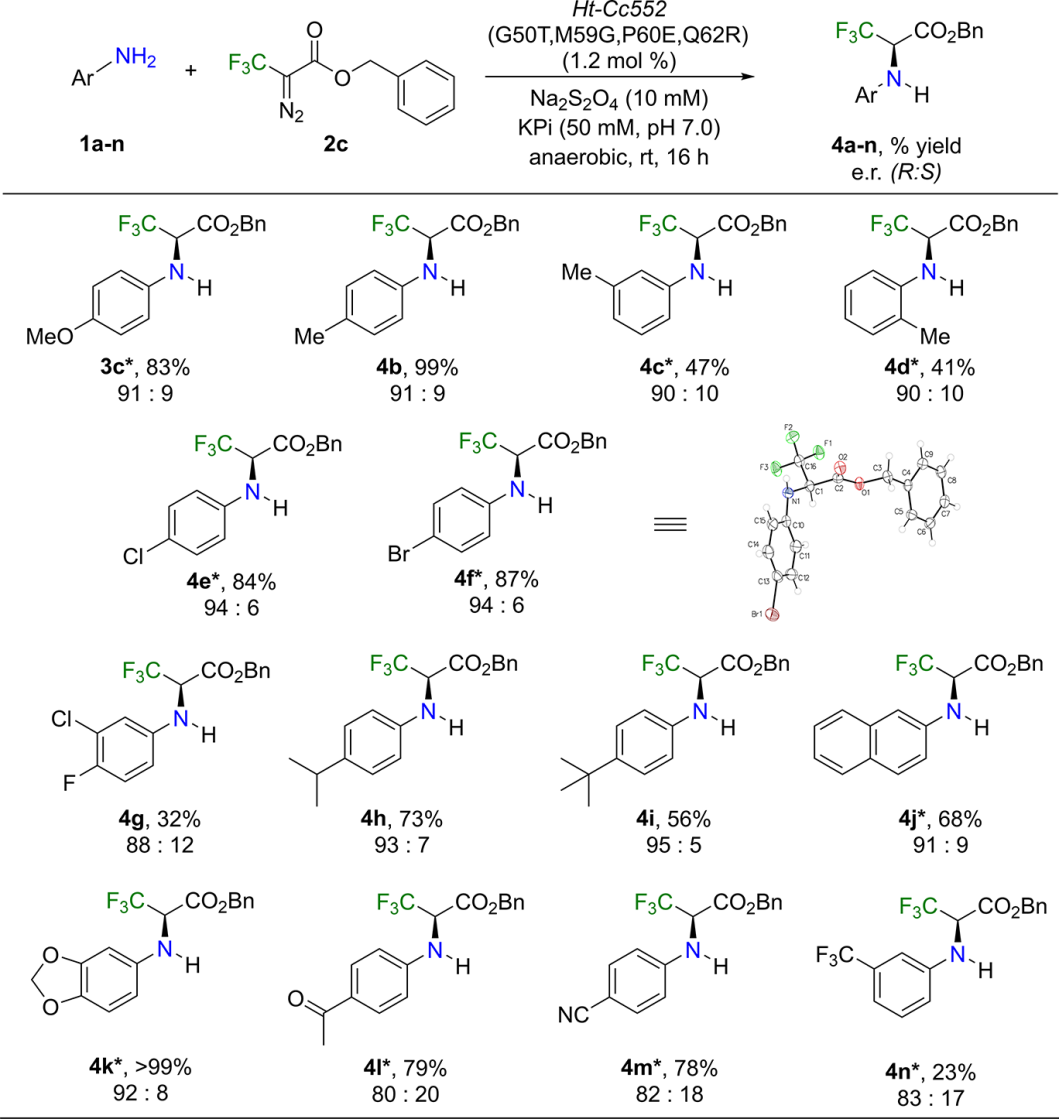
Substrate Scope of *Ht-Cc552*-Catalyzed *R*-Enantioselective N–H Insertion Reaction in the Presence of BnDTP (**2c**) s.r.c. = standard reaction conditions as in Figure 2. Asterisks denote 5% DMF. The product conversion and stereoselectivity were determined by chiral SFC and GC using a calibration curve with authentic standards.

To explore the substrate scope of the *Ht-Cc552*(G50T,M59G,P60E,Q62R) biocatalyst in the *R*-enantioselective mode, the enzyme was challenged with a panel of aniline derivatives and other aryl amines in the presence of benzyl 2-diazotrifluoropropanoate **2c** (Scheme 3). Notably, variously substituted anilines, including *para* (**1a**,**b**,**e**–**i**,**l**,**m**)-, *meta* (**1c**,**n**)-, and *ortho*-substituted (**1d**) anilines were readily accepted by the *Ht-Cc552* variant to produce the desired α-trifluoromethyl amino esters **3c**, **4b−f** in good to quantitative yields (41–99%) and with high enantioselectivity (90:10 to 95:5 er) (Scheme 3). A doubly substituted aniline substrate such as 3-chloro-4-fluoroaniline (**1g**) was also converted to the corresponding N–H insertion product **4g** with good enantioselectivity (88:12 er), albeit in more modest yield (32%). The reactions with aniline derivatives carrying electron-withdrawing groups or large substituents in the *para* position generally, albeit not always (e.g., **4l**,**m**), displayed higher levels of enantioselectivity as indicated by the results with **4e**,**f** (94:6 er), **4h** (93:7 er), and **4i** (95:5 er) in comparison to **3c** and **4b**. An opposite trend was observed for *meta*-substituted anilines (**4n** vs **4c**). Notably, keto- and nitrile-functionalized anilines could be readily converted to the desired α-trifluoromethyl amine derivatives (**4l**,**m**), despite the potential reactivity of the keto group with the amine substrate and the tendency of nitrile compounds to bind metal centers, respectively. Substrates **1j**,**k** were also tested to explore the scope of the reaction across other aromatic amines. These naphthyl- and benzo[*d*][1,3]dioxole-substituted amines were both accepted to give the desired N–H insertion products **4j**,**k**, respectively, in high yields (68–99%) and in high enantiomeric ratios (91:9 to 92:8 er), further demonstrating the broad substrate profile of the engineered *Ht-Cc552*-based biocatalyst.

Importantly, in all of these reactions the N–H insertion products were obtained with *R* enantioselectivity, highlighting the conserved and predictable enantiopreference of the biocatalyst under the applied conditions. It is also worth noting that no formation of the dimerization byproducts of BnDTP, dibenzyl 2,3-bis(trifluoromethyl)fumarate and maleate, or double-insertion products were detected in these reactions, further indicating that these biocatalytic transformations proceed with high chemoselectivity. Furthermore, a large-scale reaction using 4-bromoaniline (**1f**) and **2c** was carried out to obtain 60 mg of the α-trifluoromethyl-amino ester **4f** in 75% isolated yield, supporting the scalability of the methodology. Product **4f** was crystallized and determined to have an *R* absolute configuration by X-ray diffraction analysis (Scheme 3), serving as a reference for the stereochemical assignment of the other products and their corresponding enantiomers.

### *S*-Enantioselective N–H Insertion Reactions via a Diazo Reagent Switch

**Scheme 4:**
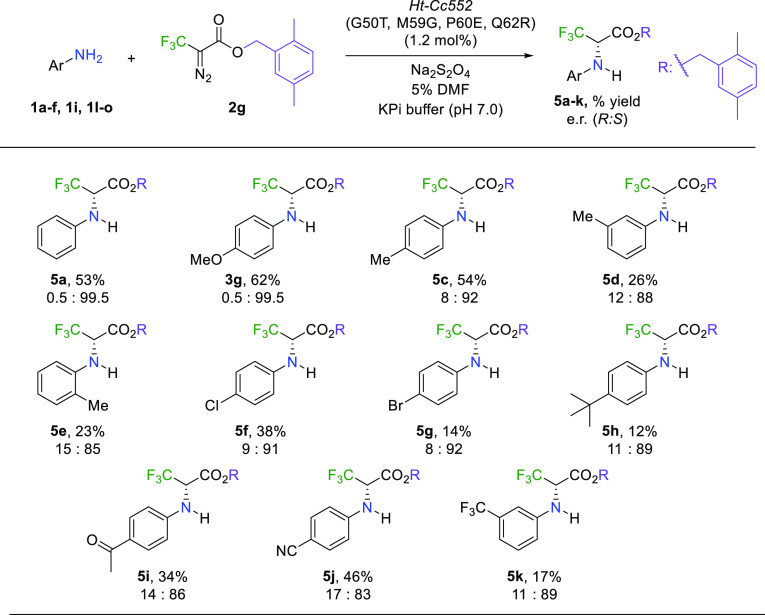
Substrate Scope of *Ht-Cc552*-Catalyzed *S*-Enantioselective N–H Insertion Reaction in the Presence of (2,5-Dimethyl)benzyl 2-Diazotrifluoropropanoate (**2g**) s.r.c. = standard reaction conditions as in Figure 2. The product conversion and stereoselectivity were determined by chiral SFC and GC using a calibration curve with authentic standards.

To probe the substrate scope of the *Ht-Cc552* biocatalyst in the *S*-selective mode, representative samples of the aniline derivatives described in Scheme 3 were then tested by applying the same enzyme variant and identical reaction conditions but in the presence of 2,5-dimethylbenzyl 2-diazotrifluoropropanoate (**2g**) as the carbene donor reagent, instead of BnDTP (Scheme 4). Gratifyingly, the desired *S*-configured N–H insertion products **5a**–**k** were obtained in all cases, showing that the diazo-substrate-induced inversion of enantiopreference is broadly maintained across the different aniline substrates. Although the yields of these reactions were generally lower than observed for the *R*-selective counterparts (34% vs 68% average yield), they all proceeded with good to excellent enantioselectivity, resulting in the formation of the *S*-configured products in enantiomeric ratios ranging from 17:83 to 0.5:99.5 (Scheme 4). In addition to the inverted enantioselectivity, distinct structure activity trends were also noted for the two enantiodivergent transformations. For example, whereas aniline derivatives with bulky groups at the *para* position were well tolerated in the *R*-selective mode with BnDTP, these represented more challenging substrates for the *S*-enantioselective variant of the reaction in the presence of **2g**, in particular in comparison to other *para*-substituted analogues (e.g., 12–34% yield for **5h**–**i** vs 38–62% for **3g**, **5c**,**f**,**j**). This difference clearly suggested distinct steric requirements with respect to the amine substrate during the *Ht-Cc552*-catalyzed reaction in the two enantiodivergent fashions. Overall, the results summarized in Schemes 3 and 4 demonstrate the utility of the engineered *Ht-Cc552* biocatalyst toward obtaining α-trifluoromethyl-α-amino esters in both enantiomeric forms.

### Origins of Enzyme-Controlled Enantioselectivity

**Figure 3:**
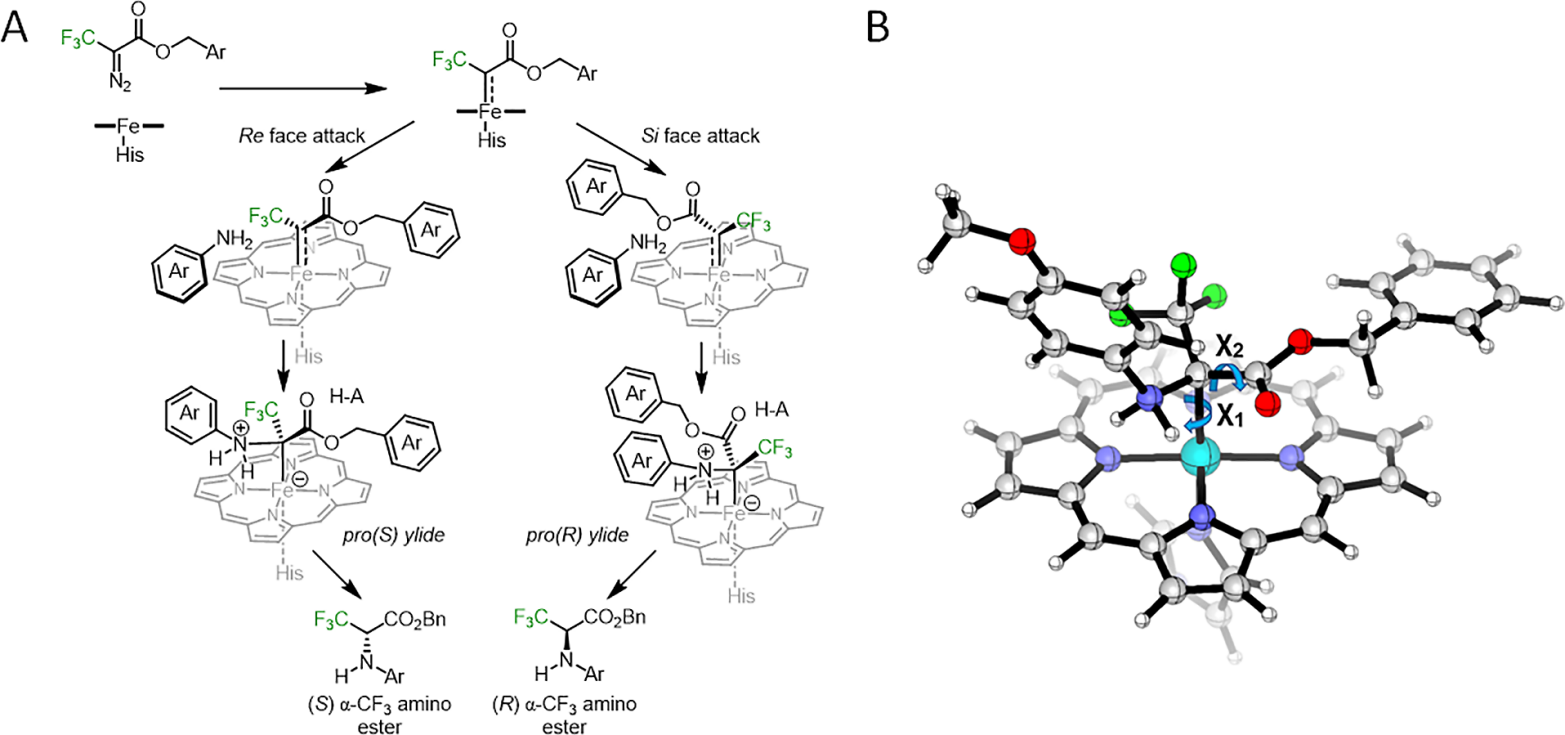
(A) Stereochemical model for enantioselective N–H insertion reaction catalyzed by *Ht-Cc552* variants. (B) DFT-calculated model of the pro*S*(−) heme-bound ylide reaction intermediate.

Computational studies were performed to better understand the role of the metalloprotein scaffold in controlling the enantioselectivity of the reaction as well as the nature of the carbene donor reagent-induced switch in enantioselectivity. Similarly to carbene S–H insertion,^29^ heme-catalyzed carbene N–H insertion was previously proposed to proceed *via* the formation of an iron ylide complex generated by nucleophilic attack of the amine substrate to the iron porphyrin carbene intermediate,^41^ followed by protonation of the ylide to give the N–H insertion product.^49^ By analogy with other metal-catalyzed N–H insertions,^50−54^ the stereochemical outcome of this reaction can be determined by the facial selectivity of amine attack to the metallo-carbene species during formation of the metal-bound ylide intermediate and/or during protonation of the dissociated ylide. In the present system, divergent enantioselectivity was obtained using the same enzyme variant in the presence of diazo reagents with varying steric bulk, indicating that enantioselectivity is primarily dictated during the formation of the heme-bound ylide intermediate, followed by a conserved mechanism for protonation of the pro*S* or pro*R* heme-ylide complex on the solvent-exposed side of these species (Figure 3A). Reasonably, the protonation step could be mediated by the protein matrix (i.e., by amino acid residues proximal to the heme cofactor) or directly from the solvent.

According to this mechanistic scenario, we performed density functional theory (DFT) calculations on the iron porphyrin bound ylide intermediate formed by the reaction with the **2c**-derived iron porphyrin carbene intermediate and *p*-methoxyaniline (**1a**). We computed the structures and energies for four conformations of the iron porphyrin ylide complex leading to either *R* or *S* enantioselectivity, and the orientation of the ester moiety (indicated by + or −) with respect to the porphyrin group plane (see the Experimental Procedures, Figure 3B, and Figure S10). The computed energy differences between the different conformations (pro*R*(+), pro*R*(−), pro*S*(+), and pro*S*(−)) of the porphyrin-bound ylide intermediate are within 1.7 kcal/mol (Figure S10), confirming that the experimentally observed enantioselectivity of the enzymatic reactions is derived from differential stabilization of these conformations within the enzyme active site.

**Figure 4:**
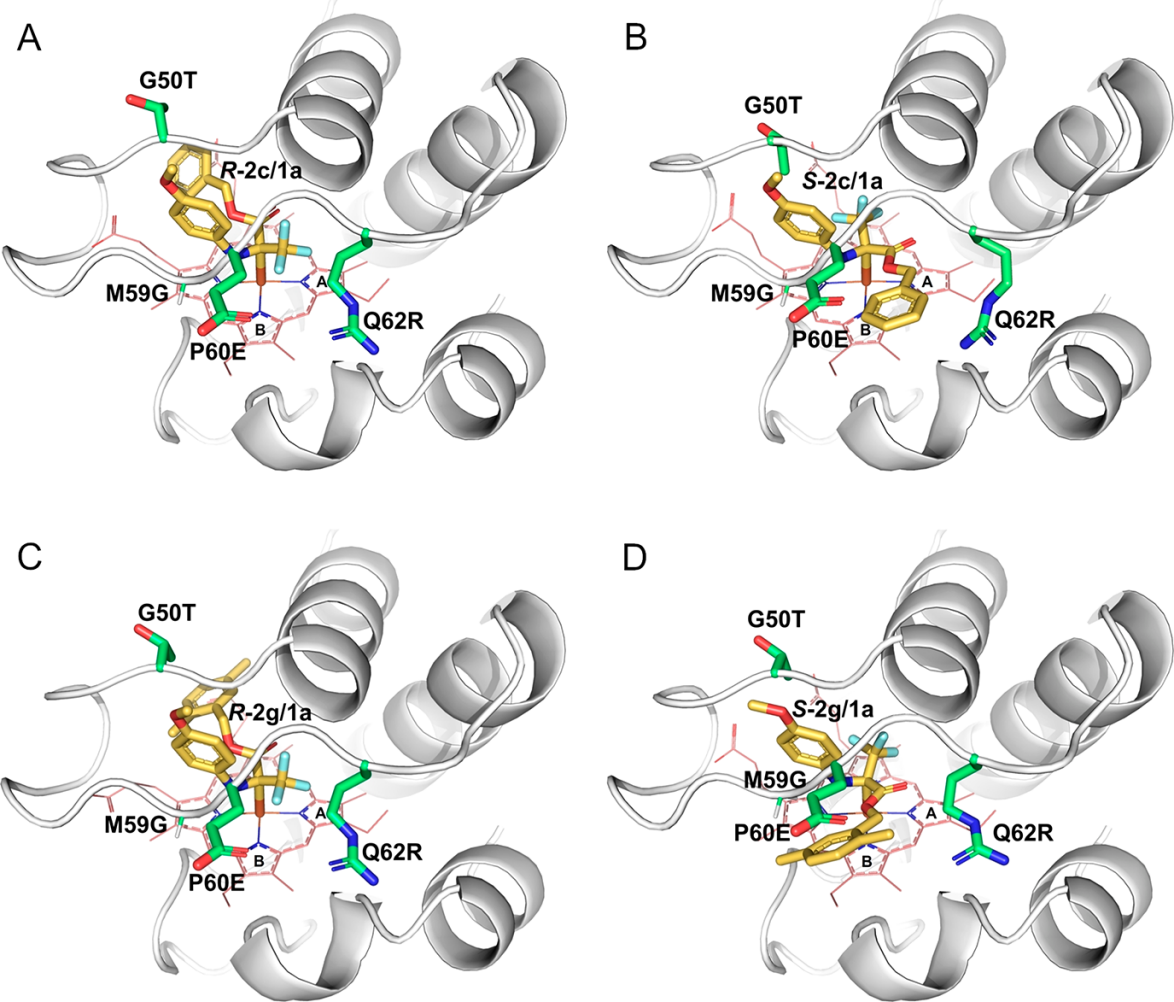
Models of the engineered *Ht*-*Cc552*(G50T,M59G,P60E,Q62R) variant in complexes with the (A) pro*R*-rot3(−) and (B) pro*S*-rot1(+) structures of the **2c**/**1c**-derived ylide intermediate and in complexes with the (C) pro*R*-rot3(−) and (D) pro*S*-rot1(+) structures of the **2g**/**1c**-derived ylide intermediate. The pro*R* configuration is favored in the presence of the **2c**-derived ylide, while the pro*S* configuration is favored in the case of the **2g**-derived ylide, explaining the diazo-substrate-induced switch in enantioselectivity.

To gain insight into the origin of the enantiopreference endowed by the engineered *Ht-Cc552* biocatalyst, we generated models for the hemoprotein-bound ylide complexes in the various *Ht-Cc552* variants using the Rosetta software suite.^55^ Specifically, we superimposed DFT-calculated Fe ylide models onto the available crystal structure of *Ht-Cc552*,^40^ introduced amino acid substitutions present in *Ht*-*Cc552*(G50T,M59G,P60E,Q62R), and optimized the structure and energy of the resulting protein–ligand complexes. Carbenes from the diazo reagents giving high activity (>50% yield) in the N–H insertion reaction were included in our calculations (Table 2, column 2) and used in the modeling. As the DFT calculations were performed with the porphyrin, which is *C*_4_ symmetric (unlike heme), each of the four DFT-generated porphyrin-bound ylide intermediates (i.e., pro*R*(+), pro*R*(−), pro*S*(+), and pro*S*(−)) can be superimposed into the protein active site in four ways by aligning to one of four heme group rings (A–D, Figure 4), resulting in 16 different arrangements for each substrate (named pro*R*-rot1(+), pro*R*-rot1(−), pro*S*-rot1(+), pro*S*-rot1(−), pro*R*-rot2(+), etc.; see the Experimental Procedures for details). The lowest energy arrangement is predicted to correspond to the preferred enantiomer generated by the enzyme. As shown in Table 2, Rosetta-based energy calculations are able to qualitatively recapitulate the enantiopreference of the engineered *Ht-Cc552*-catalyzed N–H insertion in the presence of the different diazo reagents. For both **2c** and **2a**, the pro*R* configurations of the corresponding protein ylide complexes lie 2–10 Rosetta energy units (REUs) lower in energy than the pro*S* configurations, which recapitulate well the *R* selectivity of the enzyme in the reactions with these diazo compounds. Similarly, the protein ylide complexes derived from **2e**–**g** in the pro*S* configurations are favored by 15–22 REU in comparison to the pro*R* counterparts, which is consistent with the *S* enantiopreference of the *Ht*-*Cc552*(G50T,M59G,P60E,Q62R)-catalyzed reactions in the presence of these diazo reagents. The *S* enantiopreference of the N–H insertion reaction with **2h** was also correctly predicted from a qualitative standpoint (Table 2), even though the energy differences between the various binding poses corresponding to the different pro*S* and pro*R* configurations do not fully correlate with the lower enantioselectivity observed experimentally for this reaction in comparison to those with the related diazo reagents **2e**–**g** (i.e., **3h** vs **3e**–**g**; Scheme 2). Notably, however, the calculated energy values of all configurations of the **2h**-derived complexes were found to be significantly higher in comparison to the corresponding complexes with **2e**–**g** (Table 2). This uniform destabilization suggests a less favorable fit of the **2h**-derived ylide, in comparison to the analogous ylide complexes derived from **2e**–**g**, into the near-native conformation of the protein used in our modeling. This general destabilization may explain, at least in part, the lower yield observed in the N–H reaction with this reagent (11%) in comparison to the reactions with **2e**–**g** (11% vs 45–62% yield; Scheme 2).

**Table 2:** Rosetta-Calculated Energies of the Engineered *Ht-Cc552*(G50T,M59G,P60E,Q62R) Variant Complexed with **2c**/**1c**-Derived Heme Ylide Intermediates in the pro*R* or pro*S* Configurations and +/– Conformations^a^

entry	diazo	yield (%)	er (*R*:*S*)	*proR*-rot2 (−)	*proR*-rot3 (−)	*proS*-rot1 (+)	*proS*-rot1 (−)
1	**2a**	93	81:19	–202.46	**-220.89**	–210.93	-211.69
2	**2c**	83	91:9	–183.27	**-207.24**	-205.47	–193.99
3	**2e**	45	12:88	-189.69	–187.12	**-204.22**	–196.13
4	**2f**	61	5:95	-190.77	–122.31	**-206.28**	–130.91
5	**2g**	62	0.5:99.5	-184.77	–157.02	**-205.61**	–198.40
6	**2h**	11	31:69	-169.76	–110.24	**-185.70**	–68.12

a The lowest energy value is highlighted in boldface, while the energy of the most competitive state is underlined. For complete data, see Table S2. Energies are reported in Rosetta energy units (REUs).

Having established that Rosetta-based energy calculations can recapitulate the enantiopreference of *Ht*-*Cc552*(G50T,M59G,P60E,Q62R), we further inspected the protein and ylide complex models to gain insights into the structural features underlying its higher catalytic activity and enantioselectivity in comparison to the wild type protein. In wild-type *Ht*-*Cc552* (Figure 1), the sulfur atom of the Met59 side chain coordinates to the iron center of the heme cofactor, which likely prevents an interaction of the diazo reagent with the heme center, resulting in the lack of catalytic activity observed experimentally (Figure 2). In contrast, the M59G mutation in the engineered *Ht*-*Cc552* variant creates an open coordination site at the heme iron center, which enables binding and activation of the diazo reagent to form the heme carbenoid intermediate, inducing a major increase in N–H insertion reactivity (Figure 2 and Table 1, entry 5 vs entry 4). The M59G mutation also creates a cavity above ring B of the heme *c* cofactor (Figure 4A,B), so that the aniline substrate can more readily approach the *Si* and *Re* faces of the rot3 and rot1 **2c**-derived carbenoid, respectively, to form pro*R*-rot3(−) or pro*S*-rot1(±) heme ylide complexes. In combination with the M59G mutation, the P60E mutation increases the backbone flexibility of the active site loop to better accommodate the aniline substrate.

In the pro*R* configuration of the **2c**-derived ylide complex of *Ht*-*Cc552*(G50T,M59G,P60E,Q62R), the benzyl ester group projects inward into the buried side of the distal cavity of the heme *c* cofactor (Figure 4A), whereas the trifluoromethyl group is oriented outward. The aliphatic side chain of Arg62 (introduced via the beneficial Q62R mutation, Figure 2) is packed against the trifluoromethyl group (∼3 Å), and its guanidium group is positioned at an interacting distance (∼5 Å) from the carboxylate group of Glu60, introduced via the P60E mutation. Thr50, introduced by the beneficial G50T mutation during the last round of the enzyme evolution process (Figure 2), makes apolar contacts (∼3 Å) with the benzyl ester group. This arrangement results in a “closed” structure that buries most of the ylide complex from the solvent (Figure 4A). In the pro*S* arrangement of the same complex, the benzyl ester group is directed outward and toward the solvent, whereas the trifluoromethyl group is oriented inward (Figure 4B). In this arrangement, Thr50 makes no contacts with the ylide complex and the side chain of Arg62 is “pushed away” from the benzyl group. The latter group also physically separates the Arg62/Glu60 ionic pair and brings their ionized side-chain groups at a greater distance than in the pro*R* complex (∼10 vs 5 Å). In both complexes, the aniline group adopts a similar position, making close contacts with the stem region of the long Met60-bearing loop (i.e., residues 49–50 and 58–59; Figure 4A,B). Altogether, these analyses suggest that (a) preorganization of the heme-bound carbene is critical for dictating facial selectivity of amine attack to this species to generate the ylide intermediate and (b) the pro*R* configuration is favored by the combination of steric effects and favorable van der Waals and electrostatic interactions mediated by Arg62, Glu60, and Thr50, which are disrupted (or absent in the case of Thr50) in the pro*S* configuration. Consistent with these conclusions, a per-residue energy decomposition comparison of the ylide complex models in the preferred *R*-rot3(−) arrangement versus the competing pro*S*-rot1(+) state revealed that the largest energy differences favoring the pro*R* vs pro*S* arrangement reside in contacts mediated by Arg62, Glu60, and Thr50 (Figure S12A,B), all of which have contributed to the enhancement of the *R* enantioselectivity of the enzyme during its evolution for this reaction (Figure 2).

### Origins of Reagent-Induced Enantioselectivity Switch

We next investigated how the enantiopreference of the *Ht-Cc552*-based catalyst could be completely switched to favor the *S*-configured N–H insertion product in the presence of diazo reagent **2g** (0.5:99.5 *R:S*) in comparison to the structurally related diazo reagent **2c** (91:9 *R:S*). In contrast to the **2c-**derived ylide (Figure 4A,B), the 2,5-dimethyl-substituted benzyl ester moiety of the **2g**-derived ylide in the pro*R*-rot3(−) configuration can no longer be accommodated by the inner pocket region above ring D of the heme cofactor. This state is largely destabilized by clashes with residue Thr50 (i.e., the site of G50T substitution), Leu42 and Lys45 side chains, and the propionic groups of the heme *c* cofactor (Figure 4C and Figure S12C,D). Other pro*R*-configuration states of the **2g**-derived ylide also cannot rival pro*S*-rot1(+) (Figure 4D), which can thus explain the high *S* enantioselectivity of the *Ht*-*Cc552*(G50T,M59G,P60E,Q62R)-catalyzed reaction with **2g**. For example, the most competitive state pro*R*-rot2(−) has its aniline moiety occupying an alternative cavity above ring A, breaking the hydrogen bond between the backbone atoms of Lys47 and Arg62 (Figure S11C). Its benzyl ester moiety is also destabilized by unfavorable interactions with Glu60 and Arg62 (Figure S11C), as evidenced by a per-residue energy decomposition analysis of these complexes (Figure S12E,F). In addition to enantiodivergence, the lower energy of the dominant state of the ylides derived from the less bulky diazo esters (**2a**,**c**,**h** vs **2e**–**g**; Table 2) tend to correlate with the higher yields of the corresponding reactions (Scheme 2), although other factors can contribute to these differences. Thus, these analyses show that a combination of structural and energetic factors, mediated by all four beneficial mutations as well as other residues surrounding the heme *c* cofactor, contribute to destabilize the pro*R* configurations of the **2g**-derived ylide over the pro*S* state, resulting in the dramatic switch in enantioselectivity observed experimentally in the *Ht-Cc552*(G50T,M59G,P60E,Q62R)-catalyzed N–H insertion reactions with **2g**.

### Diversification of α-Trifluoromethyl Amine Products

**Scheme 5:**
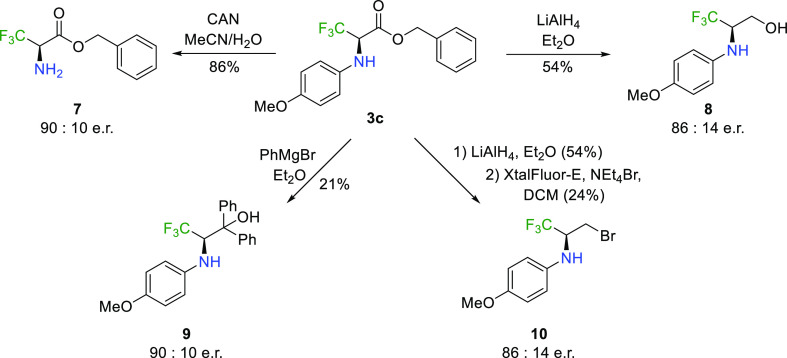
Chemoenzymatic Synthesis of Enantioenriched α-Trifluoromethyl Amine Derivatives

To further demonstrate the synthetic value of the present biocatalytic strategy, asymmetric *Ht-Cc552*-catalyzed N–H carbene insertion with DTPs was leveraged to enable the chemoenzymatic synthesis of various α-trifluoromethylated amine derivatives (Scheme 5), which are highly sought after motifs for medicinal chemistry and drug discovery.^56^ For example, benzyl-protected α-trifluoromethylamino acid **7** was synthesized in high yields and in a highly enantioenriched form (86% yield, 90:10 er) by treating enzymatically produced **3c** with ceric ammonium nitrate (CAN) (Scheme 5). α-Trifluoromethylated amino acids are valuable noncanonical amino acids^8,9^ that find applications in the design of peptidomimetics and peptide-based drugs.^7^ On the other hand, medicinally valuable β-trifluoromethyl-β-amino alcohols such as **8** and **9** could be obtained via reduction of the enzymatic N–H insertion product **3c** in the presence of lithium aluminum hydride (LAH) to give **8** in good yield and enantioenrichment (54% yield, 86:14 er) and via nucleophilic arylation of **3c** with Grignard reagents to give **9** with no erosion in enantiopurity (9:1 er) (Scheme 5). Finally, LAH reduction of **3c** followed by exposure to XtalFluor-E and tetraethylammonium bromide afforded the trifluoromethylated β-amino alkyl bromide **10** in enantioenriched form (86:14 er; Scheme 5).

## Conclusions

In conclusion, we developed a biocatalytic platform for the asymmetric synthesis of α-trifluoromethyl amines via an abiological N–H carbene insertion. Cytochrome *c*_*552*_ from *Hydrogenobacter thermophilus* was engineered into a selective biocatalyst for the enantioselective N–H insertion of aryl amines with acceptor–acceptor 2-diazotrifluoropropanoates, a reaction with no reported chemocatalytic counterpart. While active site mutations around the heme *c* cofactor have proven useful to improve the activity and enantioselectivity of this biocatalyst, a further boost in enantioselectivity as well as complete inversion of its enantiopreference could be achieved through engineering of the diazo reagent. In combination with DFT calculations, Rosetta-based molecular modeling studies provided insights into the origin of protein-mediated control of enantioselectivity in the N–H insertion reaction, along with the factors underlying the enantioselectivity switch upon variation of the ester group in the diazo compound. The enzymatic products can be diversified to obtain a variety of medicinally relevant chiral α-trifluoromethylated amine building blocks. These studies expand the scope of abiological carbene transfer reactions catalyzed by metalloproteins and pave the way to the further development of biocatalytic strategies for the synthesis of chiral organofluorines.

## Experimental Procedures

### Reagents and Synthetic Procedures

Synthetic procedures, analytical procedures, and characterization data for the diazo reagents, N–H insertion products, and chemoenzymatic products are described in the Supporting Information.

### Cloning and Plasmid Construction

Plasmid pET22 (Novagen) was used as a cloning vector, and cloning was performed using overlap extension PCR or a modified QuikChange mutagenesis protocol.^57^ The cytochrome *c*_*552*_ variants were expressed from pET22 vectors in the presence of a second plasmid (pEC86^58^) for the coexpression of the cytochrome *c* maturation system. Primer sequences are given in Table S3. Phusion DNA polymerase, dNTP mix, and *Dpn* I restriction enzyme were purchased from New England Biolabs. Chemically competent *E. coli* DH5α cells were used for molecular cloning, and E. coli C41(DE3) cells were used for protein expression. Transformed cells were grown using Terrific Broth medium supplemented with 100 μg/mL of ampicillin and 34 μg/mL of chloramphenicol (TB_amp/cam_). The *HtCc552* variants discussed in this work were cloned and expressed using a pET22(b)+ vector (Novagen). A plasmid encoding for *Hydrogenobacter thermophilus* cytochrome *c*_*552*_ (*Ht-Cc552*) was a gift from Prof. Kara Bren (University of Rochester). *Ht-Cc552* was subcloned into pET22(b)+ using overlap extension PCR between restriction sites *Nde*I and *Xho*I with an *N*-terminal peptide leader sequence from *T. versutus* cytochrome *c*_550_ (MKISIYATLAALSLALPAVA) to ensure proper periplasmic maturation^59^ and a *C*-terminal 6xHis-tag. This recombinant plasmid was cotransformed with the cytochrome *c* maturation plasmid pEC86 into *E. coli* C41(DE3) chemically competent cells.

### Library Construction

Site-saturation mutagenesis libraries were constructed using a modified “small-intelligent” focused mutagenesis protocol.^60^ To create a library for a targeted amino acid residue site, a forward mutagenizing primer and the reverse partially complementary mutagenizing primer were designed containing the degenerate codons NDT, VMA, ATG, and TGG. The four forward primers containing the degenerate codons NDT, VMA, ATG, and TGG were mixed together in a 12:6:1:1 ratio, respectively, and the reverse primers were separately mixed in the same ratio. The forward and reverse primer mixes were used to carry out site-saturation mutagenesis PCR using a modified QuikChange mutagenesis protocol. The PCR products were treated with *Dpn* I to digest the parental plasmid, and 5 μL of the reaction mixture was used to transform *E. coli* DH5α chemically competent cells. After the transformed cells were plated on a LB_amp_ agar plate, 60 single colonies were individually picked and grown in 5 mL LB_amp_ overnight cultures, and their plasmids were extracted and sequenced using a T7 forward universal primer (ACGT, Inc.).

### Protein Expression and Purification

After transformation of the pET22(b)+ plasmid with the desired *Ht-Cc552* gene and the pEC86 plasmid into *E. coli* C41 (DE3) chemically competent cells and plating onto a LB_amp/cam_ agar plate, single colonies were picked and used to inoculate an overnight culture (LB_amp/cam_, 5 mL). One liter of TB_amp/cam_ in a 2 L flask was inoculated with the overnight culture and shaken at 37 °C (200 rpm) until an OD_600_ value of ∼0.8 was reached (approximately 5 h). The cell cultures were induced with 0.5 mM isopropyl-β-d-1-thiogalactopyranoside (IPTG), supplemented with 0.3 mM δ-aminolevulinic acid (ALA), and shaken at 27 °C (180 rpm) for 20–24 h. Cell cultures were harvested by centrifugation at 4000 rpm for 30 min. The cell pellets were resuspended in Ni-NTA Lysis Buffer (50 mM KPi, 250 mM NaCl, 10 mM imidazole, pH 8.0) and flash frozen. After thawing, cells were lysed by sonication, and the cell lysate was clarified by centrifugation (14000 rpm, 4 °C, 45 min). The lysate was transferred to a Ni-NTA column equilibrated with Ni-NTA Lysis Buffer, and the resin was washed with 50 mL of Ni-NTA Wash Buffer (50 mM KPi, 250 mM NaCl, 20 mM imidazole, pH 8.0). Proteins were eluted with Ni-NTA Elution (50 mM KPi, 250 mM NaCl, 250 mM histidine, pH 7.0). After elution from the Ni-NTA column, the protein was buffer-exchanged against KPi buffer (50 mM, pH 7.0) using a 3 kDa molecular weight cutoff Centricon filter. The concentrations of the *HtCc552* variants were determined using the following extinction coefficients: ε_410_ = 85280 M^–1^ cm^–1^ (oxidized) and ε_416_ = 121880 M^–1^ cm^–1^ (reduced).^61^

### Biocatalytic Reactions and Product Analysis

Analytical-scale biocatalytic reactions were carried out with 400 μL samples using 60 μM purified *Ht-Cc552* variant, 5 mM aryl amine substrate, 10 mM alkyl 2-diazo-3,3,3-trifluoropropanoate, and 10 mM sodium dithionite. In general, excess reductant was found to be beneficial for activity (Figure S7). In a typical procedure, *Ht-Cc552* in KPi (50 mM, pH 7.0) was placed in a 5 mL glass crimp vial containing a Teflon-coated magnetic micro stir bar. Sodium dithionite (100 mM stock in KPi (50 mM, pH 7.0)) was placed in a separate 5 mL glass crimp vial, and both vials were purged in tandem with Ar(g) using a cannula for 3 min. The sodium dithionite solution was mixed with the protein solution via cannula, and the reaction was initiated by adding the arylamine substrate (5 μL, 400 mM stock in EtOH) and alkyl 2-diazo-3,3,3-trifluoropropanoate (10 μL, 400 mM stock in EtOH). The biocatalytic reaction mixture was stirred at 60 rpm for 16 h at room temperature under Ar(g) pressure. After 16 h, the reaction was quenched for product analysis by the addition of an internal standard (20 μL of 1,3-benzodioxole at 100 mM in EtOH), followed by extraction with dichloromethane (400 μL) in a 1.5 mL microcentrifuge tube and centrifugation at 14000 rpm for 5 min. The organic layers were collected and subjected to SFC analysis to calculate percent conversion, TON, and enantiomeric ratios.

### Preparative-Scale Synthesis of **6c**

The preparative-scale biocatalytic reaction of **6c** was carried out with a 20 mL sample using 1.0 mol % of purified *Ht-Cc552* variant, 1 equiv of aryl amine substrate, and 2 equiv of alkyl 2-diazo-3,3,3-trifluoropropanoate. Purified *Ht-Cc552*(G50T,M59G,P60E,Q62R) (7.9 mL, 2.1 μmol) in KPi (50 mM, pH 7.0) was placed in a 50 mL round-bottom flask containing a Teflon-coated magnetic stir bar. In a separate 25 mL round-bottom flask containing a Teflon-coated magnetic stir bar were placed sodium dithionite (0.070 g, 0.40 mmol) and KPi (50 mM, pH 7.0), and both round-bottom flasks were purged in tandem for 3 min with Ar(g) using a cannula. The sodium dithionite solution was mixed with the *HtCc552* variant via cannula, and the reaction was initiated by adding 4-bromoaniline (0.035 g, 0.21 mmol, dissolved in 500 μL of DMF) and benzyl 2-diazo-3,3,3-trifluoropropanoate (0.100 g, 0.410 mmol, dissolved in 500 μL of DMF). The biocatalytic reaction mixture was stirred at 60 rpm for 16 h at room temperature under Ar(g) pressure. The reaction was quenched by the addition of diethyl ether (15 mL), and the reaction mixtures were extracted by shaking for 3 min and vortex mixing for 1 min with diethyl ether (3×, 15 mL), followed by centrifuging (4000 rpm, 10 min). The organic layers were collected, dried over anhydrous MgSO_4_, and concentrated via rotary evaporation. The crude product was purified via flash column chromatography using silica gel and 5% EtOAc in hexanes, and the solvent was removed via rotary evaporation to give purified product **6c** (75% yield). After characterization via NMR (^1^H, ^13^C, and ^19^F), the product was subsequently recrystallized using a vapor diffusion protocol. The product **6c** was dissolved using ∼1 mL of dichloromethane in a 1 dram glass vial and was placed in a 20 mL scintillation vial containing ∼4–5 mL of hexane. The vials were placed in a −30 °C freezer, and after 24 h crystalline needles were obtained and analyzed via X-ray crystallography (see the Supporting Information).

### Reduction Potential Determination

These experiments were carried out using a slightly modified version of the UV–vis spectrochemical method reported by Raven and co-workers.^45^ Reactions were carried out on a 1 mL scale in a solution of KPi (50 mM, pH 7) containing xanthine (30 mM stock solution), protein, dye (Bindschedler’s green), catalase (10 mg/mL stock solution), and xanthine oxidase (175 μM stock solution). In a sealed vial, a solution of a buffer containing xanthine (300 μM final concentration) was degassed by bubbling argon for 3 min. A buffered solution containing the *Ht-Cc552* variants and dye was carefully degassed in a similar manner in a sealed cuvette (the concentration of the dye was adjusted by titration to give an absorbance which is approximately equal to that of the highest absorbance band in the protein spectra). The two solutions were then mixed together via cannula, and then catalase (5 μg/mL final concentration) and xanthine oxidase (50 nM final concentration) were added to initiate the two-electron oxidation of xanthine to uric acid and the corresponding reduction of the protein and dye. The reactions were monitored by UV–vis spectrophotometry, and the data were plotted. The reduction potential was determined by adding the standard reduction potential of the dye to the value of the *y* intercept obtained by fitting the data to the Nernst equation (eq 1):
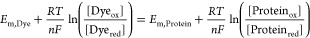
1The absorbance values corresponding to the protein (based on the Soret band of the oxidized form) and the dye (Figure S8) were used to determine the ratio of concentrations of the oxidized (ox) to the reduced (red) form of both the protein and dye at each stage of the experiment (eq 2):
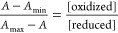
2

### DFT Calculations

The aniline substrate can approach the carbenoid from the *Re* or *Si* face of the sp^*2*^ carbene plane, chiralizing the prochiral carbenoid carbon and thus leading to the formation of an enantiomeric pair of products. Additionally, the ester group can adopt two possible conformations both parallel to the heme plane marked as + and −, respectively (χ2, Figure 3A). We performed geometry optimizations and frequency calculations for the two *R* enantioselective conformations *R*(+) and *R*(−) and the two *S* enantioselective conformations *S*(+) and *S*(−) of a truncated imidazole porphyrin Fe ylide complex. DFT calculations were conducted using the Gaussian16 software package.^62^ Starting from a broken-symmetry initial guess of unrestricted open-shell wave functions,^49^ we performed geometry optimization, frequency, and single-point energy calculations at the UωB97XD/SDD/6-311G**//UωB97XD/SDD/def2-TZVP level, among which the effective core potential (ECP) SDD was used to describe the iron atom and 6-311G(d)/def2-TZVP for other atoms. The polarizable continuum model (PCM) (diethyl ether)^63^ was used to mimic the dielectric permittivity in the enzyme active site. Coordinates of the optimized intermediate structures corresponding to all four conformations are provided in Figure S7 and the list of atom coordinates in the Supporting Information.

### Rosetta Modeling of *HtCc552*-ylide Complexes

In addition to the ± orientation of the ester moiety, a clockwise rotation of the Fe–C bond dihedral can produce four Fe ylide conformations relative to the heme cofactor and the iron-coordinating histidine, represented by rot1 (the benzyl ester group positioned between porphyrin rings A and B of the heme *c*) to rot4 (between rings D and A) here (χ1, Figure 3A; porphyrin ring names can be found in Figure 4). To take into account these degrees of freedom, the Rosetta FastRelax mover was applied to the *HtCc552* crystal structure aligned with DFT-generated Fe ylide models to make amino acid substitutions and refine the protein structures. The Rosetta score function used in modeling was REF2015_cst^64^ with the two additional score terms “fa_intra_atr_nonprotein” and “fa_intra_rep_nonprotein” to strengthen the intramolecular Lennard–Jones interactions in the Fe ylide intermediate. Coordinate constraints were applied to the protein backbone atoms to prevent large deviations of atomic positions from the crystal structure. Constraints and other Rosetta input files can be found in the Supporting Information. We ran 50 simulation trajectories for each complex, and the values reported in Table 2 are the minimum energy values with the coordinate constraint score subtracted from the total energy score over all 50 trajectories.

## Supporting Information Available

Supporting Information includes supplementary The Supporting Information is available free of charge at https://pubs.acs.org/doi/10.1021/jacs.1c10750.

Additional tables and figures as described in the text, chiral GC and SFC chromatograms, synthetic procedures, compound characterization data, NMR spectra, and atom coordinates of the DFT models (PDF)

## Accession Codes

CCDC 2113422 contains the supplementary crystallographic data for this paper. These data can be obtained free of charge via www.ccdc.cam.ac.uk/data_request/cif, or by emailing data_request@ccdc.cam.ac.uk, or by contacting The Cambridge Crystallographic Data Centre, 12 Union Road, Cambridge CB2 1EZ, UK; fax: +44 1223 336033.
