# Supplementary material for: Enantioselective Synthesis of α-Trifluoromethyl Amines via Biocatalytic N–H Bond Insertion with Acceptor-Acceptor Carbene Donors
Source: J Am Chem Soc. 2022 Feb 2;144(6):2590–602. doi: 10.1021/jacs.1c10750 (PMC8855427; doi:10.1021/jacs.1c10750)
Supplement: Supplementary file 1 — ja1c10750_si_001.pdf [file ja1c10750_si_001.pdf]

Supporting information for

Enantioselective Synthesis of α-Trifluoromethyl Amines via Biocatalytic N−H Bond Insertion with Acceptor-Acceptor Carbene Donors

Donggeon Nam[a,ǂ], Antonio Tinoco[a,c,ǂ], Zhuofan Shen[b], Ronald D. Adukure[a], Gopeekrishnan Sreenilayam, Sagar D. Khare[b,*], Rudi Fasan[a,*]

[a] *Department of Chemistry, University of Rochester, Rochester, NY 14627, United States*

[b] *Department of Chemistry and Chemical Biology, Rutgers University, New Brunswick, NJ 08854, United States*

[c] *Current address: Department of Chemistry and Chemical Biology, Harvard University, Cambridge, MA 02138, United States*

ǂ These authors contributed equally to this work.

* Corresponding authors. Email: rfasan@ur.rochester.edu; khare@chem.rutgers.edu.

Table of contents:

Supplementary Figures S1-S15	Pages S2-S36
Supplementary Tables S1-S3	Pages S37-S39
X-Ray Crystallographic Analyses	Pages S40-S42
Reagents and Analytical Methods	Pages S43-S46
Synthetic Procedures	Pages S47-S72
NMR spectra	Pages S73-S144
Computational Data	Pages S145-156
References	Page S157

Figures S1-S4. Activities and enantioselectivities of *Ht-Cc552c* variants toward stereoselective N−H carbene insertion with *p*-anisidine (**1a**) and EtDTP (**2a**). In each graphs, only the mutated positions over the parent enzyme are reported. Reaction conditions: 5 mM *p*-anisidine (**1**), 10 mM EtDTP (**2a**), 60 µM purified *Ht-Cc552* variant in KPi buffer (50 mM, pH 7), room temperature, 16 hours under Ar (g) pressure. * = 20 µM purified protein, # = 10 µM purified protein.

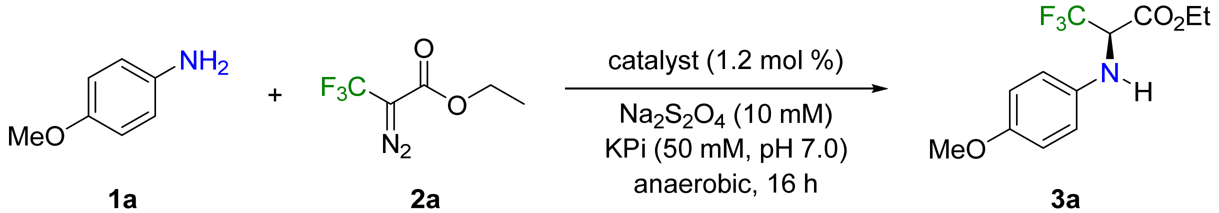

1a **2a** **3a**

Figure S1. *Ht-Cc552*(M59G,**<u>Q62X</u>**) SSM Library

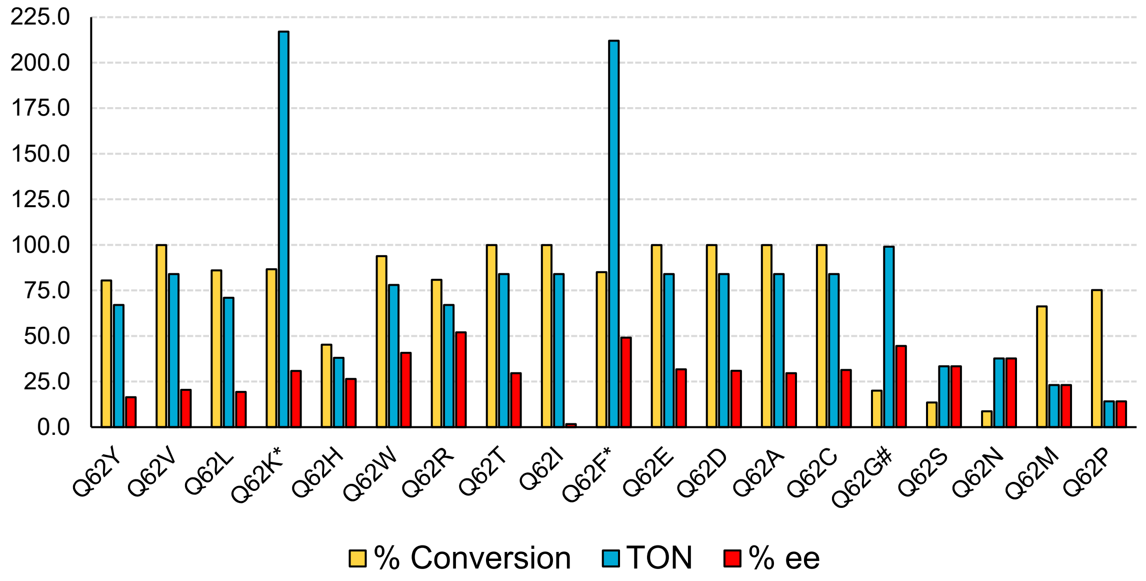

Figure S2. *Ht-Cc552(*M59G,Q62R,**<u>P60X</u>**) SSM Library

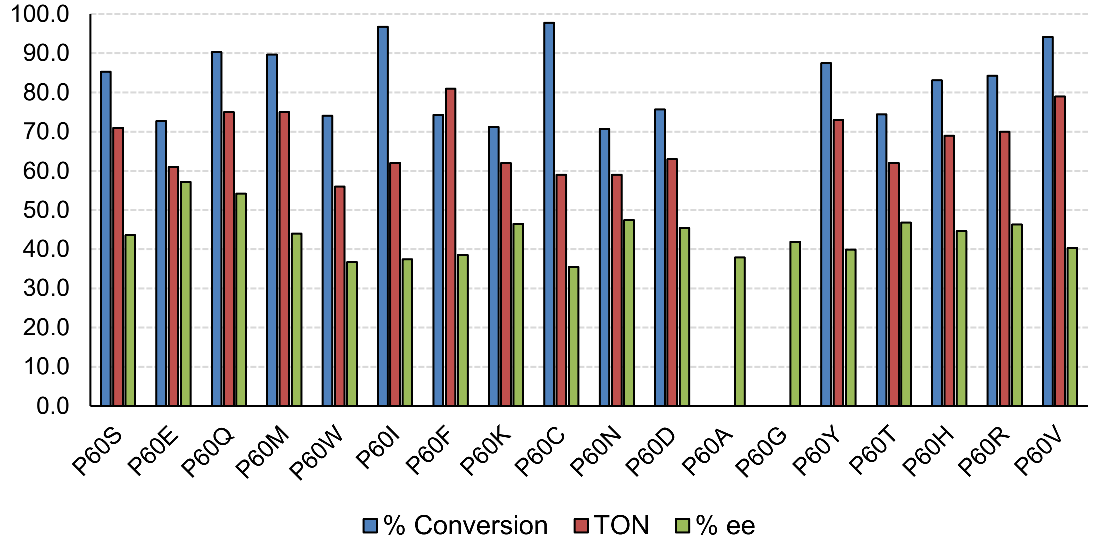

Figure S3. *Ht-Cc552(*M59G,Q62R,**<u>P61X</u>**) SSM Library

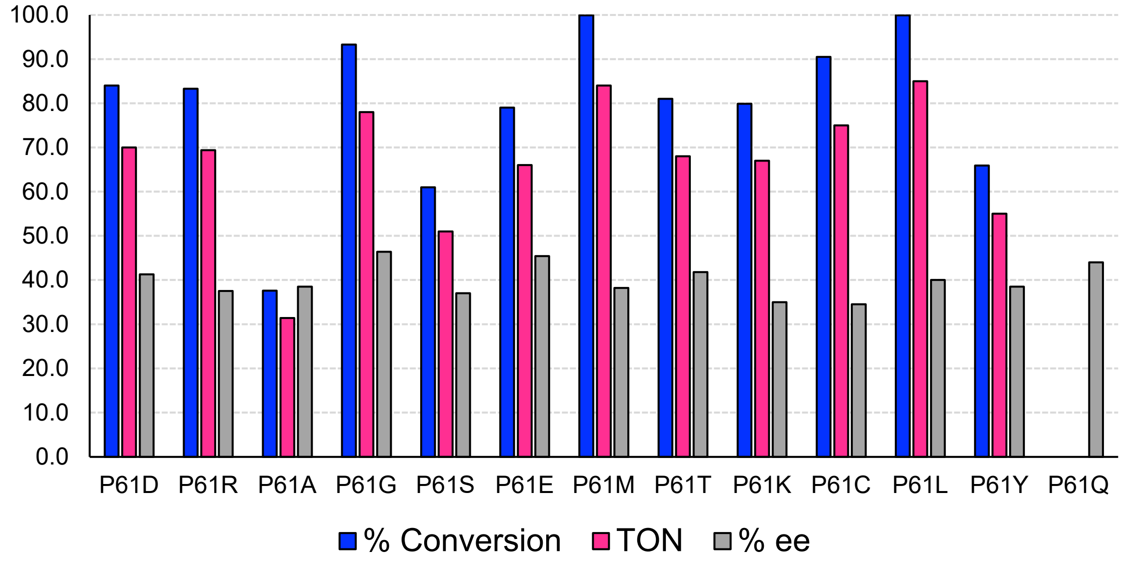

Figure S4. *Ht-Cc552*(**G50X**,M59G,P60E,Q62R) SSM Library

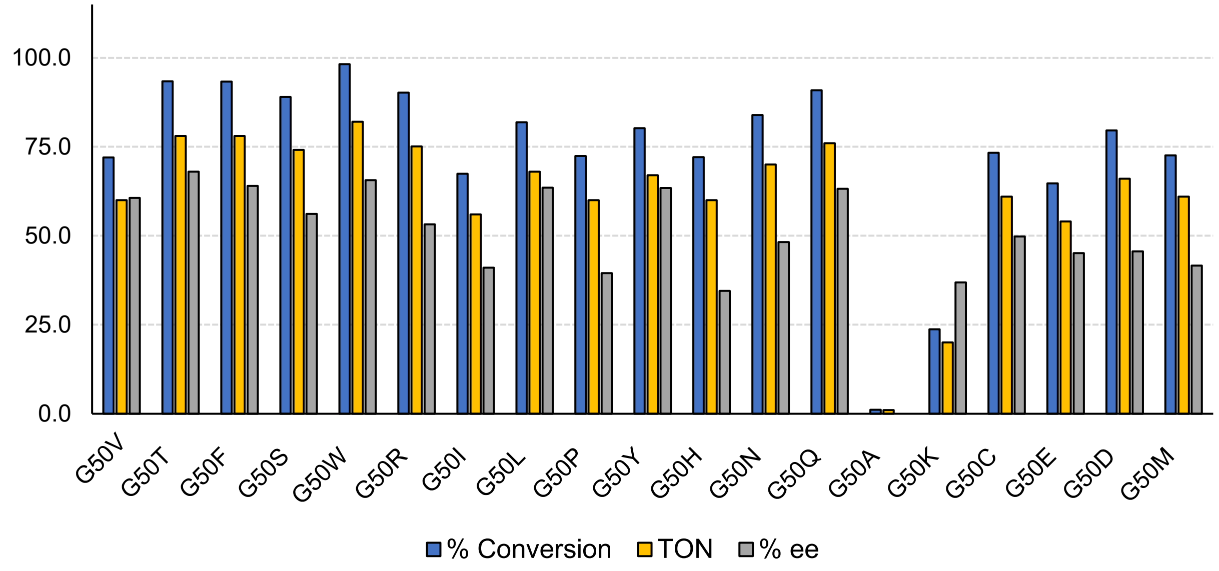

.

Figure S5. Time-course analysis of *Ht-Cc552*(G50T,M59G,P60E,Q62R)-catalyzed N−H carbene insertion of *p*-anisidine (**1a**) with EtTDP (**2a**) or BnTDP (**2c**). Reaction conditions: 5 mM *p*-anisidine (**1a**), 10 mM EtDTP (**2a**) or BnTDP (**2c**), 60 μM (1.2 mol %) catalyst in KPi (50 mM, pH 7.0), 10 mM $Na_2S_2O_4$, rt, under Ar (g) atmosphere. Reactions were quenched with 1 N HCl at the indicated time points, and the yield (%) was determined by gas chromatography using a calibration curve prepared from racemic standard **1b**. Experiments were performed in duplicates.

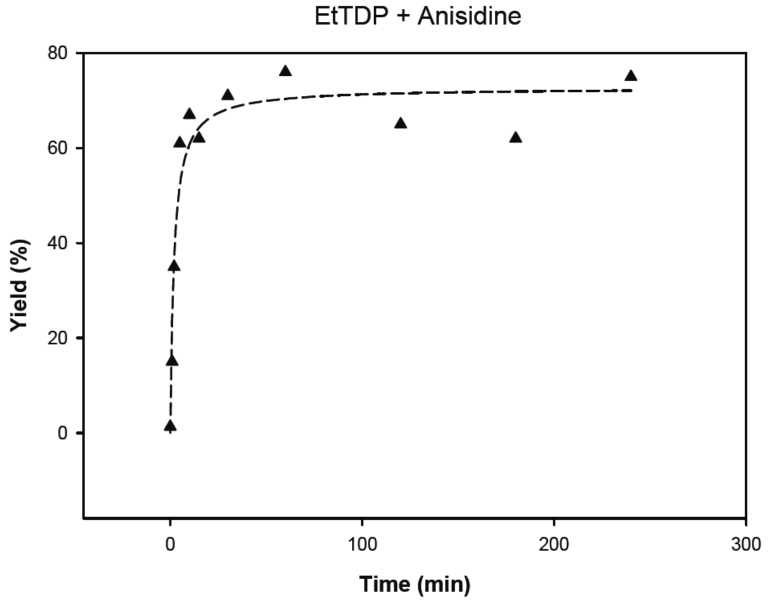

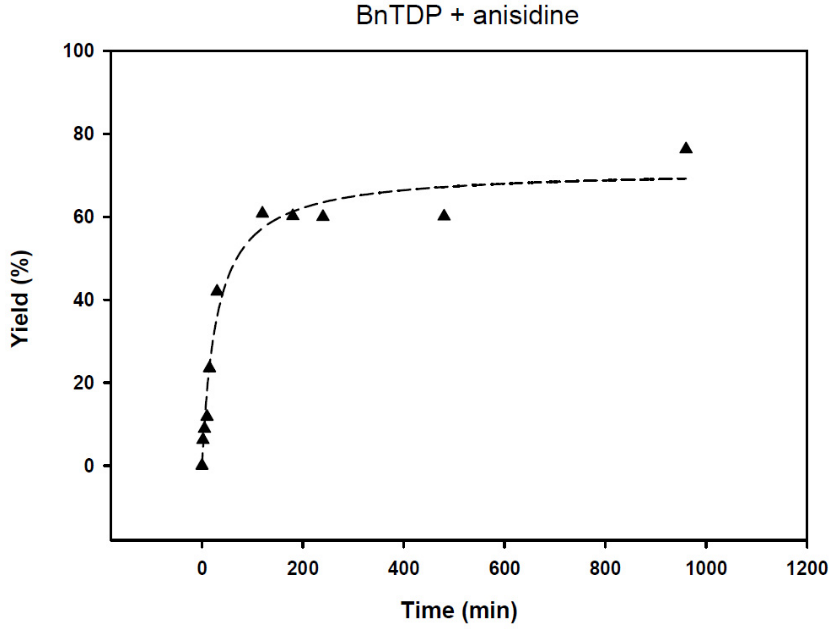

Figure S6. UV-vis analysis of *Ht-Cc552*(G50T,M59G,P60E,Q62R)-catalyzed N−H carbene insertion of *p*-anisidine (**1a**) with BnTDP (**2c**). Reaction conditions: 5 mM *p*-anisidine (**1a**), 10 mM BnDTP (**2c**), 10 μM catalyst in KPi (50 mM, pH 7.0), 10 mM $Na_2S_2O_4$, rt, under Ar (g) atmosphere.

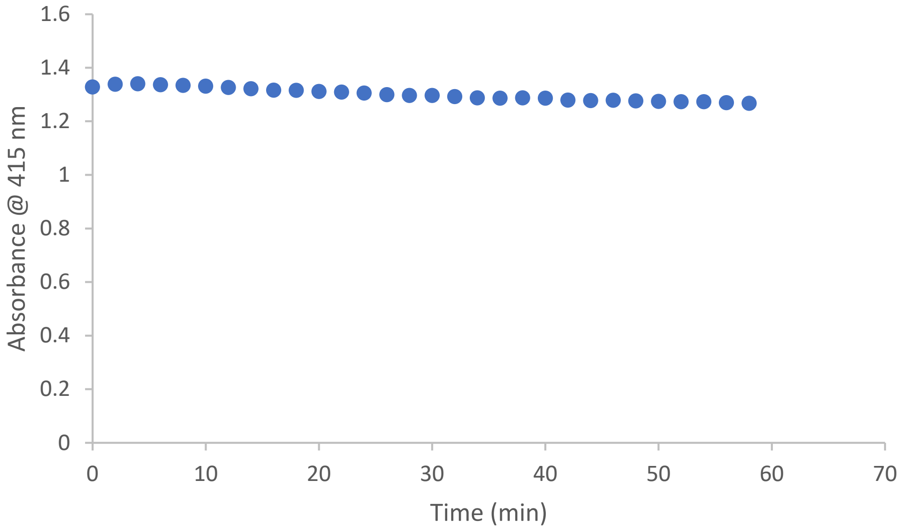

Figure S7. Effect of reductant concentration ([$Na_2S_2O_4$]) on the assay yield of the *Ht-Cc552*(G50T,M59G,P60E,Q62R)-catalyzed N–H carbene insertion reaction with *p*-anisidine (**1a**) and BnTDP (**2c**). Reaction conditions: 5 mM *p*-anisidine (**1a**), 10 mM BnDTP (**2c**), 60 µM catalyst in KPi (50 mM, pH 7.0), 0.1-10 mM $Na_2S_2O_4$, rt, under Ar (g) atmosphere.

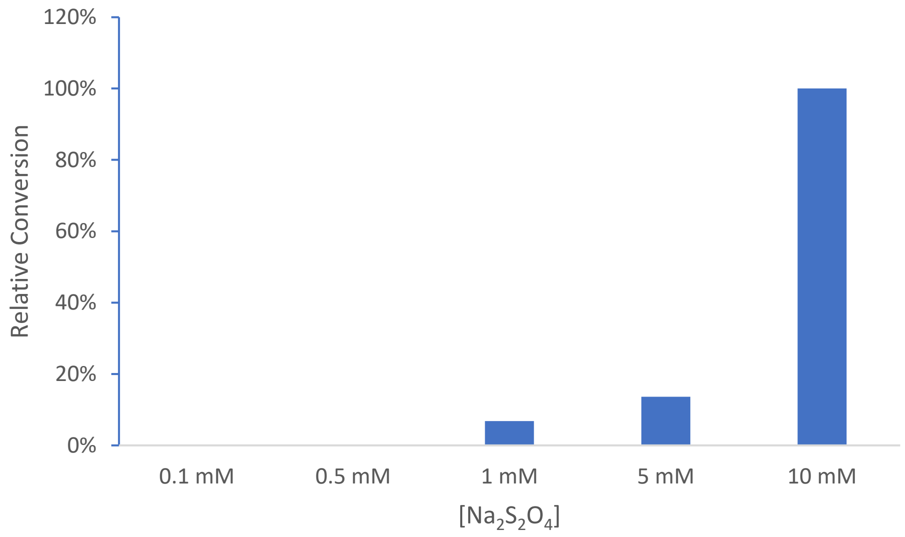

Figure S8. Redox potential determination for wild-type *Ht-Cc552* and the *Ht-Cc552*(M59G) variant. UV-vis spectra during the determination of $Fe^{3+/2+}$ reduction and Nernst plot.

A) Wild-type *Ht-Cc552* (Dye: Bindschedler's green). UV-vis spectra (top) and Nernst plot generated from UV-vis spectra (bottom).

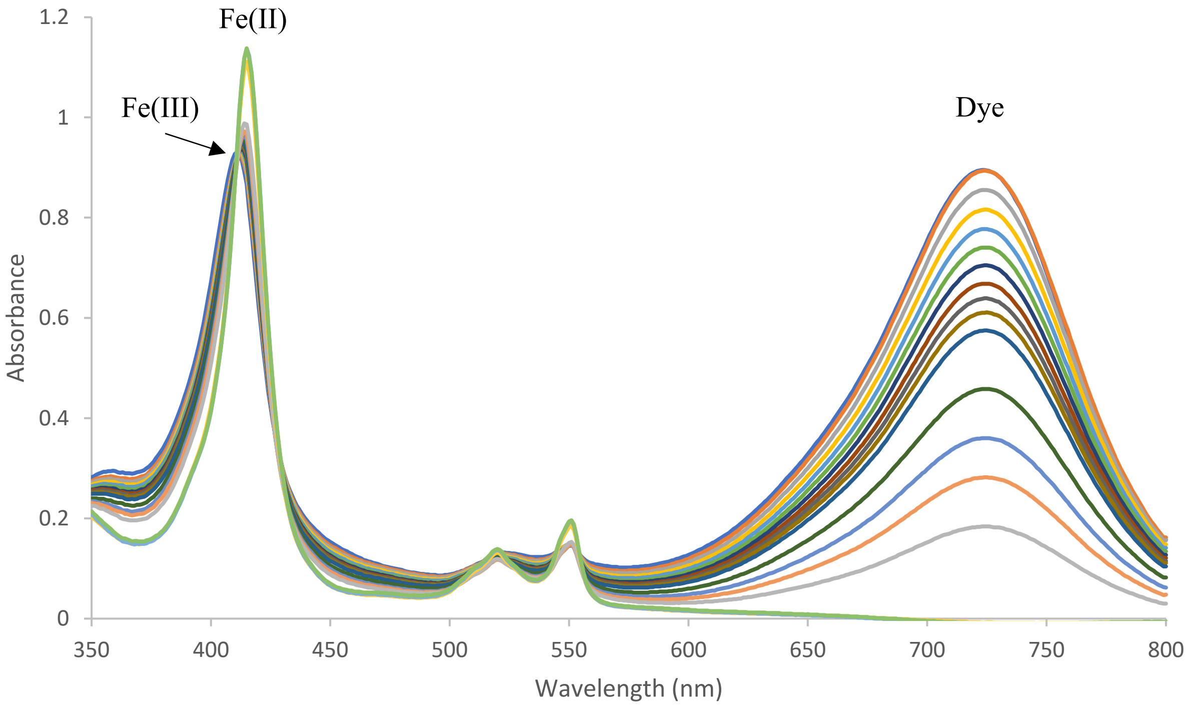

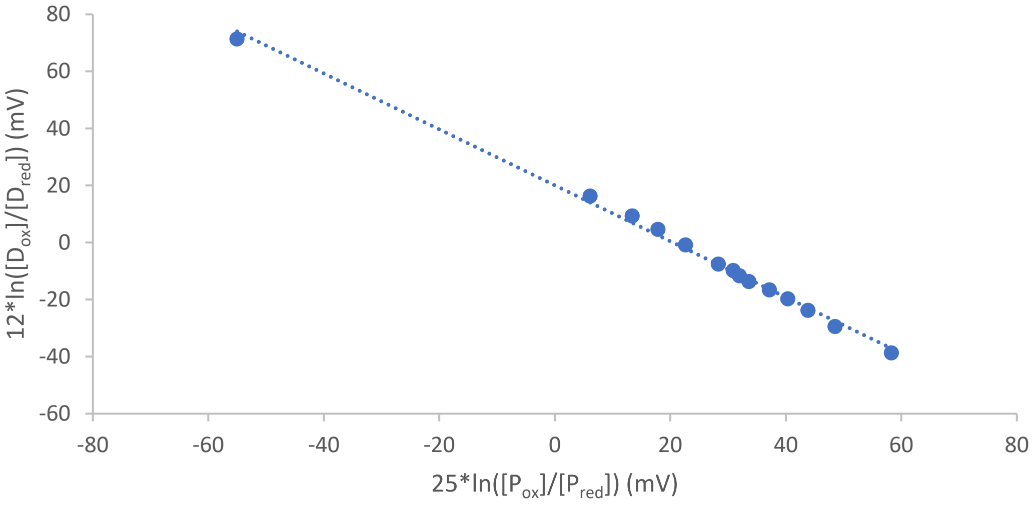

B) *Ht-Cc552*(M59G) variant (Dye: Bindschedler's green). No reduction of the protein was observed while the dye (E_m = +248 mV) was reduced, indicating that the $E^o_{Fe^{3+}/Fe^{2+}}$ of the metalloprotein must be >300 mV.

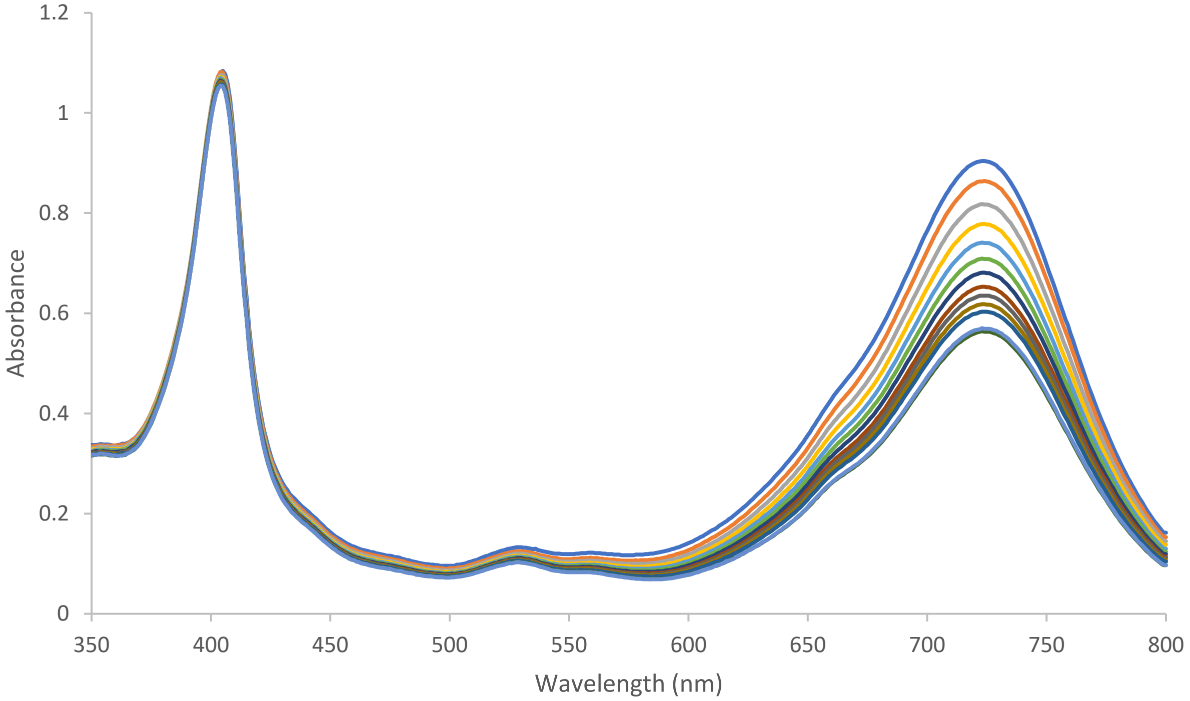

Figure S9. Chiral SFC analyses for the determination of enantiomeric ratios of *Ht-Cc552*-catalyzed N-H carbene insertion reactions. The racemic analytical standards were synthesized as described in the Experimental Procedures.

a) Chiral SFC analysis of enzymatically-produced **3a** (*top*) and racemic **3a** (*bottom*):

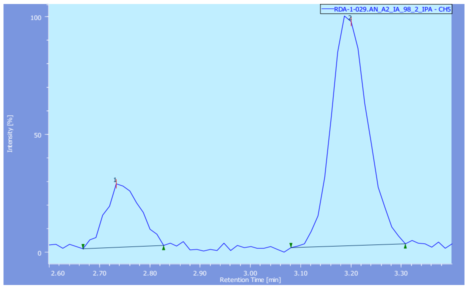

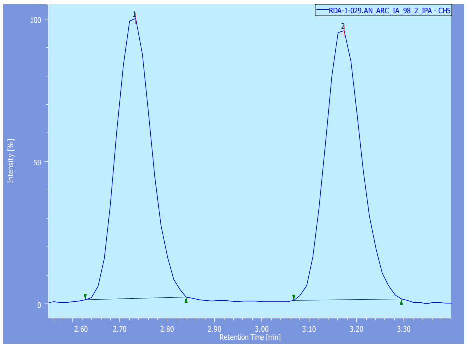

b) Chiral SFC analysis of enzymatically-produced **3b** (*top*) and racemic **3b** (*bottom*):

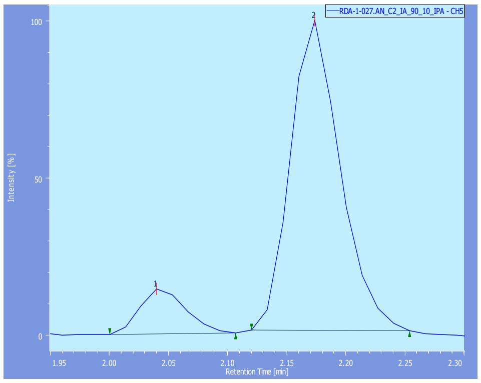

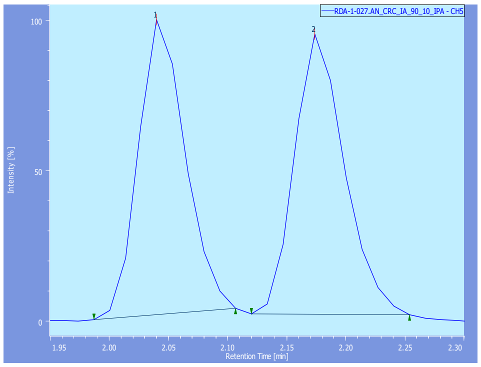

c) Chiral SFC analysis of enzymatically-produced **3c** (*top*) and racemic **3c** (*bottom*):

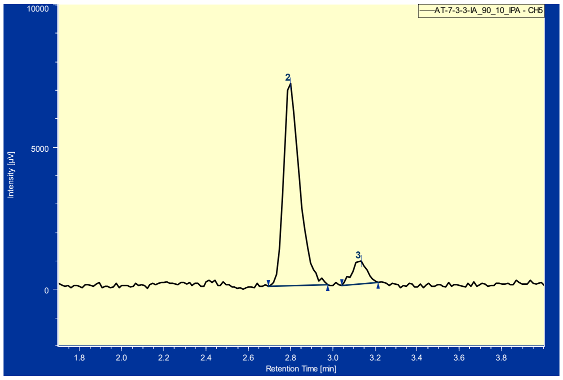

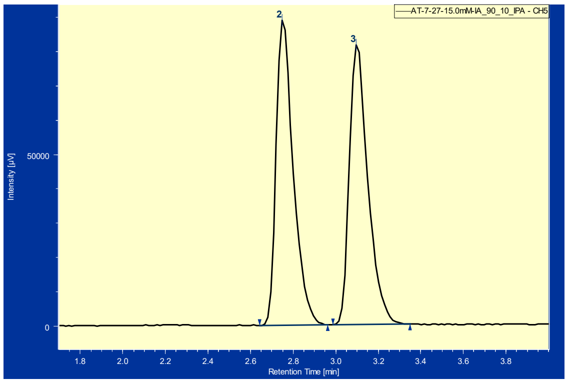

d) Chiral SFC analysis of enzymatically-produced **3d** (*top*) and racemic **3d** (*bottom*):

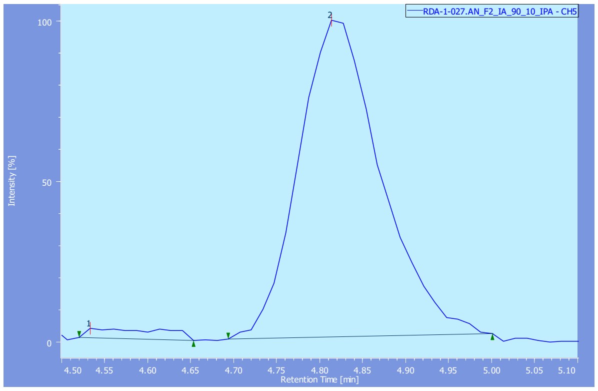

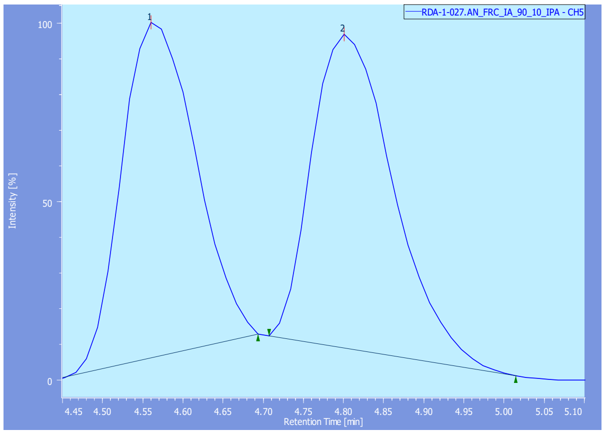

e) Chiral SFC analysis of enzymatically-produced **3e** (*top*) and racemic **3e** (*bottom*):

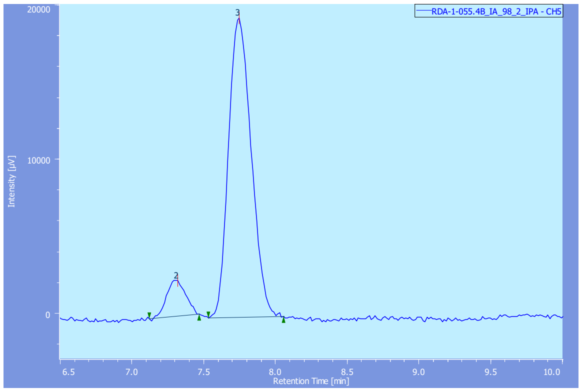

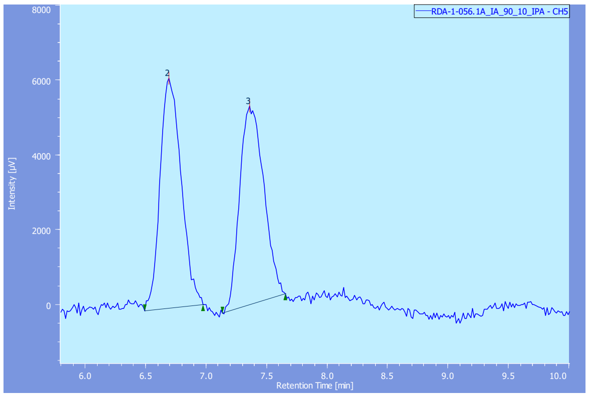

f) Chiral SFC analysis of enzymatically-produced **3f** (*top*) and racemic **3f** (*bottom*):

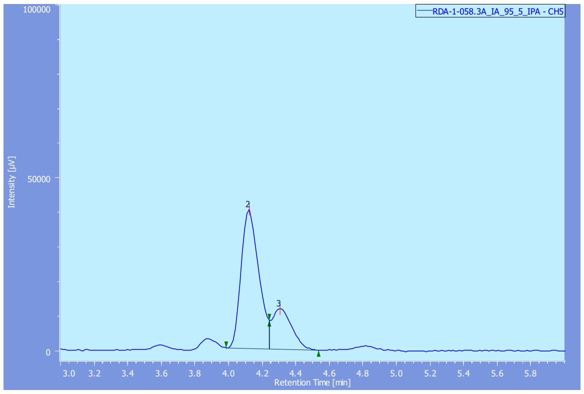

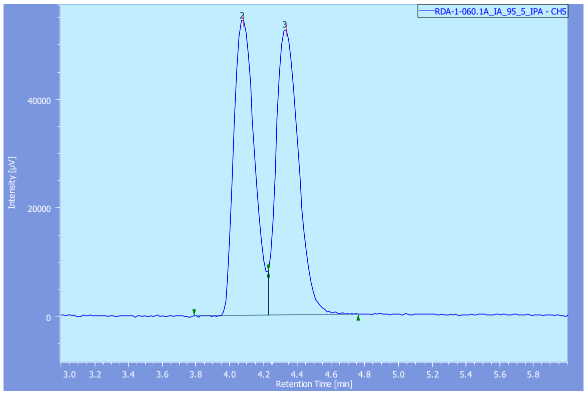

g) Chiral SFC analysis of enzymatically-produced **3g** (*top*) and racemic **3g** (*bottom*):

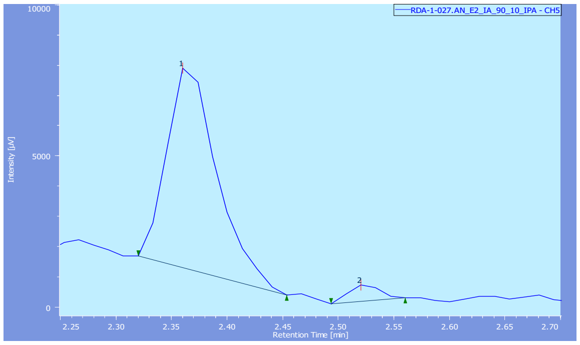

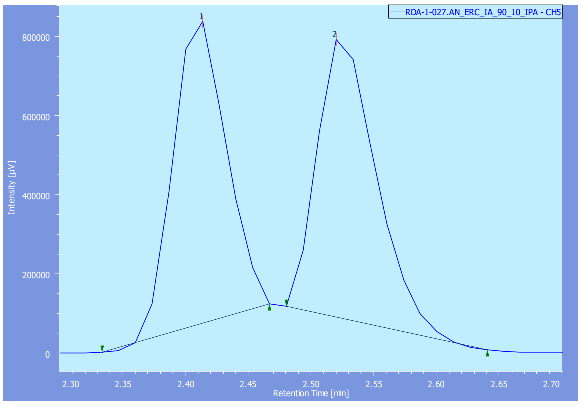

h) Chiral SFC analysis of enzymatically-produced **3h** (*top*) and racemic **3h** (*bottom*):

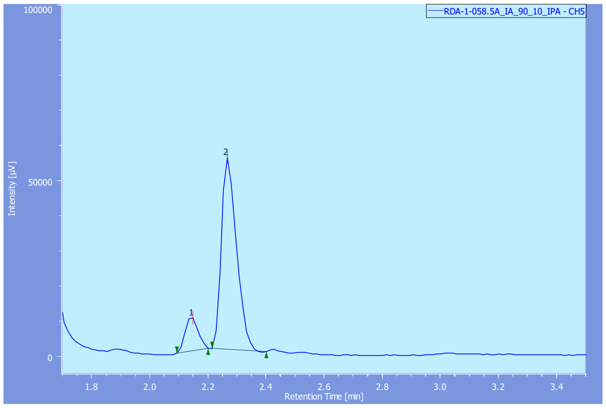

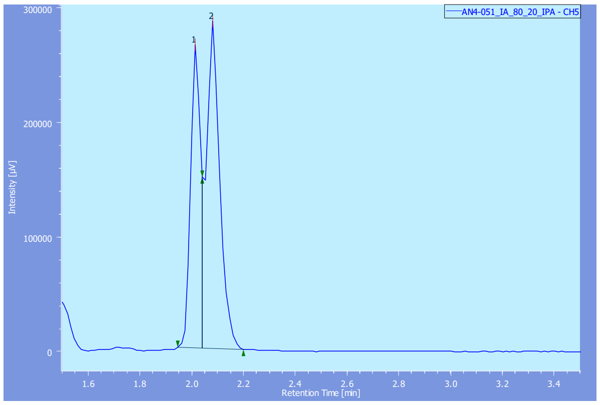

i) Chiral SFC analysis of enzymatically-produced **4b** (*top*) and racemic **4b** (*bottom*):

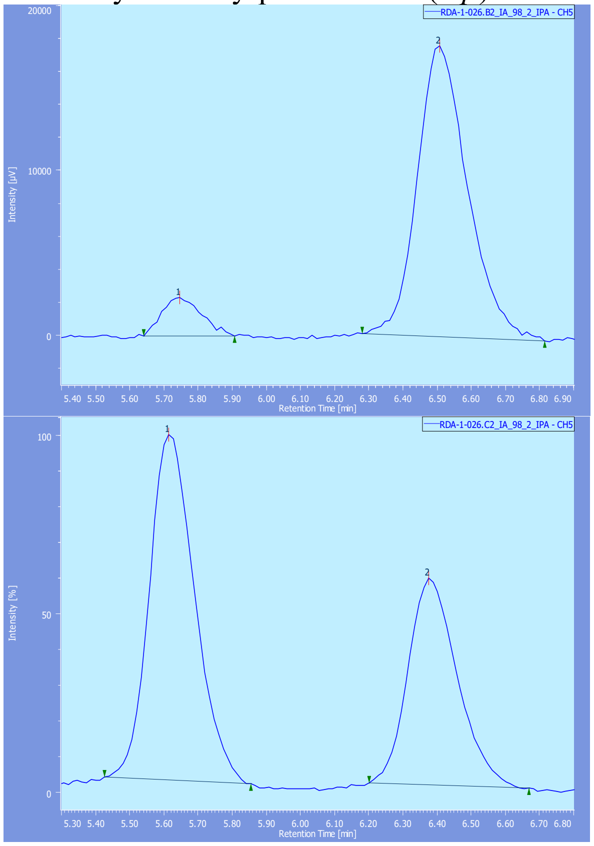

j) Chiral SFC analysis of enzymatically-produced **4c** (*top*) and racemic **4c** (*bottom*):

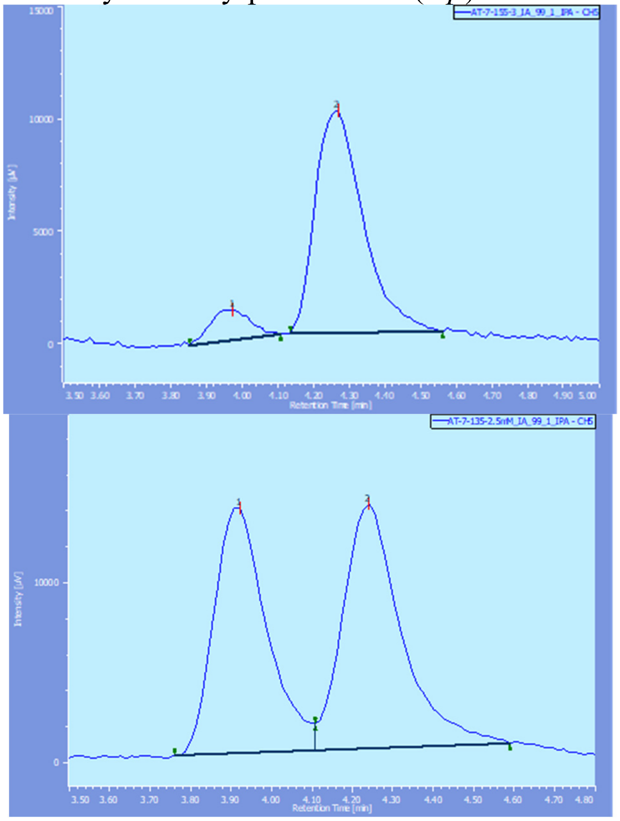

k) Chiral SFC analysis of enzymatically-produced **4d** (*top*) and racemic **4d** (*bottom*):

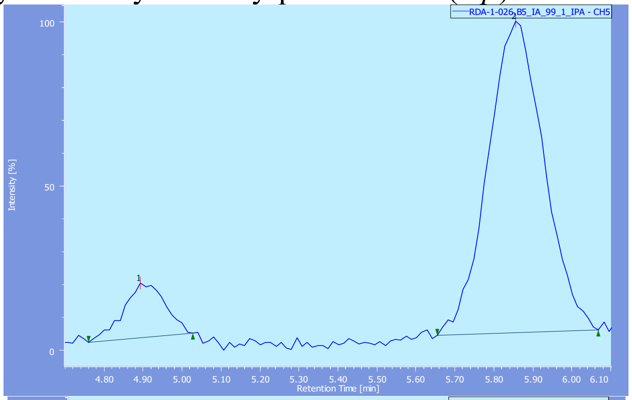

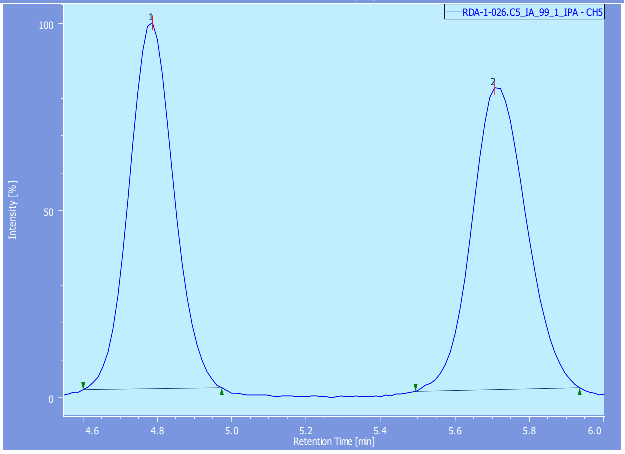

l) Chiral SFC analysis of enzymatically-produced **4e** (*top*) and racemic **4e** (*bottom*):

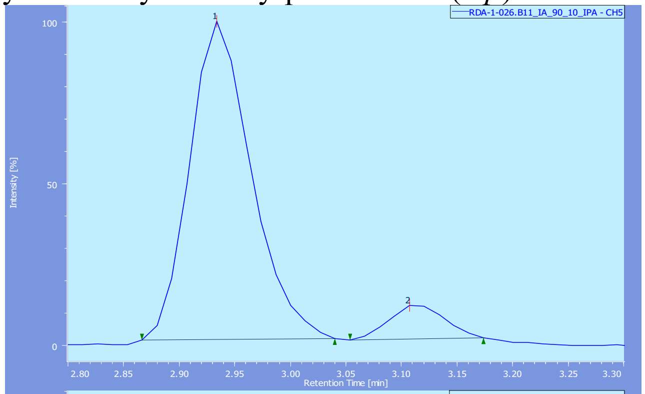

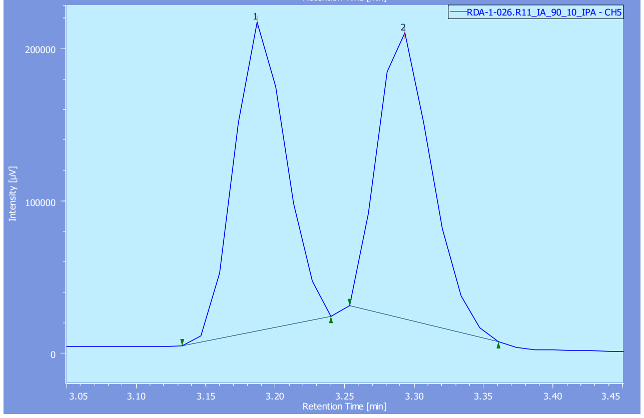

m) Chiral SFC analysis of enzymatically-produced **4f** (*top*) and racemic **4f** (*bottom*):

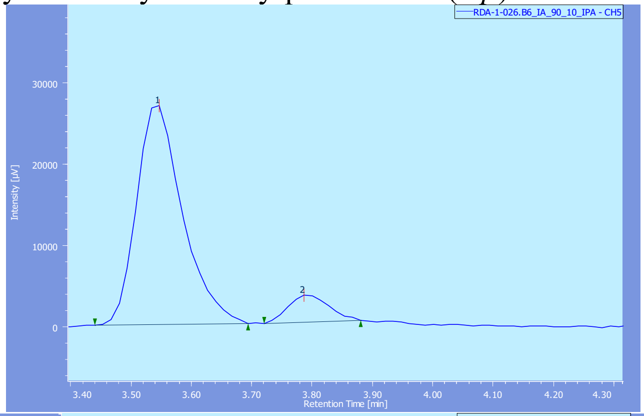

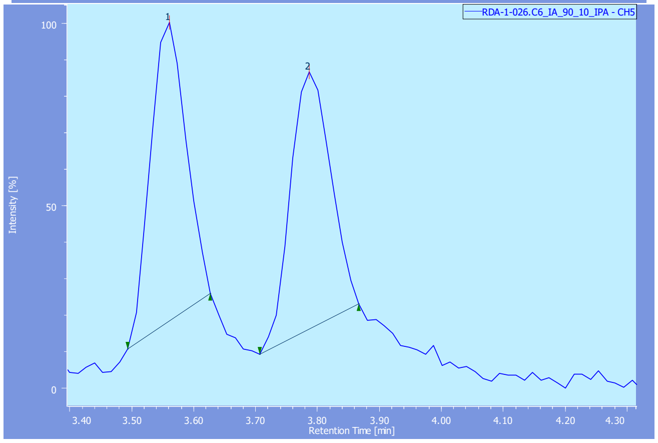

n) Chiral SFC analysis of enzymatically-produced **4g** (*top*) and racemic **4g** (*bottom*):

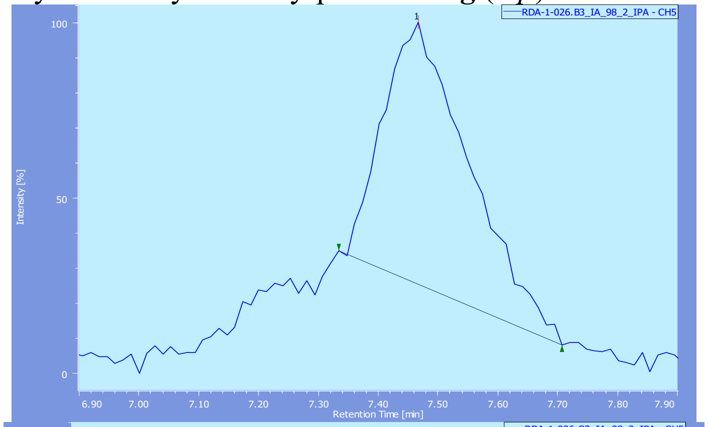

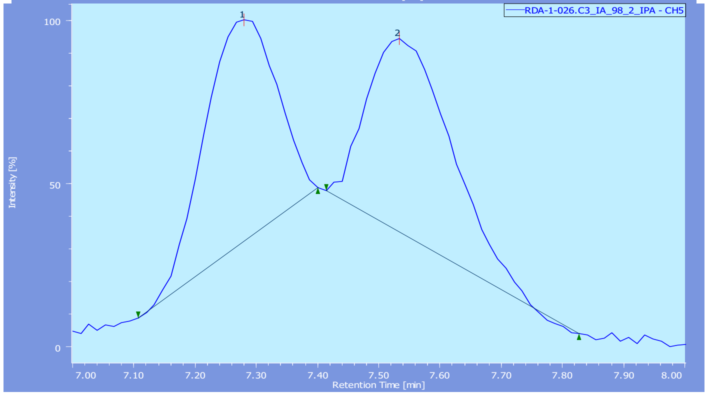

o) Chiral SFC analysis of enzymatically-produced **4h** (*top*) and racemic **4h** (*bottom*):

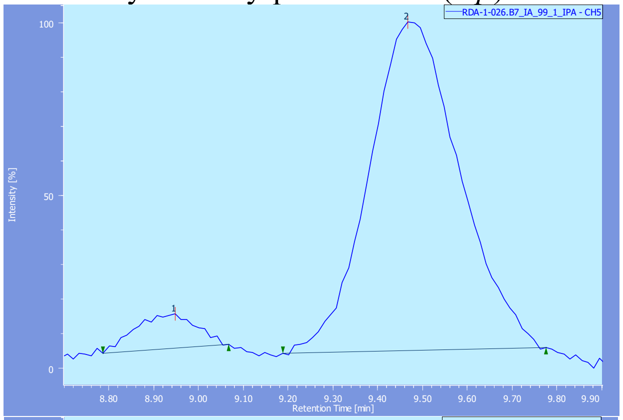

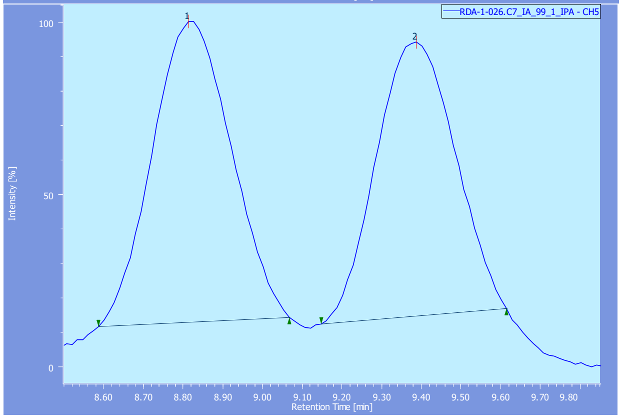

p) Chiral SFC analysis of enzymatically-produced **4i** (*top*) and racemic **4i** (*bottom*):

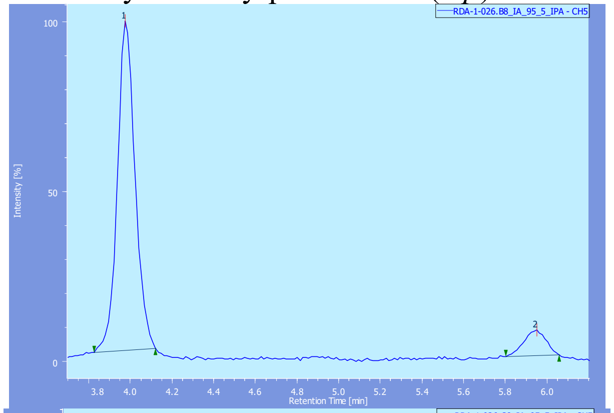

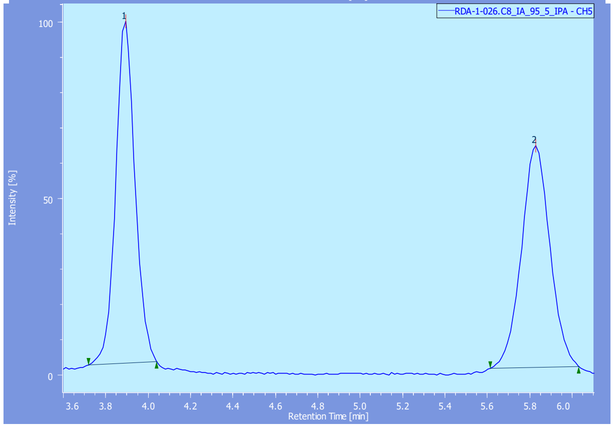

q) Chiral SFC analysis of enzymatically-produced **4j** (*top*) and racemic **4j** (*bottom*):

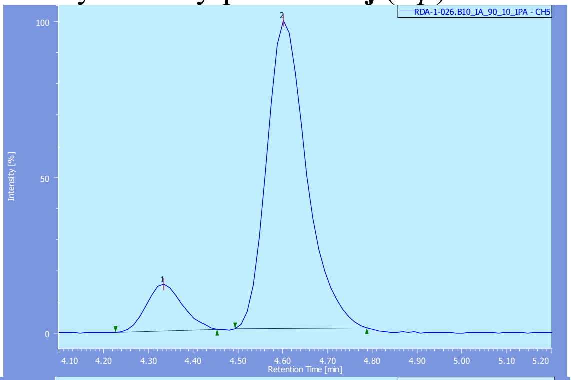

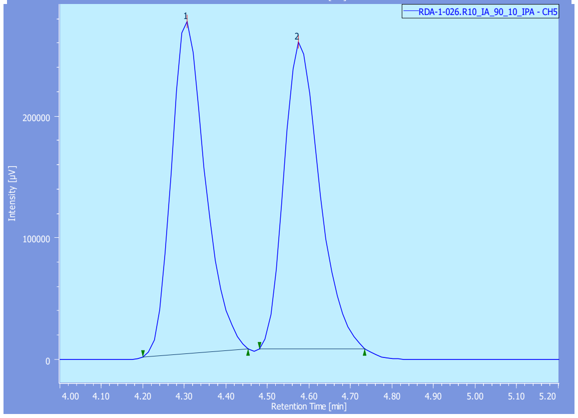

r) Chiral SFC analysis of enzymatically-produced **4k** (*top*) and racemic **4k** (*bottom*):

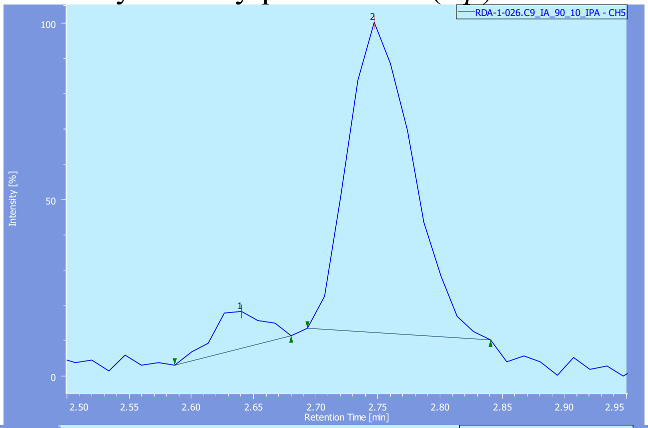

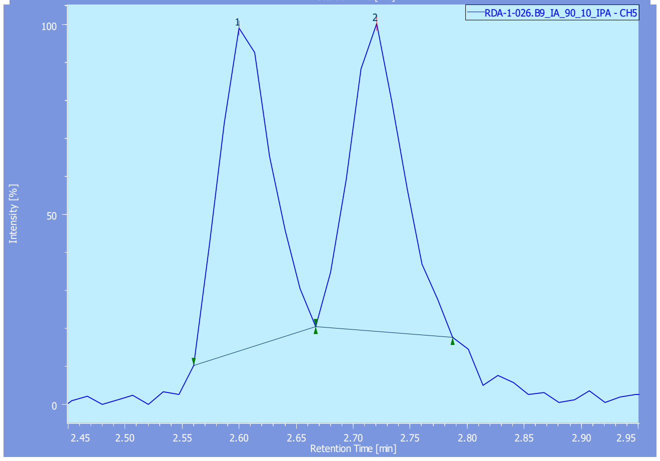

s) Chiral SFC analysis of enzymatically-produced **4l** (*top*) and racemic **4l** (*bottom*):

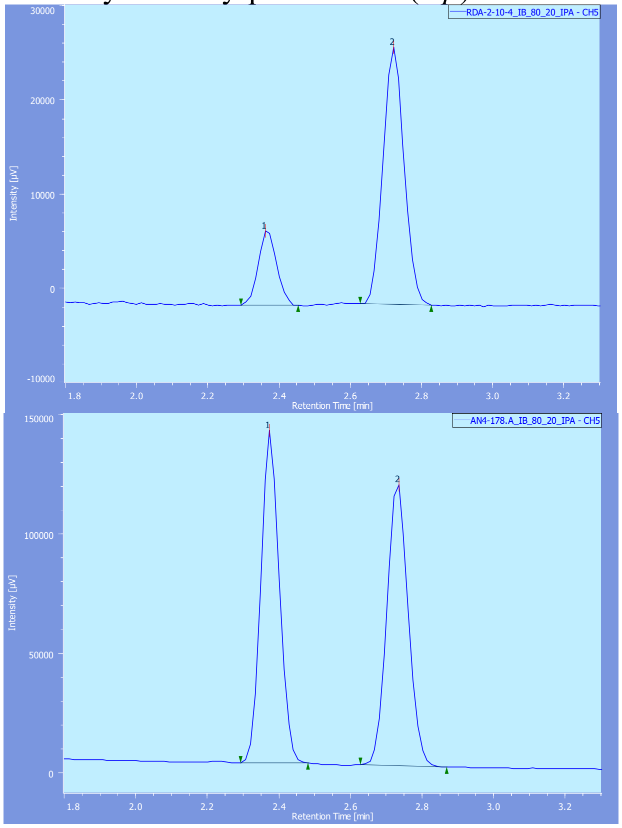

t) Chiral SFC analysis of enzymatically-produced **4m** (*top*) and racemic **4m** (*bottom*):

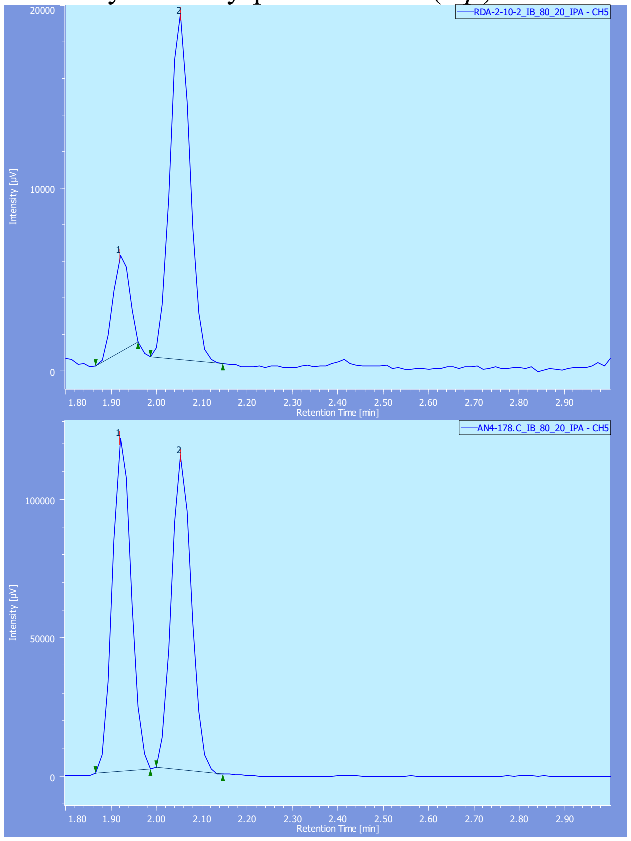

u) Chiral SFC analysis of enzymatically-produced **4n** (*top*) and racemic **4n** (*bottom*):

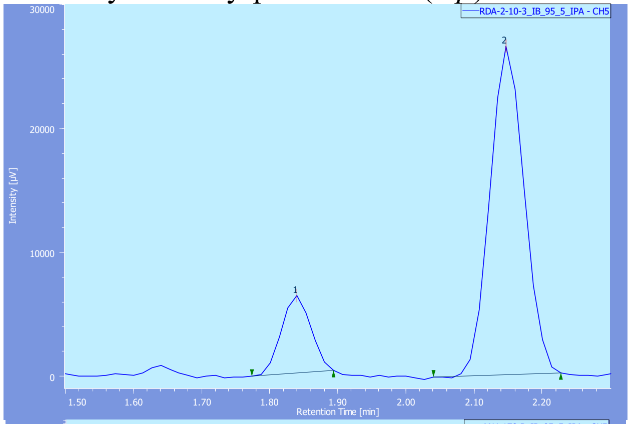

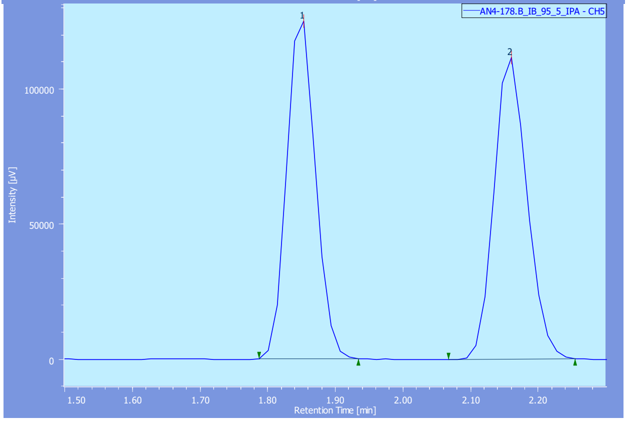

v) Chiral SFC analysis of enzymatically-produced **5a** (*top*) and racemic **5a** (*bottom*):

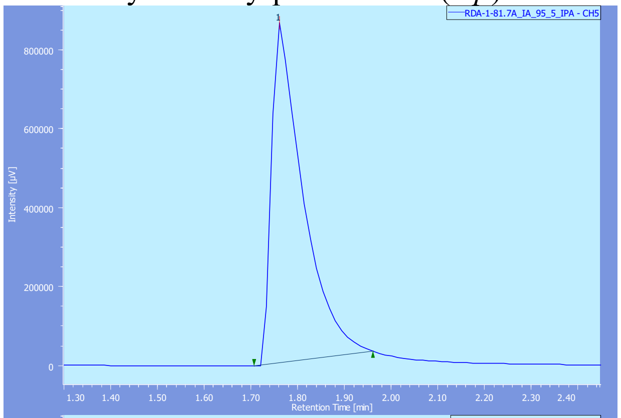

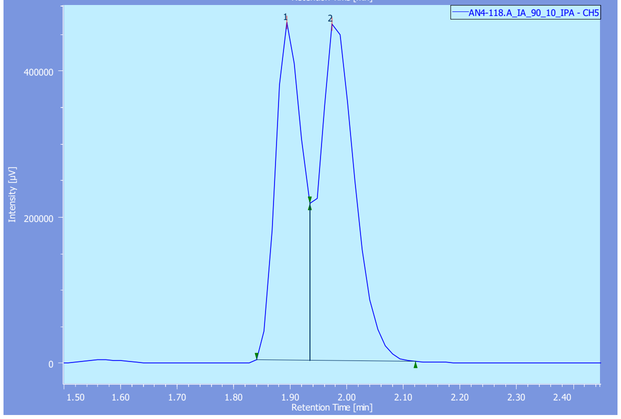

w) Chiral SFC analysis of enzymatically-produced **5c** (*top*) and racemic **5c** (*bottom*):

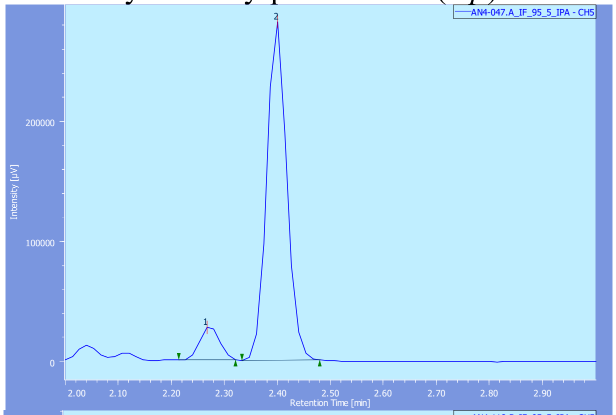

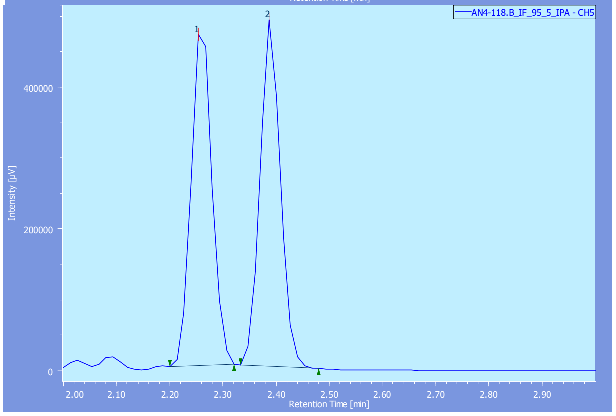

x) Chiral SFC analysis of enzymatically-produced **5d** (*top*) and racemic **5d** (*bottom*):

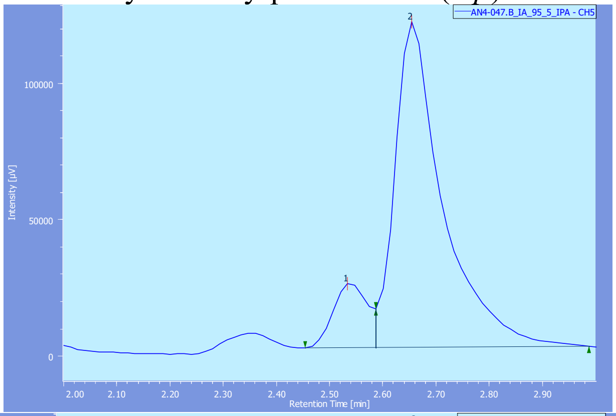

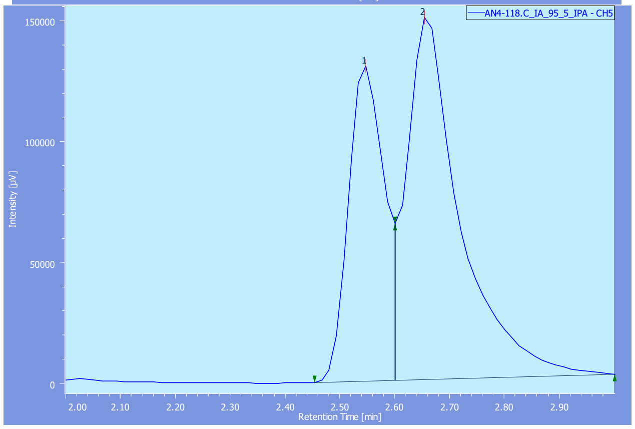

y) Chiral SFC analysis of enzymatically-produced **5e** (*top*) and racemic **5e** (*bottom*):

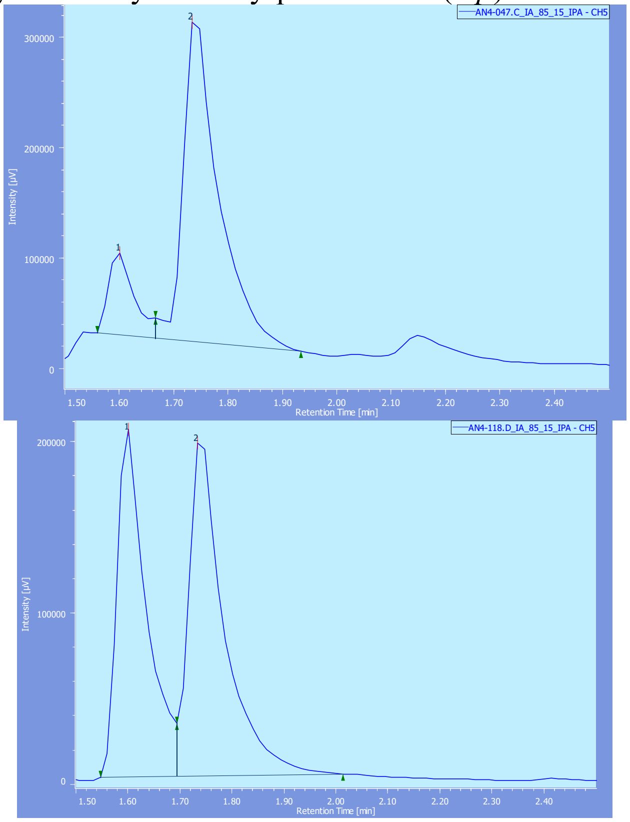

z) Chiral SFC analysis of enzymatically-produced **5f** (*top*) and racemic **5f** (*bottom*):

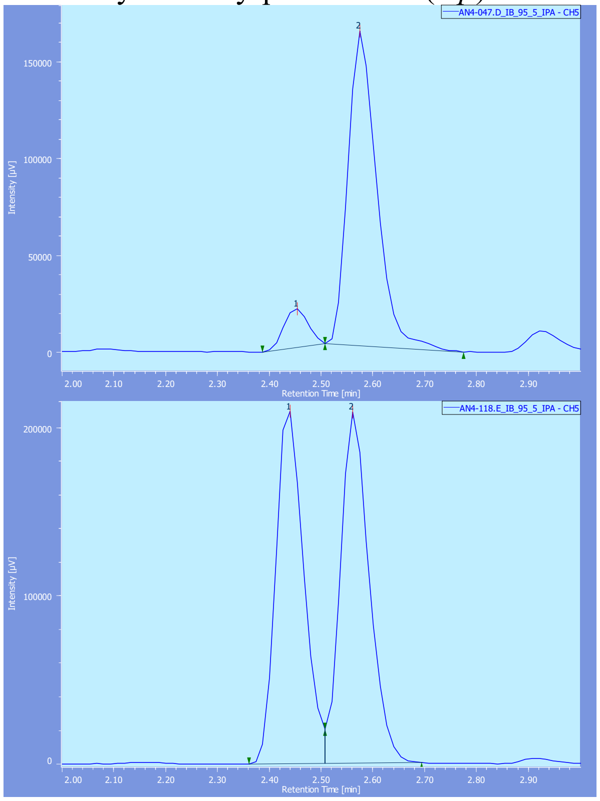

aa) Chiral SFC analysis of enzymatically-produced **5g** (*top*) and racemic **5g** (*bottom*):

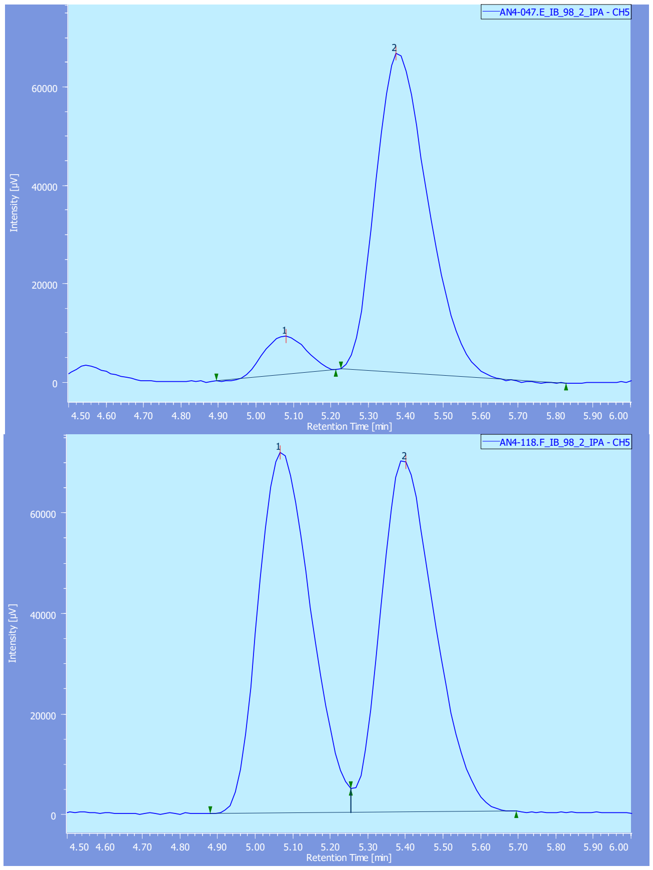

bb) Chiral SFC analysis of enzymatically-produced **5h** (*top*) and racemic **5h** (*bottom*):

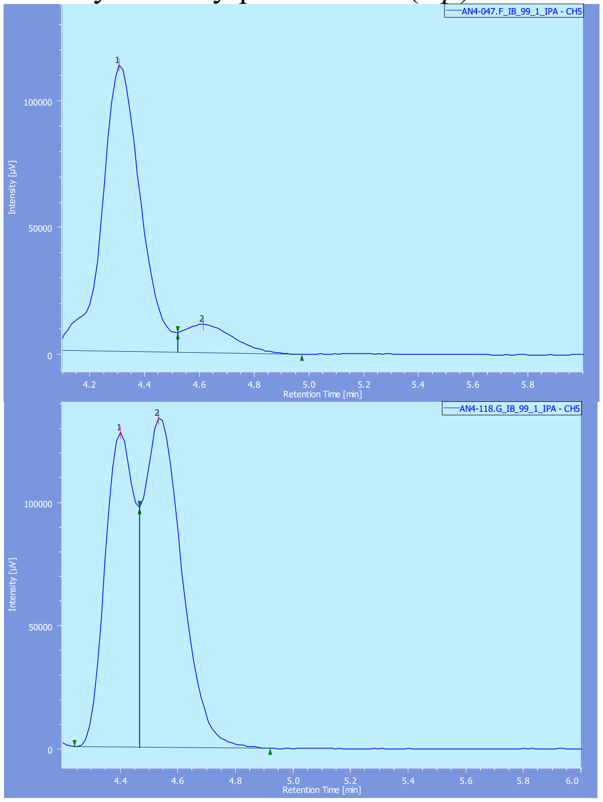

cc) Chiral SFC analysis of enzymatically-produced **5i** (*top*) and racemic **5i** (*bottom*):

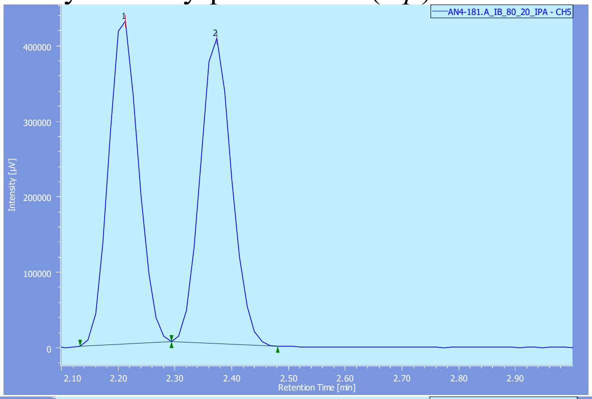

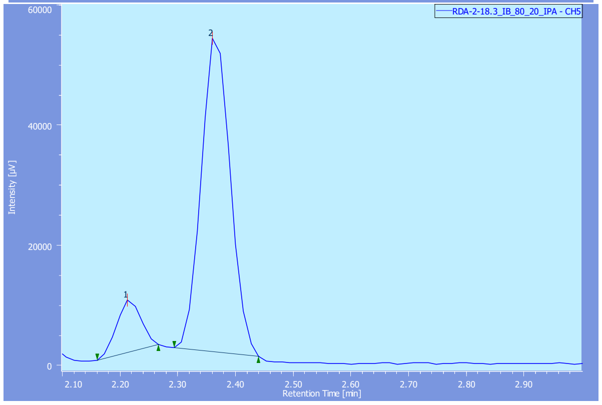

dd) Chiral SFC analysis of enzymatically-produced **5j** (*top*) and racemic **5j** (*bottom*):

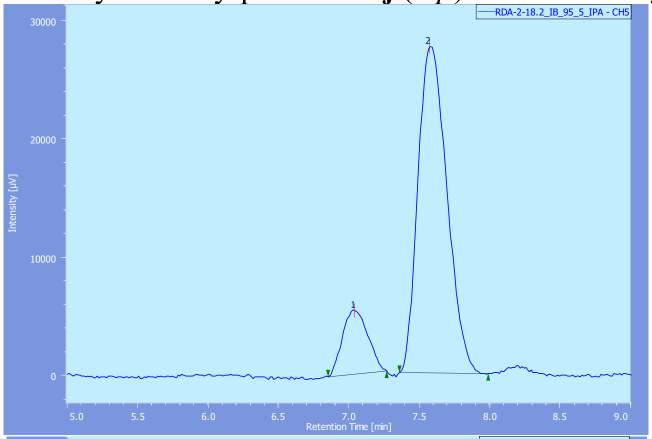

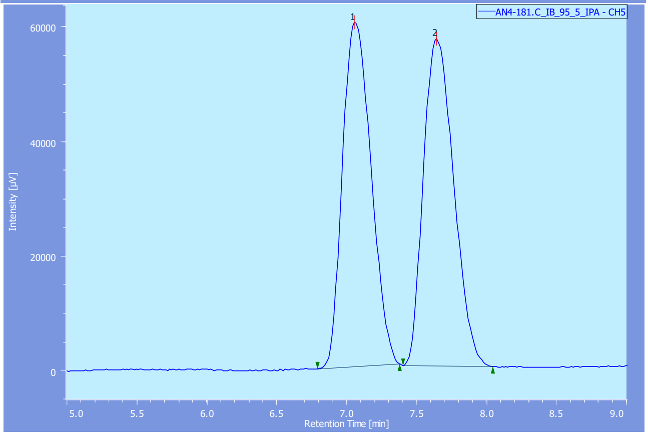

ee) Chiral SFC analysis of enzymatically-produced **5k** (*top*) and racemic **5k** (*bottom*):

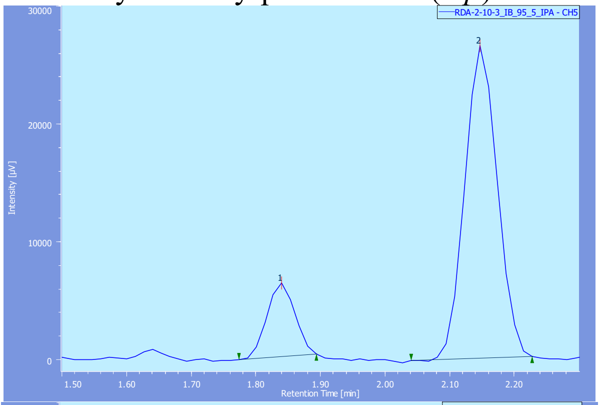

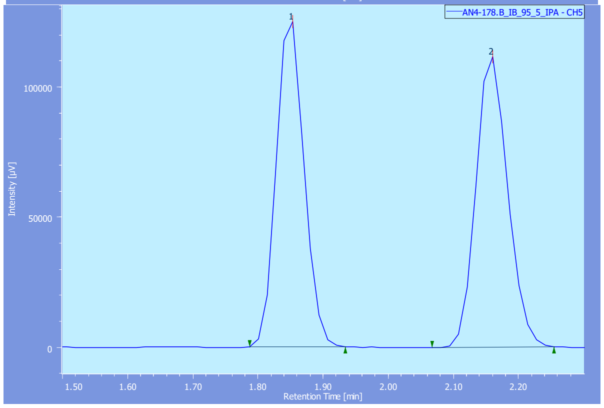

ff) Chiral SFC analysis of chemoenzymatically-produced **7** (*top*) and racemic **7** (*bottom*):

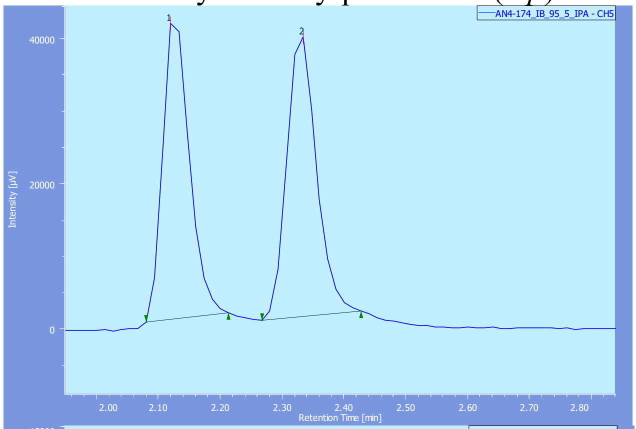

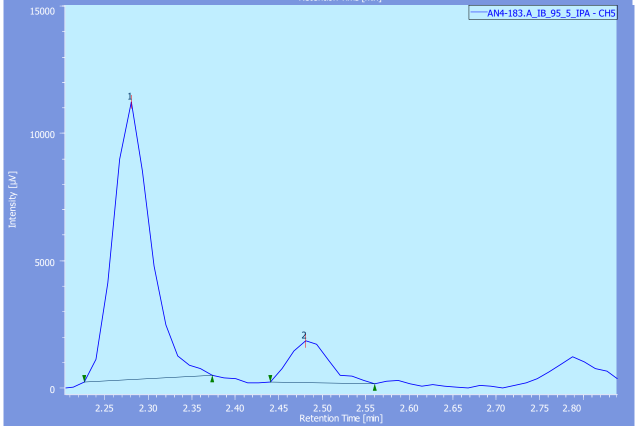

gg) Chiral SFC analysis of chemoenzymatically-produced **8** (*top*) and racemic **8** (*bottom*):

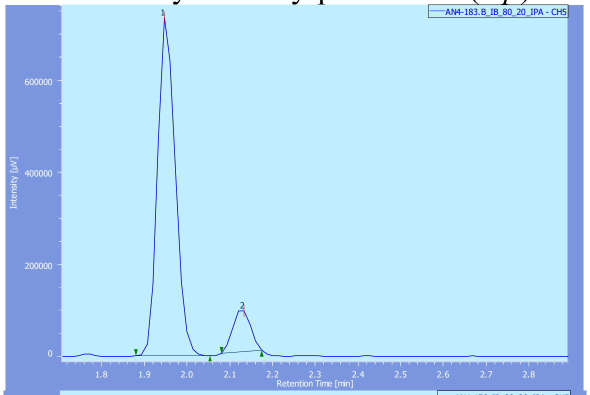

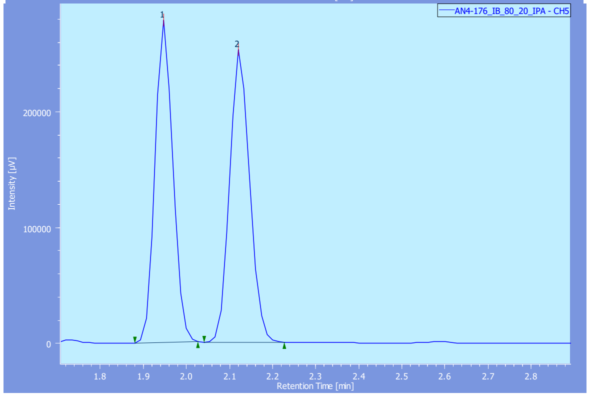

hh) Chiral SFC analysis of chemoenzymatically-produced **9** (*top*) and racemic **9** (*bottom*):

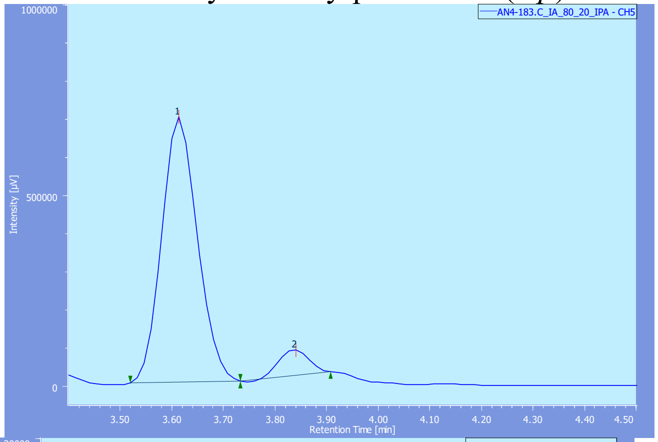

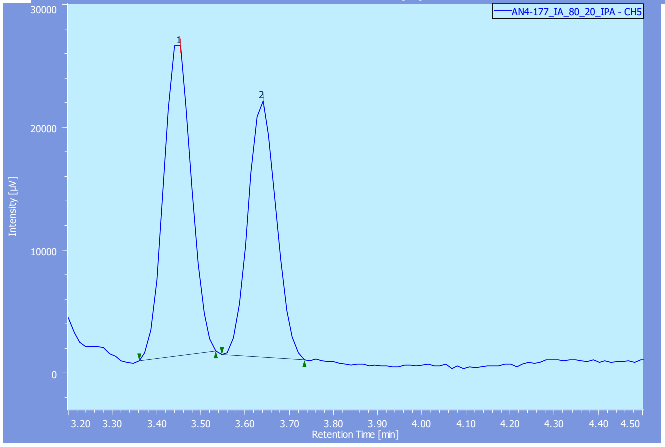

Figure S10. DFT-generated Fe-ylide reaction intermediate models and corresponding relative energies. Figures are are generated using CYLview (Legault, C. Y. CYLview, 1.0b; Université de Sherbrooke: Québec, Montreal, Canada, 2009. URL http://www.cylview.org).

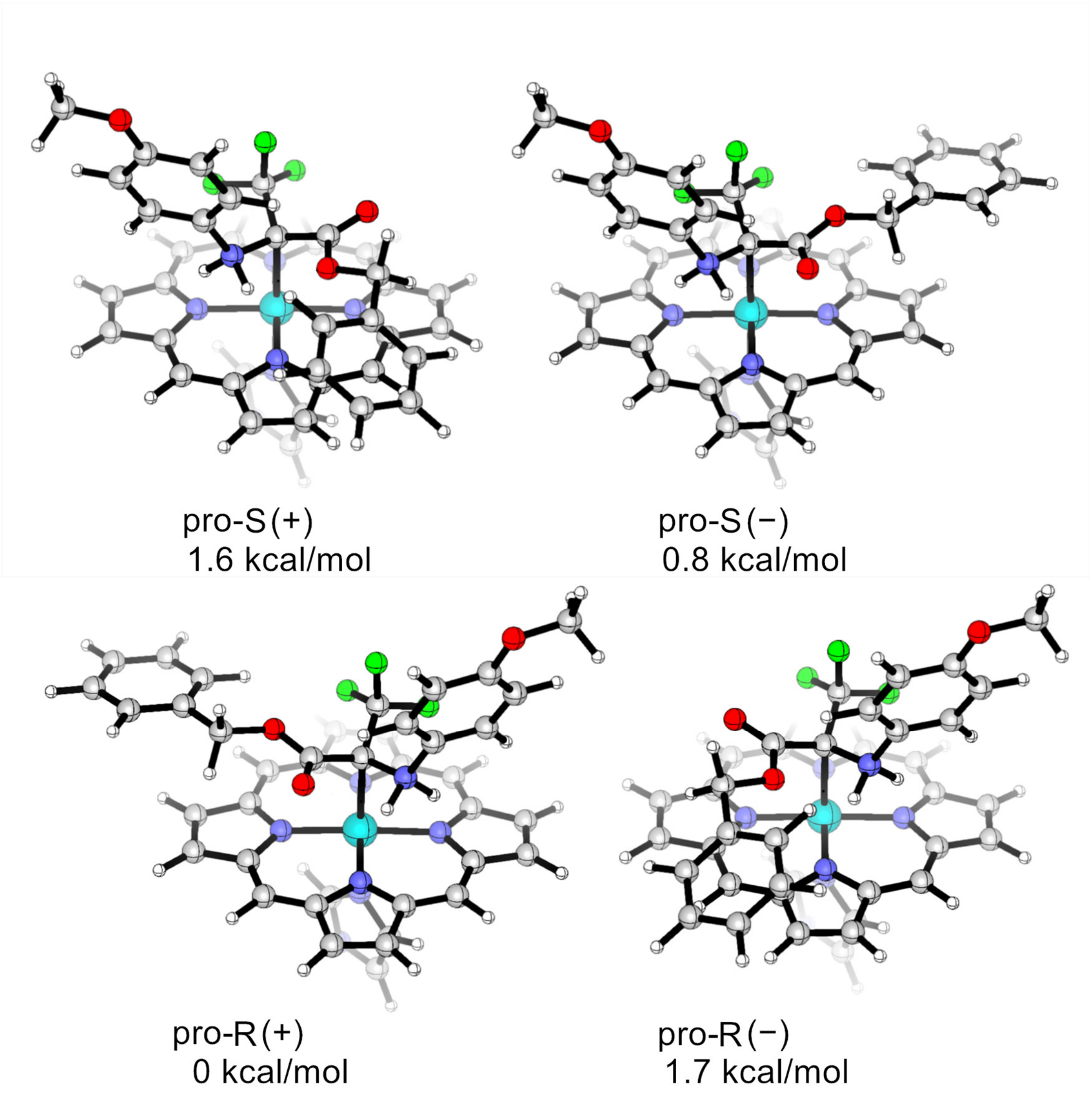

Figure S11. Rosetta model of *Ht-Cc552*(G50T,M59G,P60E,Q62R) variant complexed with (A) pro*R*-rot3(−) (**2c**/**1c**)-derived ylide (green stick models) overlayed with pro*S*-rot1(+) (**2c**/**1c**)-derived ylide (yellow stick models); (B) pro*R*-rot3(−) (**2c**/**1c**)-derived ylide (yellow stick models) and pro*R*-rot3(−) (**2g**/**1c**)-derived ylide (green stick models); (C) pro*R*-rot2(−) (**2g**/**1c**)-derived ylide (yellow stick models); and (D) pro*S*-rot1(+) (**2g**/**1c**)-derived ylide (green stick models). Energies are reported in the Rosetta energy unit (REU).

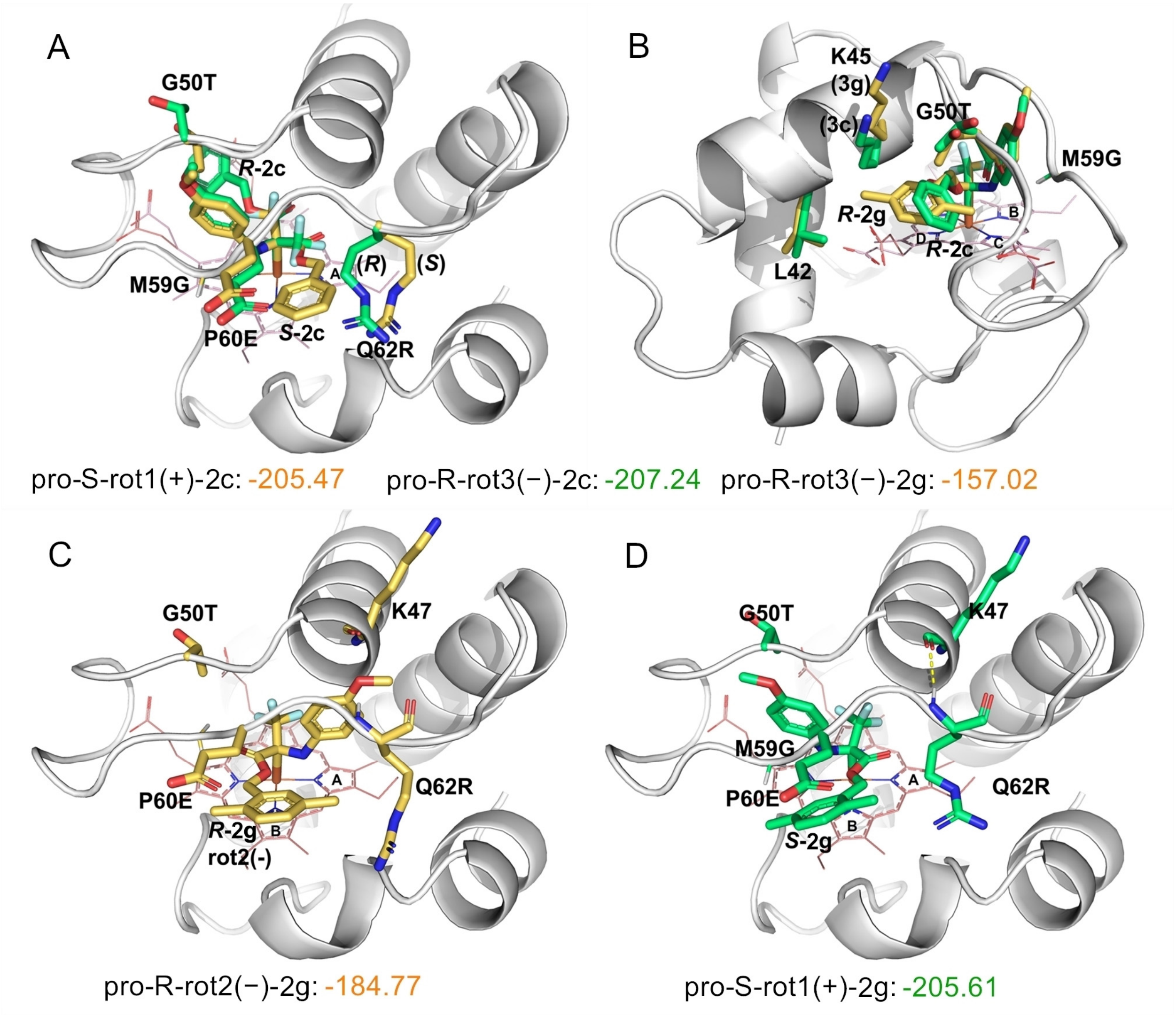

Figure S12. Per-residue energy decomposition comparison of Rosetta-generated models of the engineered *Ht-Cc552*(G50T,M59G,P60E,Q62R) variant complexed with the heme-ylide intermediates. (A-B) pro*R*-rot3(−) (**2c**/**1c**)-derived ylide (red line), pro*S*-rot1(+) (**2c**/**1c**)-derived ylide (purple line) and the energy difference (blue line). Panel B highlights region between residue 41-66. (C-D) pro*R*-rot3(−) (**2c**/**1c**)-derived ylide (red line), pro*R*-rot3(−) (**2g**/**1c**)-derived ylide (purple line) and the energy difference (blue line). Panel D highlights region between residue 41-66. (E-F) pro*R*-rot2(−) (**2g**/**1c**)-derived ylide (red line), pro*S*-rot1(+) (**2g**/**1c**)-derived ylide (purple line) and the energy difference (blue line). Panel F highlights region between residue 41-66. Energies are reported in the Rosetta energy unit (REU).

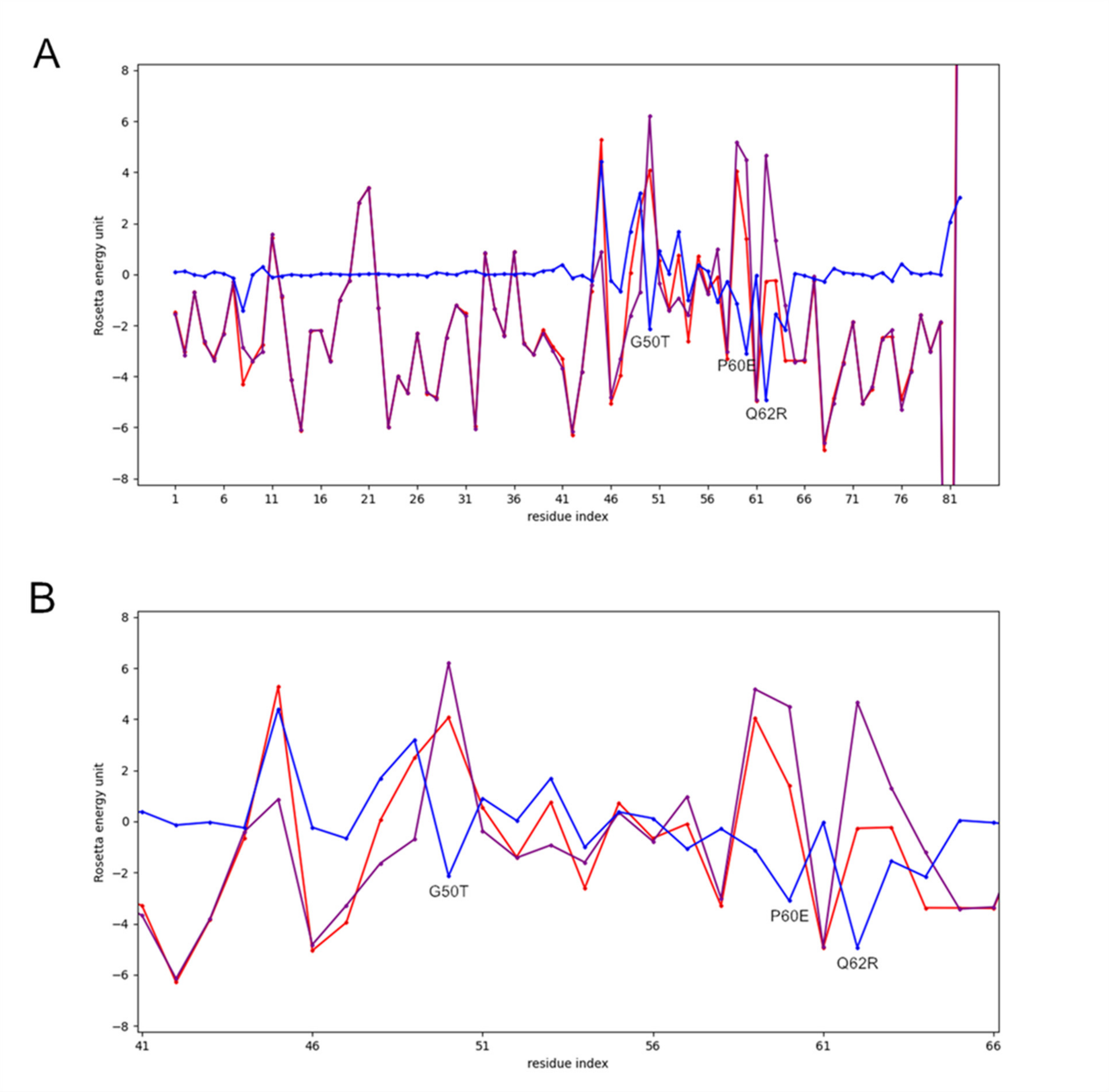

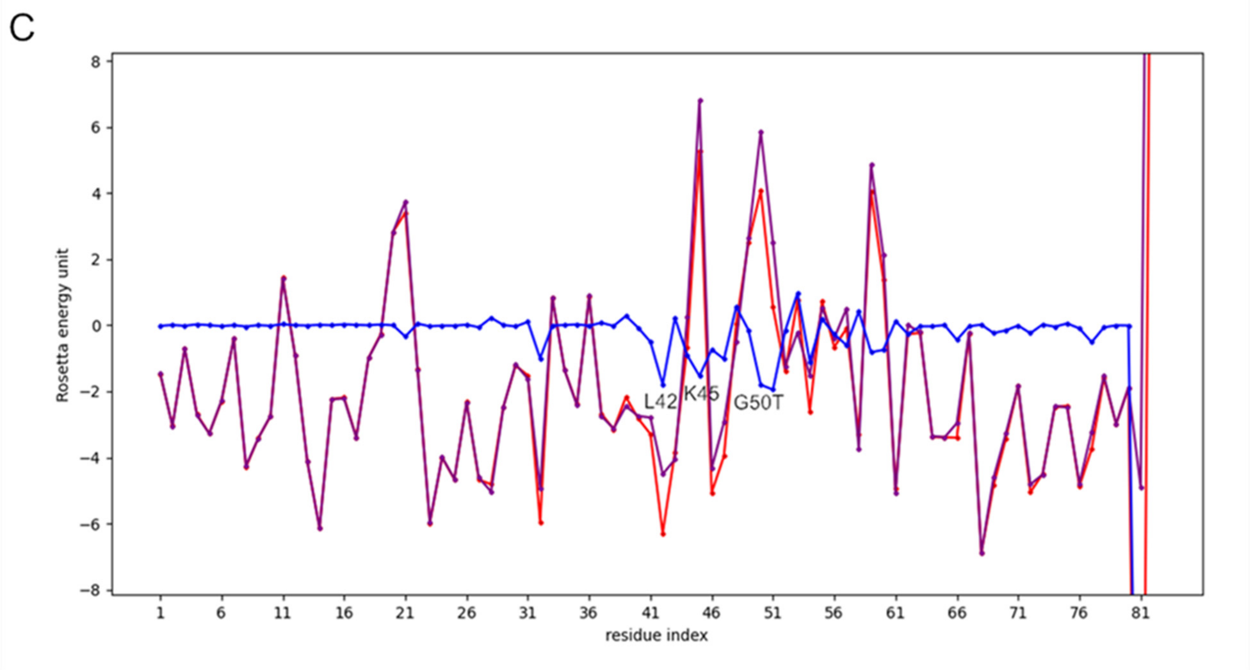
C
Rosetta energy unit
residue index
L42
K45
G50T

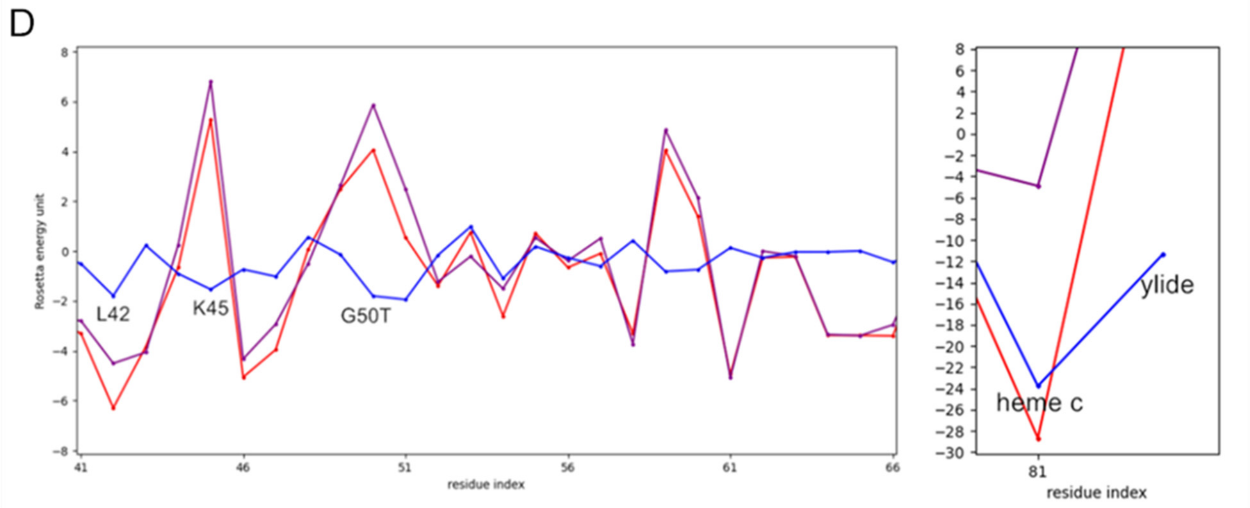
D
Rosetta energy unit
residue index
L42
K45
G50T
heme c
ylide

E

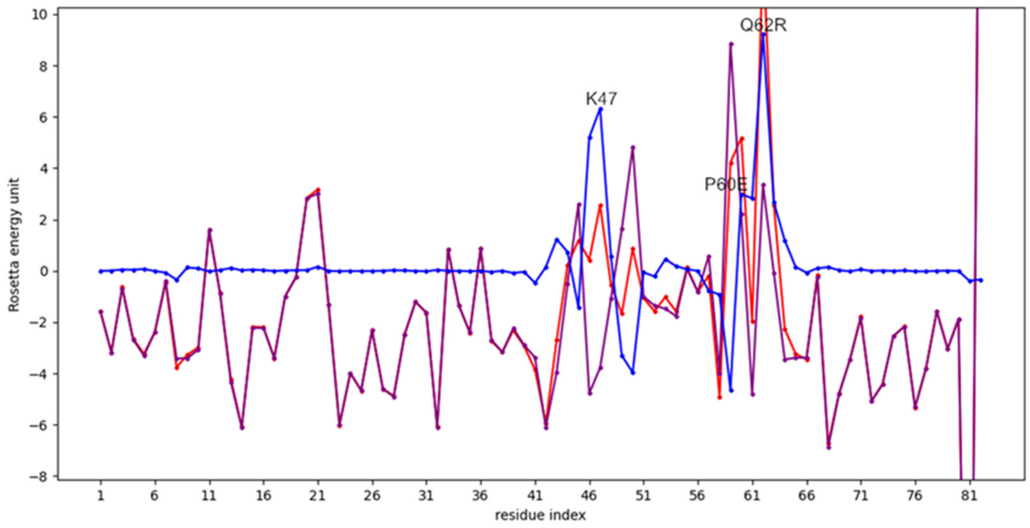
Q62R
K47
P60E
Rosetta energy unit
residue index

F

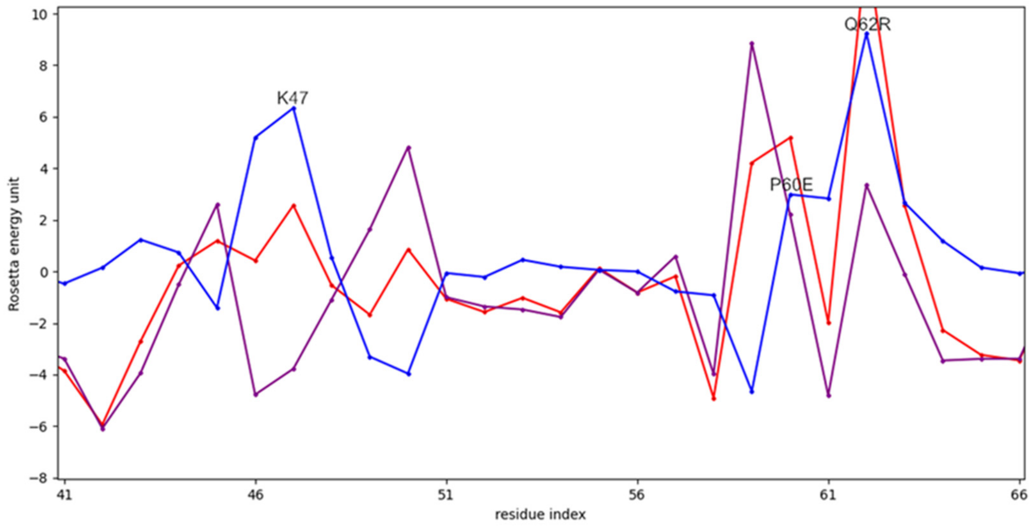
Q62R
K47
P60E
Rosetta energy unit
residue index

Figure S13. SFC traces of enantioenriched α-trifluoromethyl-α-amino acid **11** derived from hydrolysis of the enzymatic N–H insertion products obtained using (A) ethyl 2-diazo-3,3,3-trifluoropropanoate (**2a**) and (B) benzyl 2-diazo-3,3,3-trifluoropropanoate (**2c**).

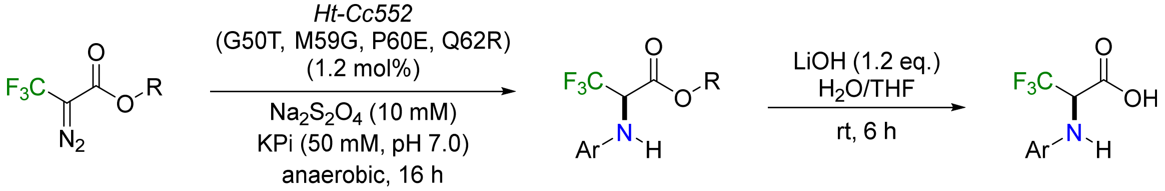

2a, 2c R: Et, Bn **14**

A

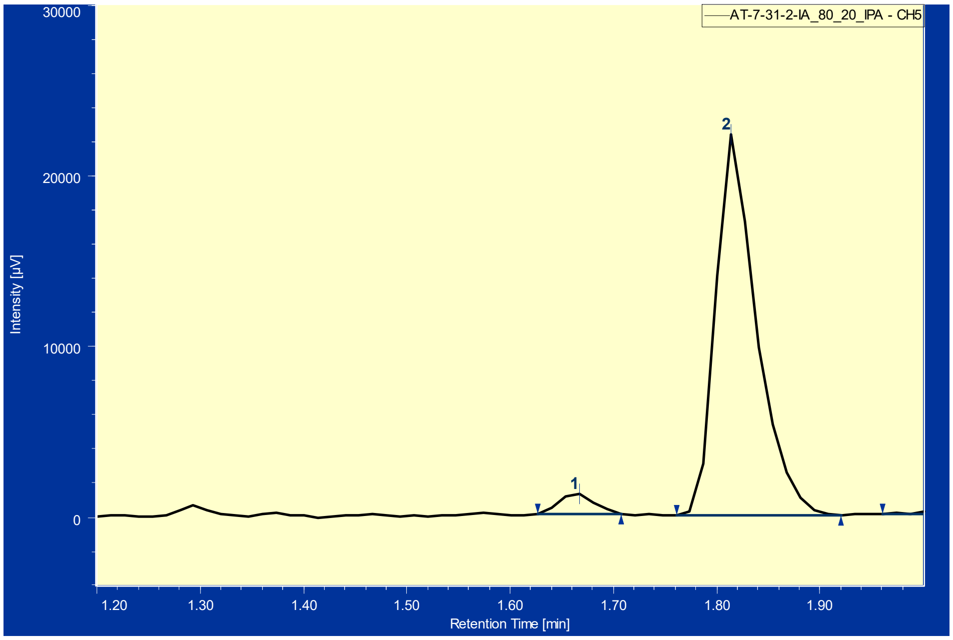

B

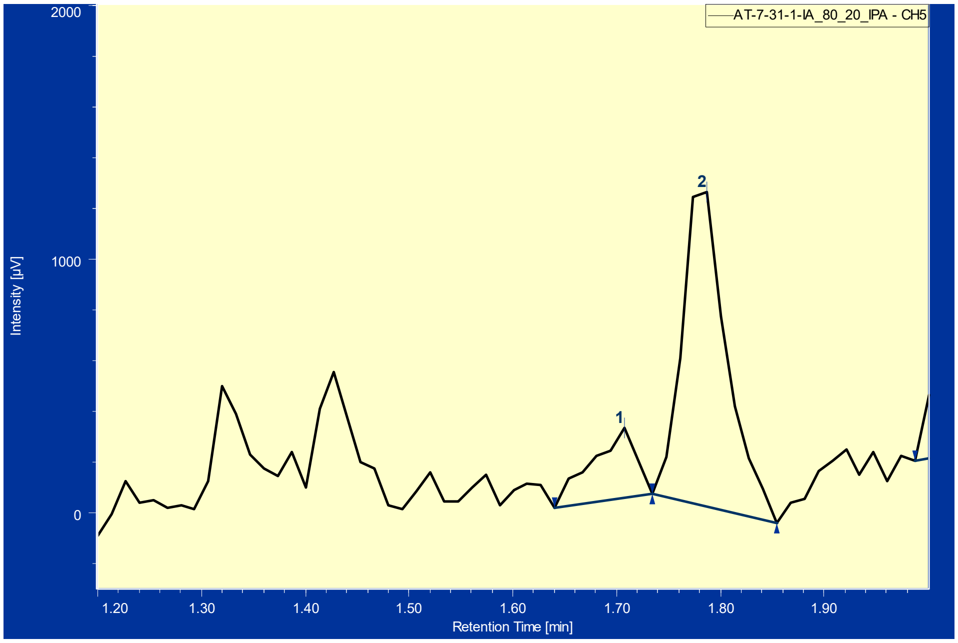

Figure S14. SFC traces of enantioenriched β-trifluoromethyl-β-amino alcohol **8** derived from reduction of the enzymatic N–H insertion products obtained using (A) cyclohexyl 2-diazo-3,3,3-trifluoropropanoate (**2b**), (B) benzyl 2-diazo-3,3,3-trifluoropropanoate (**2c**), (C) naphthalen-1-ylmethyl 2-diazo-3,3,3-trifluoropropanoate (**2d**), and (D) 2,5-dimethylbenzyl 2-diazo-3,3,3-trifluoropropanoate (**2g**).

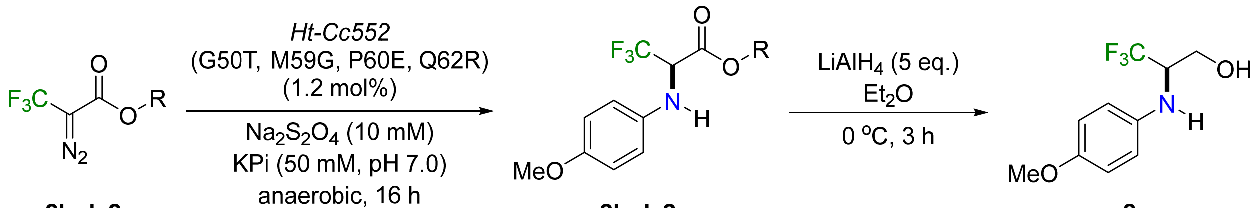

A

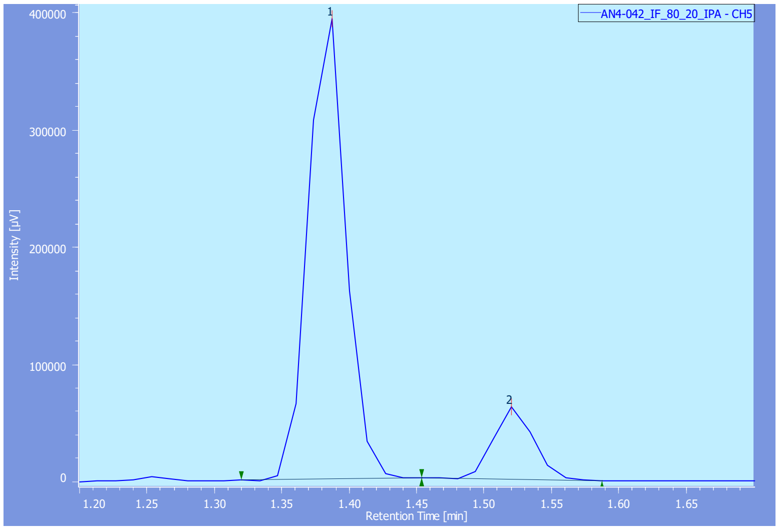

B

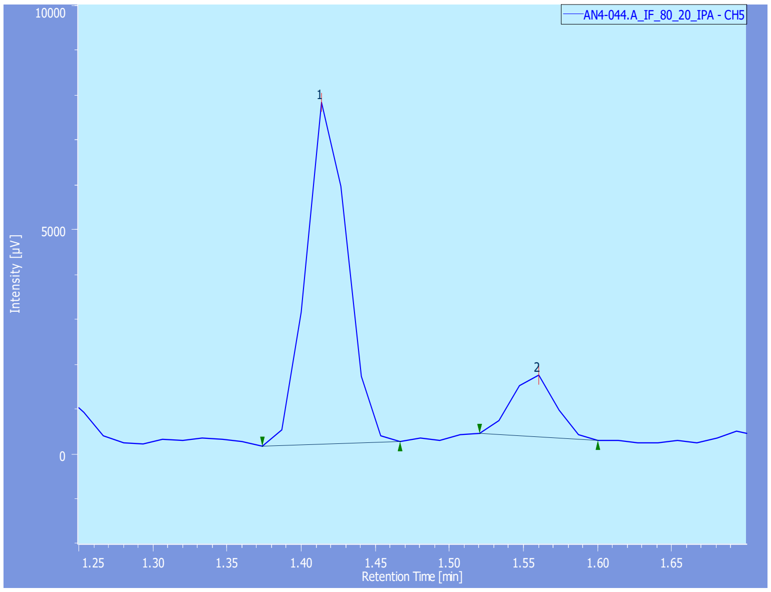

C

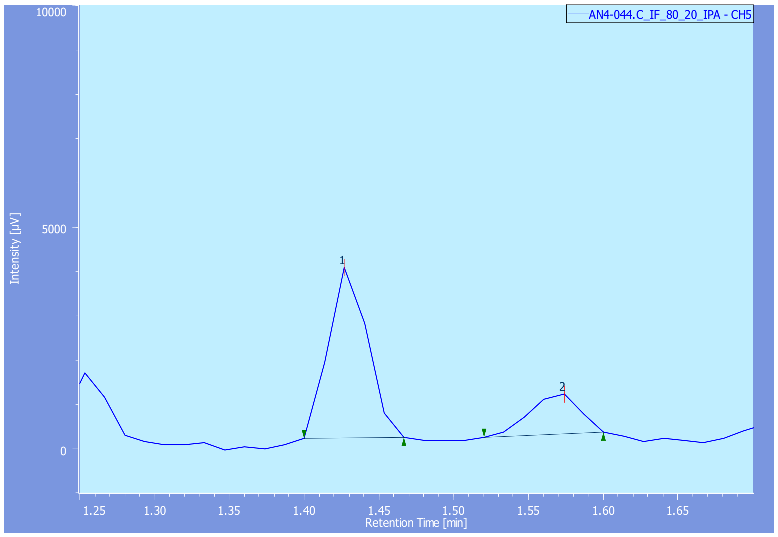
AN4-044.C_IF_80_20_IPA - CH5
10000
5000
0
Intensity [µV]
1
2
1.25
1.30
1.35
1.40
1.45
1.50
1.55
1.60
1.65
Retention Time [min]

D

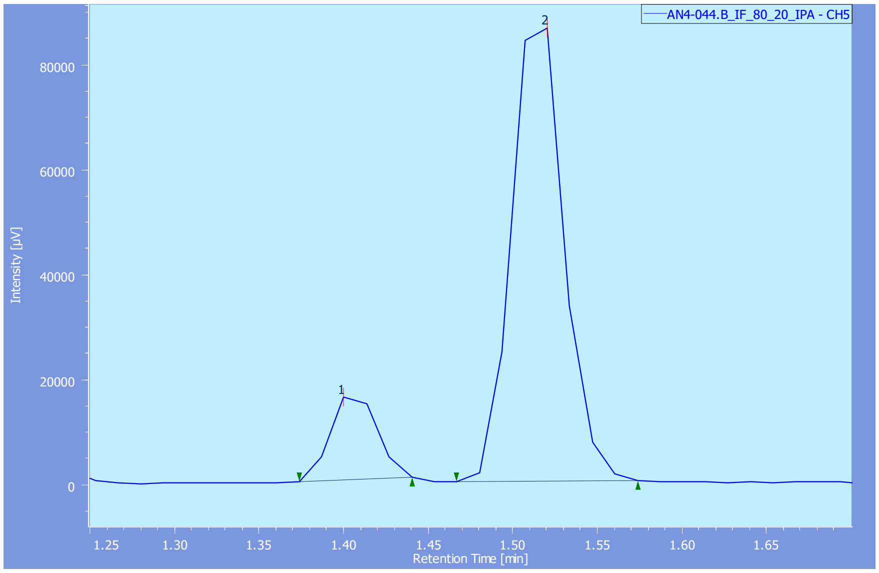
AN4-044.B_IF_80_20_IPA - CH5
80000
60000
40000
20000
0
Intensity [µV]
1
2
1.25
1.30
1.35
1.40
1.45
1.50
1.55
1.60
1.65
Retention Time [min]

Figure S15. HPLC traces of enantioenriched β-trifluoromethyl-β-amino alcohol **8** derived from reduction of the enzymatic N–H insertion products obtained using (A) 2-dimethylbenzyl 2-diazo-3,3,3-trifluoropropanoate (**2e**), (B) 2,4-dimethylbenzyl 2-diazo-3,3,3-trifluoropropanoate (**2f**), (C) 2,5-dimethylbenzyl 2-diazo-3,3,3-trifluoropropanoate (**2g**), and (D) 2,4,6-dimethylbenzyl 2-diazo-3,3,3-trifluoropropanoate (**2h**).

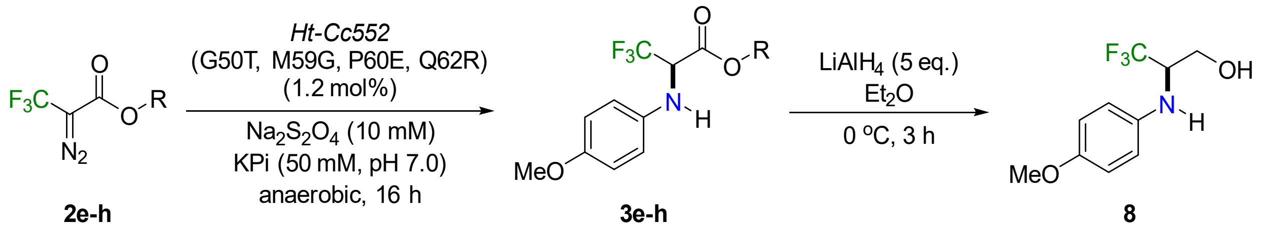

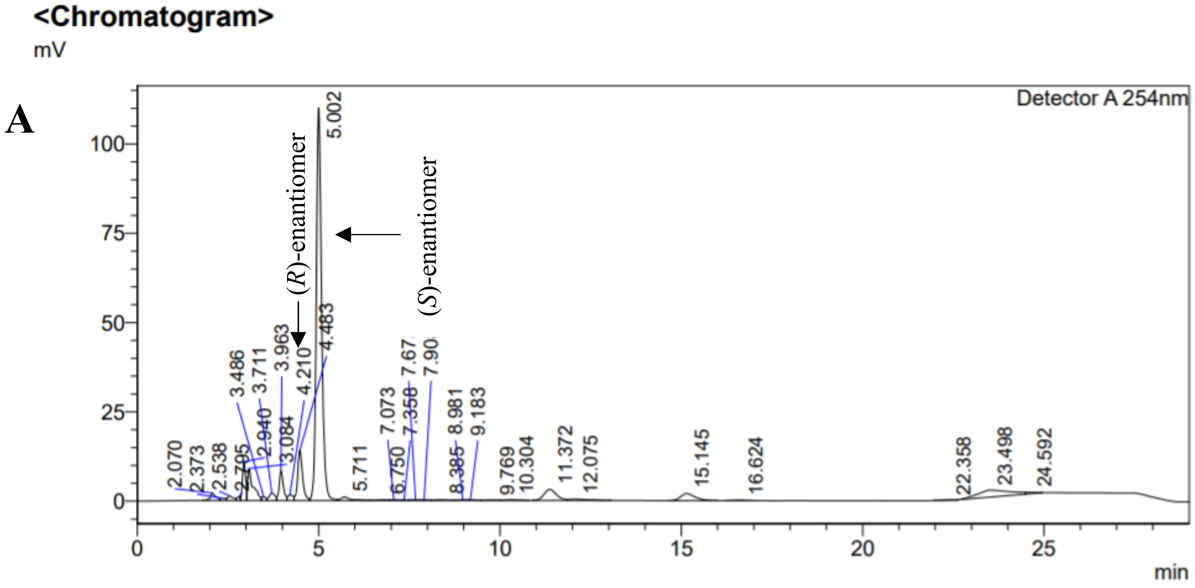

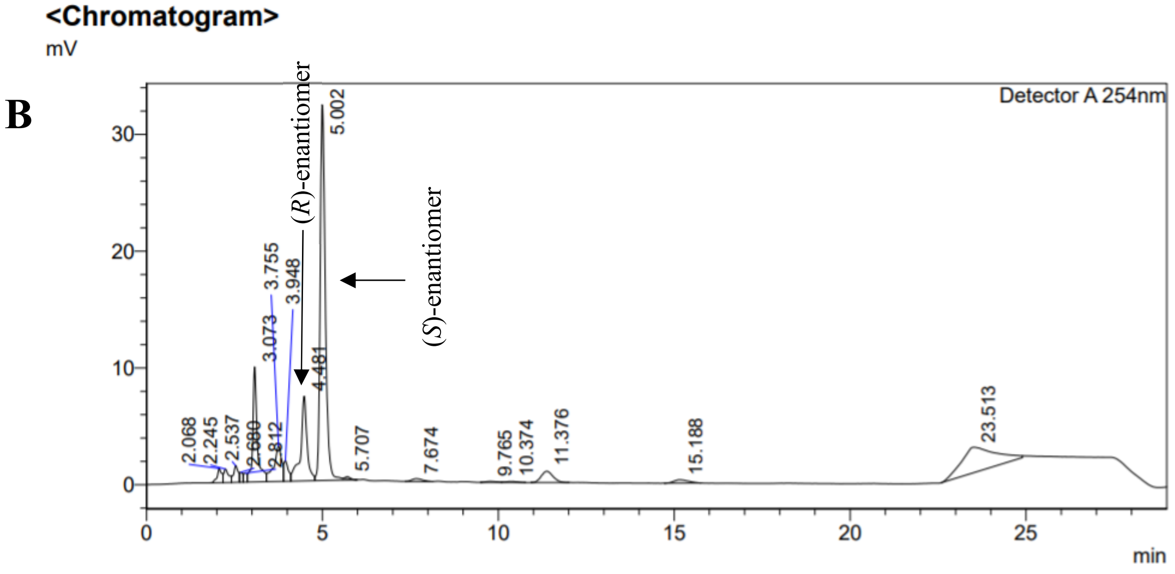

<Chromatogram>

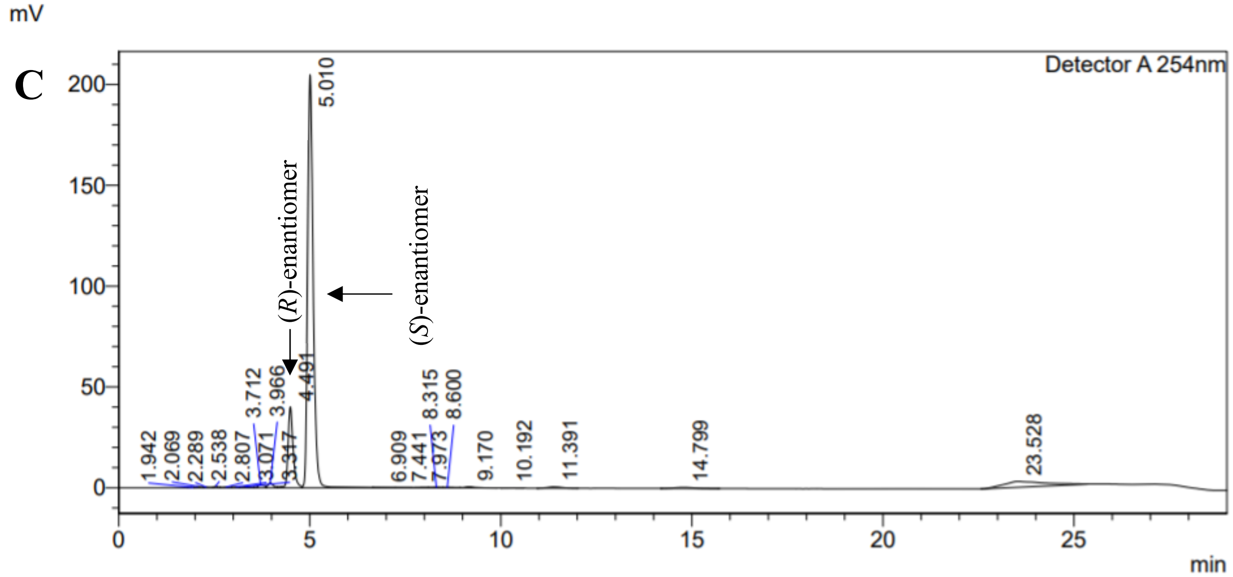

<Chromatogram>

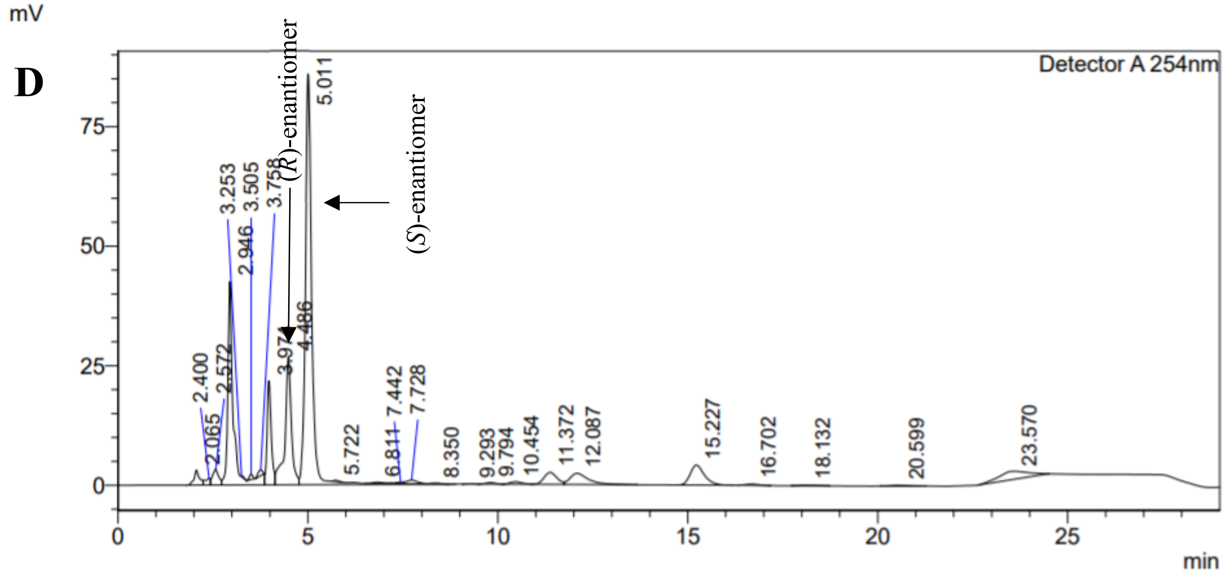

Table S1. Survey of reaction conditions for *Ht-Cc552*(G50T,M59G,P60E,Q62R)-catalyzed stereoselective N−H carbene insertion. Standard reaction conditions (std): 5 mM *p*-anisidine (**1**), 10 mM BnDTP (**2c**), 60 µM purified *Ht-Cc552* variant in KPi buffer (50 mM, pH 7), room temperature, 16 hours under Ar (g) pressure.* Citrate buffer (50 mM, pH 5). ^ Borate buffer (50 mM, pH 9).

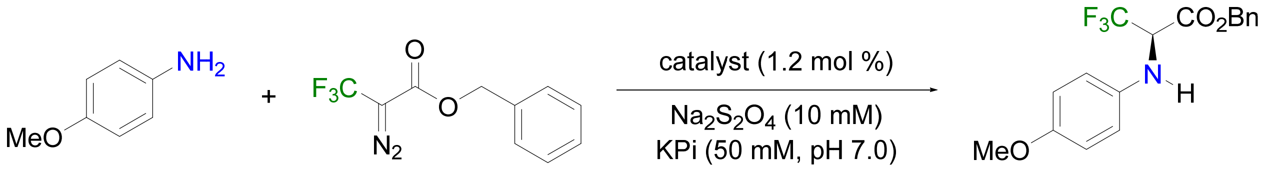

1a **2c** **3c**

Entry	*Ht-Cc552* catalyst	Conditions	Yield (%)	TON	*e.r.*
**1**	none	std	0	n.d.	n.d.
**2**	G50T,M59G,P60E,Q62R	std	83	70	91:9
**3**	G50T,M59G,P60E,Q62R	4 °C	74	62	90:10
**4**	G50T,M59G,P60E,Q62R	37 °C	63	53	89:11
**5**	G50T,M59G,P60E,Q62R	50 °C	24	20	86:14
**6**	G50T,M59G,P60E,Q62R	pH 5.0*	16	13	89:11
**7**	G50T,M59G,P60E,Q62R	pH 6.0	58	48	89:11
**8**	G50T,M59G,P60E,Q62R	pH 8.0	68	57	90:10
**9**	G50T,M59G,P60E,Q62R	pH 9.0^	59	49	89:11
**10**	G50T,M59G,P60E,Q62R	0.1 mol % cat.	57	574	89:12
**11**	G50T,M59G,P60E,Q62R	0.04 mol % cat.	30	752	91:9

Table S2. Rosetta-calculated energies (in REU) of engineered *Ht-Cc552* variant complexed with (**2**/**1c**)-derived metallic ylide reaction intermediates. The lowest energy value is highlighted in bold text while the energy of the most competitive state is underlined.

Diazo substrate	pro*R*-rot1 (+)	pro*R*-rot1 (−)	pro*R*-rot2 (+)	pro*R*-rot2 (−)	pro*R*-rot3 (+)	pro*R*-rot3 (−)	pro*R*-rot4 (+)	pro*R*-rot4 (−)
**2a**	-162.42	-169.77	-184.70	-202.46	-201.90	**-220.89**	-174.61	-179.92
**2c**	-157.51	-111.86	-168.97	-183.27	-166.32	**-207.24**	-173.14	-166.98
**2e**	-138.15	-94.57	-156.77	<u>-189.69</u>	-101.68	-187.12	-98.06	-164.02
**2f**	-160.27	-121.54	-153.63	<u>-190.77</u>	-157.21	-122.31	-105.60	-161.75
**2g**	-138.44	-138.36	-143.95	<u>-184.77</u>	-145.62	-157.02	-140.90	-146.24
**2h**	-138.52	-61.39	-97.59	<u>-169.76</u>	-87.42	-110.24	-87.59	-123.60

Diazo substrate	pro*S*-rot1 (+)	pro*S*-rot1 (−)	pro*S*-rot2 (+)	pro*S*-rot2 (−)	pro*S*-rot3 (+)	pro*S*-rot3 (−)	pro*S*-rot4 (+)	pro*S*-rot4 (−)
**2a**	-210.93	<u>-211.69</u>	-148.15	-161.17	-199.81	-201.94	-192.86	-198.74
**2c**	<u>-205.47</u>	-193.99	-145.85	-133.01	-173.24	-188.66	-172.89	-188.24
**2e**	**-204.22**	-196.13	-111.66	-154.66	-161.97	-185.91	-130.84	-111.68
**2f**	**-206.28**	-130.91	-143.53	-134.32	-164.14	-118.27	-103.44	-119.84
**2g**	**-205.61**	-198.40	-136.92	-149.93	-162.04	-178.95	-106.49	-114.92
**2h**	**-185.70**	-68.12	-95.46	-122.15	-115.14	-122.50	-55.43	-91.75

Table S3. Sequence of the oligonucleotides used for the preparation of engineered *Ht-Cc552* variants.

Entry	Primer	Sequence
1	Htcytc_M59GQ62(NDT)_for	GTGGGGTTCTGTTCCCGGGCCTCCTNDTAATGTAACC G
2	Htcytc_M59GQ62(VMA)_for	GTGGGGTTCTGTTCCCGGGCCTCCTVMAAATGTAACC G
3	Htcytc_M59GQ62(ATG)_for	GTGGGGTTCTGTTCCCGGGCCTCCTATGAATGTAACC G
4	Htcytc_M59GQ62(TGG)_for	GTGGGGTTCTGTTCCCGGGCCTCCTTGGAATGTAACC G
5	Htcytc_M59G_rev	CCCGGGAACAGAACCCCACACACCAGAACC
6	HtcytcNdeI_for	GAAGGAGATATACATATGAAGATCAGCATCTATGCC ACTC
7	HtcytcXhoI_rev	GGTGGTGCTCGAGAGAACCCTTTATGG
8	Htcytc_M59GQ62RP60(NDT)_for	GGGGTTCTGTTCCCGGGNDTCCTCGTAATGTAACCGA TG
9	Htcytc_M59GQ62RP60(VMA)_for	GGGGTTCTGTTCCCGGGVMACCTCGTAATGTAACCG ATG
10	Htcytc_M59GQ62RP60(ATG)_for	GGGGTTCTGTTCCCGGGATGCCTCGTAATGTAACCGA TG
11	Htcytc_M59GQ62RP60(TGG)_for	GGGGTTCTGTTCCCGGGTGGCCTCGTAATGTAACCGA TG
12	Htcytc_M59GQ62RP61(NDT)_for	GGGGTTCTGTTCCCGGGCCTNDTCGTAATGTAACCGA TG
13	Htcytc_M59GQ62RP61(VMA)_for	GGGGTTCTGTTCCCGGGCCTVMACGTAATGTAACCG ATG
14	Htcytc_M59GQ62RP61(ATG)_for	GGGGTTCTGTTCCCGGGCCTATGCGTAATGTAACCGA TG
15	Htcytc_M59GQ62RP61(TGG)_for	GGGGTTCTGTTCCCGGGCCTTGGCGTAATGTAACCG
16	Htcytc_G50(NDT)M59GP60EQ62R_ for	GATAAAGAAGGGCNDTTCTGGTGTGTGGGGTTCTGTT CCCGGG
17	Htcytc_G50(NDT)M59GP60EQ62R_ rev	CCCCACACACCAGAAHNGCCCTTCTTTATCTTGCCAG CCAG
18	Htcytc_G50(VMA)M59GP60EQ62R _for	GATAAAGAAGGGCVMATCTGGTGTGTGGGGTTCTGT TCCCGGG
19	Htcytc_G50(VMA)M59GP60EQ62R _rev	CCCCACACACCAGATKBGCCCTTCTTTATCTTGCCAG CCAG
20	Htcytc_G50(ATG)M59GP60EQ62R_ for	GATAAAGAAGGGCATGTCTGGTGTGTGGGGTTCTGTT CCCGGG
21	Htcytc_G50(ATG)M59GP60EQ62R_ rev	CCCCACACACCAGACATGCCCTTCTTTATCTTGCCAG CCAG
22	Htcytc_G50(TGG)M59GP60EQ62R_ for	GATAAAGAAGGGCTGGTCTGGTGTGTGGGGTTCTGTT CCCGGG
23	Htcytc_G50(TGG)M59GP60EQ62R_ rev	CCCCACACACCAGACCAGCCCTTCTTTATCTTGCCAG CCAG

X-ray crystallographic analyses

Data Collection. A crystal (0.219 x 0.119 x 0.109 mm^3) was placed onto a thin glass optical fiber or a nylon loop and mounted on a Rigaku XtaLab Synergy-S Dualflex diffractometer equipped with a HyPix-6000HE HPC area detector for data collection at 100.00(10) K. A preliminary set of cell constants and an orientation matrix were calculated from a small sampling of reflections. A short pre-experiment was run, from which an optimal data collection strategy was determined. The full data collection was carried out using a PhotonJet (Cu) X-ray source with frame times of 0.19 and 0.75 seconds and a detector distance of 31.2 mm. Series of frames were collected in 0.50º steps in ω at different 2θ, κ, and ϕ settings. After the intensity data were corrected for absorption, the final cell constants were calculated from the xyz centroids of 8491 strong reflections from the actual data collection after integration. See **Table S4** for additional crystal and refinement information.

Structure Solution and Refinement. The structure was solved using ShelXT[1] and refined using ShelXL.[2] The space group $P2_12_12_1$ was determined based on systematic absences. Most or all non-hydrogen atoms were assigned from the solution. Full-matrix least squares / difference Fourier cycles were performed which located any remaining non-hydrogen atoms. All non-hydrogen

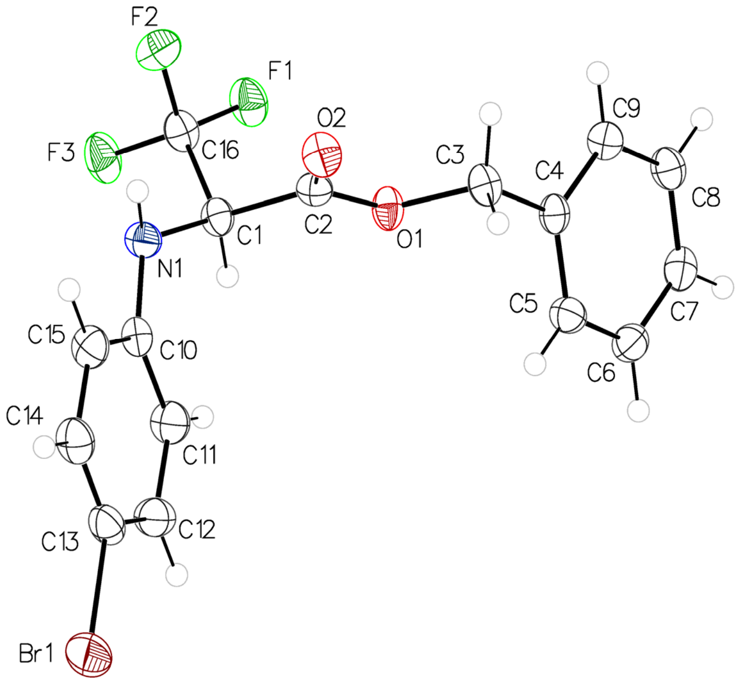

atoms were refined with anisotropic displacement parameters. The N−H hydrogen atom was refined freely; the lack of hydrogen-bonding acceptor appears to be due to the steric bulk around the N−H donor. All other hydrogen atoms were placed in ideal positions and refined as riding atoms with relative isotropic displacement parameters. The final full matrix least squares refinement converged to $R1 = 0.0309$ (F^2, $I > 2\sigma(I)$) and $wR2 = 0.0843$ (F^2, all data). Structure manipulation and figure generation were performed using Olex2.[3] Unless noted otherwise all structural diagrams containing anisotropic displacement ellipsoids are drawn at the 50% probability level.

Table S4. Crystal Data and Structure Refinement for 4f. Cambridge Crystallographic Data Centre (CCDC) entry: 2113422

Identification code	fasatv02	
Empirical formula	C16 H13 Br F3 N O2	
Formula weight	388.18	
Temperature	100.00(10) K	
Wavelength	1.54184 Å	
Crystal system	orthorhombic	
Space group	$P2_12_12_1$	
Unit cell dimensions	a = 5.16770(10) Å	α = 90°
	b = 8.8164(2) Å	β = 90°
	c = 34.3024(9) Å	γ = 90°
Volume	1562.83(6) $Å^3$	
Z	4	
Density (calculated)	1.650 Mg/m^3	
Absorption coefficient	3.973 mm^{-1}	
F(000)	776	
Crystal color, morphology	colourless, needle	
Crystal size	0.219 x 0.119 x 0.109 mm^3	
Theta range for data collection	2.576 to 77.444°	
Index ranges	$-6 \leq h \leq 4$, $-11 \leq k \leq 11$, $-40 \leq l \leq 43$	
Reflections collected	13033	
Independent reflections	3272 [R(int) = 0.0404]	
Observed reflections	3109	
Completeness to theta = 74.504°	99.9%	
Absorption correction	Multi-scan	
Max. and min. transmission	1.00000 and 0.21306	
Refinement method	Full-matrix least-squares on F^2	
Data / restraints / parameters	3272 / 0 / 212	
Goodness-of-fit on F^2	1.094	
Final R indices [I>2sigma(I)]	R1 = 0.0309, wR2 = 0.0829	
R indices (all data)	R1 = 0.0328, wR2 = 0.0843	
Absolute structure parameter	-0.044(13)	
Largest diff. peak and hole	0.444 and -0.427 e.$Å^{-3}$	

Reagents and Analytical methods

General information. All chemicals and reagents were purchased from commercial suppliers (Sigma-Aldrich, Alfa Aeser, J.T. Baker, Acros, Oakwood) and used without any further purification, unless otherwise stated. All reactions were carried out under argon pressure in oven-dried glassware with magnetic stirring using standard gas-tight syringes, cannulae, and septa. ^{1}H, ^{13}C, and ^{19}F NMR spectra were measured on a Bruker DPX-400 instrument (operating at 400 MHz for ^{1}H, 100 MHz for ^{13}C, and 375 MHz for ^{19}F) or a Bruker DPX-500 instrument (operating at 500 MHz for ^{1}H and 125 MHz for ^{13}C). Tetramethylsilane (TMS) served as the internal standard (0 ppm) for ^{1}H NMR, CDCl3 was used as the internal standard (77.0 ppm) for ^{13}C NMR, and trifluorotoluene served as the internal standard (0 ppm) for ^{19}F NMR. Column chromatography purification was carried out using AMD Silica Gel 60 Å 230-400 mesh. Thin Layer Chromatography (TLC) was carried out using Merck Millipore TLC silica gel 60 F254 glass plates.

Chiral SFC Methods. Product analysis was performed by using using a JASCO Analytical and Semi-Preparative Supercritical Fluid Chromatography (SFC) instrument equipped with a column oven (35 °C), photodiode array detector, a backpressure regulator (12.0 MPa), a carbon dioxide pump and a sample injection volume of 3 µL. Daicel Chiralpak IA, IB, IC or IF column (0.46 cm ID × 25 cm L) was used for measuring percent conversion, calculating TON, separation of enantiomers and determination of the enantiomeric ratios based on calibration curves constructed using chemically-synthesized racemic analytical standards (*vide infra*). All samples were eluted using an isocratic solvent system with the indicated modifier in liquid CO_2 at an elution rate of 4 mL/min and detected at λ = 220 nm. Total run time was 10.2 minutes. Modifier solvent percentages and retention times (t_R) for chiral SFC analyses of racemic analytical standards **3a-h, 4b-n, 5a-k, 7-9, 14** are reported in the table below:

Product	Column	Modifier Solvent (%)	t_R for enantiomer 1 (min)	t_R for enantiomer 2 (min)
**3a**	IA	IPA (2%)	2.75	3.2
**3b**	IA	IPA (10%)	2.05	2.17
**3c**	IA	IPA (10%)	2.75	3.09
**3d**	IA	IPA (10%)	4.57	4.82
**3e**	IA	IPA (5%)	4.68	5.08
**3f**	IA	IPA (5%)	4.89	5.77
**3g**	IA	IPA (10%)	2.40	2.55
**3h**	IA	IPA (5%)	2.01	2.08

Product	Column	Modifier Solvent (%)	t_R for enantiomer 1 (min)	t_R for enantiomer 2 (min)
**4b**	IA	IPA (2%)	6.25	7.71
**4c**	IA	IPA (3%)	3.40	3.85
**4d**	IA	IPA (1%)	3.92	4.24
**4e**	IA	IPA (1%)	2.68	2.95
**4f**	IA	IPA (1%)	6.52	8.25
**4g**	IA	IPA (3%)	5.79	6.81
**4h**	IA	IPA (2%)	4.89	5.44
**4i**	IA	IPA (1%)	4.85	5.21
**4j**	IA	IPA (5%)	2.92	3.40
**4k**	IA	IPA (1%)	6.47	7.61
**4l**	IB	IPA (20%)	2.37	2.73
**4m**	IB	IPA (20%)	1.92	2.05
**4n**	IB	IPA (5%)	1.85	2.16

Product	Column	Modifier Solvent (%)	t_R for enantiomer 1 (min)	t_R for enantiomer 2 (min)
**5a**	IA	IPA (5%)	1.89	1.97
**5c**	IF	IPA (5%)	2.25	2.38
**5d**	IA	IPA (5%)	2.55	2.65
**5e**	IA	IPA (15%)	1.60	1.73
**5f**	IA	IPA (1%)	2.44	2.56
**5g**	IA	IPA (3%)	5.07	5.40
**5h**	IA	IPA (2%)	4.40	4.53
**5i**	IB	IPA (20%)	2.21	2.37
**5j**	IB	IPA (5%)	7.05	7.64
**5k**	IB	IPA (5%)	2.85	2.07

Product	Column	Modifier Solvent (%)	t_R for enantiomer 1 (min)	t_R for enantiomer 2 (min)
**7**	IB	IPA (5%)	2.12	2.33
**8**	IB	IPA (20%)	1.95	2.12
**9**	IA	IPA (20%)	3.45	3.64
**14**	IA	IPA (20%)	1.68	1.82

Chiral GC Methods. Gas chromatography (GC) analysis were carried out using a Shimadzu GC-2010 gas chromatograph equipped with a FID detector, and a Cyclosil-B column (30 m x 0.25 mm x 0.25 µm film). The following GC methods were used for TON analysis for **4l-n, 5a, 5c-l**: 1 µL injection, injector temp.: 200 ºC, detector temp: 300 ºC. Gradient for Method A: column temperature set at 80 ºC for 2 min, then to 245 ºC at 40 ºC/min, then 245 ºC hold for 4 min. Total run time was 10.1 min. Gradient for Method B: column temperature set at 80 ºC for 2 min, then to 245 ºC at 40 ºC/min, then 245 ºC hold for 14 min. Total run time was 20.1 min. Gradient for Method C: column temperature set to 80 ºC, then to 245 ºC at 40 ºC/min, then 245 ºC hold for 10 minute. Total run time was 16.1 min.

Product	Method	t_R (min)
**5a**	A	8.30
**5c**	A	8.85
**5d**	A	8.74
**5e**	A	8.44
**5f**	B	10.17
**5g**	B	11.58
**5h**	B	10.62
**5i**	C	15.42
**5j**	C	15.11
**5k**	C	7.91
**4l**	C	12.69
**4m**	C	12.53
**4n**	C	7.31

Chiral HPLC Methods. Stereoisomer resolution for compound **8** was performed by Higher Performance Liquid Chromatography (HPLC) analysis, using a Prominence-I LC-2030 Plus, equipped with column oven (25 ºC), UV/PDA detector and a sample injection volume of 5 µL. Daicel Chiralpak AD-H column (0.46 cm ID × 15 cm L) was used for separation of enantiomers. All samples were eluted using an 15% isopropyl alcohol and 85% hexanes at an elution rate of 1 mL/min and detected at 254 nm. Total run time was 20 min.

Product	t_R for (*R*) isomer (min)	t_R for (*S*) isomer (min)
**8**	4.48	5.00

Stereochemical assignment of N–H insertion products. The absolute configuration of **3c** was determined via crystallographic analysis of the analog **4f**. The absolute configuration of **3a** (**Scheme 2**) was assigned based on chiral SFC analysis of its hydrolysis product and comparison

with that derived from **3c** (**Figure S10**). The absolute configuration of **3b** and **3d-h (Scheme 2**) was assigned based on chiral SFC and HPLC analysis of their ester reduction product **8** and comparison with that derived from **3c** (**Figure S11-12**).

Synthetic Procedures

Synthesis of ethyl 2-diazo-3,3,3-trifluoropropanoate (2a)

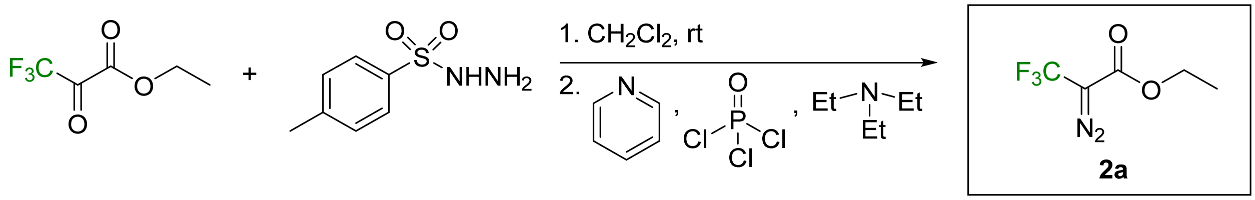

To a flame-dried two-neck 100 mL round bottom flask containing an oven-dried Teflon-coated magnetic stir bar was added tosyl hydrazide (1.310 g, 7.06 mmol, 1.2 eq). The flask was sealed with a rubber septa and purged with argon for 3 min. Anhydrous CH_2Cl_2 (40 mL) was added via a gas-tight syringe, and the reactant mixture was stirred for 5 min. Ethyl 3,3,3-trifluoropyruvate (0.78 mL, 5.88 mmol, 1.0 eq) was added via a gas-tight syringe. The reaction was stirred under argon overnight (~18 h) at room temperature, then pyridine (2.70 mL) was added followed by $POCl_3$ (0.60 mL, 5.88 mmol, 1.0 eq) dropwise via gas-tight syringe. The flask was equipped with a reflux condenser, and the reaction was refluxed at 60 °C for 30 min. The flask was cooled to room temperature, and triethylamine (1.00 mL, 7.06 mmol, 1.2 eq) was added dropwise via syringe. The reaction was refluxed for another 30 min at 60 °C, as the reaction turned from pale yellow to bright orange with white precipitate forming. After 30 min, the flask was cooled to room temperature, and the reaction mixture was transferred to a 250 mL separatory funnel. Water was added (~40 mL), the organic layer was collected, and the aqueous layer was extracted with CH_2Cl_2 (3X, 30 mL). The organic layers were pooled, washed with 1N HCl, sat. $NaHCO_3$, and sat. NaCl. The organic layer was collected and dried over anhydrous $MgSO_4$. The organic solvent was evaporated via rotary evaporation, using an ice-water bath and low vacuum. The crude product was purified via flash column chromatography using silica gel and a 10% Et_2O in pentane isocratic solvent system. After rotary evaporation using an ice-water bath, the purified diazo compound was obtained as a yellow liquid (964 mg, 90% yield).

^{1}H NMR (400 MHz, $CDCl_3$): δ 4.32-4.27 (*q*, J = 14.2, 7.1 Hz, 2H), 1.32 (*t*, J = 7.1 Hz, 3H). ^{13}C NMR (125 MHz, $CDCl_3$): δ 161.5, 126.6 (*q*, J = 535, 268 Hz), 62.8, 14.9 Diazo carbon not detected. ^{19}F NMR (375 MHz, $CDCl_3$): δ -57.7.

Synthesis of 2-diazo-3,3,3-trifluoropropanoate derivatives

Benzyl 2-diazo-3,3,3-trifluoropropanoate **2c** was synthesized in five steps starting from trifluoroacetic acid and *p*-anisidine (**Scheme S1**). Following a similar procedure, 2-diazo-3,3,3-trifluoropropanoate derivatives (**2b, 2d-h**) as well as racemic standards of 3,3,3-trifluoropropanoate (**3b-h**) were also generated.

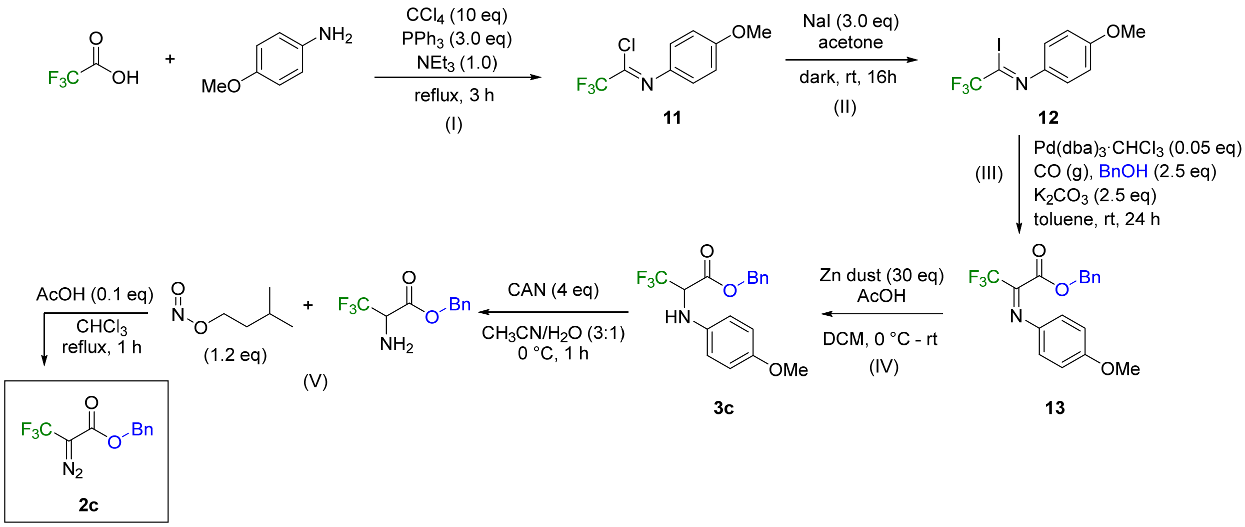

Scheme S1. Synthetic route for the preparation of 2-diazo-3,3,3-trifluoropropanoate **2c.**

I. Synthesis of 2,2,2-trifluoro-N-(4-methoxyphenyl)acetimidoyl chloride (**11**)

To a flame-dried 250 mL two-neck round-bottom flask containing an oven-dried Teflon-coated magnetic stir bar was added triphenylphosphine (31.945 g, 121.8 mmol, 3.0 eq) and *p*-anisidine (5.000 g, 40.6 mmol, 1.0 eq). The flask was sealed with rubber septa and purged with Ar (g) for 3 minutes, then charged with carbon tetrachloride (~100 mL) via gas tight syringe. The reaction mixture was stirred in an ice-water bath, and trifluoroacetic acid (3.1 mL, 40.6 mmol, 1.0 eq) was added via gas-tight syringe followed by slow addition of triethylamine (5.7 mL, 40.6 mmol, 1.0 eq). Upon complete addition of triethylamine, the reaction flask was equipped with a condenser and heated to a reflux (100 °C). The reaction was left stirring under an Ar (g) pressure for 3 h, monitoring using TLC (5% EtOAc in hexanes). The solvent was removed via rotary evaporation, the residue was diluted with hexanes (100 mL) and filtered through a pad of Celite using a medium porosity fritted-glass funnel, rinsing with hexane (3X, ~20 mL). The filtrate was concentrated via

rotary evaporation and purified via flash column chromatography using silica gel and a 5% diethyl ether in hexanes isocratic solvent system. After concentration under reduced pressure, the product was obtained as a yellow oil (6.9 g, 72% yield).

^{1}H NMR (400 MHz, $CDCl_3$): δ 7.33 (*dt*, J = 9.0, 3.3 Hz, 2H), 6.98 (*dt*, J = 9.0, 3.3 Hz, 2H), 3.84 (*s*, 3H). ^{13}C NMR (100 MHz, $CDCl_3$): δ 159.5, 135.4, 128.3 (*q*, J = 42.4 Hz), 124.3, 124.2, 121.1 (*q*, J = 275 Hz), 114.2, 55.4. ^{19}F NMR (375 MHz, $CDCl_3$): δ -71.5.

II. Synthesis of 2,2,2-trifluoro-N-(4-methoxyphenyl)acetimidoyl iodide (**12**)

To a flame-dried 250 mL round bottom flask containing an oven-dried Teflon-covered magnetic stir bar was added sodium iodide (18.257 g, 122.0 mmol, 3.0 eq) and 2,2,2-trifluoro-*N*-(4-methoxyphenyl)acetimidoyl chloride (6.987 g, 40.6 mmol, 1.0 eq). The flask was sealed with a rubber septum and purged with Ar (g) for 3 min. The flask was covered in aluminum foil, charged with anhydrous acetone (100 mL) via gas-tight syringe, and left to stir in the dark, under an Ar (g) atmosphere at room temperature. After 16 h, the reaction mixture was transferred to a separatory funnel and washed with saturated aq. $Na_2S_2O_3$, extracted with EtOAc (50 mL, 3X), dried over anhydrous $MgSO_4$, and concentrated via rotary evaporation. The residue was purified via flash column chromatography using silica gel and a 2% diethyl ether in hexanes isocratic solvent system and concentrated under reduced pressure to afford the product as a yellow oil (12.9 g, 62% yield). *Note: this compound was handled in the dark, kept cold (4 °C) and wrapped with aluminum foil when stored to prevent decomposition.*

^{1}H NMR (400 MHz, $CDCl_3$): δ 7.03 (*dt*, J = 6.8, 2.4 Hz, 2H), 6.97 (*dt*, J = 6.8, 2.4 Hz, 2H), 3.83 (*s*, 3H). ^{13}C NMR (100 MHz, $CDCl_3$): δ 159.9, 141.9, 121.4, 119.3 (*q*, J = 221 Hz), 115.2, 113.3 (q, J = 33 Hz), 56.2. ^{19}F NMR (375 MHz, $CDCl_3$): δ -69.9.

III. Synthesis of benzyl 3,3,3-trifluoro-2-((4-methoxyphenyl)imino)propanoate (**13**)

To a flame-dried 100 mL round bottom flask containing an oven-dried Teflon-coated stir bar was added tris(dibenzylideneacetone)dipalladium(0) ($Pd_2(dba)_3$) (0.315 g, 0.304 mmol, 0.05 eq),

anhydrous potassium carbonate (2.101 g, 15.2 mmol, 2.5 eq) and 2,2,2-trifluoro-*N*-(4-methoxyphenyl)acetimidoyl iodide (2.000 g, 6.08 mmol, 1.0 eq). The flask was sealed with a rubber septum, and purged with carbon monoxide using a double balloon for 3 min. The flask was charged with benzyl alcohol (1.30 mL, 12.2 mmol, 2.0 eq) and anhydrous toluene (~50 mL) and left to stir at room temperature under a carbon monoxide atmosphere in the dark for 24 h. After 24 h, the reaction was stopped mixing and filtered through a pad of Celite using a medium porosity fritted-glass funnel, rinsing with dichloromethane (3X, 25 mL). The filtrate was concentrated via rotary evaporation, and the residue was purified via flash column chromatography using silica gel and 5% EtOAc in hexanes isocratic solvent system. The product was obtained as an orange oil (1.76 g, 86% yield).

^{1}H NMR (400 MHz, $CDCl_3$): δ 7.37-7.29 (*m*, 3H), 7.17-7.15 (*m,* 2H), 6.92 (*dt*, J = 8.9, 3.2 Hz, 2H), 6.76 (*dt*, J = 8.9, 3.2 Hz, 2H), 5.21 (*s*, 2H), 3.79 (*s*, 3H). ^{13}C NMR (100 MHz, $CDCl_3$): δ 160.2, 159.4, 146.4, 146.0, 138.8, 133.6, 128.8, 128.6, 122.5 (*q*, J = 276 Hz), 122.2, 114.3, 68.2, 55.3. ^{19}F NMR (375 MHz, $CDCl_3$): δ -69.5.

IV. Synthesis of benzyl 3,3,3-trifluoro-2-((4-methoxyphenyl)amino)propanoate (**3c**)

To a flame-dried 100 mL round bottom flask containing an oven-dried Teflon-coated stir bar was added activated zinc powder (100 mesh) (3.310 g, 50.5 mmol, 30.0 eq) and 3,3,3-trifluoro-2-((4-methoxyphenyl)imino)propanoate (0.570 g, 1.68 mmol, 1.0 eq). The flask was sealed with a rubber septum and purged with Ar (g) for 3 min, charged with anhydrous dichloromethane (~30 mL) via gas-tight syringe, and placed in an ice-water bath. Glacial acetic acid (3.8 mL, 66.8 mmol, 40 eq) was added slowly to the reaction mixture via gas-tight syringe, and the reaction was stirred at 0 °C under an Ar (g) pressure for 1 h. The reaction was the filtered through a pad of Celite using a medium porosity fritted-glass funnel, rinsing with ethyl acetate (3X, 25 mL). The filtrate was transferred to a 250 mL separatory funnel, washed with sat. $NaHCO_3$ (2X, 50 mL) and sat. NaCl (1X, 50 mL). The organic layers were pooled, dried over anhydrous $MgSO_4$, and the crude product was concentrated via rotary evaporation. The residue was purified using flash column chromatography, silica gel and a 10% EtOAc in hexanes isocratic solvent system. The product was obtained as yellow oil (0.867 g, 99% yield).

^1H NMR (400 MHz, $CDCl_3$): δ 7.39-7.36 (*m*, 3H), 7.33-7.31 (*m*, 2H), 6.80 (*d*, J = 8.9 Hz, 2H), 6.70 (*d*, J = 8.9 Hz, 2H), 5.30 (*s*, 2H), 4.57-4.50 (*m*, 1H), 4.33 (*d*, J = 9.4 Hz, 1H), 3.75 (*s*, 3H). ^{13}C NMR (100 MHz, $CDCl_3$): δ 166.4, 154.0, 138.9, 128.7, 128.6, 128.3, 123.3 (*q*, J = 280 Hz), 116.2, 114.9, 68.5, 61.1 (*q*, J = 40 Hz), 55.6. ^{19}F NMR (375 MHz, $CDCl_3$): δ -72.6 (*d*, J = 6.9 Hz).

V. *Synthesis of benzyl 2-amino-3,3,3-trifluoropropanoate and benzyl 2-diazo-3,3,3-trifluoropropanoate* (**2c**)

To a 100 mL round bottom flask containing a Teflon-coated magnetic stir bar was added cerium ammonium nitrate (CAN) (3.825 g, 6.98 mmol, 4.0 eq) and benzyl 3,3,3-trifluoro-2-((4-methoxyphenyl)amino)propanoate (0.592 g, 1.74 mmol, 1.0 eq). The flask was charged with 3:1 acetonitrile/water (30 mL), capped with a yellow Caplug, placed into an ice-water bath and stirred for 1 hour. The reaction mixture was transferred to a 125 mL separatory funnel and washed with sat. $NaHCO_3$ (1X, 30 mL), and extracted with dichloromethane (3X, 40 mL). *Note: upon washing the crude reaction mixture with sat. $NaHCO_3$, a thick brown emulsion was formed.* The organic layers were collected, dried over anhydrous $MgSO_4$, and the crude product was concentrated via rotary evaporation using an ice-water bath and low vacuum. The residue was purified via flash column chromatography using silica gel and a 20% EtOAc in hexanes isocratic solvent system, monitoring using TLC (20% EtOAc/hexanes) and visualizing using a ninhydrin stain. The purified product was obtained as a yellow-orange liquid (88.5% yield) and used in the next step without further characterization. To a 50 mL round bottom flask containing an oven-dried Teflon-coated magnetic stir bar and benzyl 2-amino-3,3,3-trifluoropropanoate (1.306 g, 5.60 mmol, 1.0 eq) was added isoamyl nitrite (1.13 mL, 8.40 mmol, 1.5 eq) and glacial acetic acid (32.1 µL, 0.560 mmol, 0.1 eq). The reaction flask was equipped with a condenser that was sealed with a rubber septum, purged with Ar (g) for 3 min, and charged with anhydrous chloroform (15 mL) via gas-tight syringe. The reaction flask was stirred and heated to a reflux (80 °C) for 45 min under an Ar (g) pressure. The reaction mixture was transferred to a separatory funnel, washed with sat. $NaHCO_3$ (1X, 20 mL), and extracted with dichloromethane (3X, 20 mL). The organic layers were pooled, the solvent was removed via rotary evaporation using an ice-water bath and low vacuum. The crude product was purified via flash column chromatography using silica gel and a 5% diethyl

ether in hexanes solvent system. The product was concentrated via rotary evaporation, using an ice-water bath and low vacuum, and was obtained as a faint yellow liquid (289 mg, 68% yield).

^{1}H NMR (400 MHz, $CDCl_3$): *δ* 7.40-7.33 (*m*, 5H), 5.28 (*s*, 2H). ^{13}C NMR (125 MHz, $CDCl_3$): *δ* 160.7, 134.8, 129.3, 128.7, 128.3, 126.6 (*q*, *J* = 268 Hz), 67.4 (Note: the *α*-diazo carbon was not detected). ^{19}F NMR (375 MHz, $CDCl_3$): *δ* -57.5.

Cyclohexyl 2-diazo-3,3,3-trifluoropropanoate (2b)

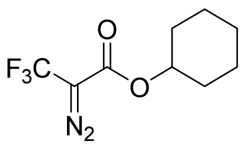

Following the standard procedure above, diazo propanoate ***2*b** was isolated as yellow oil, 21 mg, 18% yield over five steps. ^{1}H NMR (400 MHz, $CDCl_3$) δ 4.96 (hept, *J* = 3.8 Hz, 1H), 1.95 – 1.80 (m, 2H), 1.80 – 1.66 (m, 2H), 1.64 – 1.46 (m, 3H), 1.46 – 1.19 (m, 3H). ^{13}C NMR (101 MHz, $CDCl_3$) δ 166.6, 123.7 (q, *J* = 282.2 Hz), 75.1, 57.2 (q, *J* = 30.4 Hz), 31.2, 31.0, 25.1, 23.3, 23.2. ^{19}F NMR (376 MHz, $CDCl_3$) δ -57.6.

Naphthalen-1-ylmethyl 2-diazo-3,3,3-trifluoropropanoate (2d)

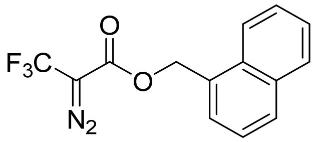

Following the standard procedure above, diazo propanoate **2d** was isolated as yellow oil, 16 mg, 16% yield over five steps. ^{1}H NMR (400 MHz, $CDCl_3$) δ 8.00 (dd, *J* = 8.3, 1.4 Hz, 1H), 7.94 – 7.86 (m, 2H), 7.61 – 7.51 (m, 3H), 7.47 (dd, *J* = 8.2, 7.0 Hz, 1H), 5.75 (s, 2H). ^{13}C NMR (126 MHz, $CDCl_3$) δ 134.5, 132.3, 131.1, 130.5, 129.5, 128.6, 127.5, 126.8, 126.0, 124.0, 66.6. Trifluoromethyl, diazo and carbonyl carbon not detected. ^{19}F NMR (376 MHz, C_6D_6) δ -57.53.

2-Methylbenzyl 2-diazo-3,3,3-trifluoropropanoate (2e)

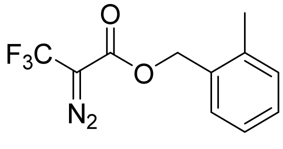

Following the standard procedure above, diazo propanoate **2e** was isolated as yellow solid, 20 mg, 14% yield over five steps. ^{1}H NMR (400 MHz, $CDCl_3$): δ 7.35 – 7.17 (m, 4H), 5.31 (s, 2H), 2.36 (s, 3H). ^{13}C NMR (101 MHz, $CDCl_3$): δ 160.2, 132.8, 130.4, 129.4, 128.9, 126.1, 66.0, 18.8. Trifluoromethyl and α-diazo carbon not detected. ^{19}F NMR (376 MHz, $CDCl_3$) δ -57.5.

2,4-Dimethylbenzyl 2-diazo-3,3,3-trifluoropropanoate (2f)

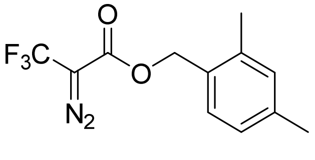

Following the standard procedure above, diazo propanoate **2f** was isolated as yellow solid, 56 mg, 18% yield over five steps. ^{1}H NMR (400 MHz, $CDCl_3$) δ 7.13 (s, 1H), 7.12 – 7.06 (m, 2H), 5.27 (s, 2H), 2.32 (s, 3H), 2.31 (s, 3H). ^{13}C NMR (126 MHz, $CDCl_3$) δ 161.5, 139.7, 136.4, 134.8, 132.7 (q, J = 157.2 Hz), 131.2, 131.1, 130.4, 127.5, 66.9, 21.6, 19.1. ^{19}F NMR (376 MHz, C_6D_6) δ -57.5.

2,5-Dimethylbenzyl 2-diazo-3,3,3-trifluoropropanoate (2g)

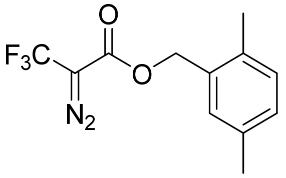

Following the standard procedure above, diazo propanoate **2g** was isolated as yellow solid, 97 mg, 16% yield over five steps. ^{1}H NMR (400 MHz, $CDCl_3$) δ 7.13 (s, 1H), 7.11 – 7.05 (m, 2H), 5.27 (s, 2H), 2.32 (s, 3H), 2.31 (s, 3H). ^{13}C NMR (126 MHz, $CDCl_3$) δ 161.5, 139.7, 136.4, 134.8, 132.7 (q, J = 157.2 Hz), 131.2, 131.1, 130.4, 127.5, 66.9, 21.6, 19.1. ^{19}F NMR (376 MHz, C_6D_6) δ -57.5.

2,4,6-Trimethylbenzyl 2-diazo-3,3,3-trifluoropropanoate (2h)

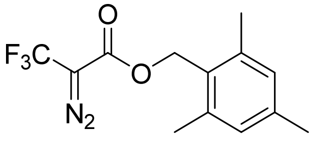

Following the standard procedure above, diazo propanoate **2h** was isolated as yellow solid, 29 mg, 15% yield over five steps. ^{1}H NMR (400 MHz, $CDCl_3$): δ 6.88 (s, 2H), 5.36 (s, 2H), 2.36 (s, 6H), 2.28 (s, 3H). ^{13}C NMR (101 MHz, $CDCl_3$): δ 138.9, 129.1, 62.5, 21.0, 19.4. Trifluoromethyl and α-diazo carbon not detected. ^{19}F NMR (376 MHz, $CDCl_3$) δ -57.6.

Cyclohexyl 3,3,3-trifluoro-2-((4-methoxyphenyl)amino)propanoate (3b)

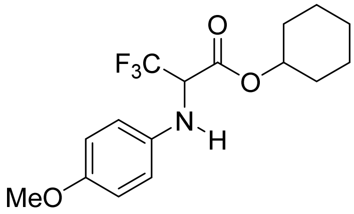

Following the standard procedure above, trifluoropropanoate **3b** was isolated as yellow oil, 412 mg, 36% yield over four steps. ^{1}H NMR (400 MHz, $CDCl_3$) δ 6.84 – 6.77 (m, 2H), 6.74 – 6.68 (m, 2H), 4.92 (hepta, J = 3.8 Hz, 1H), 4.44 (dq, J = 8.6, 7.0 Hz, 1H), 4.34 (d, J = 8.6 Hz, 1H), 3.75 (s, 3H), 1.86 – 1.80 (m, 2H), 1.77 – 1.67 1.71 (m, 2H), 1.57 – 1.45 (m, 3H), 1.44 – 1.25 (m, 3H). ^{13}C NMR (101 MHz, $CDCl_3$) δ 165.8, 153.8, 139.2, 123.4 (q, J = 283.2 Hz), 116.0, 114.8, 75.7, 61.1 (q, J = 30.8 Hz), 55.6, 31.2, 30.9, 25.1, 23.3, 23.2. ^{19}F NMR (376 MHz, $CDCl_3$) δ -72.73 (d, J = 6.9 Hz). GC-MS *m/z* (% relative intensity): 77.1 (12.9), 83.1 (100.0), 91.1 (10.9), 134.1 (13.8), 204.1 (52.3), 331.4 (5.2).

Benzyl 3,3,3-trifluoro-2-((4-methoxyphenyl)amino)propanoate (3c)

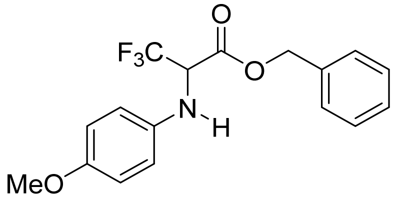

Following the standard procedure above, trifluoropropanoate **3c** was isolated as yellow oil, 698 mg, 38% yield over four steps. ^{1}H NMR (400 MHz, $CDCl_3$) δ 7.47 – 7.26 (m, 5H), 6.85 – 6.75 (m, 2H), 6.74 – 6.65 (m, 2H), 5.26 (s, 2H), 4.53 (dq, J = 9.4, 7.0 Hz, 1H), 4.32 (d, J = 9.4 Hz, 1H), 3.76 (s, 3H). ^{13}C NMR (101 MHz, $CDCl_3$) δ 166.5, 154.0, 138.9, 134.3, 128.7, 128.7, 128.3, 123.3 (q, J = 283.4 Hz), 116.2, 114.9, 68.5, 61.1 (q, J = 31.2 Hz), 55.6. ^{19}F NMR (376 MHz, $CDCl_3$) δ -72.6 (d, J = 7.1 Hz). GC-MS *m/z* (% relative intensity): 107 (5), 134 (22.5), 157 (17.1), 184 (11.7), 204 (100), 205 (11), 339 (49), 340 (10).

Naphthalen-1-ylmethyl 3,3,3-trifluoro-2-((4-methoxyphenyl)amino)propanoate (3d)

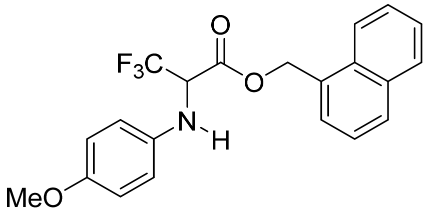

Following the standard procedure above, trifluoropropanoate **3d** was isolated as yellow solid, 397 mg, 31% yield over four steps. ^{1}H NMR (400 MHz, $CDCl_3$) δ 7.96 – 7.88 (m, 3H), 7.59 – 7.51 (m, 3H), 7.46 (dd, *J* = 8.2, 7.0 Hz, 1H), 6.79 – 6.73 (m, 2H), 6.68 – 6.62 (m, 2H), 5.71 (s, 2H), 4.54 (q, *J* = 7.0 Hz, 1H), 3.75 (s, 3H). ^{13}C NMR (126 MHz, $CDCl_3$) δ 167.40, 154.76, 139.57, 134.50, 132.26, 130.72, 130.62, 129.56, 128.84, 127.60, 126.90, 125.96, 124.09 (q, *J* = 283.1 Hz), 124.02, 117.03, 115.65, 67.71, 61.90 (q, *J* = 31.0 Hz), 56.36. ^{19}F NMR (376 MHz, C_6D_6) δ -72.58 (d, *J* = 6.9 Hz). GC-MS *m/z* (% relative intensity): 77.1 (8.8), 92.1 (7.9), 115.0 (24.9), 141.1 (100.0), 204.1 (64.9), 389.2 (9.3).

2-Methylbenzyl 3,3,3-trifluoro-2-((4-methoxyphenyl)amino)propanoate (3e)

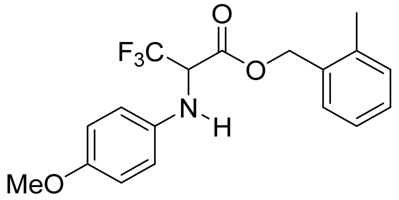

Following the standard procedure above, trifluoropropanoate **3e** was isolated as pale-yellow oil, 256 mg, 32% yield over four steps. ^{1}H NMR (400 MHz, $CDCl_3$) δ 7.31 – 7.24 (m, 2H), 7.23 – 7.16 (m, 2H), 6.81 – 6.75 (m, 2H), 6.73 – 6.65 (m, 2H), 5.26 (d, *J* = 1.4 Hz, 2H), 4.50 (q, *J* = 7.0 Hz, 1H), 3.75 (s, 3H), 2.31 (s, 3H). ^{13}C NMR (101 MHz, $CDCl_3$) δ 166.5, 154.0, 138.9, 137.3, 132.2, 130.5, 129.7, 129.1, 126.1, 116.2, 114.9, 67.1, 61.0 (*q*, *J* = 31 Hz), 55.6, 18.6. Trifluoromethyl carbon not detected. ^{19}F NMR (376 MHz, $CDCl_3$) δ -72.7 (d, *J* = 7.1 Hz). GC-MS *m/z* (% relative intensity): 79.1 (12.8), 92.1 (10.0), 105.1 (100.0), 134.1 (19.4), 157.1 (11.5), 204.1 (66.4), 353.2 (11.2).

2,4-Dimethylbenzyl 3,3,3-trifluoro-2-((4-methoxyphenyl)amino)propanoate (3f)

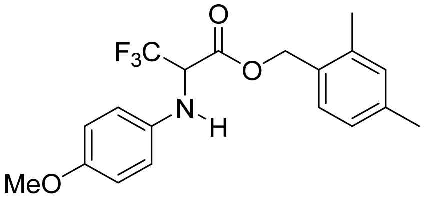

Following the standard procedure above, trifluoropropanoate **3f** was isolated as yellow oil, 612 mg, 35% yield over four steps. ^{1}H NMR (400 MHz, $CDCl_3$) δ 7.12 – 7.06 (m, 3H), 6.82 – 6.76 (m, 2H), 6.72 – 6.64 (m, 2H), 5.23 (s, 2H), 4.51 (q, *J* = 7.1 Hz, 1H), 3.75 (s, 3H), 2.31 (s, 3H), 2.26 (s, 3H). ^{13}C NMR (126 MHz, $CDCl_3$) δ 167.3, 154.8, 139.7, 136.4, 134.9, 132.8, 131.2, 131.2, 130.6, 117.0, 115.7, 67.9, 61.9 (q, *J* = 31.2 Hz), 56.4, 21.6, 18.9. Trifluoromethyl carbon not detected. ^{19}F NMR (376 MHz, $CDCl_3$) δ -72.68 (d, *J* = 7.0 Hz). GC-MS *m/z* (% relative intensity): 77.1 (12.9), 91.1 (15.9), 119.1 (100.0), 134.1 (12.8), 204.1 (54.3), 367.2 (5.1).

2,5-Dimethylbenzyl 3,3,3-trifluoro-2-((4-methoxyphenyl)amino)propanoate (3g)

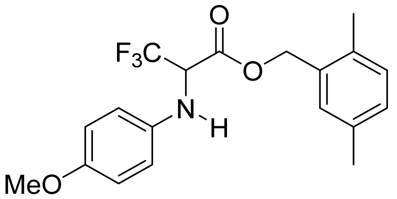

Following the standard procedure above, trifluoropropanoate **3g** was isolated as yellow oil, 430 mg, 29% yield over four steps. ^{1}H NMR (400 MHz, $CDCl_3$) δ 7.09 (s, 3H), 6.79 (d, *J* = 8.9 Hz, 2H), 6.68 (d, *J* = 9.0 Hz, 2H), 5.23 (s, 2H), 4.56 – 4.46 (m, 1H), 4.30 (d, *J* = 9.0 Hz, 1H), 3.75 (s, 3H), 2.31 (s, 3H), 2.26 (s, 3H). ^{13}C NMR (126 MHz, $CDCl_3$) δ 167.27, 154.75, 139.66, 136.42, 134.84, 132.77, 131.19, 131.18, 130.56, 116.99, 115.66, 67.93, 61.98, 61.73, 56.39, 21.56, 18.93. GC-MS *m/z* (% relative intensity): 77.1 (12.9), 91.1 (15.9), 119.1 (100.0), 134.1 (12.8), 204.1 (54.3), 367.2 (5.1).

2,4,6-trimethylbenzyl 3,3,3-trifluoro-2-((4-methoxyphenyl)amino)propanoate (3h)

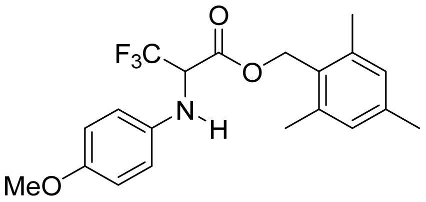

Following the standard procedure above, trifluoropropanoate **3h** was isolated as pale-yellow oil, 194 mg, 25% yield over four steps. ^{1}H NMR (400 MHz, $CDCl_3$): δ 6.89 (s, 2H), 6.79 (d, J = 2.3 Hz, 1H), 6.77 (d, J = 2.4 Hz, 1H), 6.68 (d, J = 2.4 Hz, 1H), 6.66 (d, J = 2.3 Hz, 1H), 5.38 – 5.22 (m, 2H), 4.47 (q, J = 7.0 Hz, 1H), 3.75 (s, 3H), 2.30 (s, 6H), 2.29 (s, 3H). ^{13}C NMR (101 MHz, $CDCl_3$): δ 166.8, 153.9, 139.1, 138.9, 138.4, 129.1, 127.5, 116.1, 114.9, 63.4, 61.0 (*q*, J = 31 Hz), 55.61, 21.0, 19.2. Trifluoromethyl carbon not detected. ^{19}F NMR (376 MHz, $CDCl_3$): δ -72.8 (d, J = 7.0 Hz). GC-MS *m/z* (% relative intensity): 77.1 (9.2), 91.1 (10.3), 133.1 (100.0), 204.1 (48.6), 381.2 (5.1).

Synthesis of racemic analytical standards

The following general procedure was followed to synthesize racemic analytical standards **3a, 4b-k, 5a, 5c-h** and used in the preparation of calibration curves for SFC analysis. To a flame-dried 25-mL round bottom flask containing an oven-dried Teflon-coated magnetic stir bar was added the desired aryl amine (0.310 mmol, 3.0 eq) and $Rh_2(OAc)_4$ (0.005 mmol, 0.05 eq). The flask was purged with Ar (g) for 3 minutes, charged with anhydrous toluene (5 mL) via gas-tight syringe, and placed in an ice-water bath with stirring under Ar (g) pressure. In a separate flame-dried 5-mL flask, either ethyl or benzyl 2-diazo-3,3,3-trifluoropropanoate (0.102 mmol, 1.0 eq) was added and diluted with anhydrous toluene (1 mL). The alkyl 2-diazo-3,3,3-trifluoropropanoate solution was added drop wise to the reaction flask for 1 hour using an automated syringe pump. Upon complete addition of the diazo solution, the reaction mixture was stirred for an additional 5 min, then the flask was equipped with a condenser and heated to a reflux (80 °C) while stirring under Ar (g) pressure for 18 h. The reaction was monitored via TLC, visualizing with a ninhydrin stain. After 18 hours, the reaction was stopped stirring, the solvent was removed via rotary evaporation and the residue was purified via flash column chromatography using silica gel and a 20% EtOAc in hexanes isocratic solvent system. The purified compounds were characterized using ^{1}H, ^{13}C and ^{19}F NMR and GC-MS.

Ethyl 3,3,3-trifluoro-2-((4-methoxyphenyl)amino)propanoate (3a)

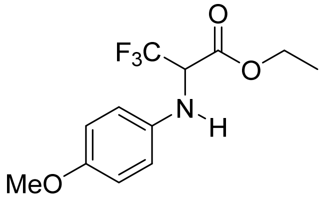

Following the standard procedure above, trifluoropropanoate **3a** was isolated as yellow solid, 76 mg, 53% yield.^{1}H NMR (400 MHz, $CDCl_3$) δ 6.85 – 6.78 (m, 2H), 6.76 – 6.66 (m, 2H), 4.46 (dq, J = 9.0, 6.9 Hz, 1H), 4.35 (d, J = 9.0, 1H), 4.30 (m, 2H), 3.75 (s, 3H), 1.31 (t, J = 7.1 Hz, 3H). ^{13}C NMR (101 MHz, $CDCl_3$) δ 166.5, 153.9, 139.1, 123.3 (q, J = 283.1 Hz), 116.1, 114.9, 62.9, 60.9 (q, J = 31.1 Hz), 55.6, 55.6, 13.9. ^{19}F NMR (376 MHz, C_6D_6) δ -72.85 (d, J = 6.9 Hz). GC-MS *m/z* (% relative intensity): 134 (26.6), 157 (12.6), 184 (8.9), 204 (100), 205 (11), 277 (56.0), 278 (8.0).

Benzyl 3,3,3-trifluoro-2-(*p*-tolylamino)propanoate (4b)

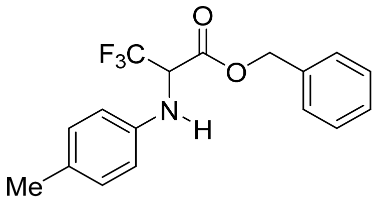

Following the standard procedure above, trifluoropropanoate **4b** was isolated as pale-yellow crystalline solid, 16 mg, 48% yield. ^{1}H NMR (400 MHz, CDCl3) δ 7.40 – 7.35 (m, 3H), 7.34 – 7.30 (m, 2H), 7.04 (d, *J* = 8.4 Hz, 2H), 6.64 (d, *J* = 8.4 Hz, 2H), 5.26 (s, 2H), 4.61 (q, *J* = 7.0 Hz, 1H), 2.26 (s, 3H). ^{13}C NMR (126 MHz, CDCl3) δ 167.1, 143.5, 135.1, 130.7, 130.3, 129.5, 129.5, 129.1, 124.1 (q, *J* = 283.3 Hz), 115.3, 69.3, 61.0 (q, *J* = 31.5 Hz), 21.2. ^{19}F NMR (376 MHz, CDCl3) δ -72.6 (d, *J* = 6.9 Hz). GC-MS *m/z* (% relative intensity): 118.1 (27.7), 148.0 (6.2), 188 (100.0), 189 (10.4), 323.1 (30.5), 324.1 (6).

Benzyl 3,3,3-trifluoro-2-(*m*-tolylamino)propanoate (4c)

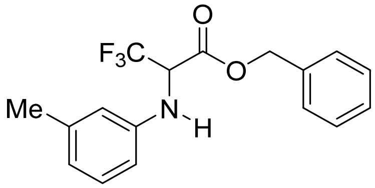

Following the standard procedure above, trifluoropropanoate **4c** was isolated as pale-yellow oil, 16 mg, 48% yield. ^{1}H NMR (400 MHz, CDCl3) δ 7.36 (m, 3H), 7.32 (m, 2H), 7.10 (dd, *J* = 7.6, 7.6 Hz, 1H), 6.68 (d, *J* = 7.6 Hz, 1H) 6.53 (*s,* 1H), 6.52 (d, *J* = 8.3 Hz, 1H), 5.26 (s, 2H), 4.64 (q, *J* = 6.9 Hz, 1H), 2.28 (s, 3H). ^{13}C NMR (126 MHz, CDCl3) δ 145.8, 140.2, 135.1, 130.1, 129.5, 129.5, 129.1, 121.8, 115.8, 112.0, 69.3, 60.5 (q, *J* = 31.3 Hz), 22.3. Trifluoromethyl carbon not detected. ^{19}F NMR (376 MHz, CDCl3) δ -72.6 (d, *J* = 6.9 Hz). GC-MS *m/z* (% relative intensity): 118.1 (22.6), 148.0 (5.6), 188 (100.0), 189 (10.5), 323.1 (27.7), 324.1 (5.4).

Benzyl 3,3,3-trifluoro-2-(*o*-tolylamino)propanoate (4d)

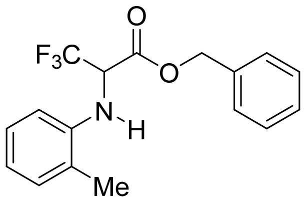

Following the standard procedure above, trifluoropropanoate **4d** was isolated as pale-yellow oil, 20 mg, 53% yield. ^{1}H NMR (400 MHz, $CDCl_3$) δ 7.41 – 7.31 (m, 5H), 7.12 (d, *J* = 7.4 Hz, 1H) 7.11 (dd, *J* = 7.4, 7.4 Hz, 1H), 6.80 (dd, *J* = 8.0, 7.4 Hz, 1H), 6.63 (d, *J* = 8.0 Hz, 1H), 5.28 (s, 2H), 4.69 (q, *J* = 4.9 Hz, 1H), 4.52 (br, 1H), 2.25 (s, 3H). ^{13}C NMR (126 MHz, $CDCl_3$) δ 167.2, 144.0, 135.1, 131.5, 129.6, 129.5, 129.1, 127.9, 124.6, 120.6, 112.2, 69.4, 60.6 (q, *J* = 31.6 Hz), 18.05. Trifluoromethyl carbon not detected. ^{19}F NMR (376 MHz, C_6D_6) δ -72.5 (d, *J* = 7.0 Hz). GC-MS *m/z* (% relative intensity): 118.1 (22.6), 148.0 (5.6), 188 (100.0), 189 (10.5), 323.1 (27.7), 324.1 (5.4).

Benzyl 2-((4-chlorophenyl)amino)-3,3,3-trifluoropropanoate (4e)

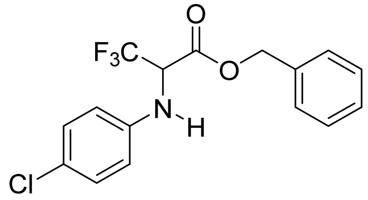

Following the standard procedure above, trifluoropropanoate **4e** was isolated as yellow crystalline solid, 20 mg, 57% yield. ^{1}H NMR (400 MHz, $CDCl_3$) δ 7.43 – 7.36 (m, 3H), 7.36 – 7.30 (m, 2H), 7.16 (d, *J* = 8.4 Hz, 2H), 6.63 (d, *J* = 8.4 Hz, 2H), 5.28 (s, 2H), 4.64 – 4.54 (m, 1H), 4.57 (br, 1H). ^{13}C NMR (126 MHz, $CDCl_3$) δ 166.6, 144.3, 134.9, 130.1, 129.6, 129.5, 129.1, 125.7, 123.9 (q, *J* = 283.8 Hz), 116.1, 69.5, 60.5 (q, *J* = 31.6 Hz). ^{19}F NMR (376 MHz, $CDCl_3$) δ -72.5 (d, *J* = 5.5 Hz). GC-MS *m/z* (% relative intensity): 111.0 (18.7), 113.0 (6.1), 138.0 (40.2), 140.0 (13.2), 188 (8.4), 207.9 (100.0), 209.0 (9.4), 210.0 (32.8), 343.0 (35.7), 344.0 (6.7) 345.0 (11.8).

Benzyl 2-((4-bromophenyl)amino)-3,3,3-trifluoropropanoate (4f)

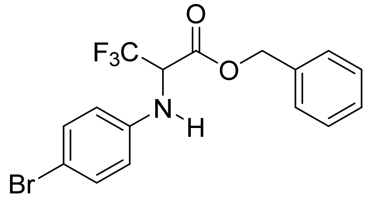

Following the standard procedure above, trifluoropropanoate **4f** was isolated as pale-yellow solid, 16 mg, 41% yield. ^{1}H NMR (400 MHz, $CDCl_3$) δ 7.42 – 7.27 (m, 7H), 6.59 (d, *J* = 8.3 Hz, 2H), 5.28 (s, 2H), 4.58 (m, 2H). ^{13}C NMR (126 MHz, $CDCl_3$) δ 172.6, 144.8, 134.9, 133.0, 129.6, 129.5, 129.1, 116.5, 112.8, 69.5, 60.3 (q, *J* = 31.5 Hz). Trifluoromethyl carbon not detected. ^{19}F NMR

(376 MHz, $CDCl_3$) δ -72.46 (d, J = 5.0 Hz). GC-MS *m/z* (% relative intensity): 104.0 (14.1), 152.9 (16.9), 181.8 (24.8), 183.8 (23.7), 190.9 (18.5), 194.0 (12.4), 251.8 (100.0), 252.8 (10.5), 253.8 (99.5), 254.8 (9.2), 294.0 (10.9), 295.0 (51.5), 296.0 (28.1), 322.0 (30.7), 324.0 (10.6), 343.0 (11.2), 368.0 (65.0), 371.0 (80.7), 372.0 (22.4), 386.8 (38.7), 388.8 (37.6).

Benzyl 2-((3-chloro-4-fluorophenyl)amino)-3,3,3-trifluoropropanoate (4g)

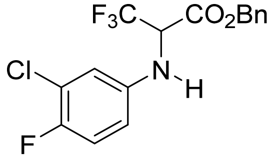

Following the standard procedure above, trifluoropropanoate **4g** was isolated as colorless oil, 29 mg, 79% yield. ^{1}H NMR (400 MHz, $CDCl_3$) δ 7.38 (m, 3H), 7.34 (m, 2H), 6.98 (dd, J = 8.8, 8.8 Hz, 1H), 6.73 (dd, J = 5.9, 3.0 Hz, 1H), 6.55 (ddd, J = 8.9, 3.3, 3.0 Hz, 1H), 5.28 (s, 2H), 4.53 (br, 1H), 4.52 (q, J = 3.8 Hz, 1H). ^{13}C NMR (101 MHz, $CDCl_3$) δ 165.7, 153.7, 151.3, 141.8 (d, J = 2.8 Hz), 134.1, 128.6 (d, J = 39.8 Hz), 124.4, 121.6 (d, J = 4.9 Hz), 121.4, 117.1 (d, J = 23.2 Hz), 115.9, 113.7 (d, J = 2.5 Hz), 68.9, 59.9 (q, J = 32.0 Hz). Trifluoromethyl carbon not detected. ^{19}F NMR (376 MHz, $CDCl_3$) δ -72.5 (d, J = 6.4 Hz), -127.4 (ddd, J = 8.9, 5.9, 3.6 Hz). GC-MS *m/z* (% relative intensity): 129.0 (15.3), 131.0 (5.4), 156.0 (45.5), 158.0 (15.1), 225.9 (100.0), 226.9 (9.4), 227.9 (32.4), 361.0 (27.7), 362.0 (5.1), 363.0 (9.2).

Benzyl 3,3,3-trifluoro-2-((4-isopropylphenyl)amino)propanoate (4h)

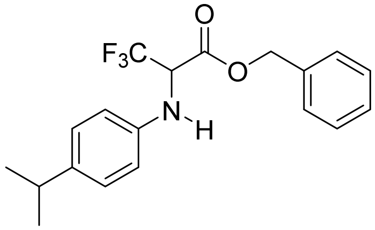

Following the standard procedure above, trifluoropropanoate **4h** was isolated as orange oil, 20 mg, 58% yield. ^{1}H NMR (400 MHz, $CDCl_3$) δ 7.39 – 7.35 (m, 3H), 7.34 – 7.30 (m, 2H), 7.08 (d, J = 8.5 Hz, 2H), 6.66 (d, J = 8.5 Hz, 2H), 5.26 (s, 2H), 4.61 (q, J = 7.0 Hz, 1H), 2.83 (hept, J = 7.0 Hz, 1H), 1.21 (d, J = 7.0 Hz, 6H). ^{13}C NMR (126 MHz, $CDCl_3$) δ 167.1, 143.7, 141.5, 135.1, 129.5, 129.5, 129.1, 128.1, 124.1 (q, J = 283.6 Hz), 115.1, 69.3, 60.8 (q, J = 31.5 Hz), 34.0, 24.9. ^{19}F NMR (376 MHz, $CDCl_3$) δ -72.7 (d, J = 6.9 Hz). GC-MS *m/z* (% relative intensity): 103.0

(5.3), 104.0 (8.9), 154.0 (9.6), 201.0 (7.3), 216.0 (100.0), 217.0 (12.6), 336.1 (5.1), 351.1 (36.6), 352.1 (7.9).

Benzyl 2-((4-(*tert*-butyl)phenyl)amino)-3,3,3-trifluoropropanoate (4i)

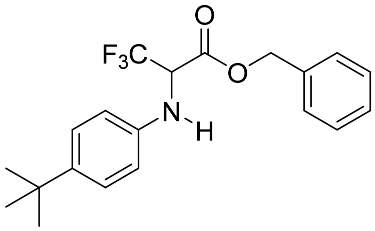

Following the standard procedure above, trifluoropropanoate **4i** was isolated as yellow-orange oil, 14 mg, 39% yield. ^{1}H NMR (400 MHz, $CDCl_3$) δ 7.40 – 7.36 (m, 3H), 7.36 – 7.31 (m, 2H), 7.26 (d, *J* = 8.7 Hz, 2H), 6.69 (d, *J* = 8.7 Hz, 1H), 5.27 (s, 2H), 4.64 (q, *J* = 7.2 Hz, 1H), 4.52 (br, 1H), 1.30 (s, 9H). ^{13}C NMR (126 MHz, $CDCl_3$) δ 167.1, 143.8, 143.4, 135.1, 129.5, 129.5, 129.1, 127.1, 124.1 (q, *J* = 283.4 Hz), 114.8, 69.3, 60.8 (q, *J* = 31.4 Hz), 34.8, 32.2. ^{19}F NMR (376 MHz, $CDCl_3$) δ -72.6 (d, *J* = 7.0 Hz). GC-MS *m/z* (% relative intensity): 117.1 (6.1), 118.1 (9.0), 200.0 (8.01), 214.0 (5.3), 215.0 (17.7), 230.1 (100.0), 231.0 (14.2), 350.1 (36.2), 351.1 (8.1), 365.1 (36.5), 366.1 (9.1).

Benzyl 3,3,3-trifluoro-2-(naphthalen-2-ylamino)propanoate (4j)

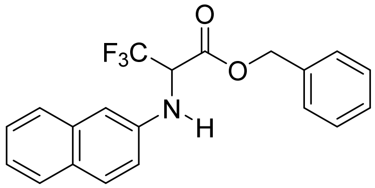

Following the standard procedure above, trifluoropropanoate **4j** was isolated as a beige crystalline solid, 30 mg, 84% yield. ^{1}H NMR (400 MHz, $CDCl_3$) δ 7.75 – 7.69 (m, 2H), 7.63 (dd, *J* = 8.1, 1.1 Hz, 1H), 7.42 (ddd, *J* = 8.2, 6.8, 1.3 Hz, 1H), 7.39 – 7.28 (m, 6H), 7.00 (dd, *J* = 8.8, 2.5 Hz, 1H), 6.94 (d, *J* = 2.4 Hz, 1H), 5.29 (s, 2H), 4.83 (dq, *J* = 9.1, 6.7 Hz, 1H), 4.75 (d, *J* = 9.1 Hz, 1H). ^{13}C NMR (126 MHz, $CDCl_3$) δ 229.4, 166.9, 143.4, 135.3, 135.1, 130.3, 129.6, 129.5, 129.4, 129.1, 128.4, 127.4, 127.1, 124.1, 118.6, 107.9, 69.4, 60.4 (q, *J* = 31.7 Hz). Trifluoromethyl carbon not detected. ^{19}F NMR (376 MHz, $CDCl_3$) δ -72.31 (d, *J* = 6.8 Hz). GC-MS *m/z* (% relative intensity): 126.0 (8.1), 127.0 (46.7), 128.0 (10.1), 154.0 (15.5), 155.0 (6.1), 177.0 (11.5), 204.0 (6.4), 224.0 (100.0), 225.0 (14.0), 359.1 (52.0), 360.1 (11.9).

Benzyl 2-(benzo[*d*][1,3]dioxol-5-ylamino)-3,3,3-trifluoropropanoate (4k)

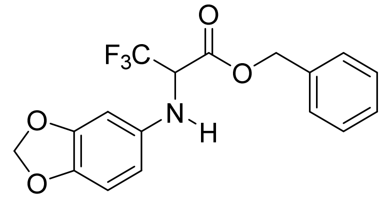

Following the standard procedure above, trifluoropropanoate **4k** was isolated as yellow oil, 9 mg, 24% yield. ^{1}H NMR (400 MHz, $CDCl_3$) δ 7.42 – 7.29 (m, 5H), 6.65 (d, *J* = 8.3 Hz, 1H), 6.33 (d, *J* = 2.3 Hz, 1H), 6.15 (d, *J* = 8.3 Hz, 1H), 5.89 (s, 2H), 5.26 (s, 2H), 4.54 – 4.44 (m, 1H), 4.33 (d, *J* = 9.4 Hz, 1H). ^{13}C NMR (101 MHz, $CDCl_3$) δ 164.5, 146.7, 139.9, 138.5, 132.5, 126.9, 126.9, 126.5, 121.3 (q, *J* = 283.4 Hz), 106.7, 104.9, 99.2, 96.2, 66.7, 59.3 (q, *J* = 31.4 Hz). ^{19}F NMR (376 MHz, $CDCl_3$) δ -72.6 (d, *J* = 7.2 Hz). GC-MS *m/z* (% relative intensity): 121.0 (9.2), 141.0 (7.0), 148.0 (8.8), 168.0 (45.2), 171.0 (5.2), 218.0 (100.0), 219.0 (10.4), 353.0 (67.4), 354.0 (13.1).

Benzyl 2-((4-acetylphenyl)amino)-3,3,3-trifluoropropanoate (4l)

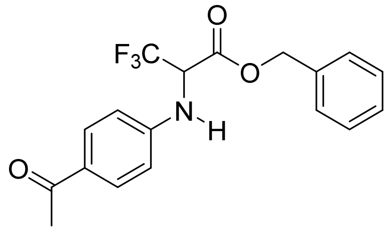

Following the standard procedure above, trifluoropropanoate **4l** was isolated as yellow oil, 6 mg, 52% yield. ^{1}H NMR (400 MHz, $CDCl_3$) δ 7.86 (d, *J* = 8.7 Hz, 2H), 7.43 – 7.31 (m, 5H), 6.70 (d, *J* = 8.7 Hz, 2H), 5.30 (s, 2H), 5.00 (d, *J* = 8.6 Hz, 1H), 4.75 (dq, *J* = 8.6, 6.7 Hz, 1H), 2.52 (s, 3H). ^{13}C NMR (126 MHz, $CDCl_3$) δ 197.1, 168.3, 149.6, 131.4, 130.1, 129.7, 129.5, 129.2, 113.6, 69.8, 26.9. Trifluoromethyl carbon not detected. ^{19}F NMR (376 MHz, $CDCl_3$) δ -72.3 (d, *J* = 6.6 Hz). GC-MS *m/z* (% relative intensity): 76.1 (6.2), 91.1 (100.0), 173.1 (7.7), 216.1 (45.3), 351.2 (4.8).

Benzyl 2-((4-cyanophenyl)amino)-3,3,3-trifluoropropanoate (4m)

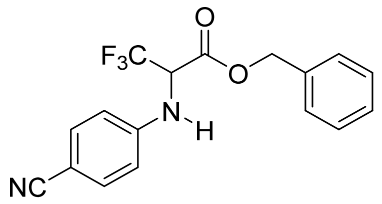

Following the standard procedure above, trifluoropropanoate **4m** was isolated as yellow oil, 7 mg, 62% yield. ^{1}H NMR (400 MHz, $CDCl_3$) δ 7.49 (d, *J* = 8.7 Hz, 2H), 7.41 – 7.33 (m, 5H), 6.70 (d, *J* = 8.7 Hz, 2H), 5.31 (s, 2H), 5.03 (d, *J* = 8.5 Hz, 1H), 4.75 – 4.63 (m, 1H). ^{13}C NMR (126 MHz, $CDCl_3$) δ 167.1, 149.0, 134.6, 129.8, 129.6, 129.3, 114.4, 103.2, 69.9, 59.0 (q, *J* = 32.3 Hz). Trifluoromethyl carbon not detected. ^{19}F NMR (376 MHz, $CDCl_3$) δ -72.2 (d, *J* = 6.8 Hz). GC-MS *m/z* (% relative intensity): 77.1 (6.7), 91.1 (14.9), 102.0 (9.2), 119.1 (100.0), 129.1 (14.7), 199.1 (12.1), 362.2 (1.7).

Benzyl 3,3,3-trifluoro-2-((3-(trifluoromethyl)phenyl)amino)propanoate (4n)

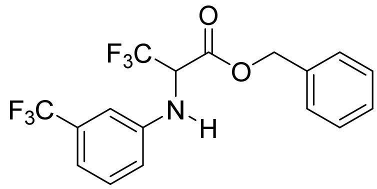

Following the standard procedure above, trifluoropropanoate **4n** was isolated as yellow oil, 8 mg, 61% yield. ^{1}H NMR (400 MHz, $CDCl_3$) δ 7.43 – 7.28 (m, 6H), 7.10 (d, *J* = 7.7 Hz, 1H), 6.91 (s, 1H), 6.86 (dd, *J* = 8.3, 2.5 Hz, 1H), 5.29 (s, 2H), 4.77 (d, *J* = 8.7 Hz, 1H), 4.70 – 4.63 (m, 1H). ^{13}C NMR (126 MHz, $CDCl_3$) δ 166.4, 146.0, 134.8, 130.8, 129.7, 129.5, 129.2, 117.8, 117.4, 111.2, 69.7, 59.9 (q, *J* = 32.0 Hz). Trifluoromethyl carbon not detected. ^{19}F NMR (376 MHz, $CDCl_3$) δ -63.1, -72.5 (d, *J* = 6.5 Hz). GC-MS *m/z* (% relative intensity): 77.1 (5.8), 91.1 (14.4), 103.1 (4.7), 119.1 (100.0), 145.0 (7.5), 172.1 (14.6), 242.0 (29.5), 405.1 (2.3).

2,5-Dimethylbenzyl 3,3,3-trifluoro-2-(phenylamino)propanoate (5a)

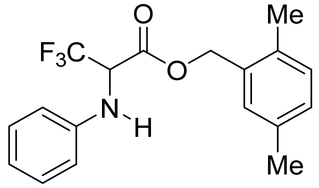

Following the standard procedure above, trifluoropropanoate **5a** was isolated as yellow oil, 12 mg, 81% yield. ^{1}H NMR (400 MHz, $CDCl_3$): δ 7.26 – 7.18 (m, 2H), 7.12 – 7.08 (m, 3H), 6.86 (t, *J* = 7.4 Hz, 1H), 6.75 – 6.69 (m, 2H), 5.25 (s, 2H), 4.64 (q, *J* = 6.5 Hz, 1H), 4.57 (br, 1H) 2.31 (s, 3H), 2.28 (s, 3H). ^{13}C NMR (126 MHz, $CDCl_3$): δ 167.0, 145.8, 136.4, 134.9, 132.7, 131.2, 130.6, 130.2, 120.9, 114.9, 68.1, 60.39 (q, *J* = 31.4 Hz), 21.6, 18.9. Trifluoromethyl carbon not detected.

^{19}F NMR (376 MHz, $CDCl_3$): δ -72.6 (d, J = 6.7 Hz). GC-MS *m/z* (% relative intensity): 77.1 (19.6), 91.0 (12.1), 104.0 (20.3), 119.1 (100.0), 120.1 (13.3), 174.1 (73.9), 337.2 (9.0).

2,5-Dimethylbenzyl 3,3,3-trifluoro-2-(p-tolylamino)propanoate (5c)

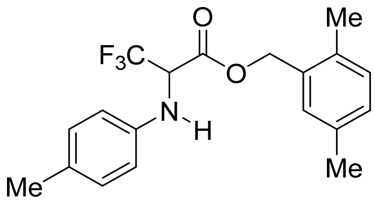

Following the standard procedure above, trifluoropropanoate **5c** was isolated as yellow oil, 16 mg, 95% yield. ^1H NMR (400 MHz, $CDCl_3$): δ 7.12 – 7.08 (m, 3H), 7.03 (d, J = 8.2 Hz, 2H), 6.63 (d, J = 8.2 Hz, 2H), 5.23 (s, 2H), 4.59 (q, J = 7.3 Hz, 1H), 4.45 (br, 1H), 2.31 (s, 3H), 2.27 (s, 3H), 2.26 (s, 3H). ^{13}C NMR (126 MHz, $CDCl_3$): δ 167.1, 143.5, 136.44, 134.9, 132.8, 131.2, 130.7, 130.6, 130.3, 115.2, 68.0, 60.9 (q, J = 31.3 Hz), 21.6, 21.2, 18.9. Trifluoromethyl carbon not detected. ^{19}F NMR (376 MHz, $CDCl_3$): δ -72.6 (d, J = 6.8 Hz). GC-MS *m/z* (% relative intensity): 66.1 (10.2), 77.1 (8.0), 91.1 (30.1), 118.1 (22.9), 119.1 (100.0), 120.1 (12.6), 188.1 (88.3), 351.2 (14.4).

2,5-Dimethylbenzyl 3,3,3-trifluoro-2-(m-tolylamino)propanoate (5d)

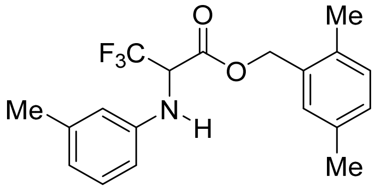

Following the standard procedure above, trifluoropropanoate **5d** was isolated as yellow oil, 11 mg, 71% yield. ^1H NMR (400 MHz, $CDCl_3$): δ 7.13-7.07 (m, 4H), 6.68 (d, J = 7.5 Hz, 1H), 6.53 (s, 1H), 6.52 (d, J = 9.0 Hz, 1H), 5.24 (s, 2H), 4.63 (q, J = 7.0 Hz, 1H), 4.45 (br, 1H), 2.31 (s, 3H), 2.28 (s, 3H), 2.27 (s, 3H). ^{13}C NMR (126 MHz, $CDCl_3$): δ 167.0, 145.8, 140.2, 136.4, 134.9, 132.7, 131.2, 130.6, 130.1, 121.8, 115.8, 112.0, 68.0, 60.4 (q, J = 31.6 Hz), 22.2, 21.6, 18.9. Trifluoromethyl carbon not detected. ^{19}F NMR (376 MHz, $CDCl_3$) δ -72.6 (d, J = 6.9 Hz). GC-MS *m/z* (% relative intensity): 65.1 (9.7), 77.1 (6.9), 91.1 (29.5), 118.1 (18.3), 119.1 (100.0), 188.1 (83.5), 189.1 (8.7), 351.2 (11.8).

2,5-Dimethylbenzyl 3,3,3-trifluoro-2-(o-tolylamino)propanoate (5e)

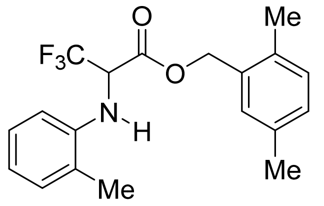

Following the standard procedure above, trifluoropropanoate **5e** was isolated as yellow oil, 13 mg, 84% yield. ^{1}H NMR (400 MHz, $CDCl_3$): δ 7.12 – 7.08 (m, 5H), 6.80 (t, J = 7.2 Hz, 1H), 6.63 (d, J = 8.1 Hz, 1H), 5.25 (d, J = 2.8 Hz, 2H), 4.72 – 4.63 (m, 1H), 4.53 (br, 1H), 2.32 (s, 3H), 2.28 (s, 3H), 2.25 (s, 3H). ^{13}C NMR (126 MHz, $CDCl_3$) δ 167.2, 144.1, 136.4, 134.8, 132.7, 131.5, 131.2, 131.2, 130.6, 127.9, 124.6, 120.6, 112.2, 68.0, 60.5 (q, J = 31.4 Hz), 21.6, 18.9, 18.0. Trifluoromethyl carbon not detected. ^{19}F NMR (376 MHz, $CDCl_3$) δ -72.5 (d, J = 6.9 Hz). GC-MS *m/z* (% relative intensity): 65.1 (10.1), 91.1 (36.7), 117.1 (9.0), 118.1 (20.9), 119.1 (100.0), 120.1 (13.1), 188.1 (87.4), 351.2 (12.8).

2,5-Dimethylbenzyl 2-((4-chlorophenyl)amino)-3,3,3-trifluoropropanoate (5f)

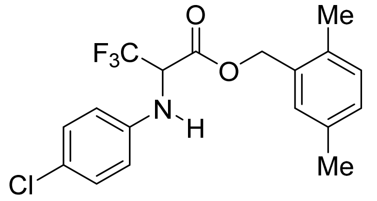

Following the standard procedure above, trifluoropropanoate **5f** was isolated as yellow solid, 17 mg, 92% yield. ^{1}H NMR (400 MHz, $CDCl_3$): δ 7.16 (d, J = 8.8 Hz, 2H), 7.12 – 7.08 (m, 3H), 6.63 (d, J = 8.8 Hz, 2H), 5.25 (s, 2H), 4.61 – 4.53 (m, 2H), 2.31 (s, 3H), 2.28 (s, 3H). ^{13}C NMR (126 MHz, $CDCl_3$) δ 166.7, 144.4, 136.5, 134.9, 132.6, 131.3, 131.2, 130.7, 130.1, 125.7, 116.1, 68.2, 60.4 (q, J = 31.9 Hz), 21.6, 18.9. Trifluoromethyl carbon not detected. ^{19}F NMR (376 MHz, $CDCl_3$) δ -72.5 (d, J = 6.0 Hz). GC-MS *m/z* (% relative intensity): 91.0 (10.6), 119.1 (100.0), 120.1 (13.7), 138.0 (10.4), 208.1 (33.9), 210.1 (10.6), 371.1 (7.4).

2,5-Dimethylbenzyl 2-((4-bromophenyl)amino)-3,3,3-trifluoropropanoate (5g)

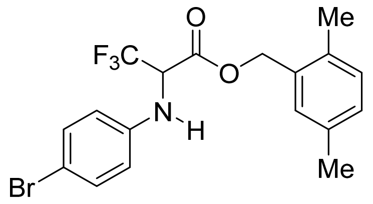

Following the standard procedure above, trifluoropropanoate **5g** was isolated as yellow solid, 13 mg, 71% yield. ^{1}H NMR (400 MHz, $CDCl_3$) δ 7.30 (d, *J* = 8.7 Hz, 2H), 7.12 – 7.08 (m, 3H), 6.58 (d, *J* = 8.7 Hz, 2H), 5.25 (s, 2H), 4.63 – 4.53 (m, 2H), 2.31 (s, 3H), 2.28 (s, 3H). ^{13}C NMR (126 MHz, $CDCl_3$) δ 166.6, 144.8, 136.5, 134.9, 133.0, 132.6, 131.3, 131.2, 130.7, 116.5, 112.8, 68.2, 60.3 (q, *J* = 31.3 Hz), 21.6, 18.9. Trifluoromethyl carbon not detected. ^{19}F NMR (376 MHz, $CDCl_3$) δ -72.5 (d, *J* = 5.9 Hz). GC-MS *m/z* (% relative intensity): 91.0 (10.2), 119.1 (100.0), 252.0 (23.7), 254.0 (22.2), 415.1 (4.8), 417.1 (5.0).

2,5-Dimethylbenzyl 2-((4-(tert-butyl)phenyl)amino)-3,3,3-trifluoropropanoate (5h)

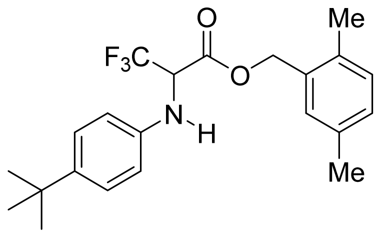

Following the standard procedure above, trifluoropropanoate **5h** was isolated as yellow oil, 14 mg, 81% yield. ^{1}H NMR (400 MHz, $CDCl_3$) δ 7.24 (d, *J* = 8.6 Hz, 2H), 7.12 – 7.08 (m, 3H), 6.66 (d, *J* = 8.6 Hz, 2H), 5.23 (s, 2H), 4.59 (q, *J* = 7.9 Hz, 1H), 4.50 (br, 1H), 2.31 (s, 3H), 2.26 (s, 3H), 1.28 (s, 9H). ^{13}C NMR (126 MHz, $CDCl_3$) δ 143.8, 143.4, 136.4, 134.9, 132.8, 131.2, 130.6, 127.0, 120.7, 114.7, 68.0, 60.7 (q, *J* = 31.8 Hz), 34.7, 32.2, 21.6, 18.9. Trifluoromethyl carbon not detected. ^{19}F NMR (376 MHz, $CDCl_3$) δ -72.7 (d, *J* = 6.8 Hz). GC-MS *m/z* (% relative intensity): 57.1 (5.4), 77.1 (6.2), 91.0 (14.8), 119.1 (100.0), 200.1 (5.0), 215.1 (8.6), 230.2 (87.5), 393.3 (11.8).

2,5-Dimethylbenzyl 2-((4-acetylphenyl)amino)-3,3,3-trifluoropropanoate (5i)

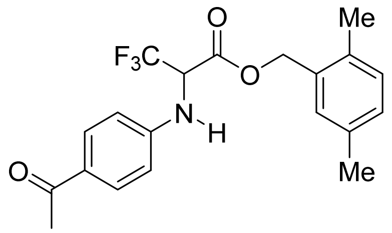

Following the standard procedure above, trifluoropropanoate **5i** was isolated as yellow oil, 14 mg, 71% yield. ^{1}H NMR (400 MHz, $CDCl_3$) δ 7.86 (d, *J* = 8.8 Hz, 2H), 7.14 – 7.06 (m, 3H), 6.70 (d, *J* = 8.8 Hz, 2H), 5.28 (s, 2H), 5.01 (d, *J* = 8.6 Hz, 1H), 4.73 (dq, *J* = 8.6, 6.7 Hz, 1H), 2.52 (s, 3H), 2.31 (s, 3H), 2.29 (s, 3H). ^{13}C NMR (126 MHz, $CDCl_3$) δ 197.1, 166.1, 149.7, 136.5, 134.9, 132.5, 131.4, 131.4, 131.3, 130.8, 130.1, 113.6, 68.4, 59.1 (q, *J* = 31.9 Hz), 26.9, 21.6, 19.0. Trifluoromethyl carbon not detected. ^{19}F NMR (376 MHz, $CDCl_3$) δ -72.3 (d, *J* = 6.7 Hz). GC-MS *m/z* (% relative intensity): 77.1 (8.2), 91.1 (17.7), 104.1 (6.4), 119.1 (100.0), 173.1 (7.8), 216.0 (43.7), 379.2 (4.7).

2,5-Dimethylbenzyl 2-((4-cyanophenyl)amino)-3,3,3-trifluoropropanoate (5j)

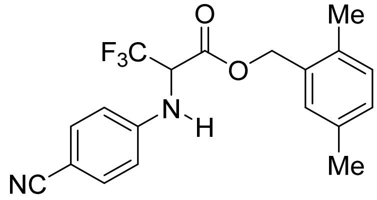

Following the standard procedure above, trifluoropropanoate **5j** was isolated as yellow oil, 12 mg, 65% yield. ^{1}H NMR (400 MHz, $CDCl_3$) δ 7.49 (d, *J* = 8.7 Hz, 2H), 7.11 (s, 3H), 6.74 – 6.67 (m, 2H), 5.28 (s, 2H), 5.04 (d, *J* = 8.5 Hz, 1H), 4.68 (dq, *J* = 8.4, 6.6 Hz, 1H), 2.32 (s, 3H), 2.30 (s, 3H). ^{13}C NMR (126 MHz, $CDCl_3$) δ 165.8, 149.1, 136.5, 135.0, 134.6, 132.4, 131.4, 131.3, 130.9, 120.1, 114.3, 103.1, 68.6, 58.9 (q, *J* = 32.3 Hz), 21.6, 19.0. Trifluoromethyl carbon not detected. ^{19}F NMR (376 MHz, $CDCl_3$) δ -72.2 (d, *J* = 6.7 Hz). GC-MS *m/z* (% relative intensity): 77.1 (6.7), 91.1 (14.9), 102.0 (9.2), 119.1 (100.0), 129.1 (14.7), 199.1 (12.1), 362.2 (1.7).

2,5-Dimethylbenzyl 3,3,3-trifluoro-2-((3-(trifluoromethyl)phenyl)amino)propanoate (5k)

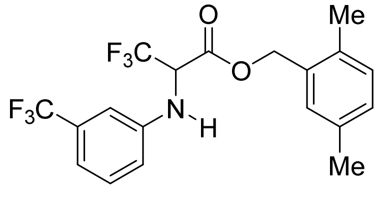

Following the standard procedure above, trifluoropropanoate **5k** was isolated as yellow oil, 13 mg, 63% yield. ^{1}H NMR (400 MHz, $CDCl_3$) δ 7.32 (t, *J* = 7.9 Hz, 1H), 7.14 – 7.06 (m, 4H), 6.91 (t, *J* = 2.0 Hz, 1H), 6.86 (dd, *J* = 7.9, 2.5 Hz, 1H), 5.27 (s, 2H), 4.78 (d, *J* = 8.7 Hz, 1H), 4.65 (dq, *J* = 8.7, 6.7 Hz, 1H), 2.31 (s, 3H), 2.29 (s, 3H). ^{13}C NMR (126 MHz, $CDCl_3$) δ 166.4, 146.1, 136.5, 134.9, 132.8, 132.5, 131.3, 131.3, 130.8, 130.7, 117.8, 117.4, 111.2, 68.4, 59.9 (q, *J* = 32.1 Hz), 21.6, 18.9. Trifluoromethyl carbon not detected. ^{19}F NMR (376 MHz, C_6D_6) δ -63.14, -72.47 (d, *J* = 6.8 Hz). GC-MS *m/z* (% relative intensity): 77.1 (5.8), 91.1 (14.4), 103.1 (4.7), 119.1 (100.0), 145.0 (7.5), 172.1 (14.6), 242.0 (29.5), 405.1 (2.3).

Synthesis of benzyl 2-amino-3,3,3-trifluoropropanoate (7)

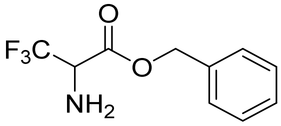

Following the procedure in step V in the synthesis of 2-diazo-3,3,3-trifluoropropanoate derivative, trifluoropropanoate **7** was isolated as yellow oil, 198 mg, 83% yield. ^{1}H NMR (400 MHz, $CDCl_3$) δ 7.42 – 7.31 (m, 5H), 5.25 (s, 2H), 4.05 (q, J = 7.4 Hz, 1H), 2.01 (br, 2H). ^{13}C NMR (126 MHz, $CDCl_3$) δ 167.8, 135.3, 129.4, 129.1, 68.9, 57.9 (q, J = 30.9 Hz). Trifluoromethyl carbon not detected. ^{19}F NMR (376 MHz, $CDCl_3$) δ -75.0 (d, J = 7.2 Hz). GC-MS *m/z* (% relative intensity): 65.1 (14.5), 77.1 (7.6), 91.1 (100.0), 98.0 (35.7).

Synthesis of 3,3,3-trifluoro-2-((4-methoxyphenyl)amino)propan-1-ol (8)

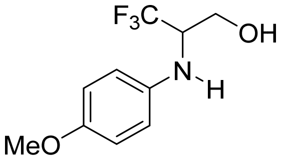

In a flame-dried 5 mL RBF, lithium aluminum hydride (10 mg, 0.275 mmol, 5 eq.) was suspended in 0.5 mL of dry Et_2O. Benzyl 3,3,3-trifluoro-2-((4-methoxyphenyl)amino)propanoate (**3c**) (18.7 mg, 0.055 mmol, 1 eq) dissolved in 0.5 mL of dry Et_2O was added dropwise via syringe and stirred at 0 ºC for 3 hrs. After quenching with sat. $NaHCO_3$ (2 mL), the aqueous layer was extracted with Et_2O (3 x 1 mL). The combined organic layers were dried over $MgSO_4$ and the crude product was purified by silica-gel column chromatography and 30% EtOAc in hexanes isocratic solvent system. The product was obtained as pale-yellow oil (7 mg, 54% yield). ^{1}H NMR (400 MHz, $CDCl_3$) δ 6.80 (d, J = 8.9 Hz, 2H), 6.72 (d, J = 8.9 Hz, 2H), 3.89 (m, 4H), 3.76 (s, 3H). ^{13}C NMR (101 MHz, $CDCl_3$) δ 171.2, 153.6, 139.8, 116.1, 114.9, 59.8, 58.7 (q, J = 27.3 Hz), 55.6. Trifluoromethyl carbon not detected. ^{19}F NMR (376 MHz, $CDCl_3$) δ -73.8 (d, J = 7.1 Hz). GC-MS *m/z* (% relative intensity): 108.1 (10.6), 123.1 (10.4), 134.1 (29.0), 157.1 (19.1), 166.1 (10.0), 204.1 (100.0), 235.1 (61.2).

Synthesis of 3,3,3-trifluoro-2-((4-methoxyphenyl)amino)-1,1-diphenylpropan-1-ol (9)

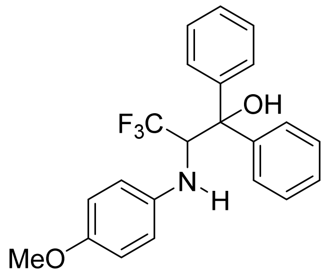

In a flame-dried 5 mL RBF, benzyl 3,3,3-trifluoro-2-((4-methoxyphenyl)amino)propanoate (**3c**) (18.7 mg, 0.082 mmol, 1 eq) was dissolved in 0.5 mL of dry Et_2O and 3M phenylmagnesium bromide in THF (69 µL, 0.328 mmol, 4 eq) was added dropwise via syringe and stirred at 0 ºC for 3 hrs. After quenching with sat. $NaHCO_3$ (2 mL), the aqueous layer was extracted with Et_2O (3 x 1 mL). The combined organic layers were dried over $MgSO_4$ and the crude product was purified by silica-gel column chromatography and 20% EtOAc in hexanes isocratic solvent system. The product was obtained as brown oil (7 mg, 54% yield). ^{1}H NMR (400 MHz, $CDCl_3$) δ 7.50 – 7.45 (m, 2H), 7.35 – 7.31 (m, 4H), 7.24 – 7.07 (m, 4H), 6.73 (d, *J* = 8.8 Hz, 2H), 6.53 (d, *J* = 8.8 Hz, 2H), 4.80 (q, *J* = 7.1 Hz, 1H), 3.75 (s, 3H), 2.98 (br, 1H). ^{13}C NMR (126 MHz, $CDCl_3$) δ 138.4, 133.2, 130.8, 129.1, 129.0, 129.0, 128.3, 128.2, 126.3, 126.0, 115.7, 115.5, 56.4, 30.4. Trifluoromethyl carbon not detected. ^{19}F NMR (376 MHz, $CDCl_3$) δ -65.98 (d, *J* = 7.2 Hz). GC-MS *m/z* (% relative intensity): 51.1 (7.3), 77.1 (58.2), 92.0 (9.4), 105.0 (100.0), 134.1 (11.4), 157.1 (5.8), 183.2 (47.3), 205.1 (31.2).

Synthesis *N*-(3-bromo-1,1,1-trifluoropropan-2-yl)-4-methoxyaniline (10)

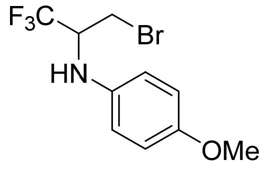

In a flame-dried 5 mL RBF, 3,3,3-trifluoro-2-((4-methoxyphenyl)amino)propan-1-ol (8) (9.3 mg, 0.039 mmol, 1 eq) was dissolved in 100 µL of dry DCM and tetrabutylammonium bromide (14 mg, 0.059 mol, 1.5 eq) and 2,6-lutidine (15 µL, 0.119 mol, 3 eq.). XtalFluor-E (15 mg, 0.059 mol, 1.5 eq.) was then added and the solution was stirred for 3 hrs at room temperature. After the reaction was quenched with sat. $NaHCO_3$ (1 mL), the aqueous layer was extracted with DCM (3 x 1 mL). The combined organic layers were dried over $MgSO_4$ and the crude product was purified by silica-gel column chromatography and 10% EtOAc in hexanes isocratic solvent system. The

product was obtained as slightly yellow oil (3 mg, 24% yield). ^{1}H NMR (400 MHz, $CDCl_3$) δ 6.81 (d, J = 9.0 Hz, 1H), 6.72 (d, J = 9.0 Hz, 1H), 4.17 – 4.04 (m, 1H), 3.76 (s, 1H), 3.73 – 3.67 (m, 2H), 3.58 – 3.50 (m, 1H). ^{13}C NMR (126 MHz, $CDCl_3$) δ 154.6, 140.0, 117.1, 115.7, 59.1 (q, J = 29.4 Hz), 56.4, 29.6. Trifluoromethyl carbon not detected. ^{19}F NMR (376 MHz, $CDCl_3$) δ -74.1 (d, J = 6.4 Hz). GC-MS *m/z* (% relative intensity): 52.1 (59.5), 63.0 (38.5), 69.0 (26.6), 77.0 (51.0), 95.0 (39.3), 107.0 (38.0), 122.1 (56.7), 134.1 (76.6), 146.1 (100), 157.0 (22.1), 176.9 (7.4), 184.1 (6.2), 199.1 (7.7), 204.0 (71.5), 227.9 (16.6), 229.9 (17.3), 297.0 (25.8), 299.0 (21.7).

NMR Spectra

Ethyl 2-diazo-3,3,3-trifluoropropanoate (**2a**), ^{1}H NMR (400 MHz, $CDCl_3$):

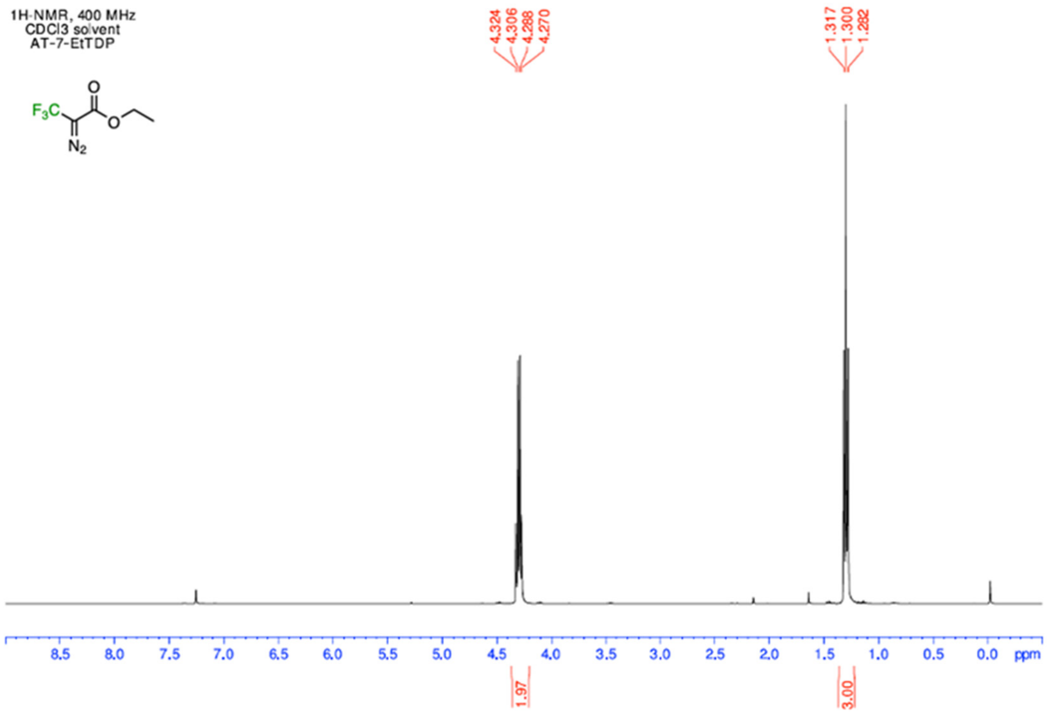

Ethyl 2-diazo-3,3,3-trifluoropropanoate (**2a**), ^{13}C NMR (126 MHz, $CDCl_3$):

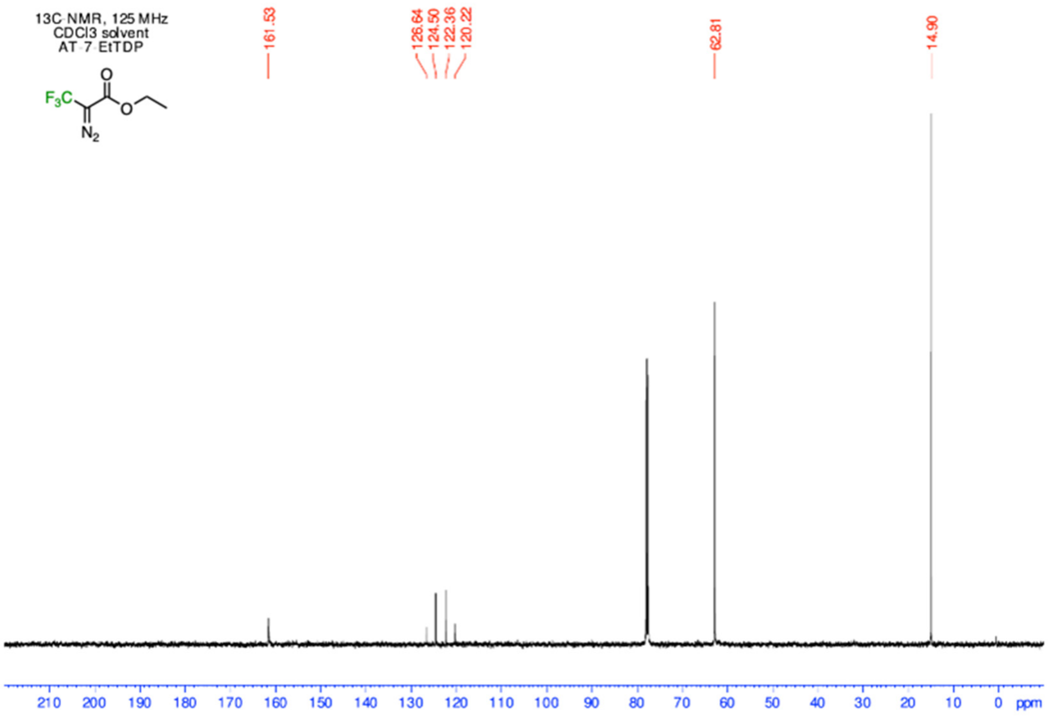

Ethyl 2-diazo-3,3,3-trifluoropropanoate (**2a**), ^{19}F NMR (376 MHz, $CDCl_3$):

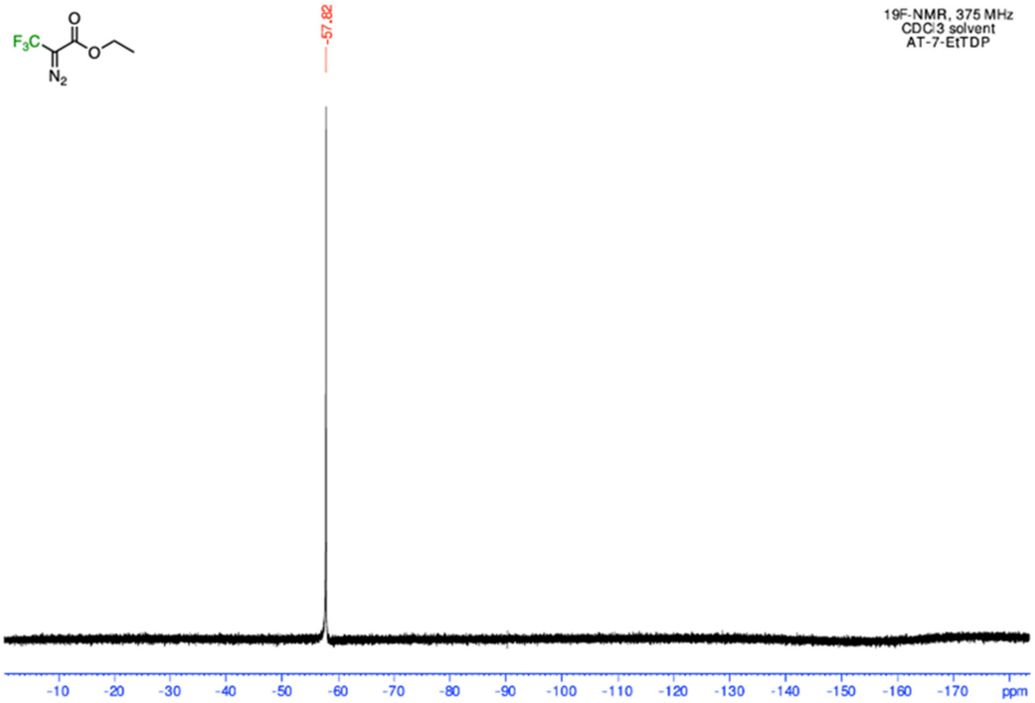

2,2,2-Trifluoro-N-(4-methoxyphenyl)acetimidoyl chloride (**11**), ^{1}H NMR (400 MHz, $CDCl_3$):

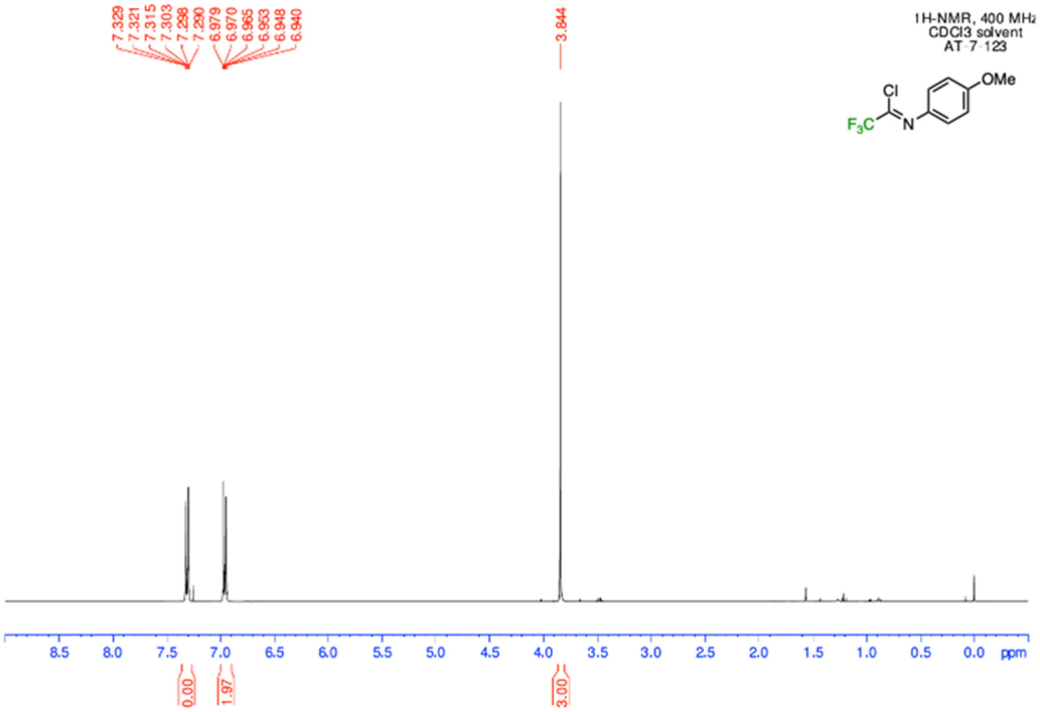

2,2,2-Trifluoro-N-(4-methoxyphenyl)acetimidoyl chloride (**11**), ^{13}C NMR (126 MHz, $CDCl_3$):

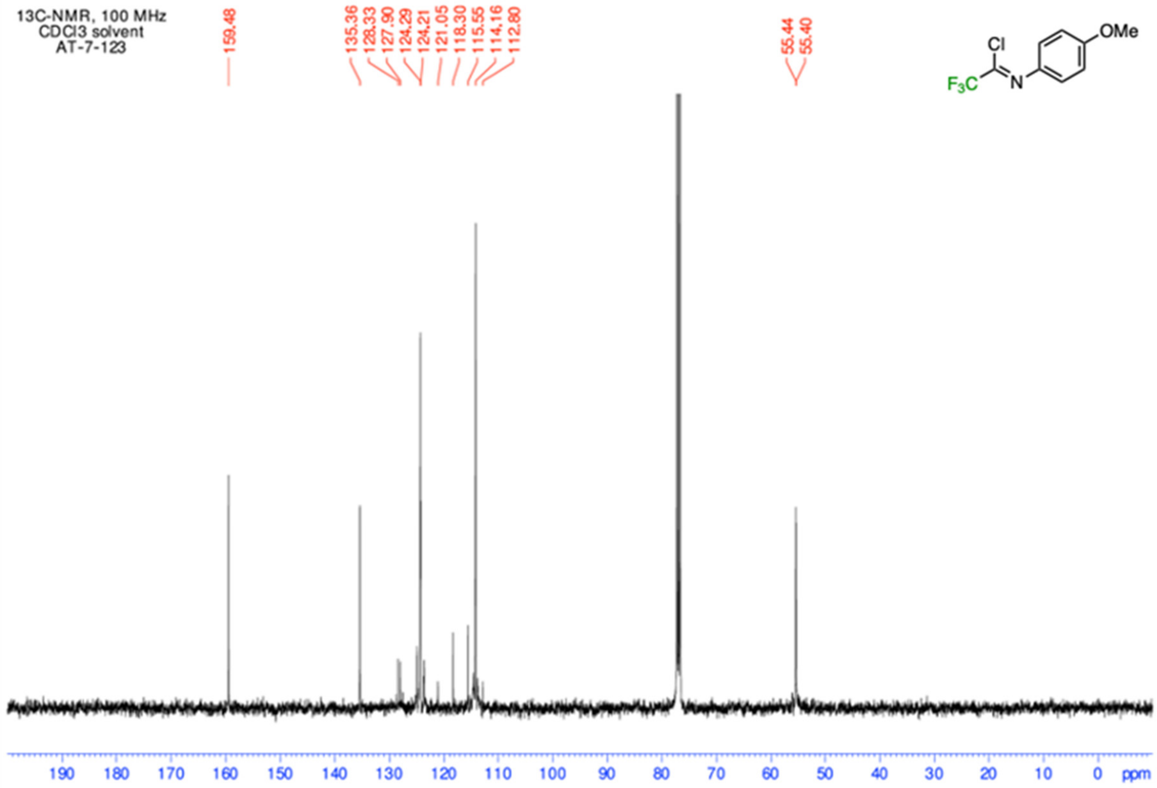

2,2,2-Trifluoro-N-(4-methoxyphenyl)acetimidoyl chloride (**11**), ^{19}F NMR (376 MHz, $CDCl_3$):

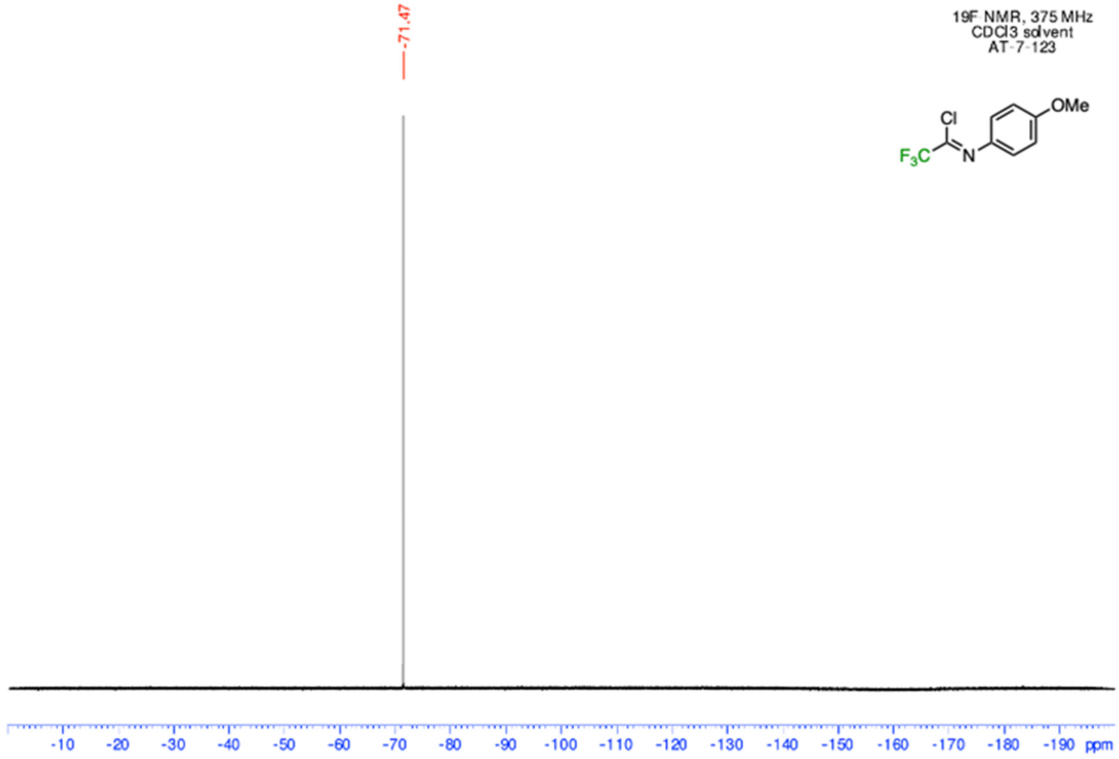

2,2,2-Trifluoro-N-(4-methoxyphenyl)acetimidoyl iodide (**12**), ^{1}H NMR (400 MHz, $CDCl_3$):

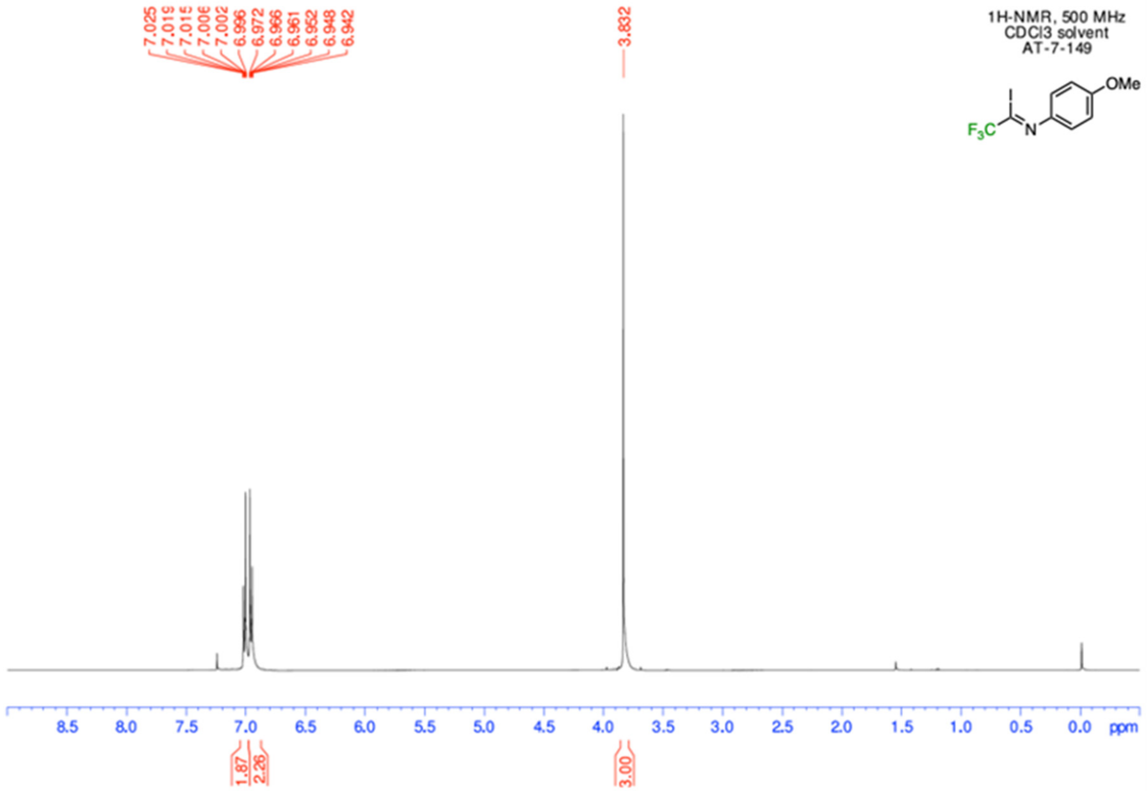

2,2,2-Trifluoro-N-(4-methoxyphenyl)acetimidoyl iodide (**12**), ^{13}C NMR (126 MHz, $CDCl_3$):

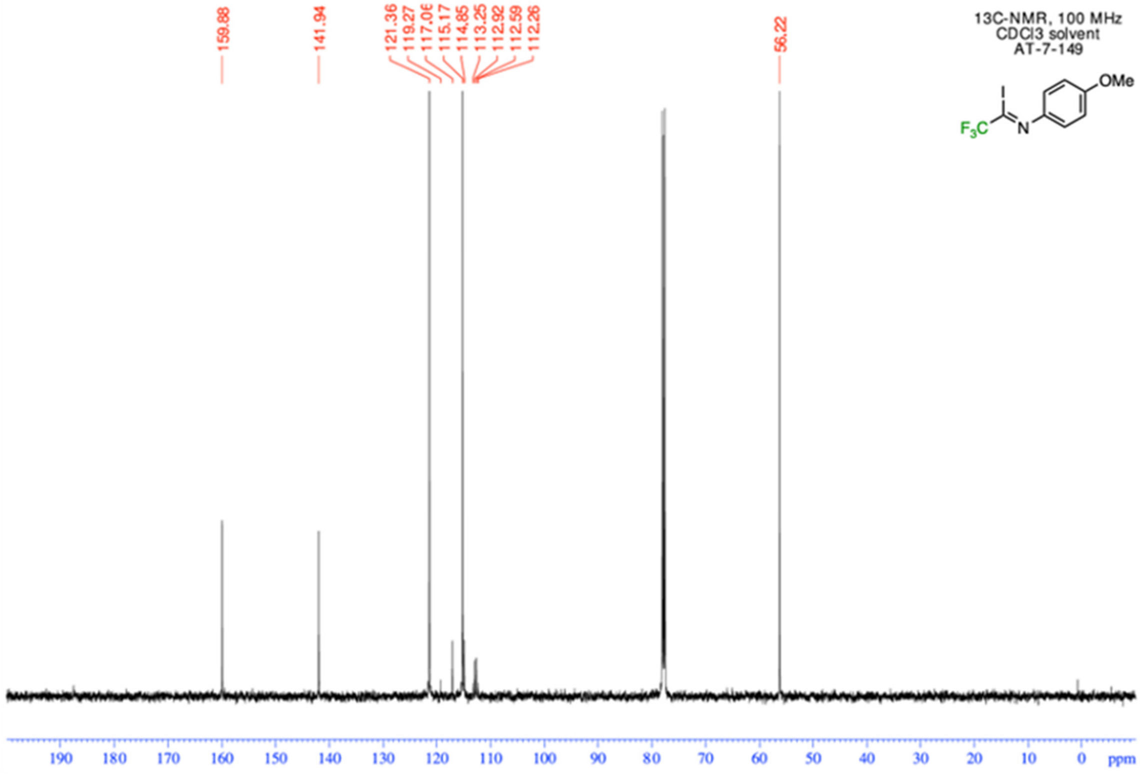

2,2,2-Trifluoro-N-(4-methoxyphenyl)acetimidoyl iodide (**12**), ^{19}F NMR (376 MHz, $CDCl_3$):

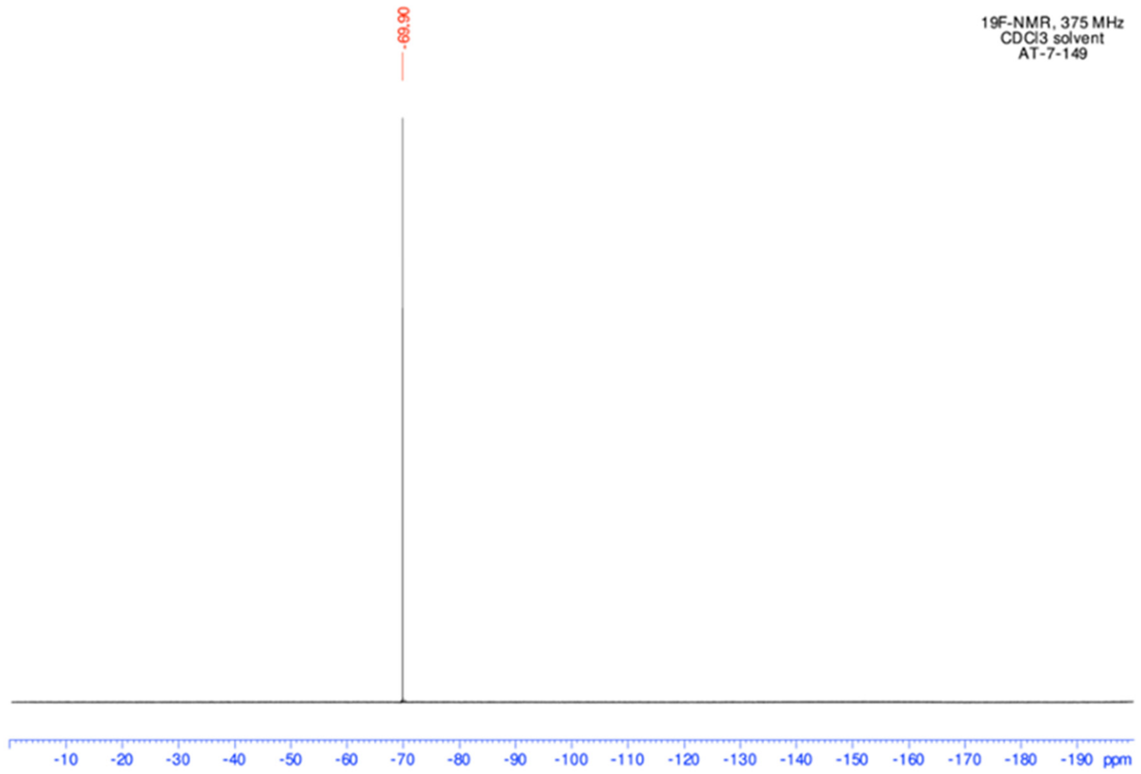

Benzyl 3,3,3-trifluoro-2-((4-methoxyphenyl)imino)propanoate (**13**), ^{1}H NMR (400 MHz, $CDCl_3$):

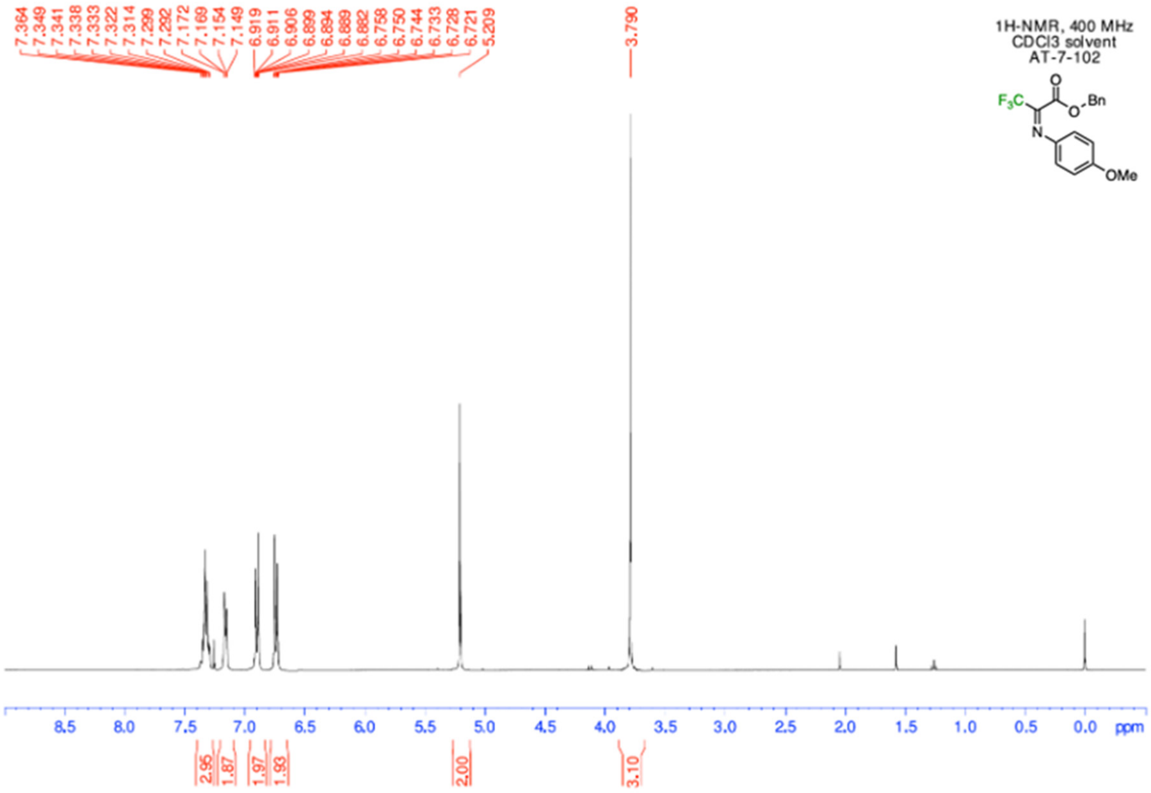

Benzyl 3,3,3-trifluoro-2-((4-methoxyphenyl)imino)propanoate (**13**), ^{13}C NMR (126 MHz, $CDCl_3$):

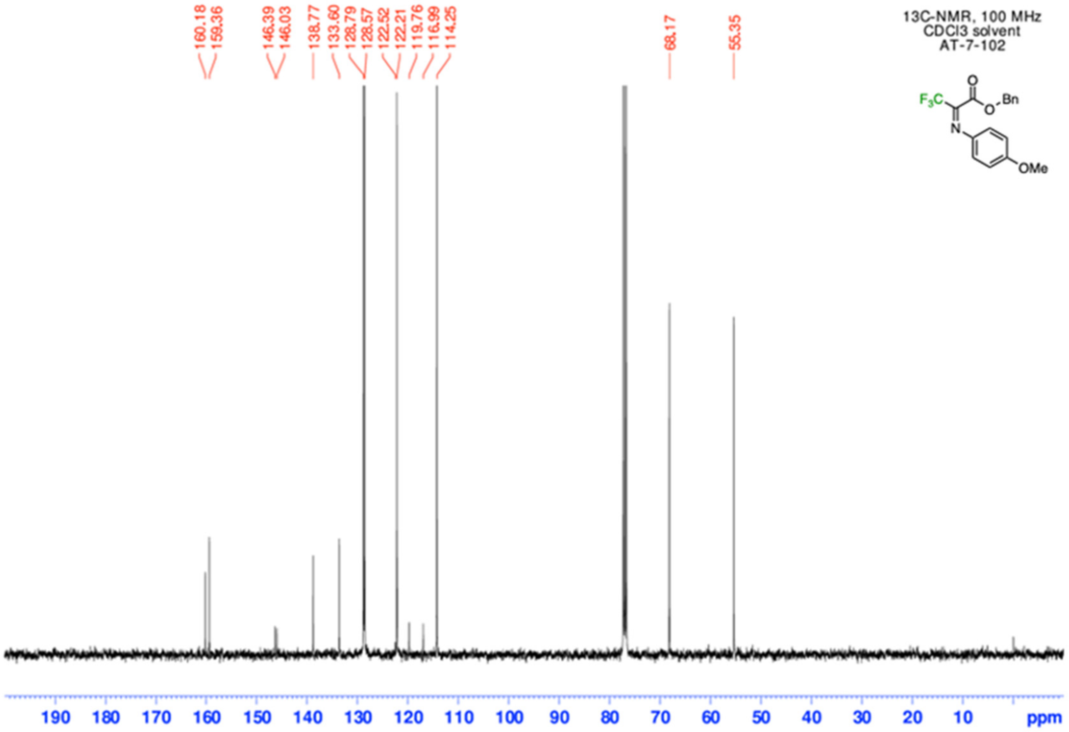

Benzyl 3,3,3-trifluoro-2-((4-methoxyphenyl)imino)propanoate (**13**), ^{19}F NMR (376 MHz, $CDCl_3$):

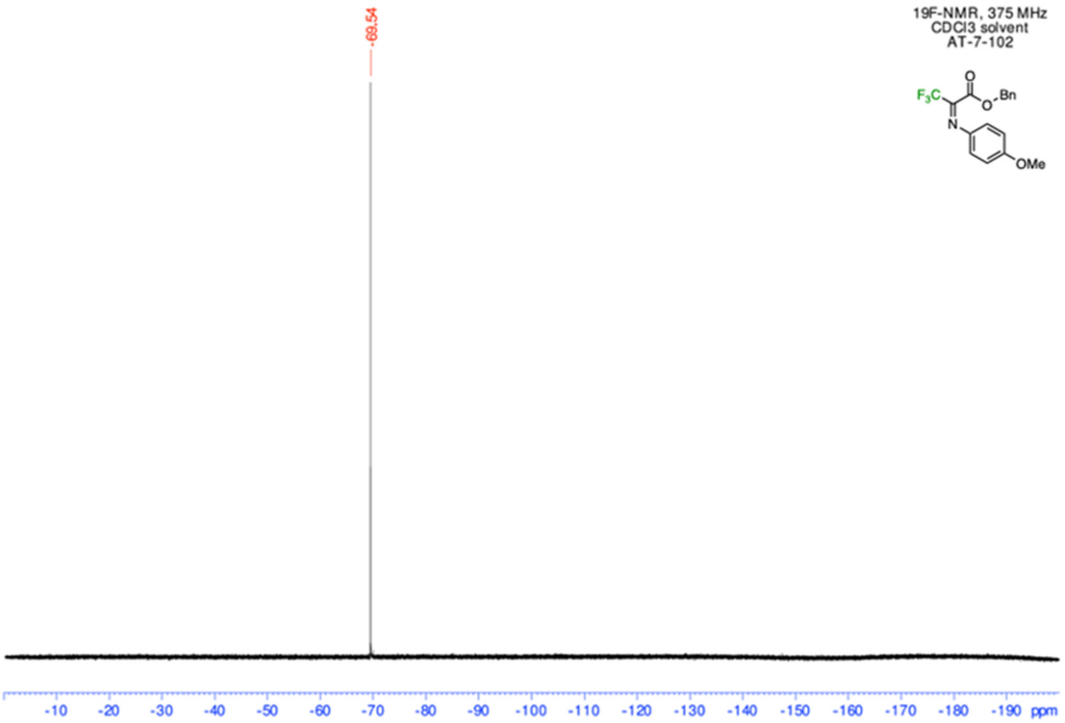

Benzyl 3,3,3-trifluoro-2-((4-methoxyphenyl)amino)propanoate (**3c**), ^{1}H NMR (400 MHz, $CDCl_3$):

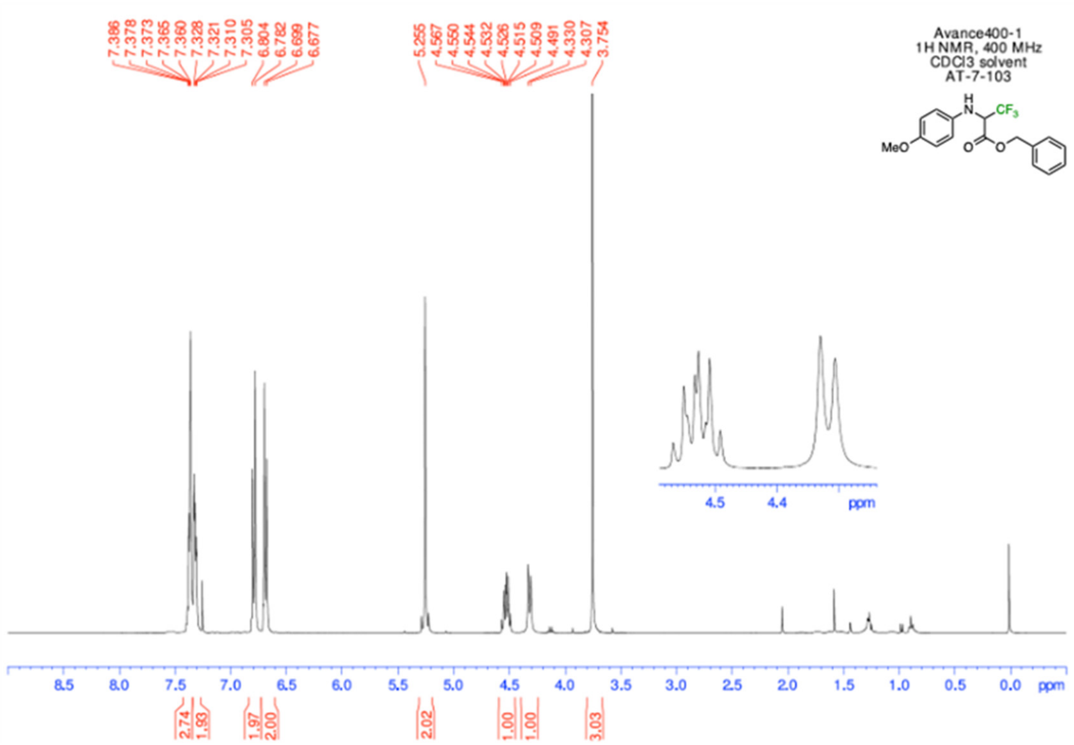

Benzyl 3,3,3-trifluoro-2-((4-methoxyphenyl)amino)propanoate (**3c**), ^{13}C NMR (126 MHz, $CDCl_3$):

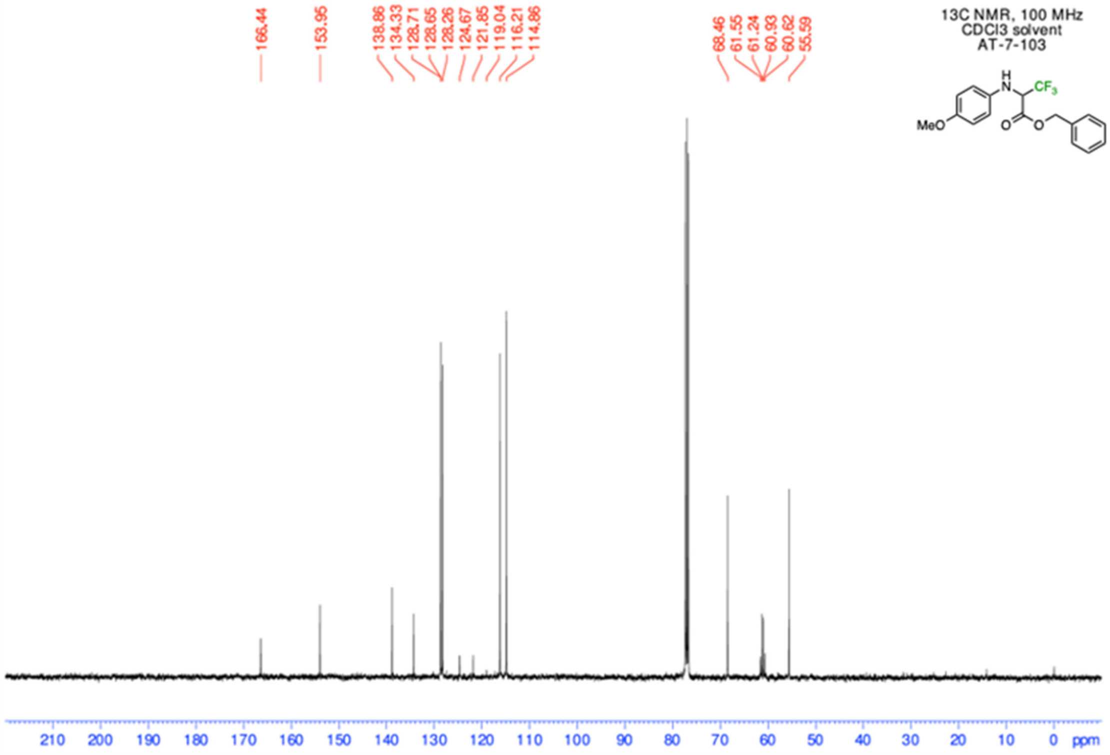

Benzyl 3,3,3-trifluoro-2-((4-methoxyphenyl)amino)propanoate (**3c**), ^{19}F NMR (376 MHz, $CDCl_3$):

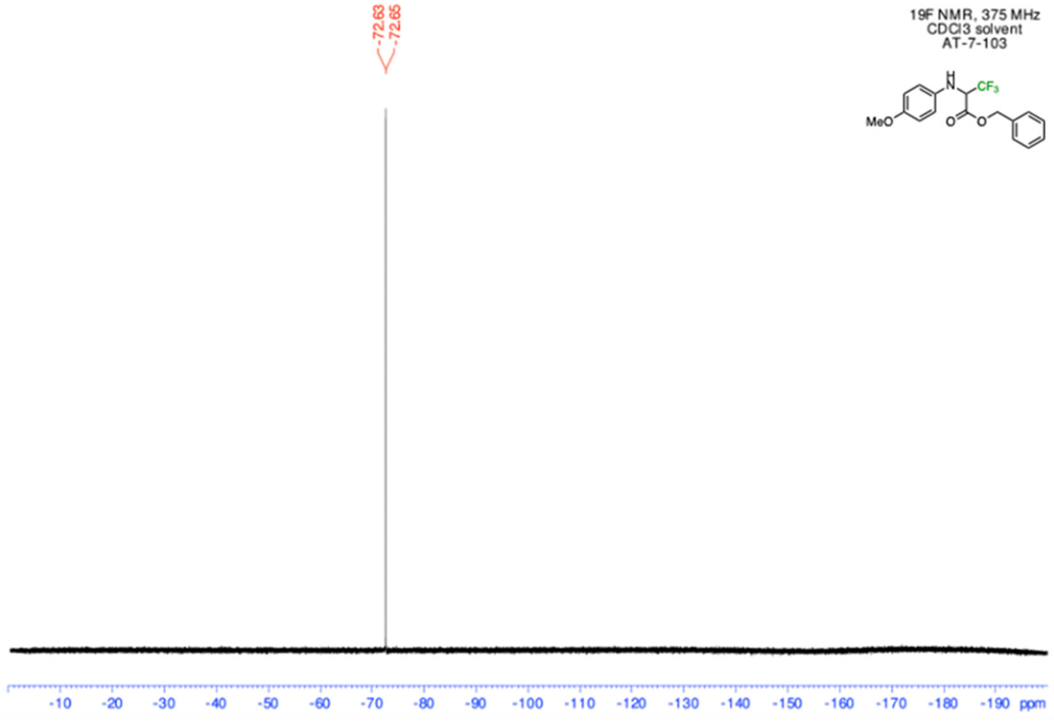

Cyclohexyl 2-diazo-3,3,3-trifluoropropanoate (**2b**), ^{1}H NMR (400 MHz, $CDCl_3$):

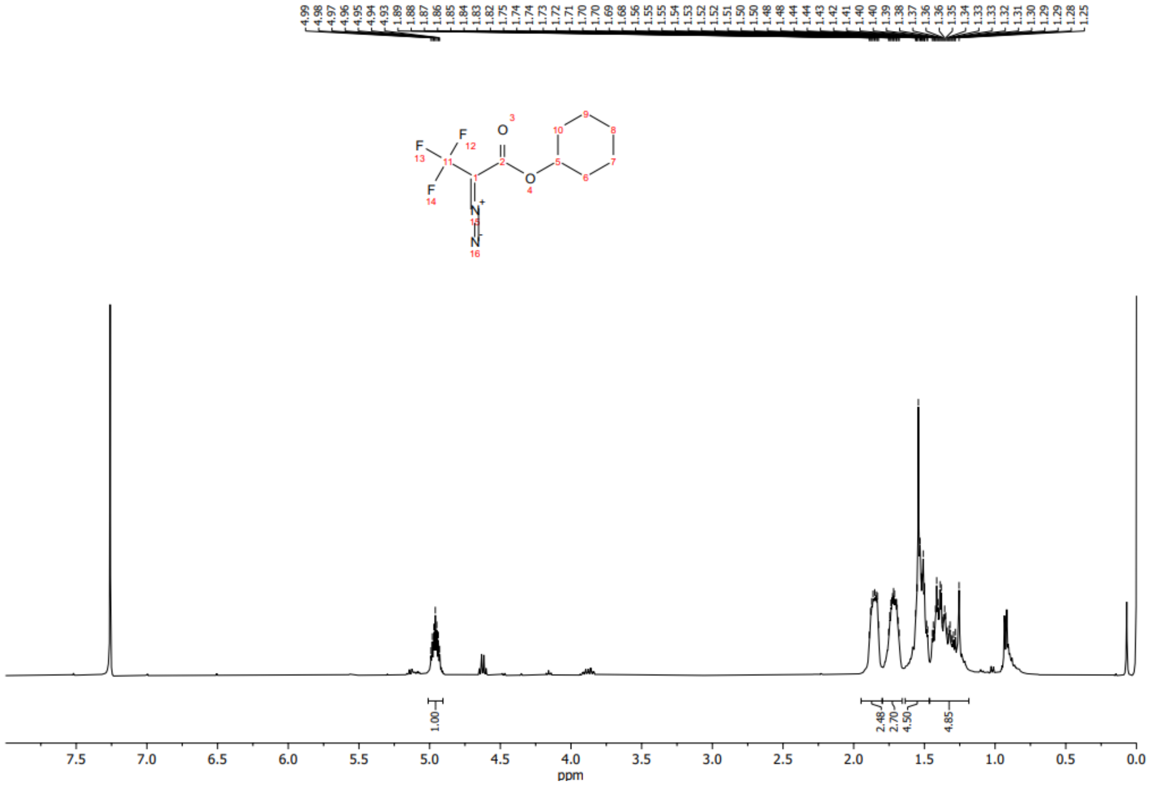

Cyclohexyl 2-diazo-3,3,3-trifluoropropanoate (**2b**), ^{13}C NMR (126 MHz, $CDCl_3$):

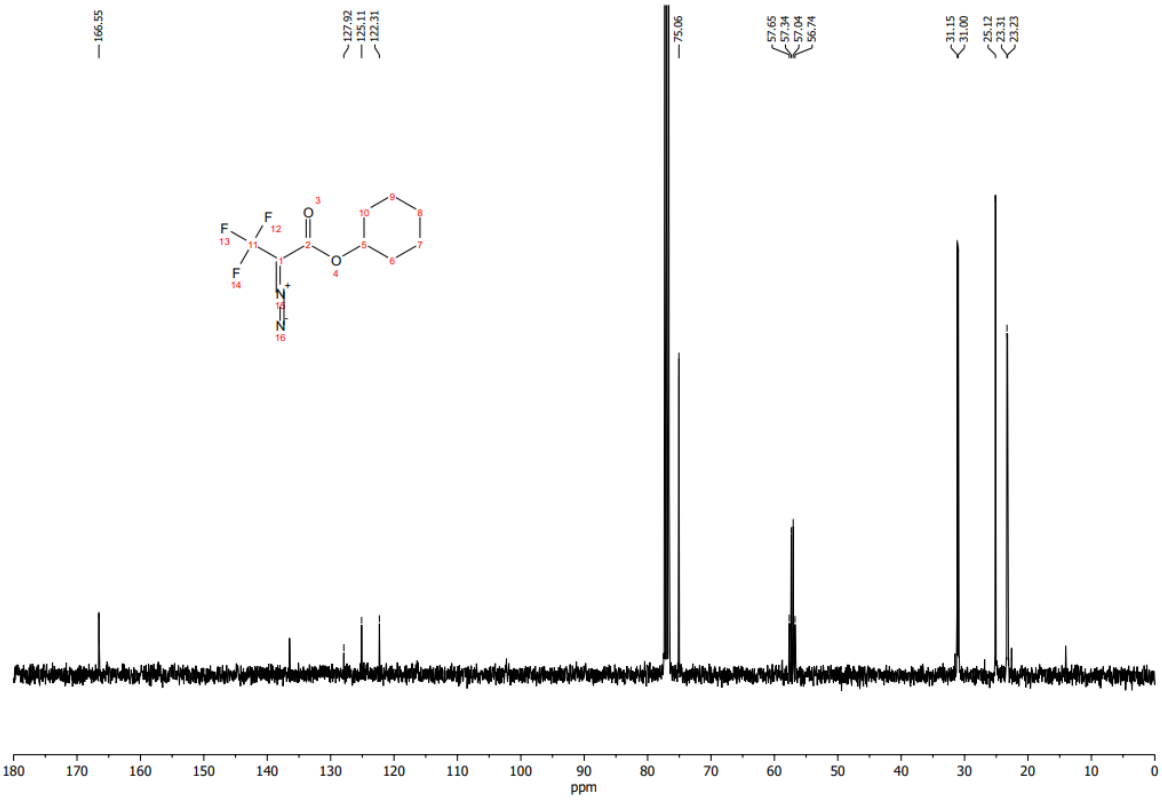

Cyclohexyl 2-diazo-3,3,3-trifluoropropanoate (**2b**), ^{19}F NMR (376 MHz, $CDCl_3$):

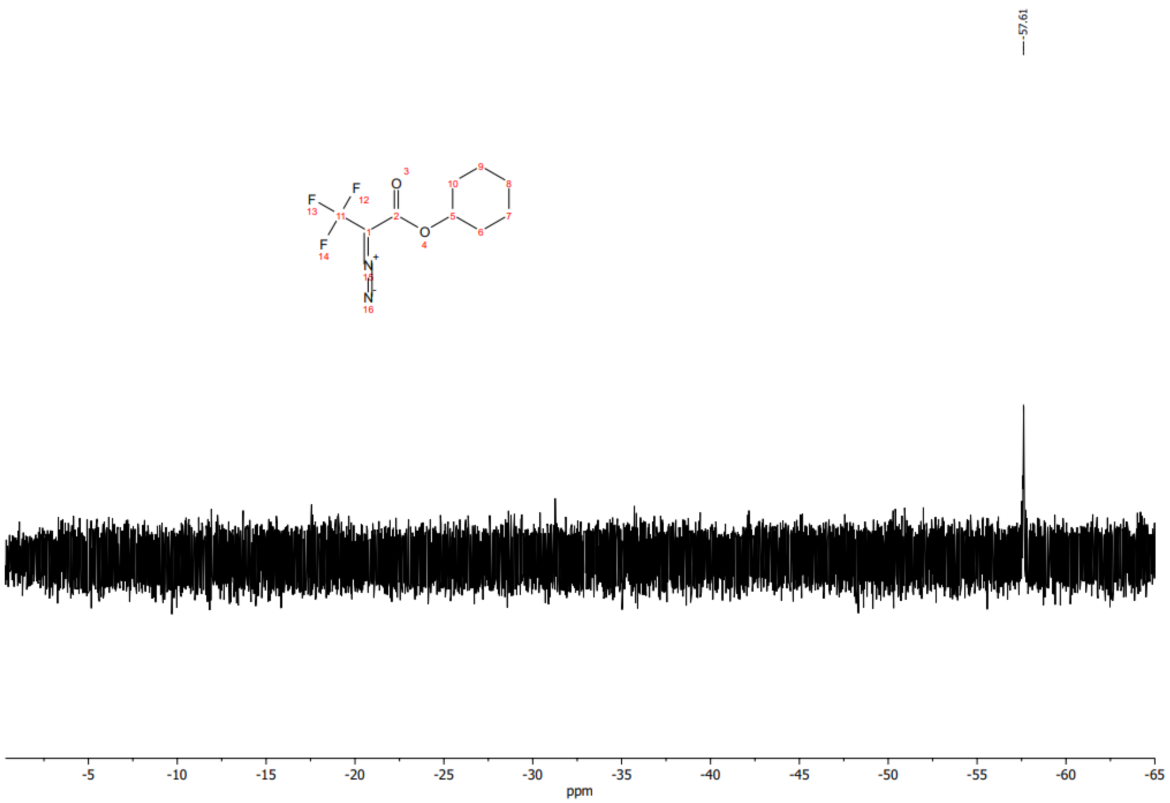

Benzyl 2-diazo-3,3,3-trifluoropropanoate (**2c**), ^{1}H NMR (400 MHz, $CDCl_3$):

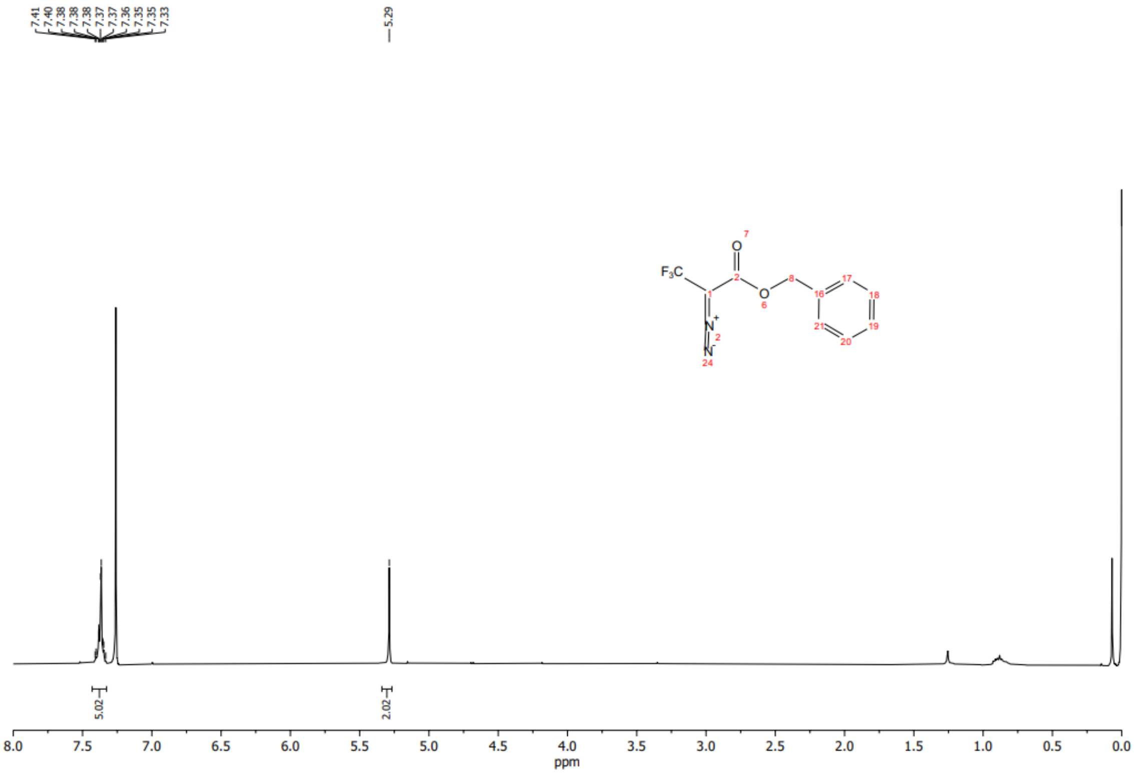

Benzyl 2-diazo-3,3,3-trifluoropropanoate (**2c**), ^{13}C NMR (126 MHz, $CDCl_3$):

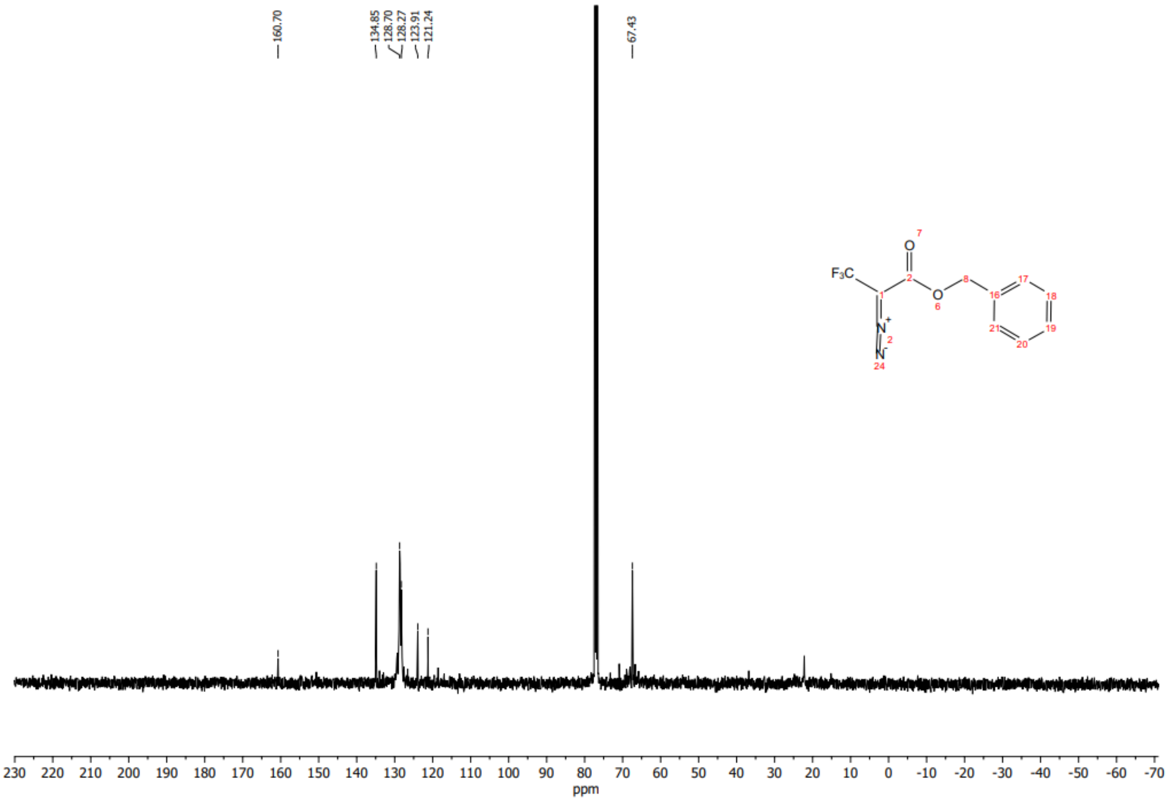

Benzyl 2-diazo-3,3,3-trifluoropropanoate (**2c**), ^{19}F NMR (376 MHz, $CDCl_3$):

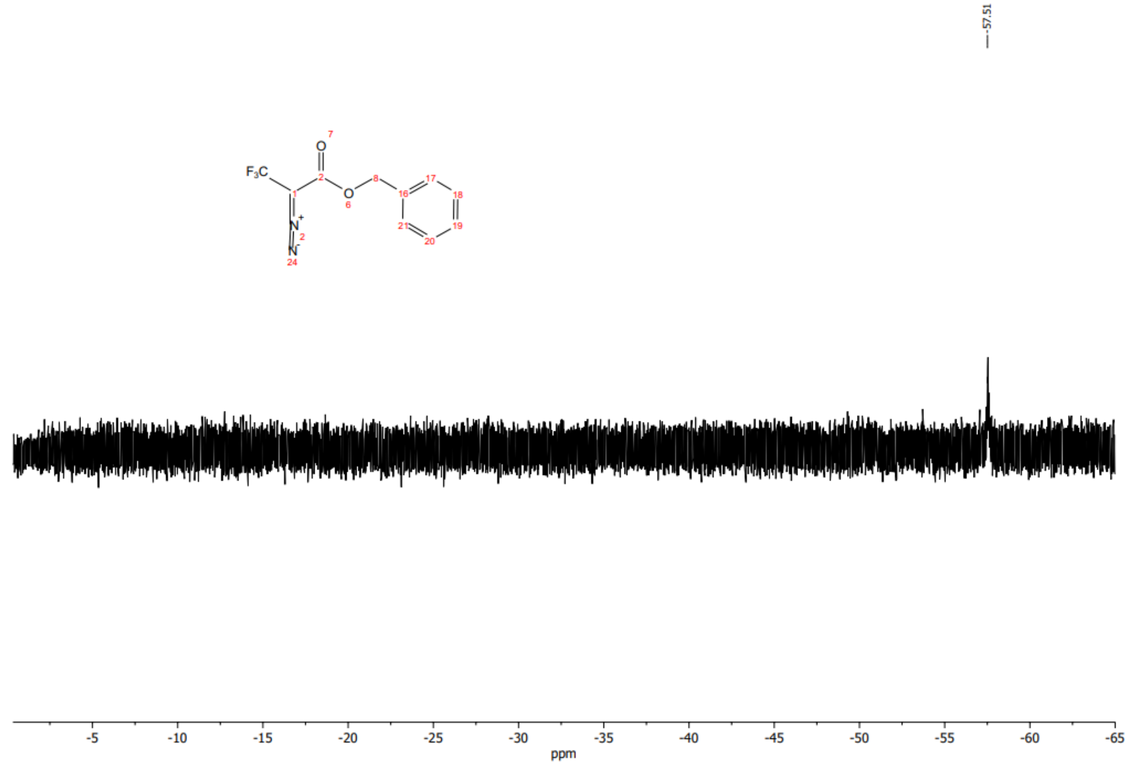

Naphthalen-1-ylmethyl 2-diazo-3,3,3-trifluoropropanoate (**2d**), ^{1}H NMR (400 MHz, $CDCl_3$):

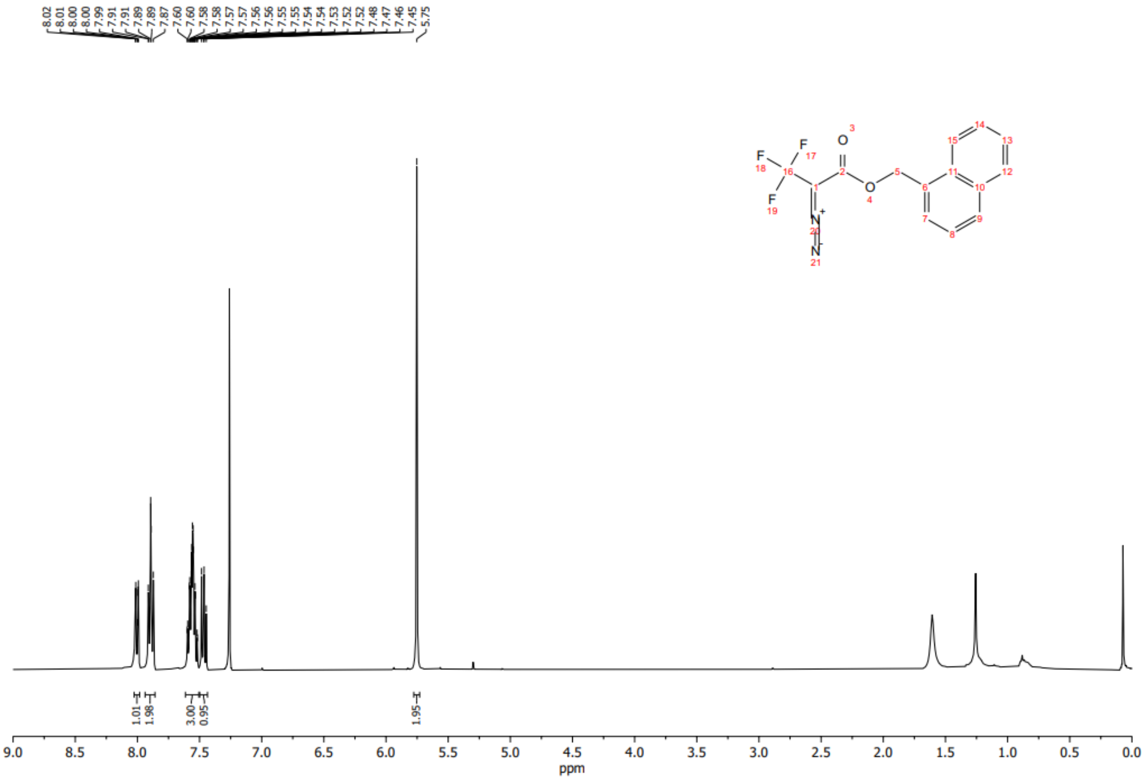

Naphthalen-1-ylmethyl 2-diazo-3,3,3-trifluoropropanoate (**2d**), ^{13}C NMR (126 MHz, $CDCl_3$):

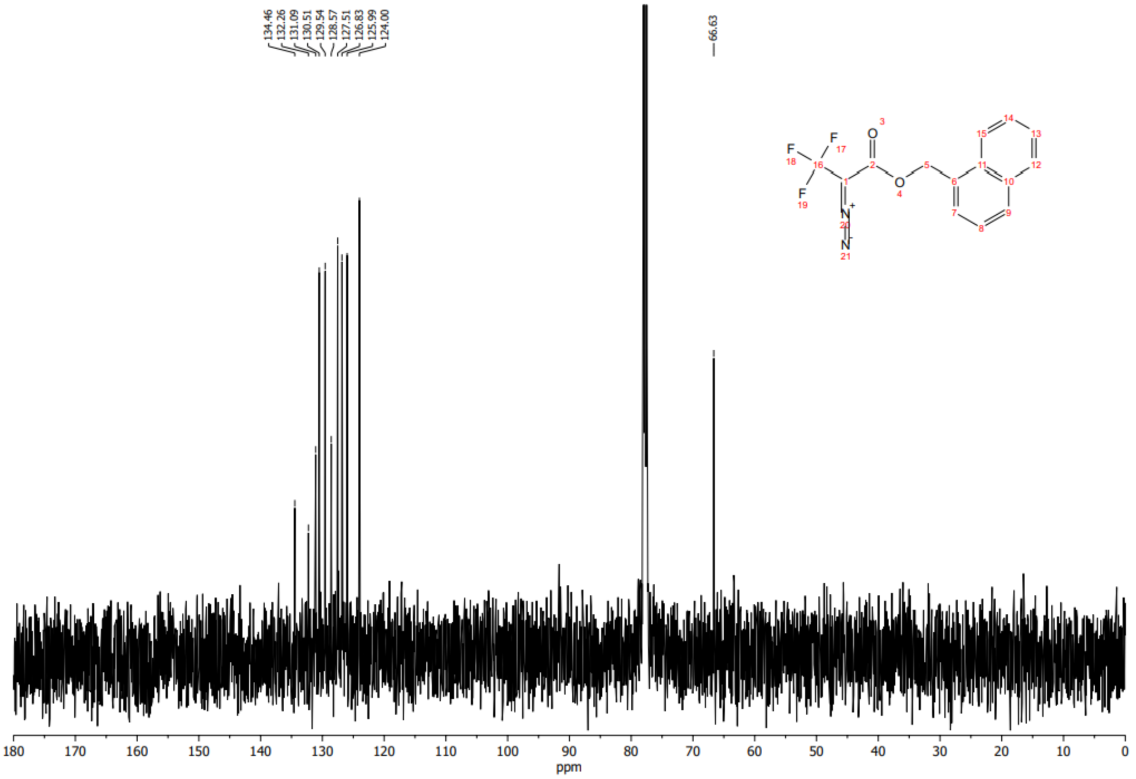

Naphthalen-1-ylmethyl 2-diazo-3,3,3-trifluoropropanoate (**2d**), ^{19}F NMR (376 MHz, $CDCl_3$):

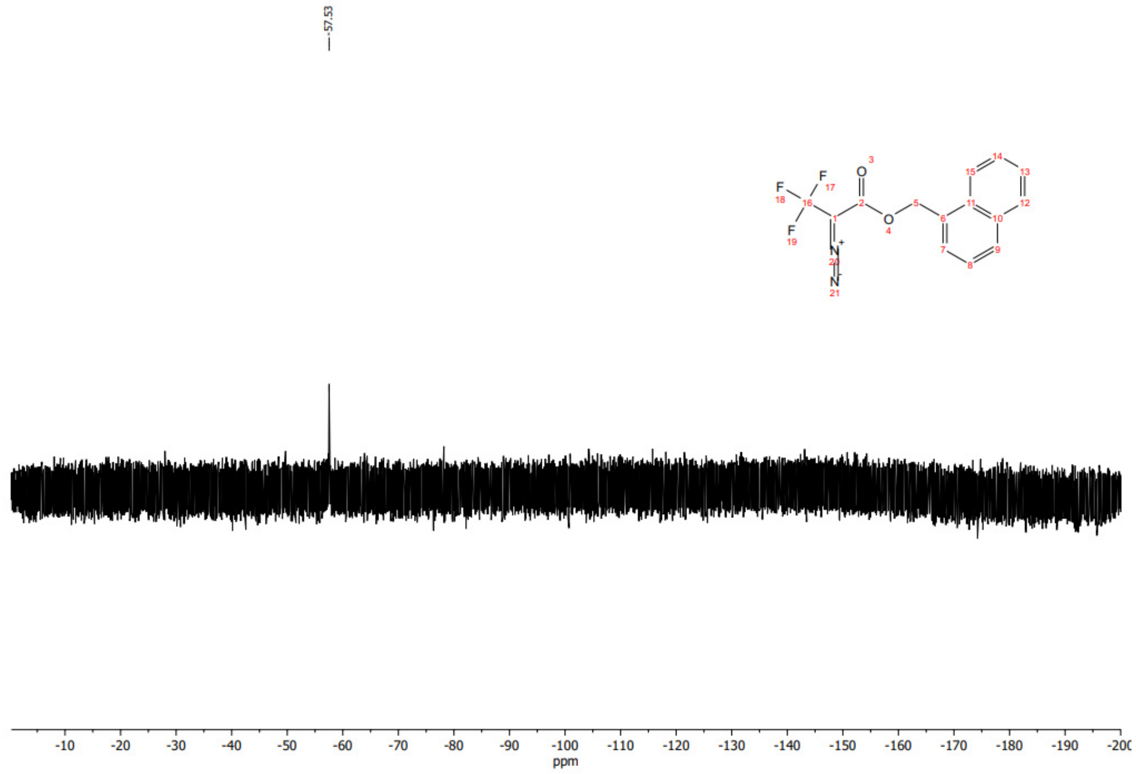

2-Methylbenzyl 2-diazo-3,3,3-trifluoropropanoate (**2e**), ^{1}H NMR (400 MHz, $CDCl_3$):

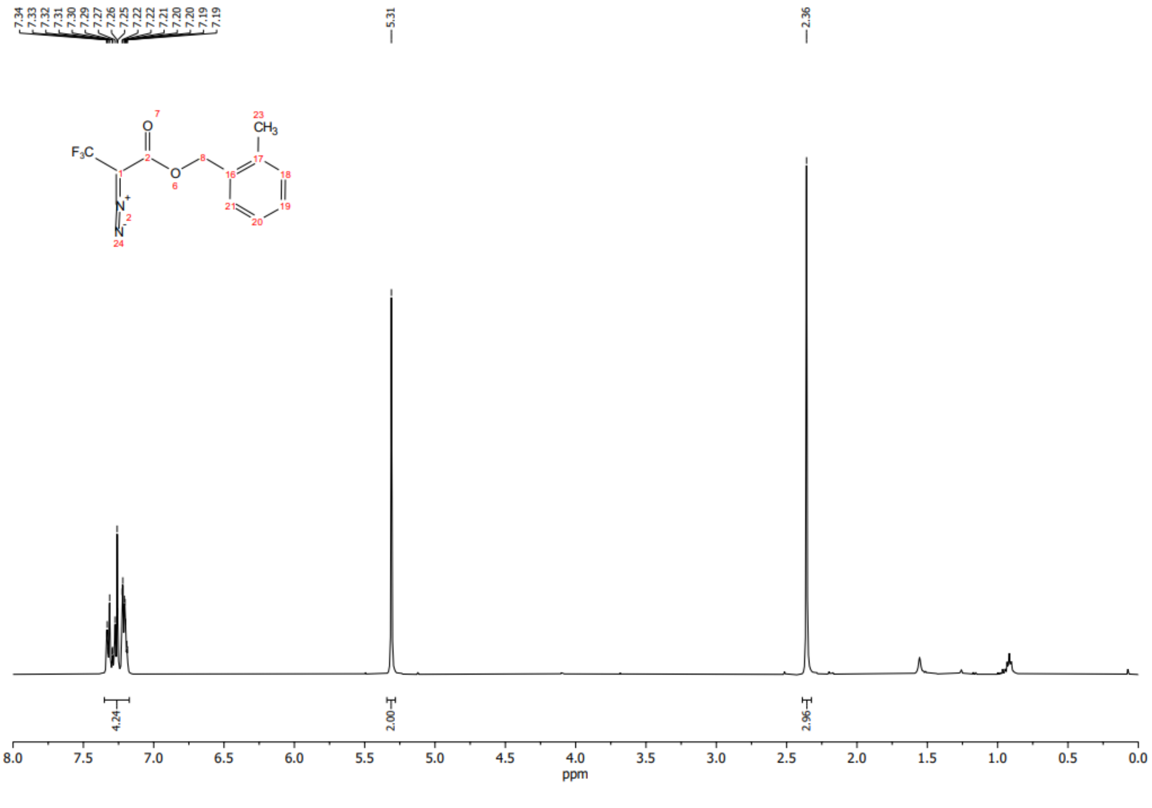

2-Methylbenzyl 2-diazo-3,3,3-trifluoropropanoate (**2e**), ^{13}C NMR (126 MHz, $CDCl_3$):

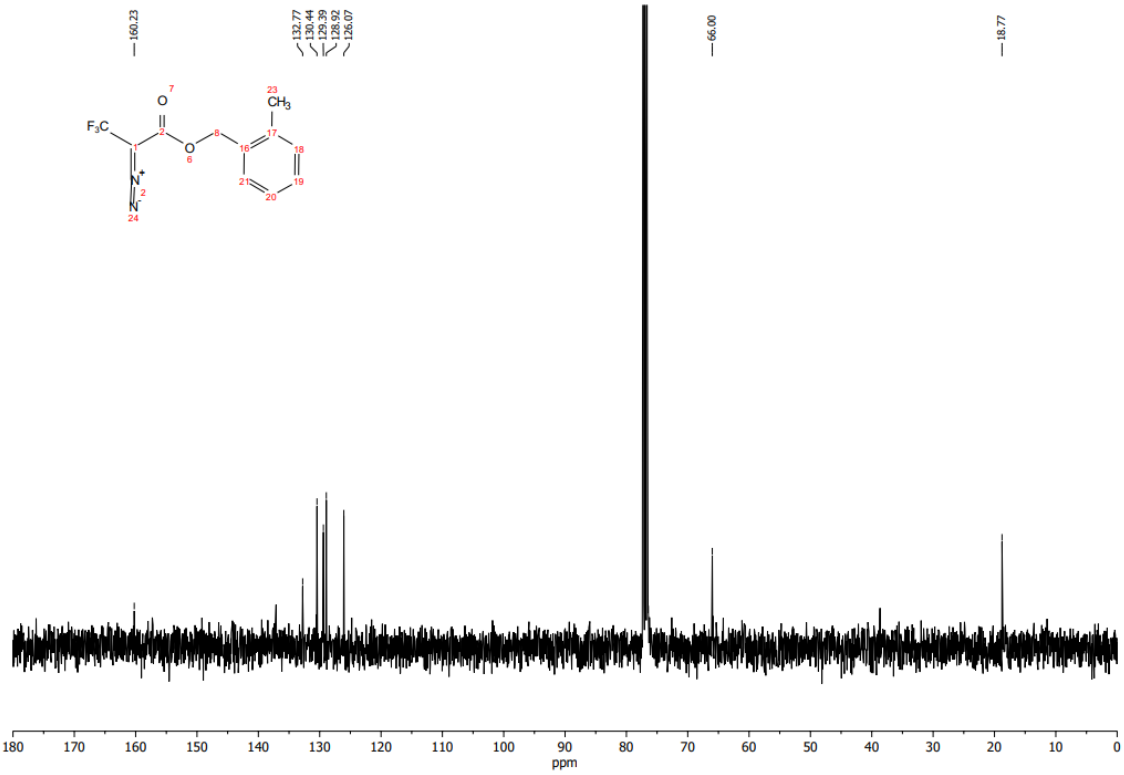

2-Methylbenzyl 2-diazo-3,3,3-trifluoropropanoate (**2e**), ^{19}F NMR (376 MHz, $CDCl_3$):

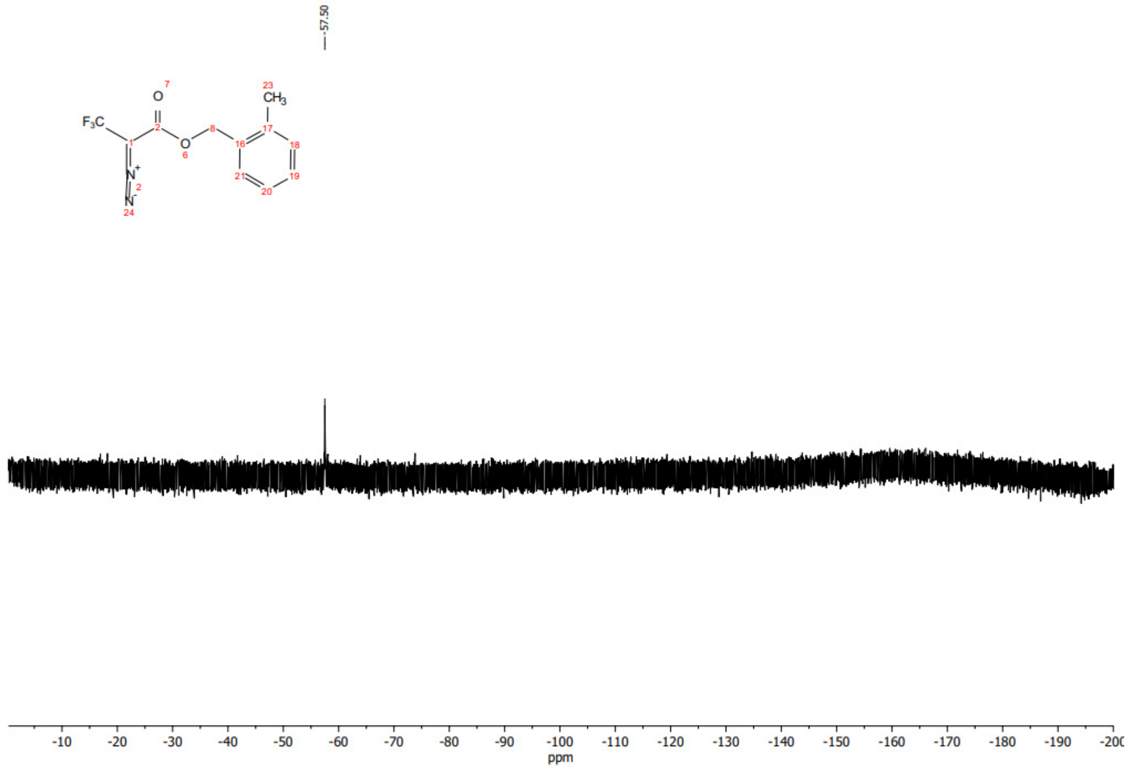

2,4-Dimethylbenzyl 2-diazo-3,3,3-trifluoropropanoate (**2f**), ^{1}H NMR (400 MHz, $CDCl_3$):

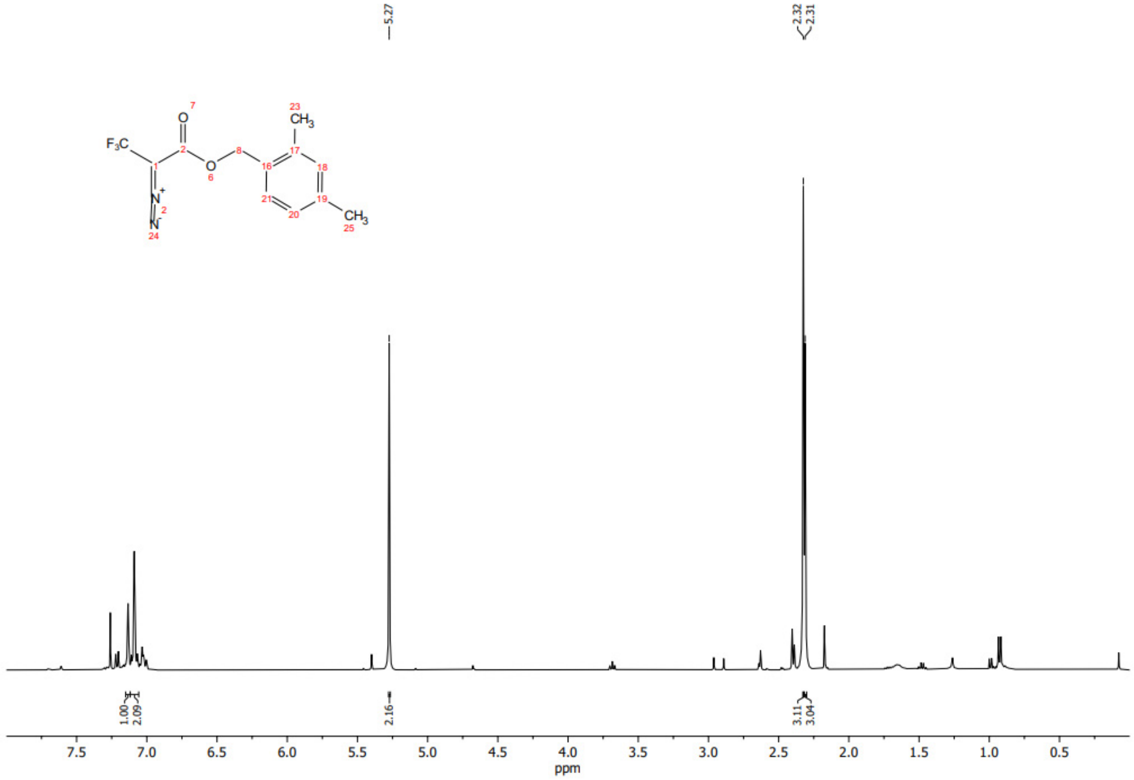

2,4-Dimethylbenzyl 2-diazo-3,3,3-trifluoropropanoate (**2f**), ^{13}C NMR (126 MHz, $CDCl_3$):

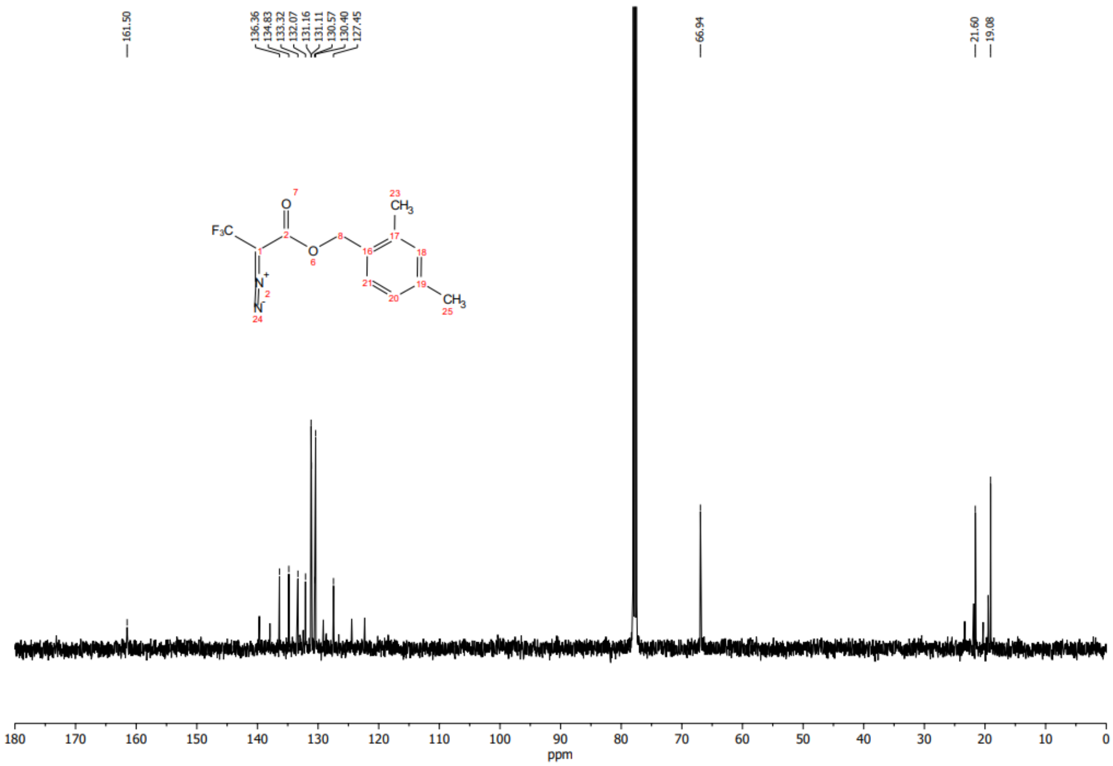

2,4-Dimethylbenzyl 2-diazo-3,3,3-trifluoropropanoate (**2f**), ^{19}F NMR (376 MHz, $CDCl_3$):

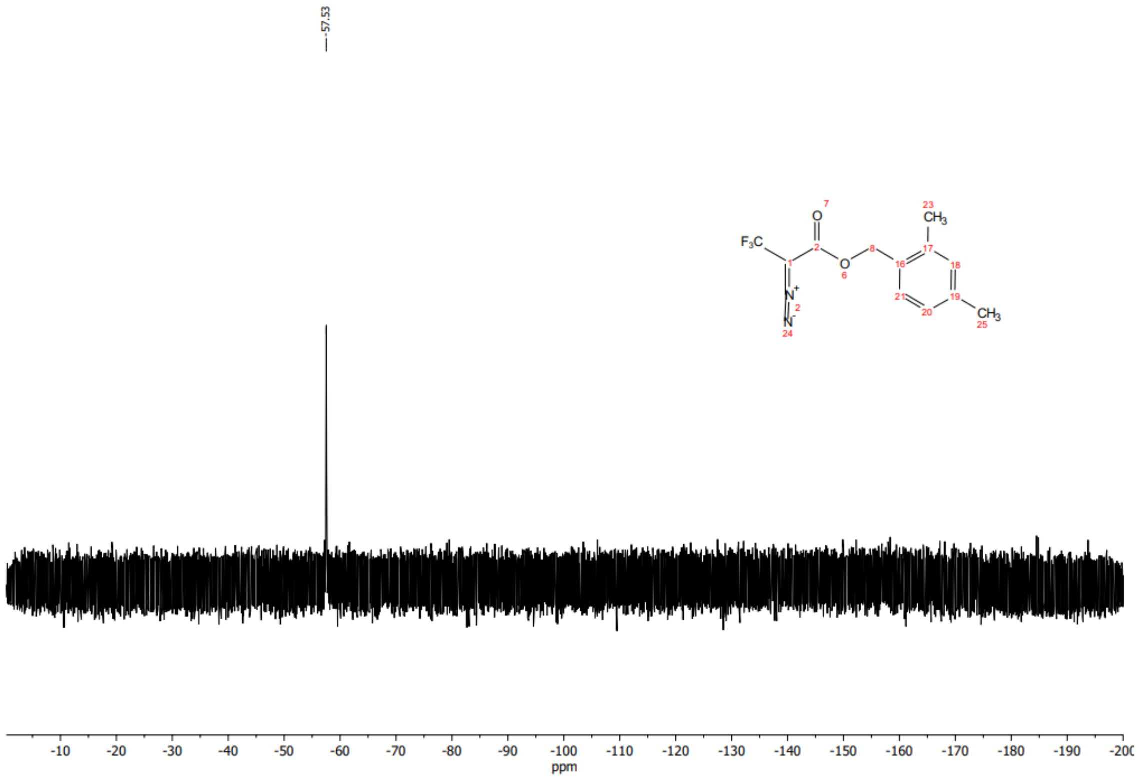

2,5-Dimethylbenzyl 2-diazo-3,3,3-trifluoropropanoate (**2g**), ^{1}H NMR (400 MHz, $CDCl_3$):

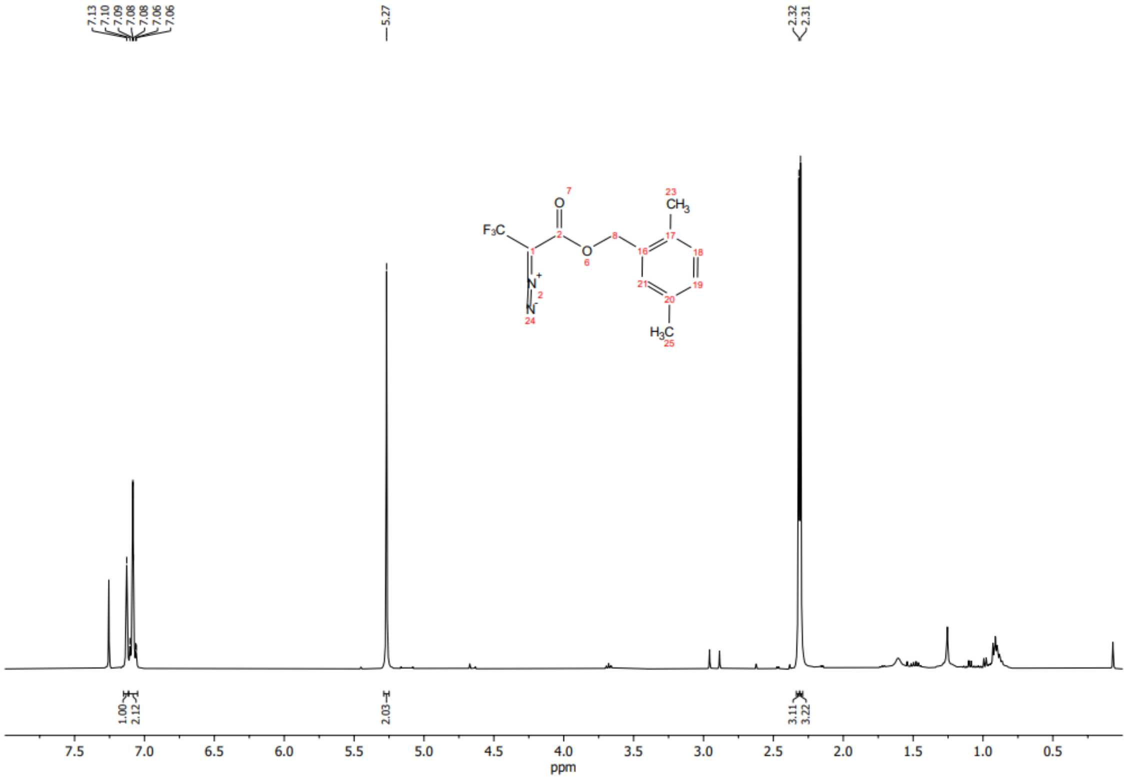

2,5-Dimethylbenzyl 2-diazo-3,3,3-trifluoropropanoate (**2g**), ^{13}C NMR (126 MHz, $CDCl_3$):

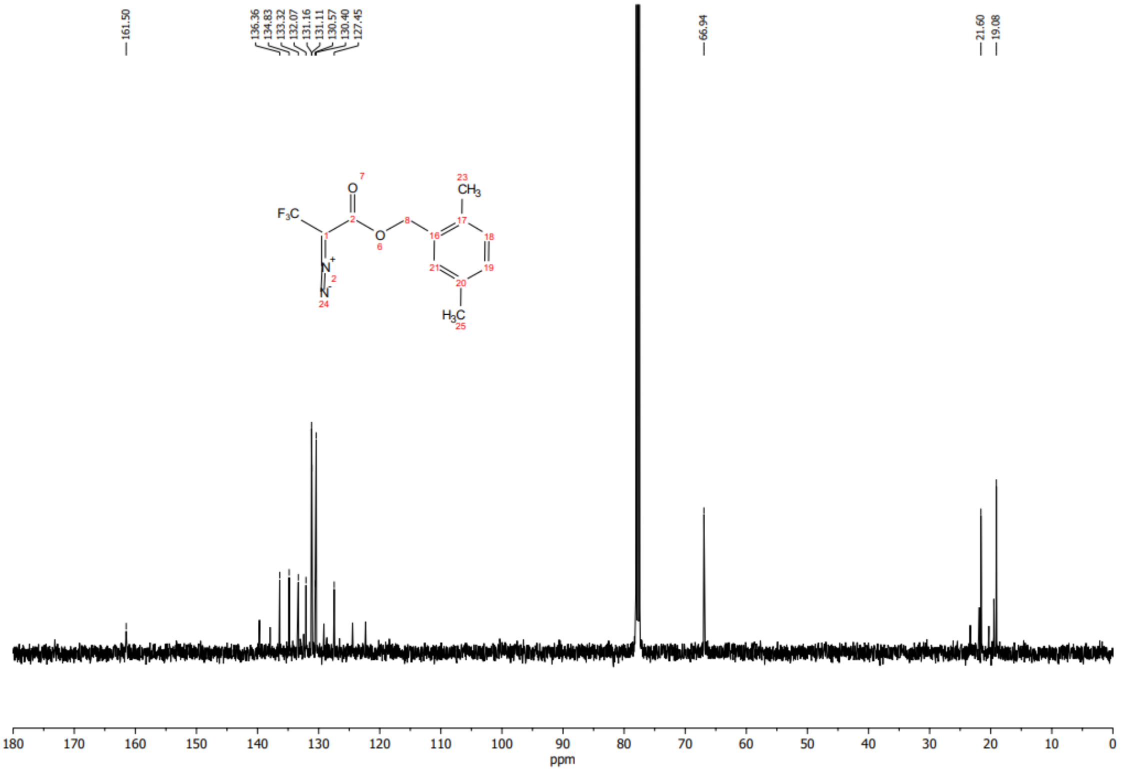

2,5-Dimethylbenzyl 2-diazo-3,3,3-trifluoropropanoate (**2g**), ^{19}F NMR (376 MHz, $CDCl_3$):

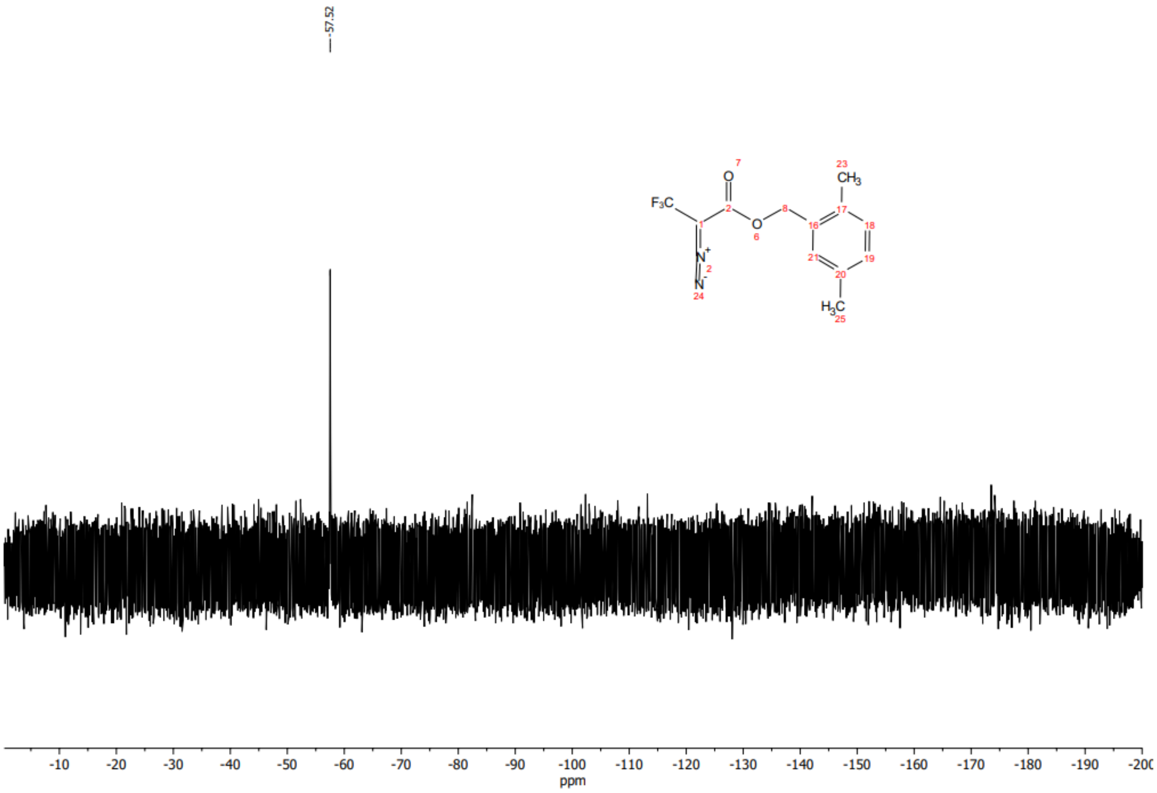

2,4,6-trimethylbenzyl 2-diazo-3,3,3-trifluoropropanoate (**2h**), ^{1}H NMR (400 MHz, $CDCl_3$):

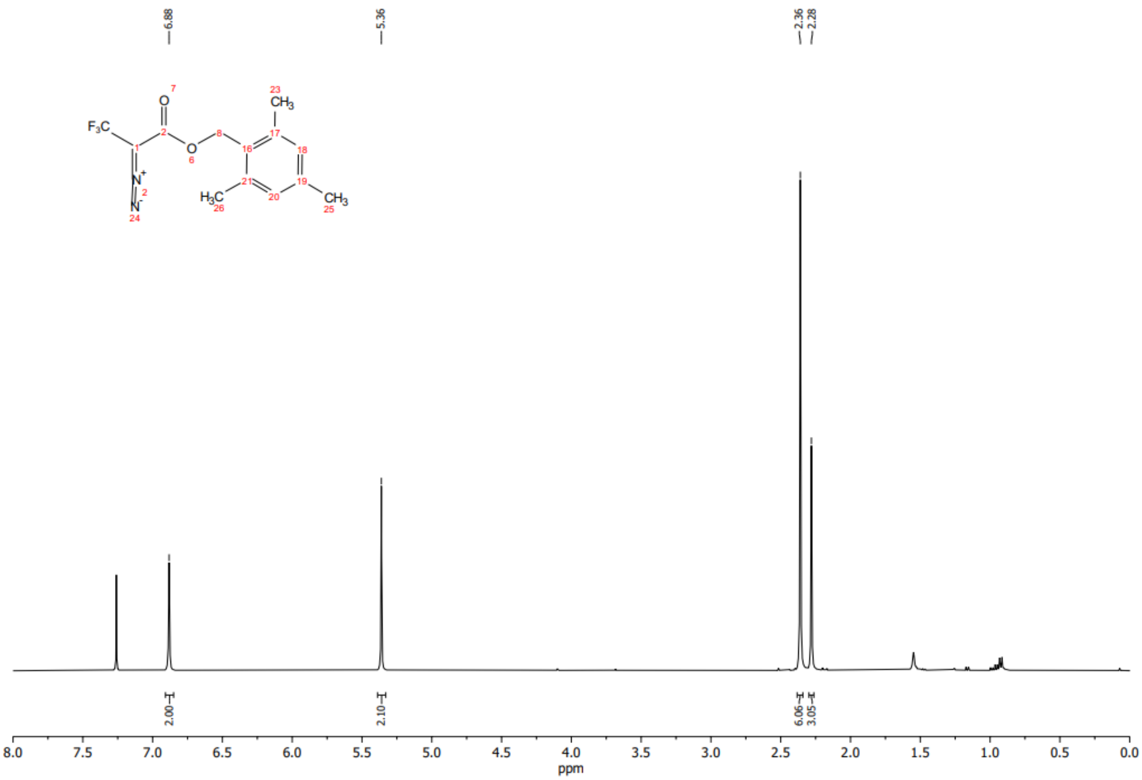

2,4,6-trimethylbenzyl 2-diazo-3,3,3-trifluoropropanoate (**2h**), ^{13}C NMR (126 MHz, $CDCl_3$):

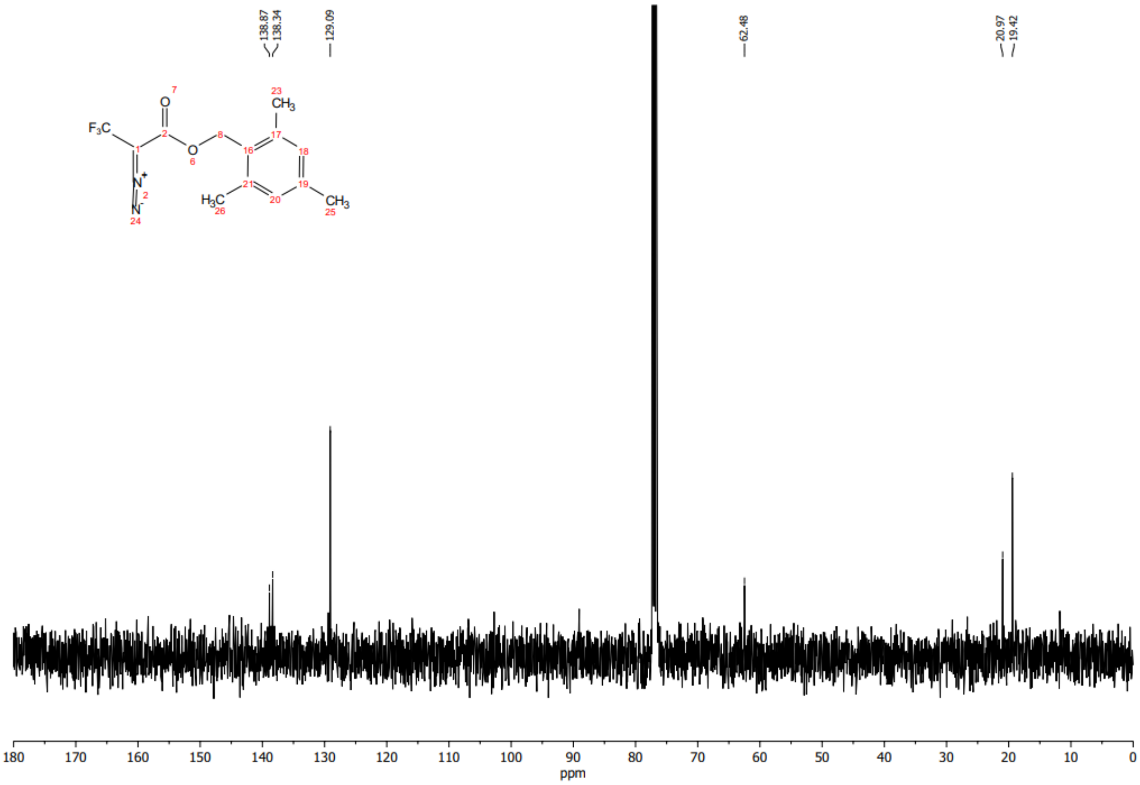

2,4,6-trimethylbenzyl 2-diazo-3,3,3-trifluoropropanoate (**2h**), ^{19}F NMR (376 MHz, $CDCl_3$):

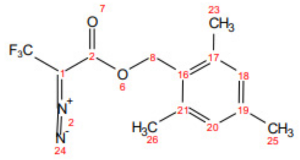

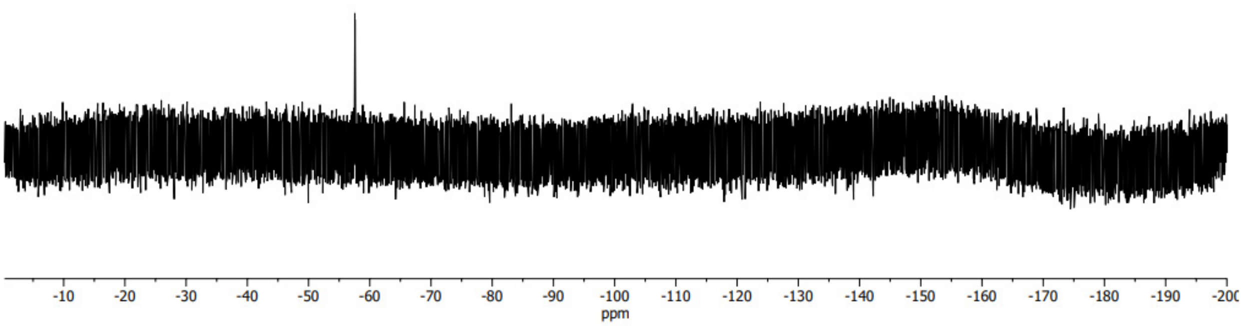

Ethyl 3,3,3-trifluoro-2-((4-methoxyphenyl)amino)propanoate (**3a**), ^{1}H NMR (400 MHz, $CDCl_3$):

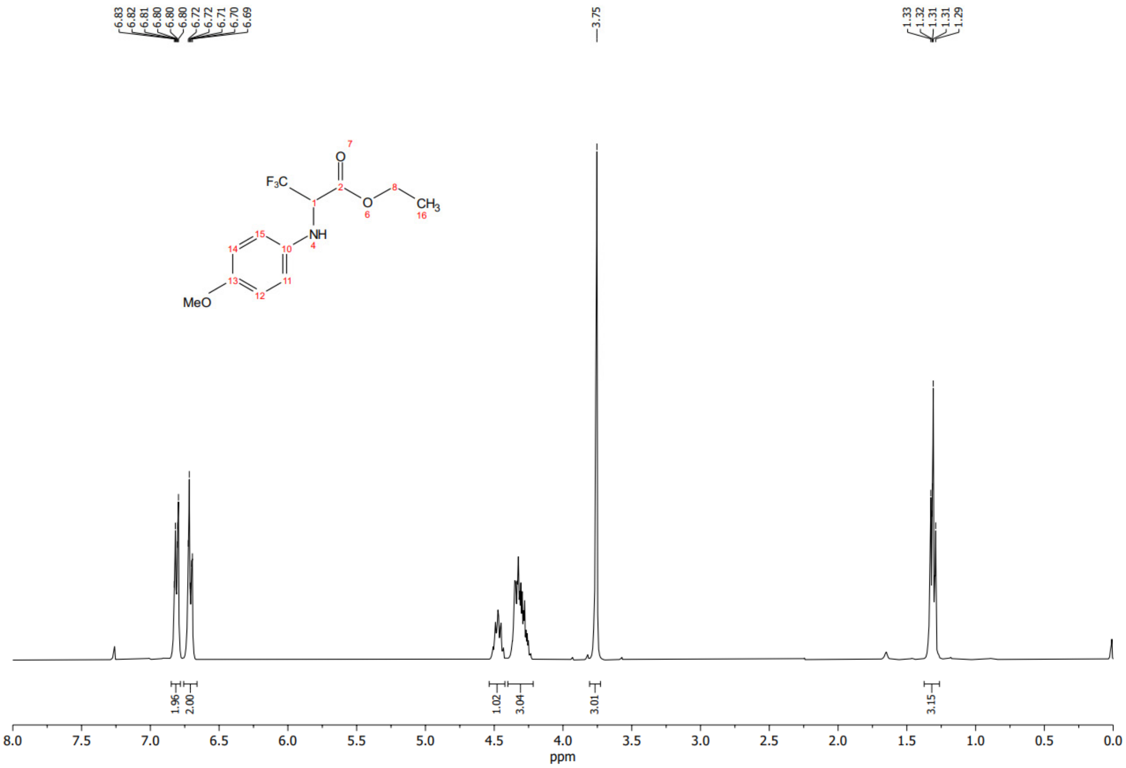

Ethyl 3,3,3-trifluoro-2-((4-methoxyphenyl)amino)propanoate (**3a**), ^{13}C NMR (126 MHz, $CDCl_3$):

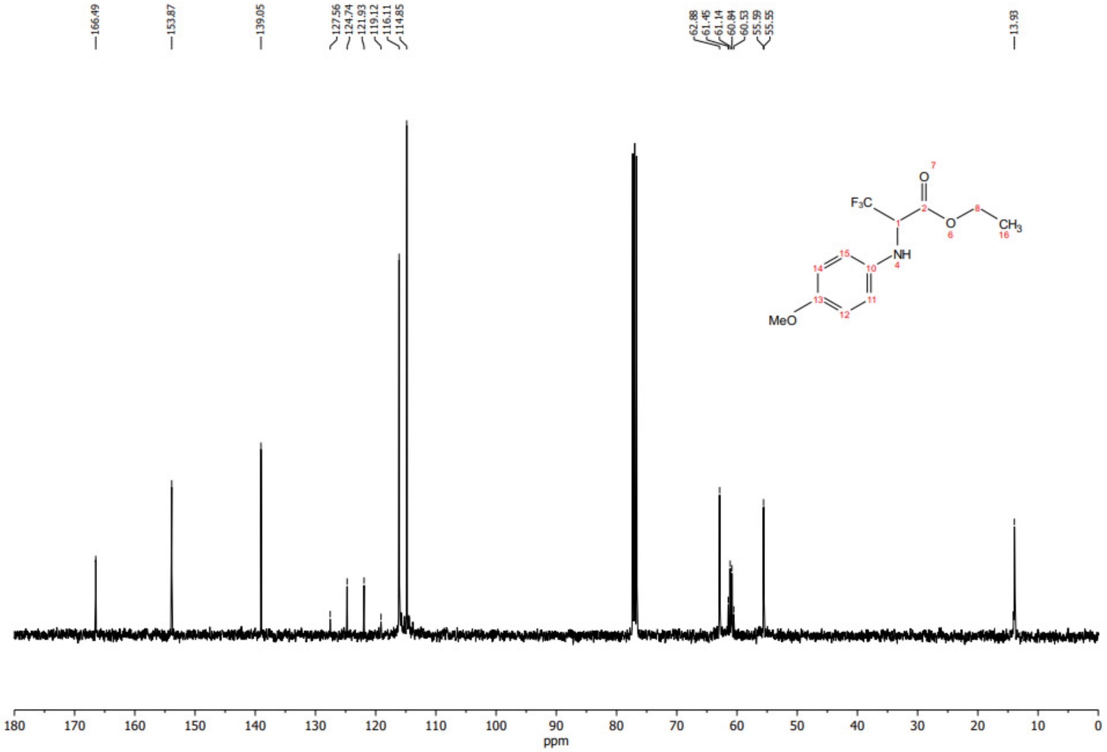

Ethyl 3,3,3-trifluoro-2-((4-methoxyphenyl)amino)propanoate (**3a**), ^{19}F NMR (376 MHz, $CDCl_3$):

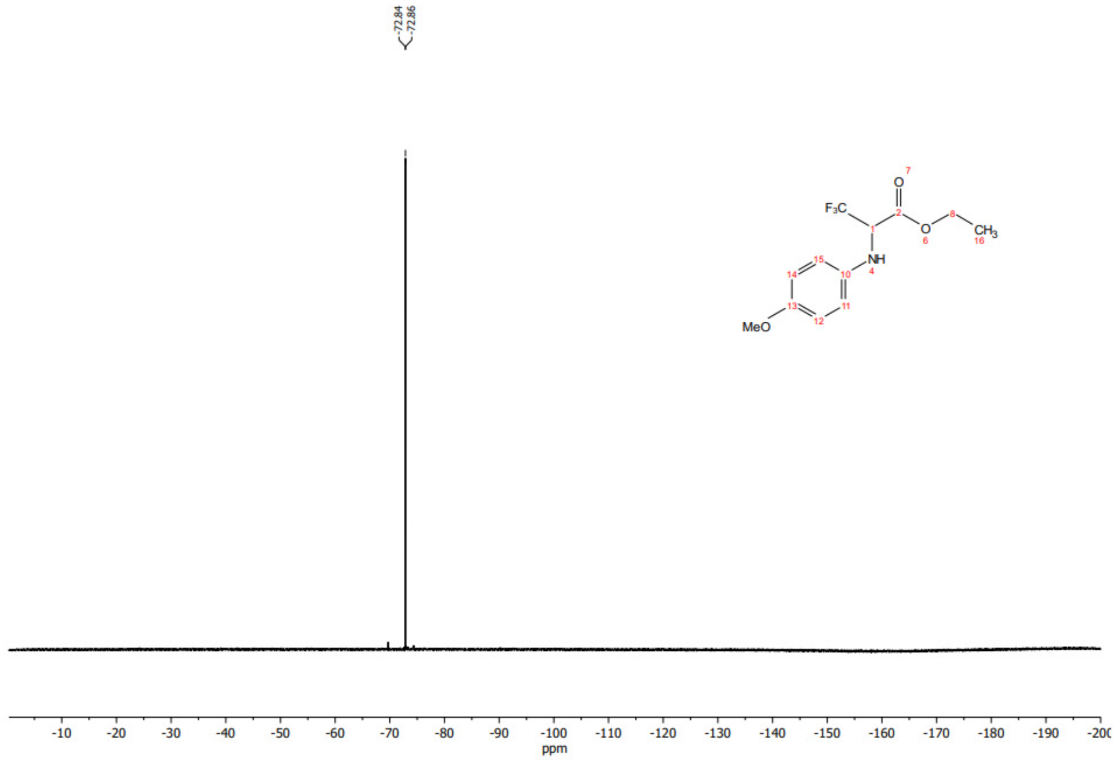

Cyclohexyl 3,3,3-trifluoro-2-((4-methoxyphenyl)amino)propanoate (**3b**), ^{1}H NMR (400 MHz, $CDCl_3$):

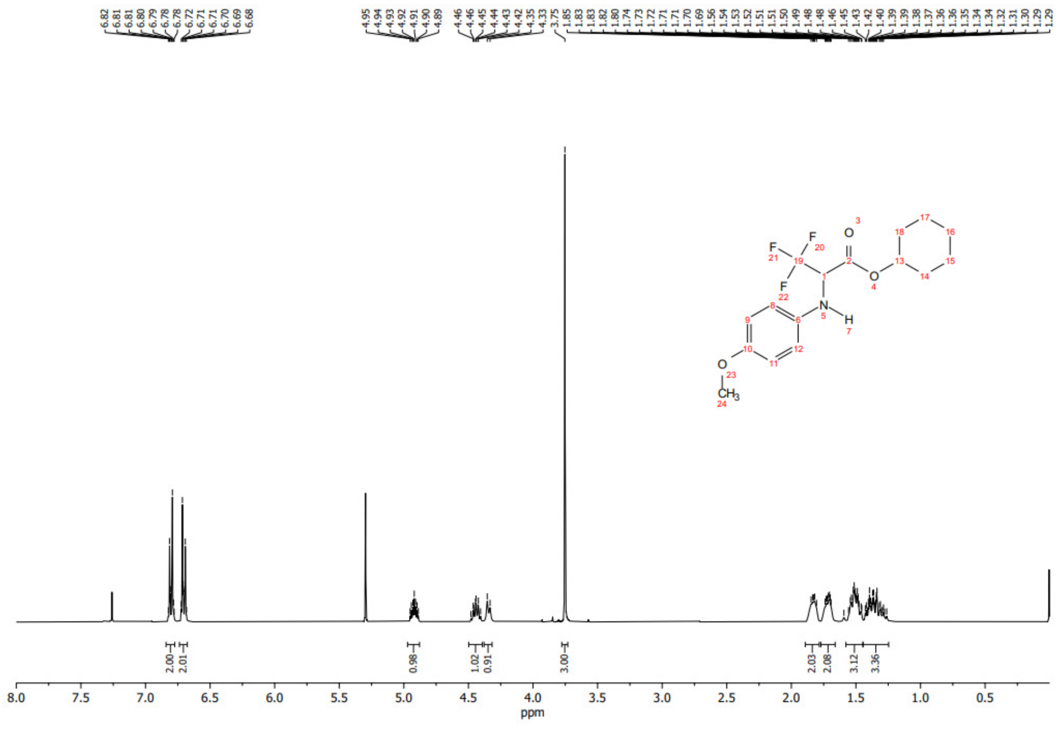

Cyclohexyl 3,3,3-trifluoro-2-((4-methoxyphenyl)amino)propanoate (**3b**), ^{13}C NMR (126 MHz, $CDCl_3$):

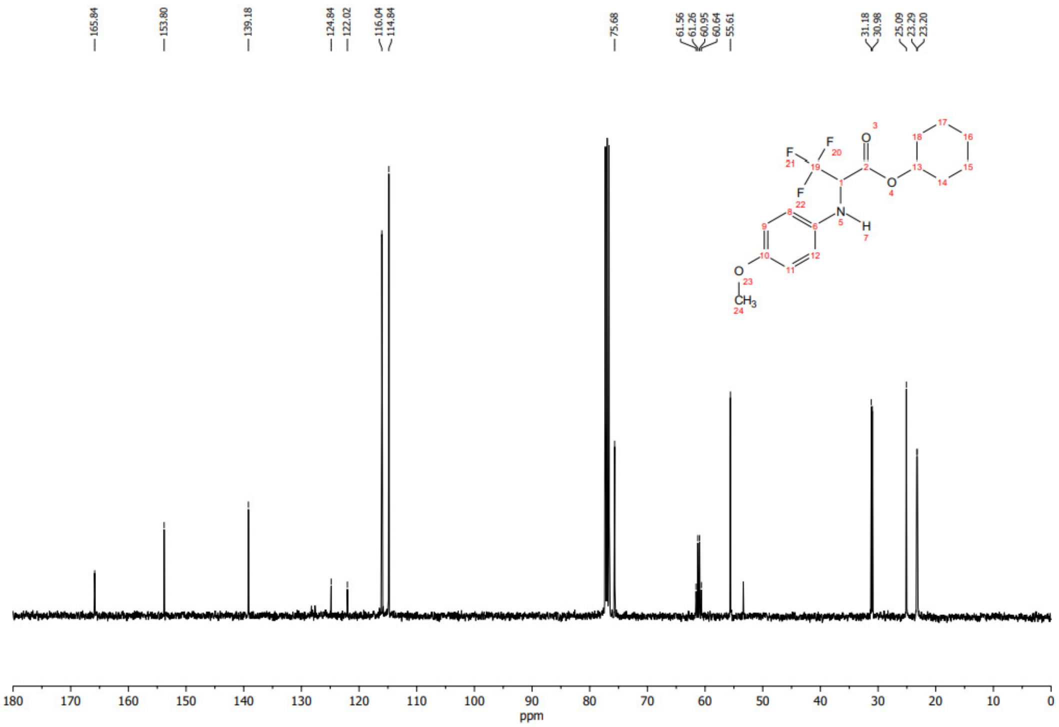

Cyclohexyl 3,3,3-trifluoro-2-((4-methoxyphenyl)amino)propanoate (**3b**), ^{19}F NMR (376 MHz, $CDCl_3$):

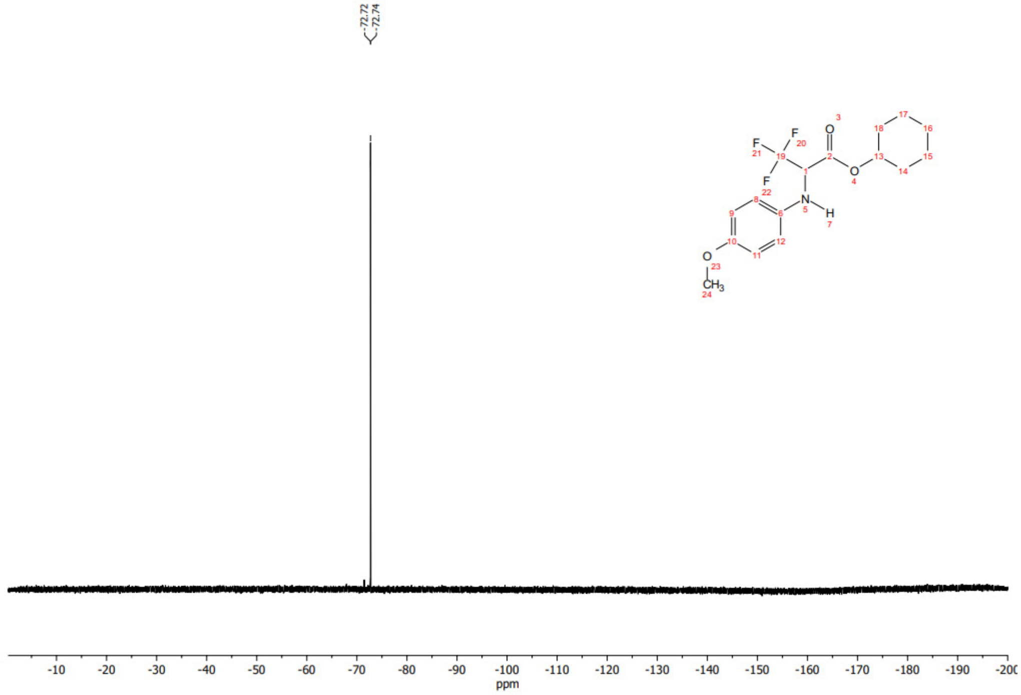

Naphthalen-1-ylmethyl 3,3,3-trifluoro-2-((4-methoxyphenyl)amino)propanoate (**3d**), ^{1}H NMR (400 MHz, $CDCl_3$):

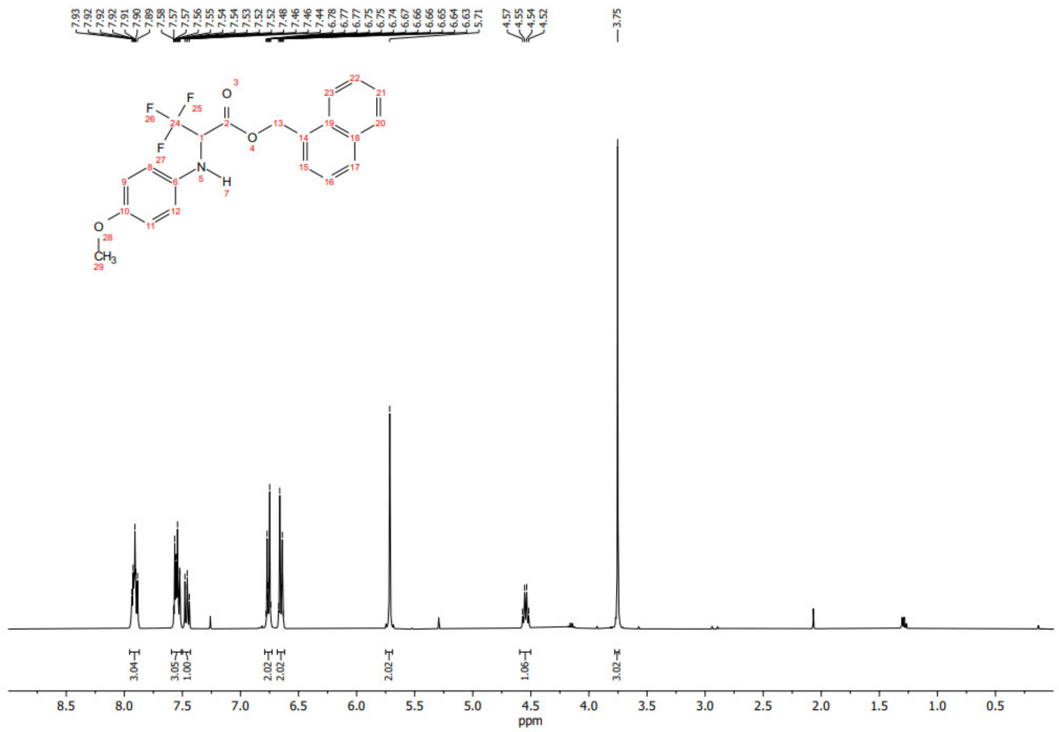

Naphthalen-1-ylmethyl 3,3,3-trifluoro-2-((4-methoxyphenyl)amino)propanoate (**3d**), ^{13}C NMR (126 MHz, $CDCl_3$):

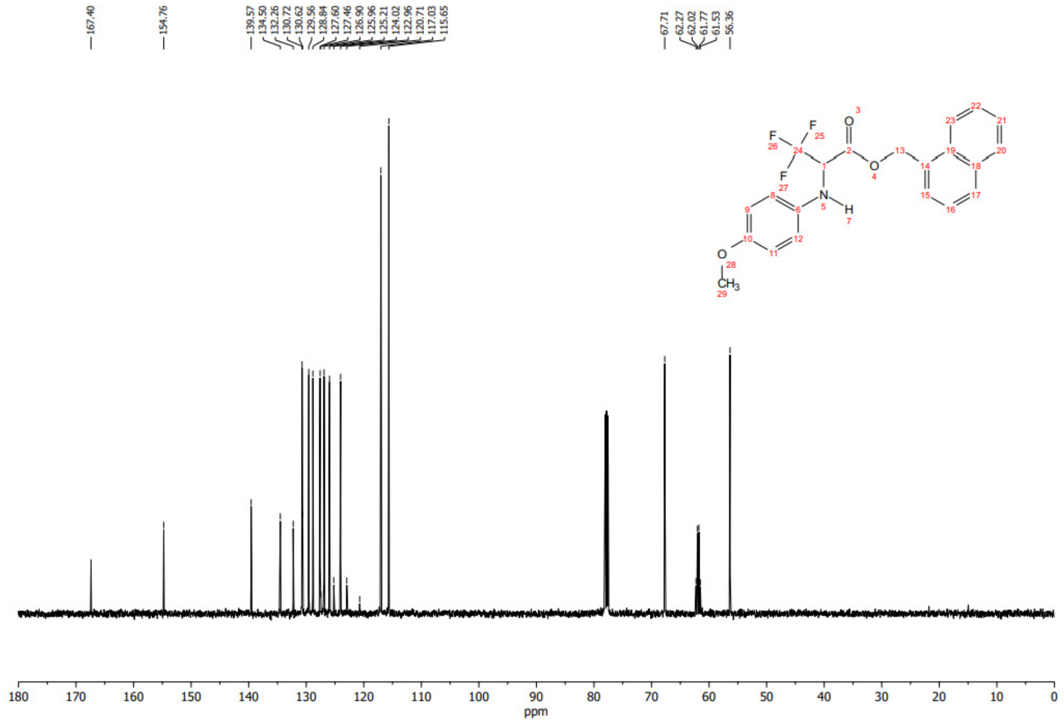

Naphthalen-1-ylmethyl 3,3,3-trifluoro-2-((4-methoxyphenyl)amino)propanoate (**3d**), ^{19}F NMR (376 MHz, $CDCl_3$):

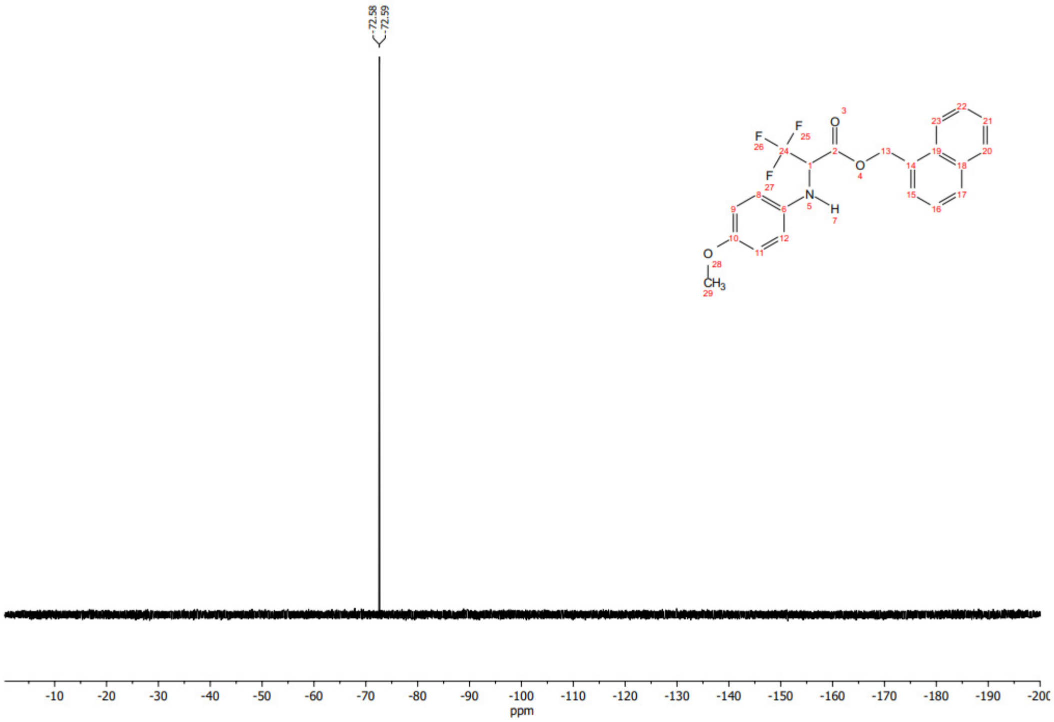

2-Methylbenzyl 3,3,3-trifluoro-2-((4-methoxyphenyl)amino)propanoate (**3e**), ^{1}H NMR (400 MHz, $CDCl_3$):

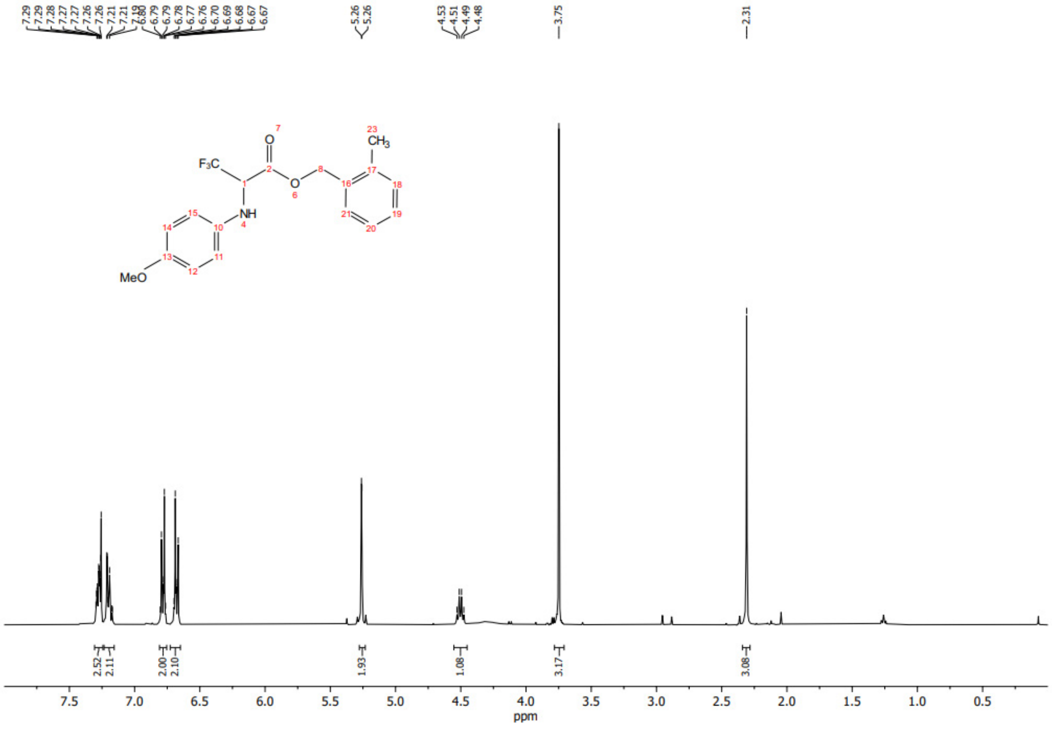

2-Methylbenzyl 3,3,3-trifluoro-2-((4-methoxyphenyl)amino)propanoate (**3e**), ^{13}C NMR (126 MHz, $CDCl_3$):

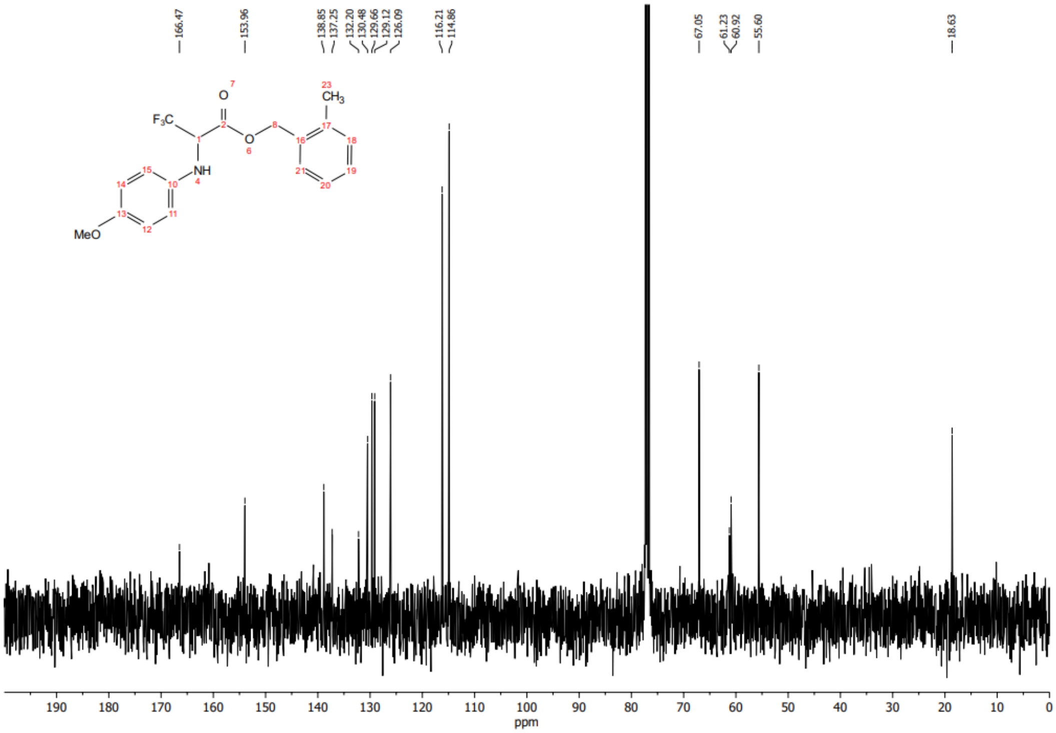

2-Methylbenzyl 3,3,3-trifluoro-2-((4-methoxyphenyl)amino)propanoate (**3e**), ^{19}F NMR (376 MHz, $CDCl_3$):

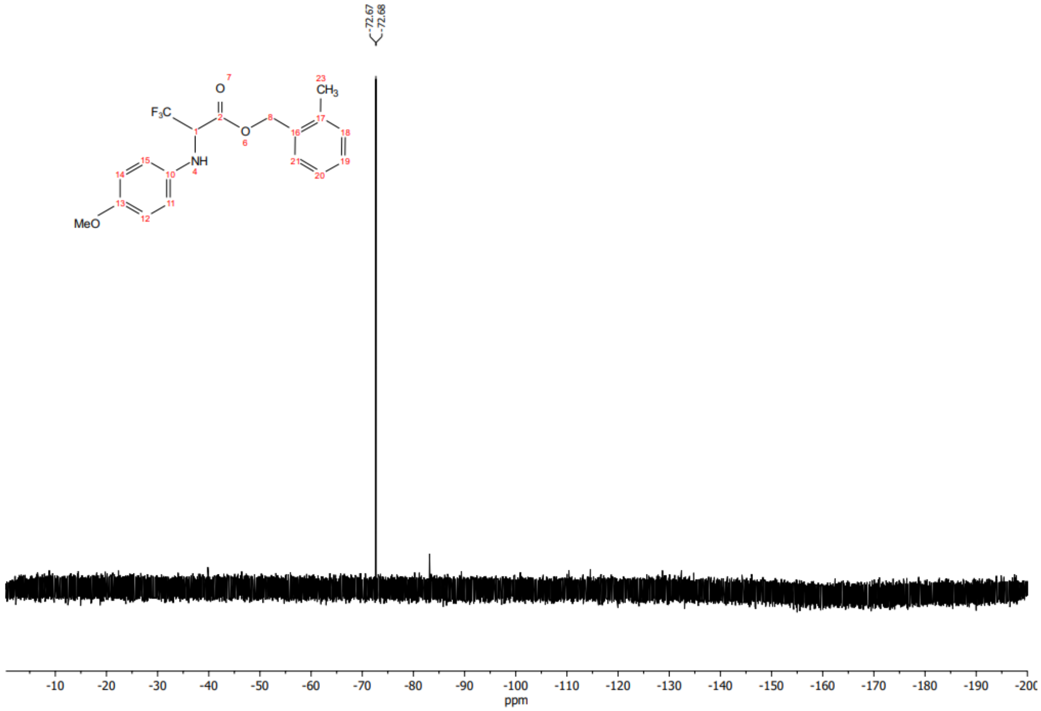

2,4-Dimethylbenzyl 3,3,3-trifluoro-2-((4-methoxyphenyl)amino)propanoate (**3f**), ^{1}H NMR (400 MHz, $CDCl_3$):

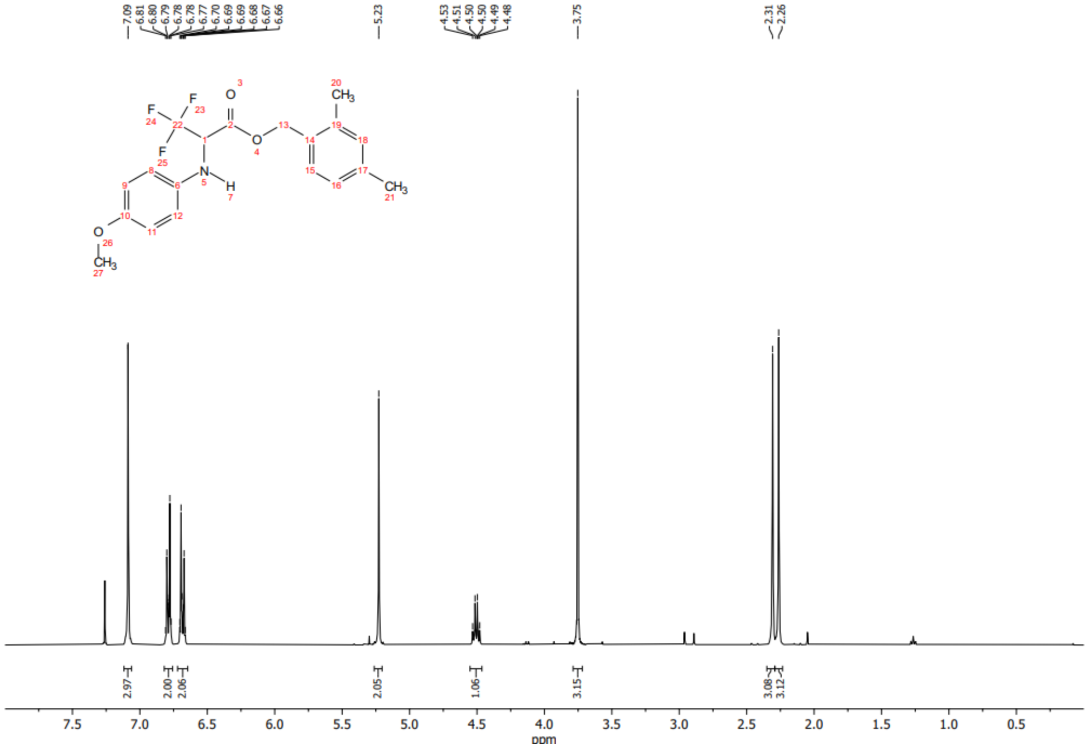

2,4-Dimethylbenzyl 3,3,3-trifluoro-2-((4-methoxyphenyl)amino)propanoate (**3f**), ^{13}C NMR (126 MHz, $CDCl_3$):

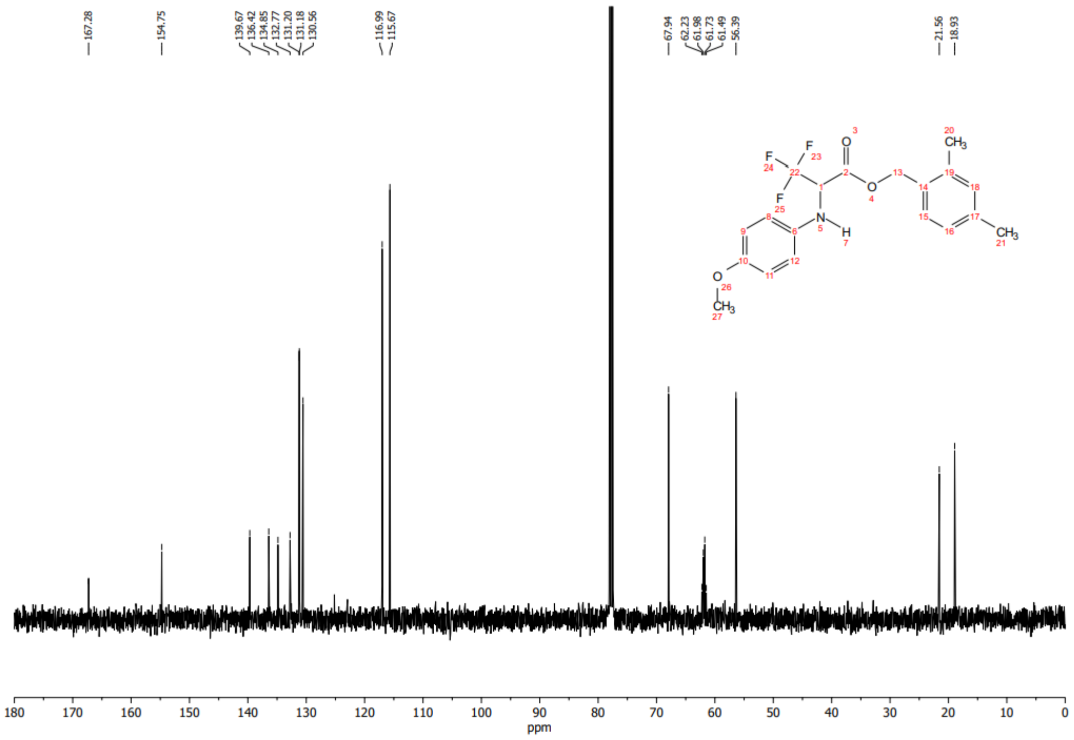

2,4-Dimethylbenzyl 3,3,3-trifluoro-2-((4-methoxyphenyl)amino)propanoate (**3f**), ^{19}F NMR (376 MHz, $CDCl_3$):

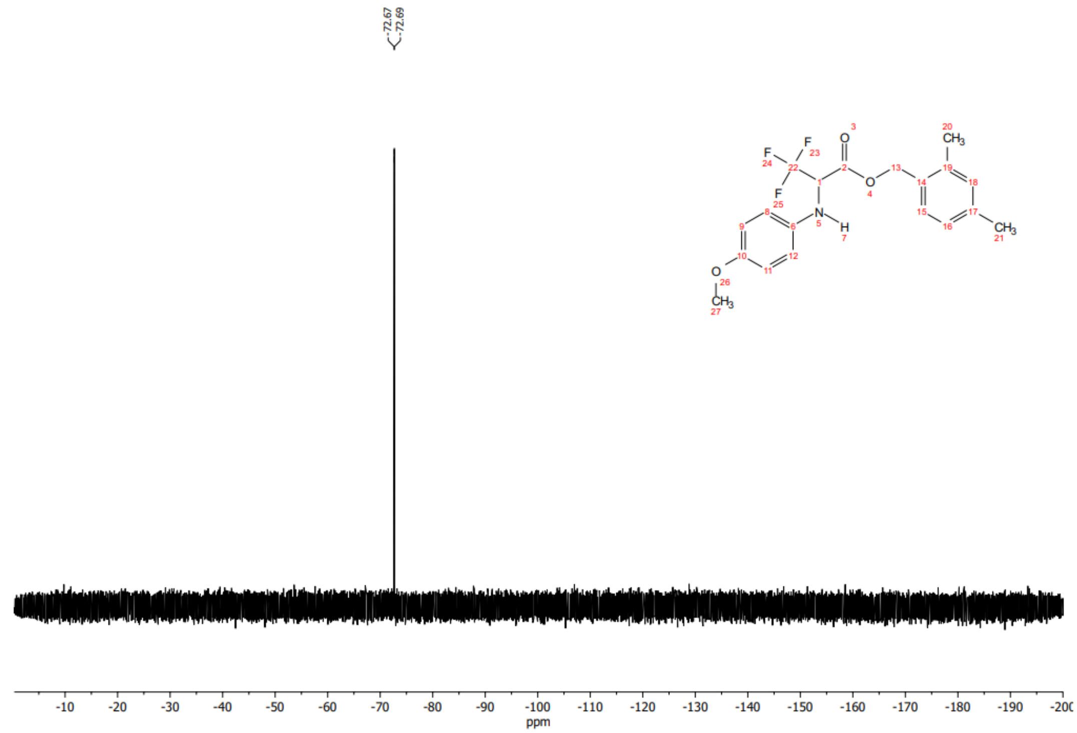

2,5-Dimethylbenzyl 3,3,3-trifluoro-2-((4-methoxyphenyl)amino)propanoate (**3g**), ^{1}H NMR (400 MHz, $CDCl_3$):

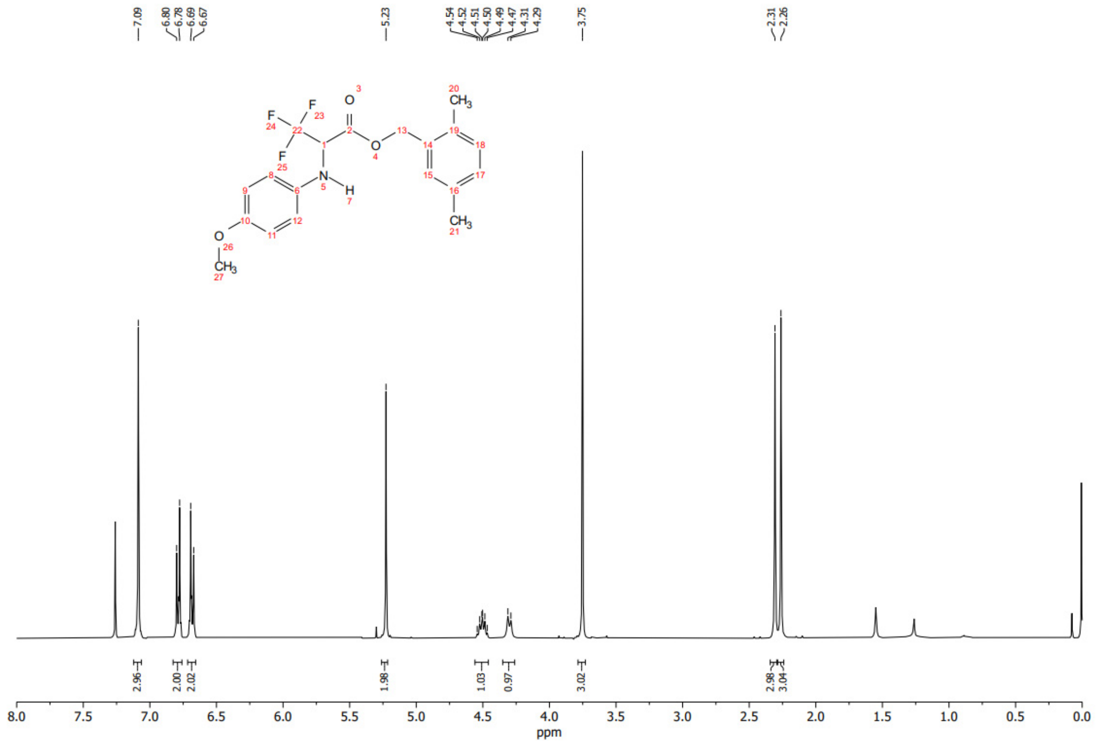

2,5-Dimethylbenzyl 3,3,3-trifluoro-2-((4-methoxyphenyl)amino)propanoate (**3g**), ^{13}C NMR (126 MHz, $CDCl_3$):

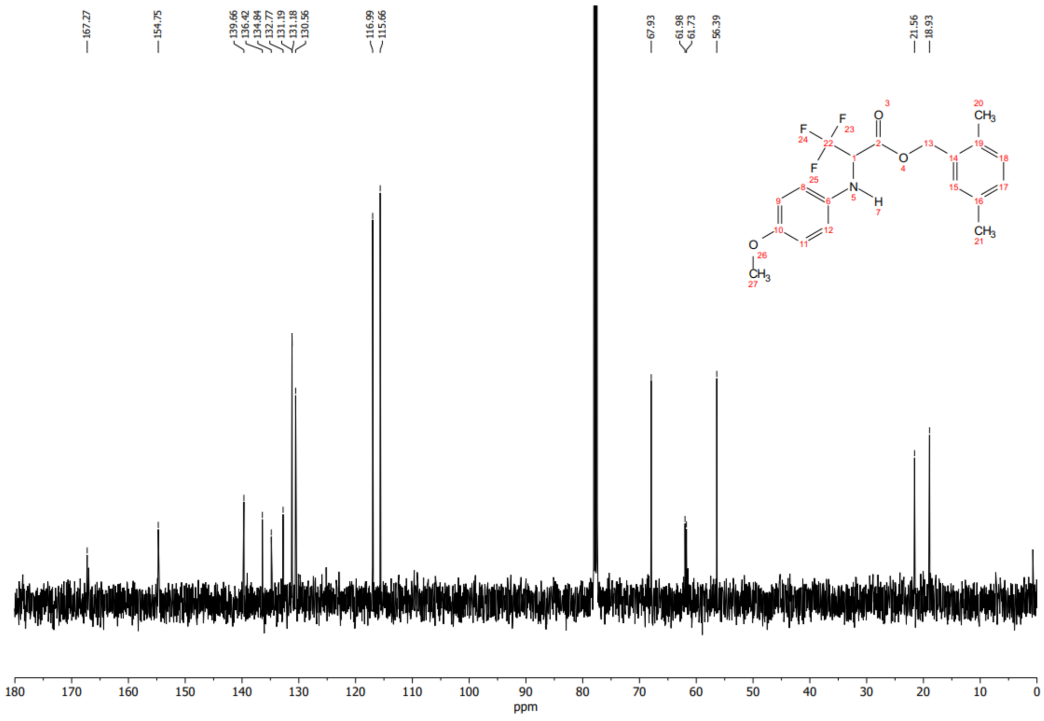

2,5-Dimethylbenzyl 3,3,3-trifluoro-2-((4-methoxyphenyl)amino)propanoate (**3g**), ^{19}F NMR (376 MHz, $CDCl_3$):

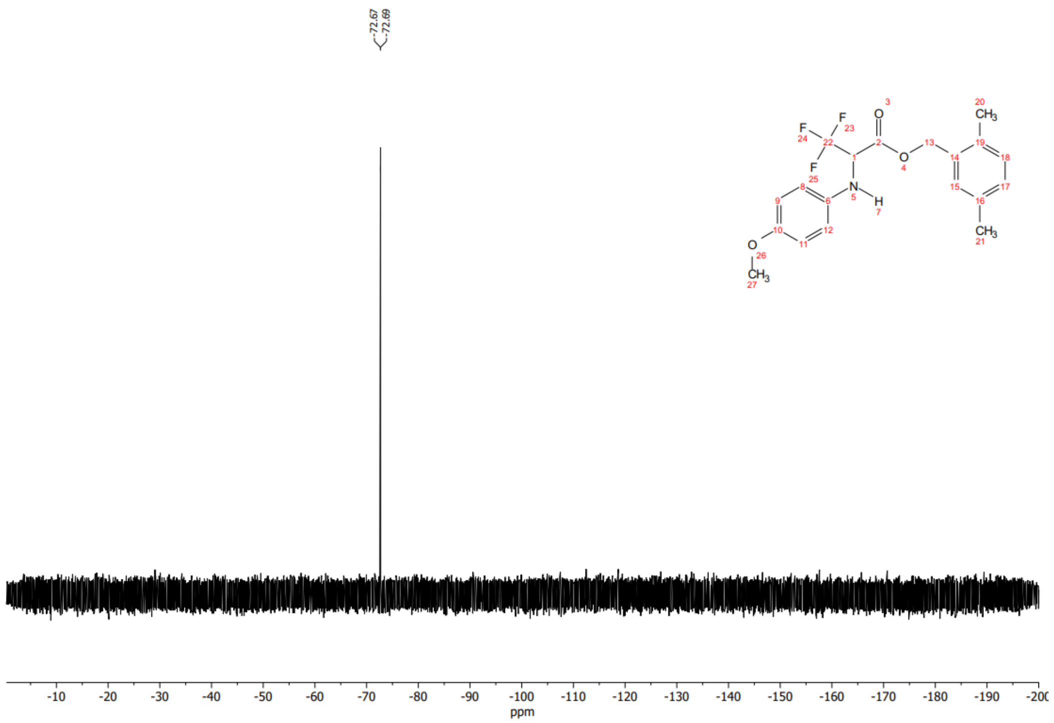

2,4,6-trimethylbenzyl 3,3,3-trifluoro-2-((4-methoxyphenyl)amino)propanoate (**3h**), ^{1}H NMR (400 MHz, $CDCl_3$):

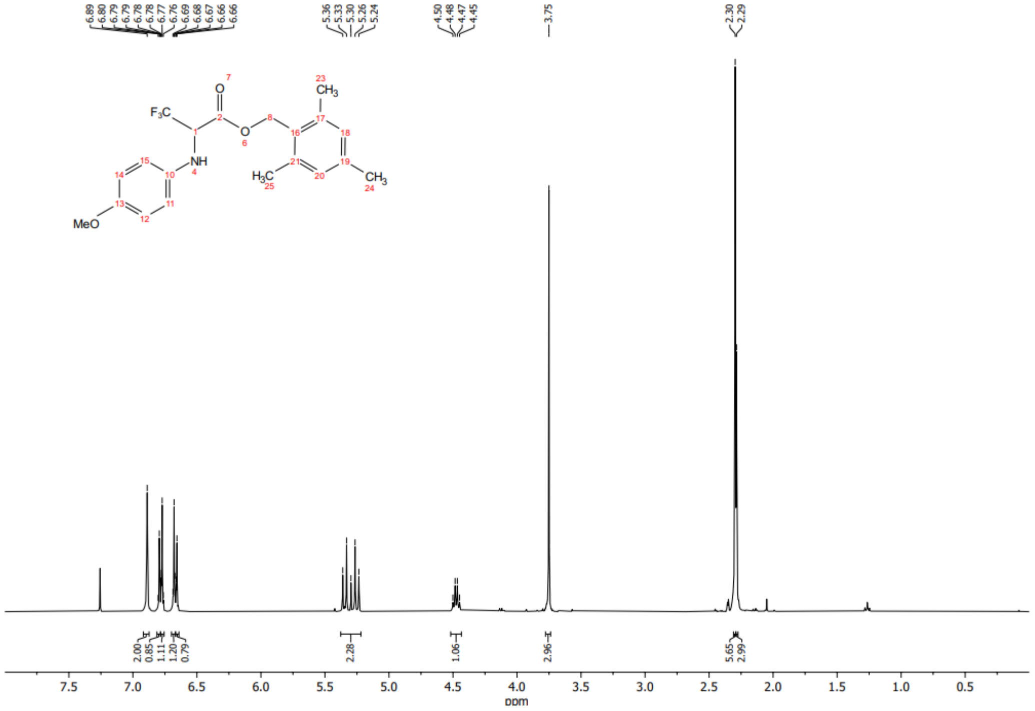

2,4,6-trimethylbenzyl 3,3,3-trifluoro-2-((4-methoxyphenyl)amino)propanoate (**3h**), ^{13}C NMR (126 MHz, $CDCl_3$):

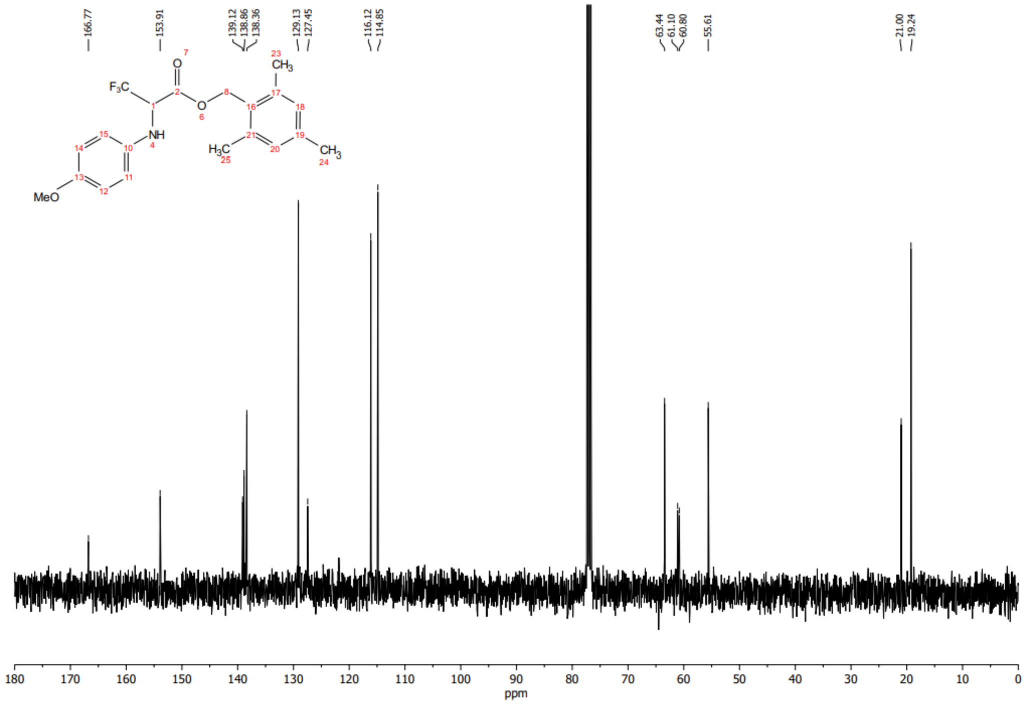

2,4,6-trimethylbenzyl 3,3,3-trifluoro-2-((4-methoxyphenyl)amino)propanoate (**3h**), ^{19}F NMR (376 MHz, $CDCl_3$):

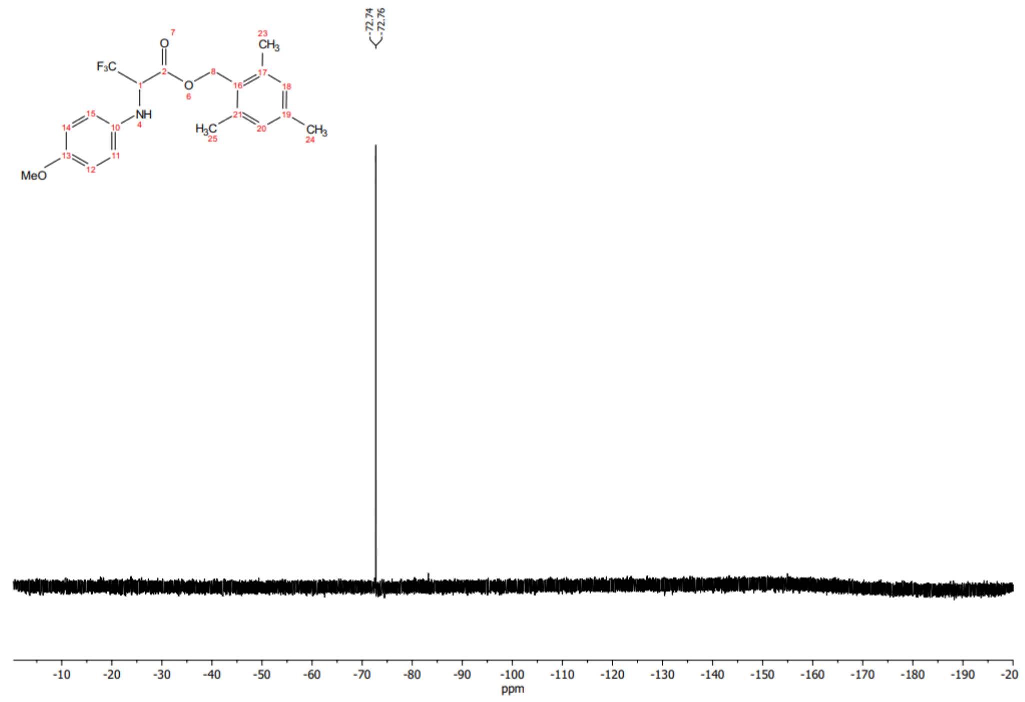

Benzyl 3,3,3-trifluoro-2-(*p*-tolylamino)propanoate (**4b**), ^{1}H NMR (400 MHz, $CDCl_3$):

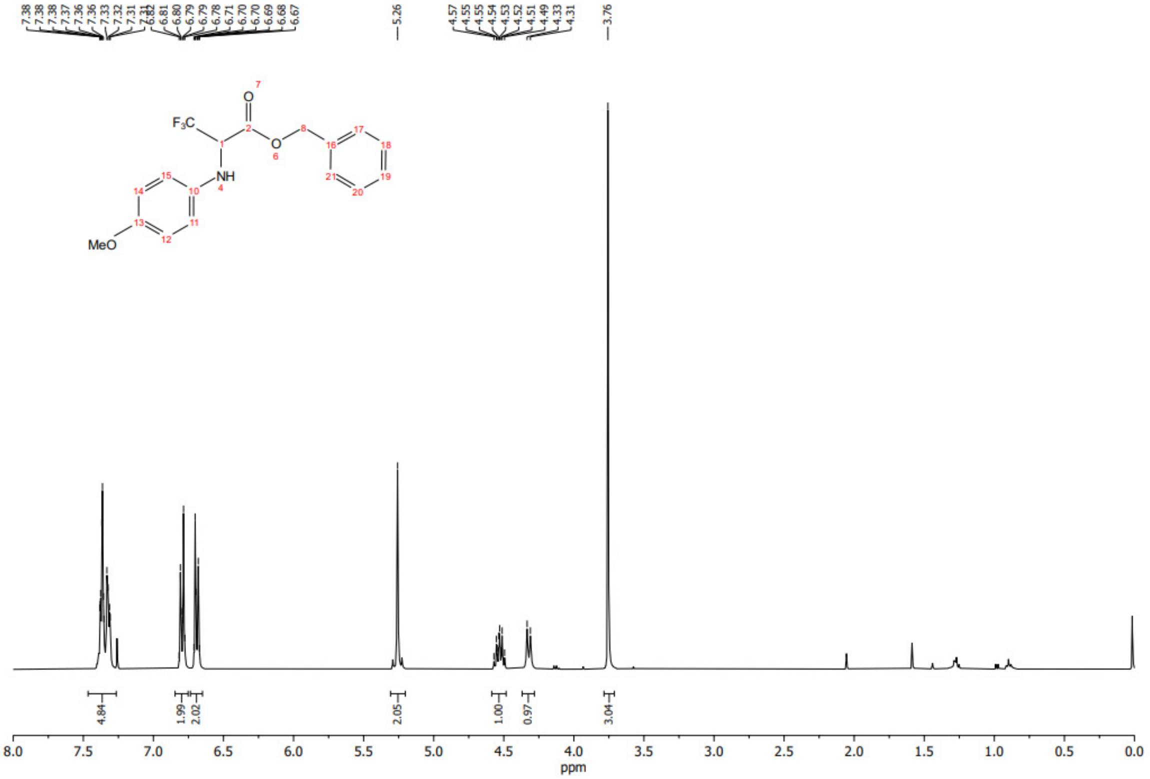

Benzyl 3,3,3-trifluoro-2-(*p*-tolylamino)propanoate (**4b**), ^{13}C NMR (126 MHz, $CDCl_3$):

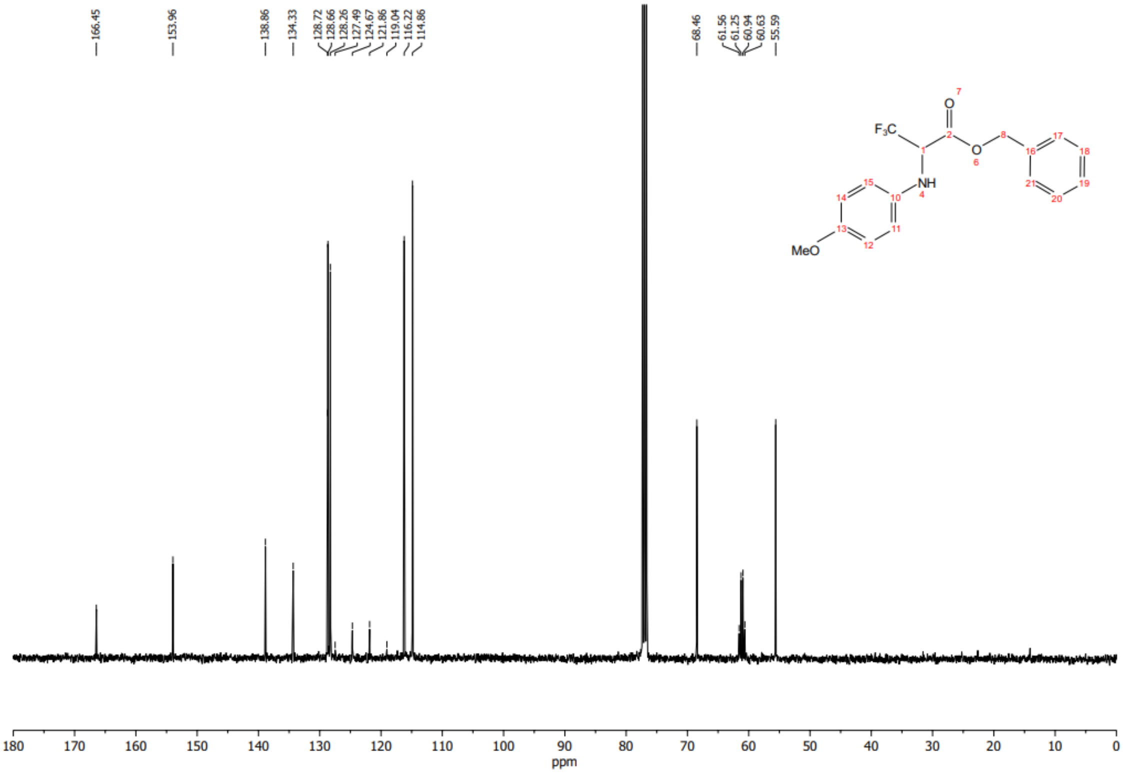

Benzyl 3,3,3-trifluoro-2-(*p*-tolylamino)propanoate (**4b**), ^{19}F NMR (376 MHz, $CDCl_3$):

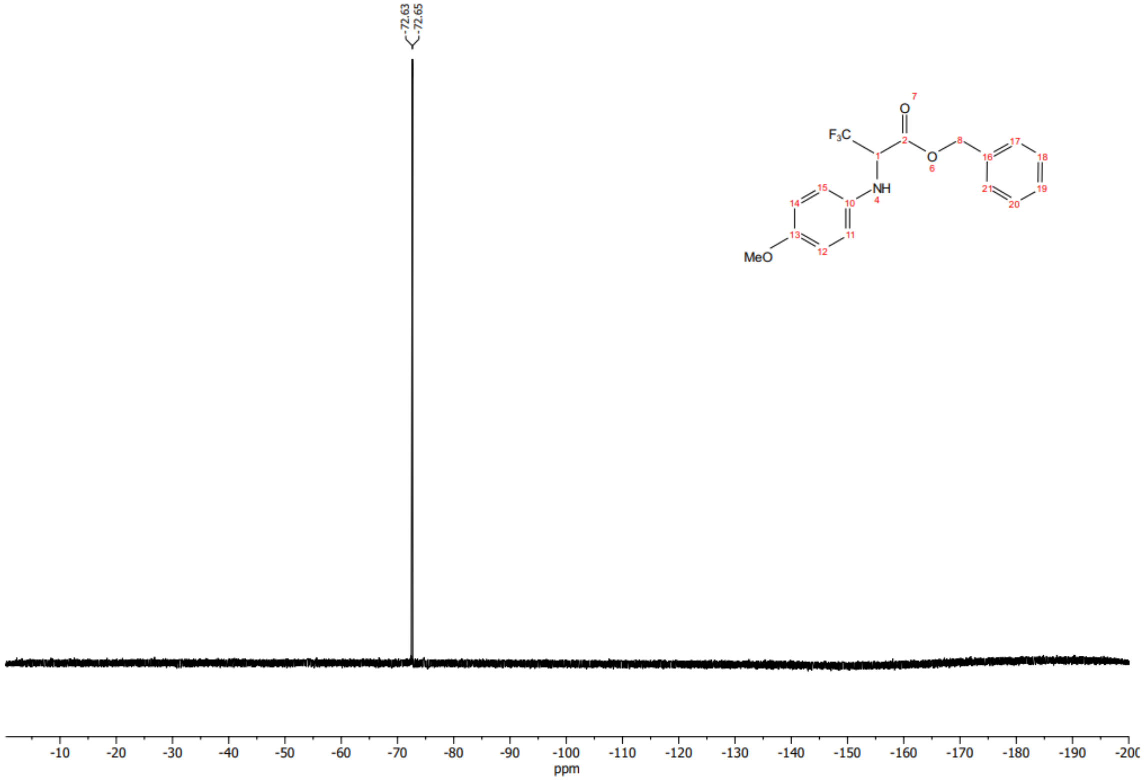

Benzyl 3,3,3-trifluoro-2-(*m*-tolylamino)propanoate (**4c**), ^{1}H NMR (400 MHz, $CDCl_3$):

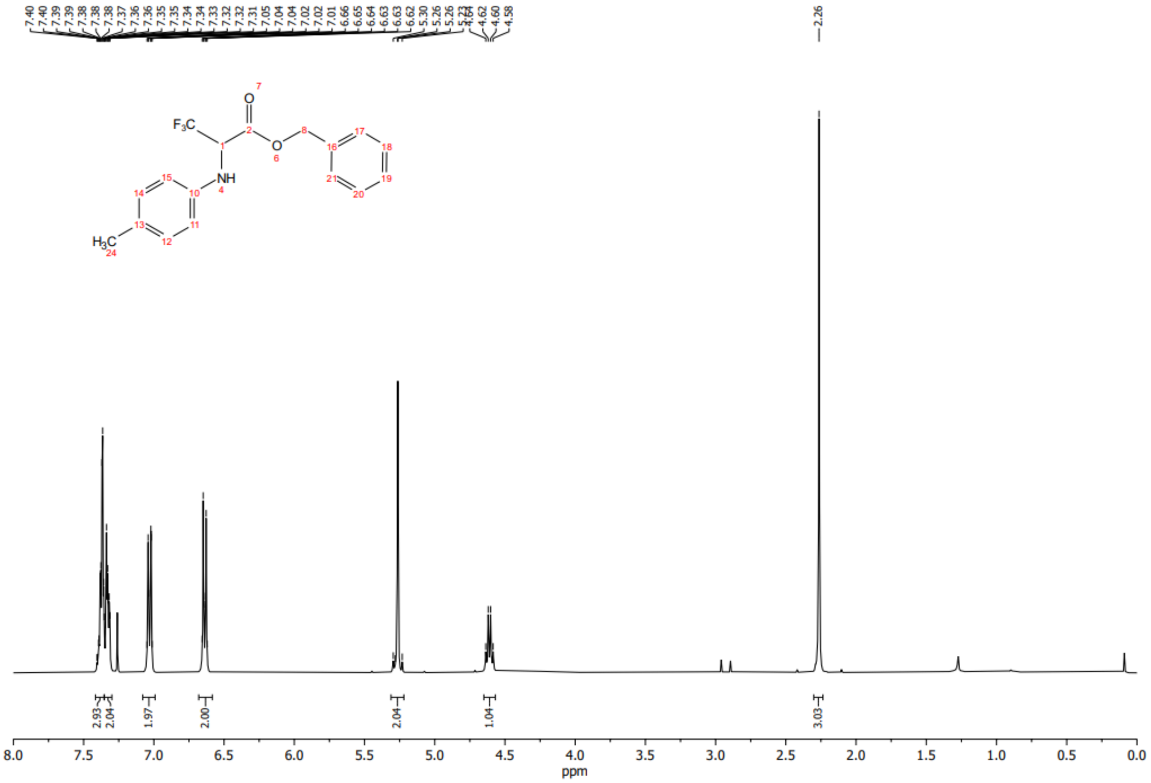

Benzyl 3,3,3-trifluoro-2-(*m*-tolylamino)propanoate (**4c**), ^{13}C NMR (126 MHz, $CDCl_3$):

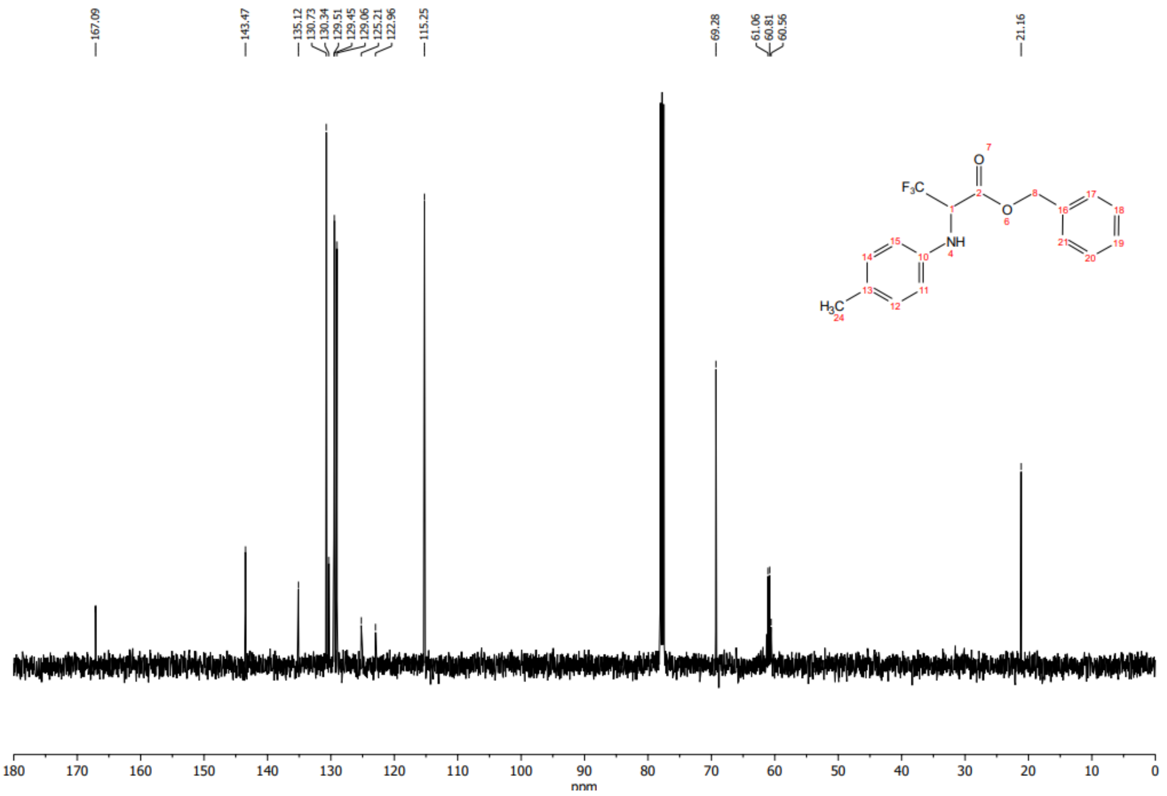

Benzyl 3,3,3-trifluoro-2-(*m*-tolylamino)propanoate (**4c**), ^{19}F NMR (376 MHz, $CDCl_3$):

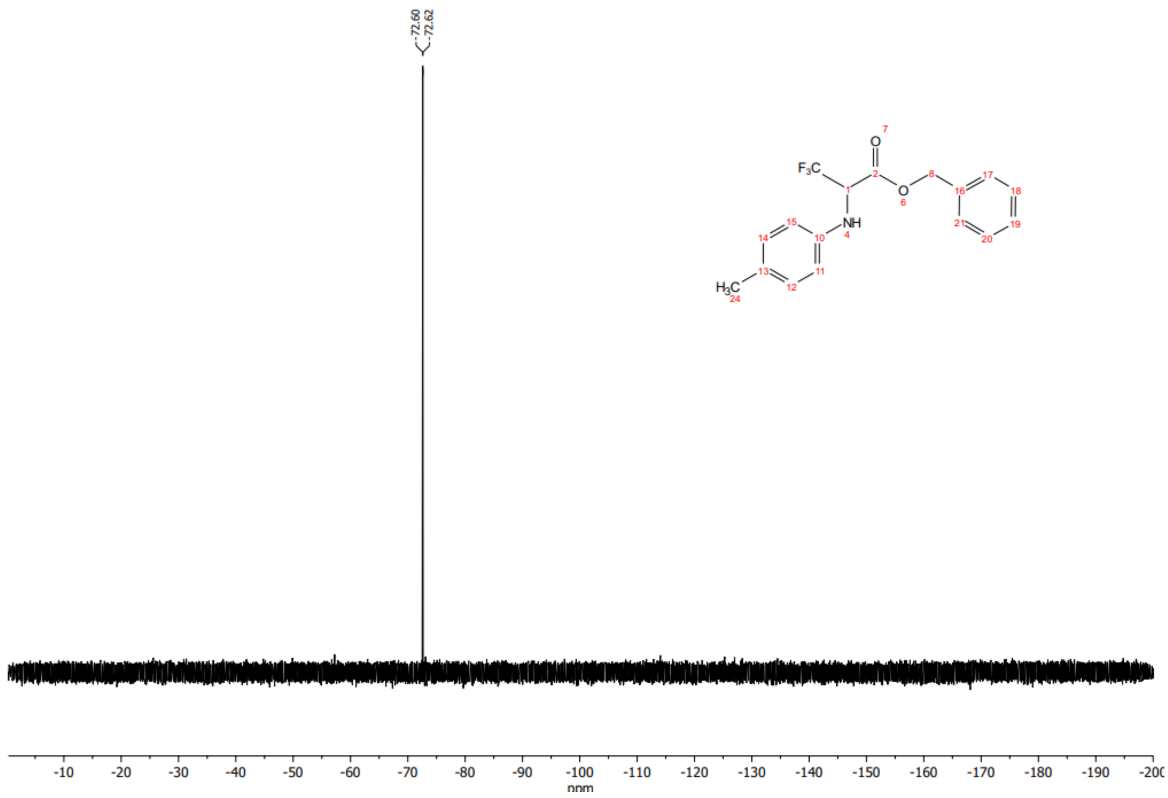

Benzyl 3,3,3-trifluoro-2-(*o*-tolylamino)propanoate (**4d**), ^{1}H NMR (400 MHz, $CDCl_3$):

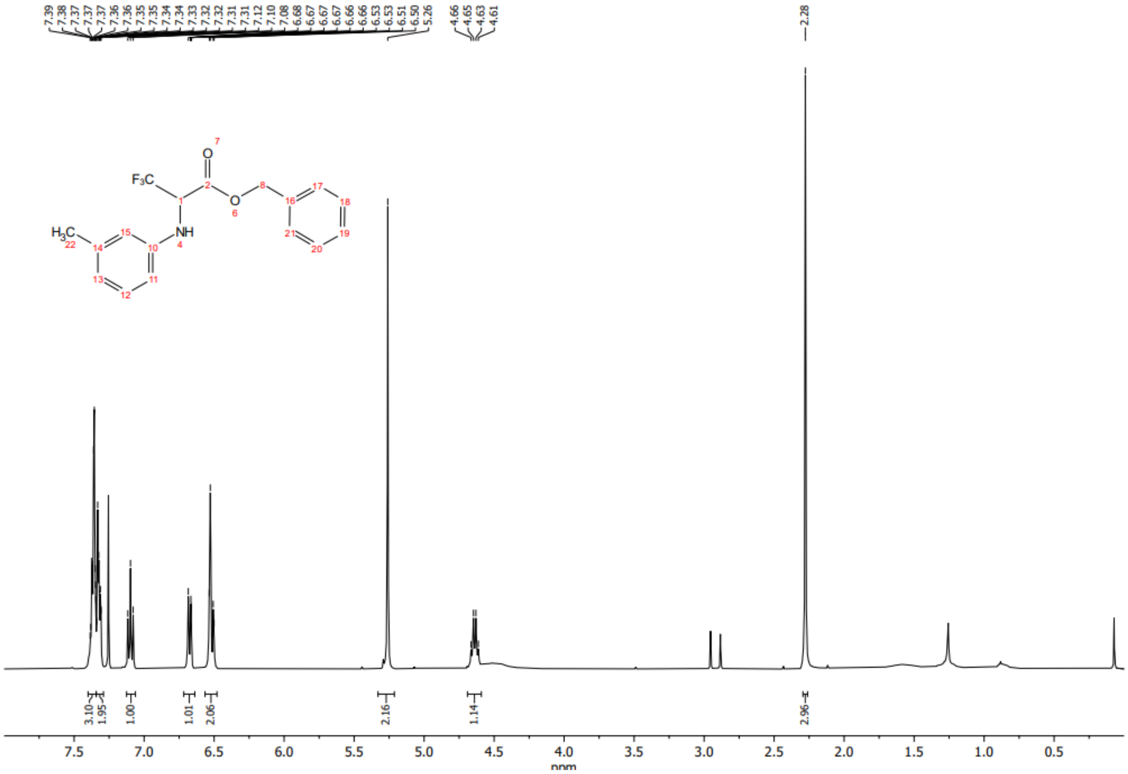

Benzyl 3,3,3-trifluoro-2-(*o*-tolylamino)propanoate (**4d**), ^{13}C NMR (126 MHz, $CDCl_3$):

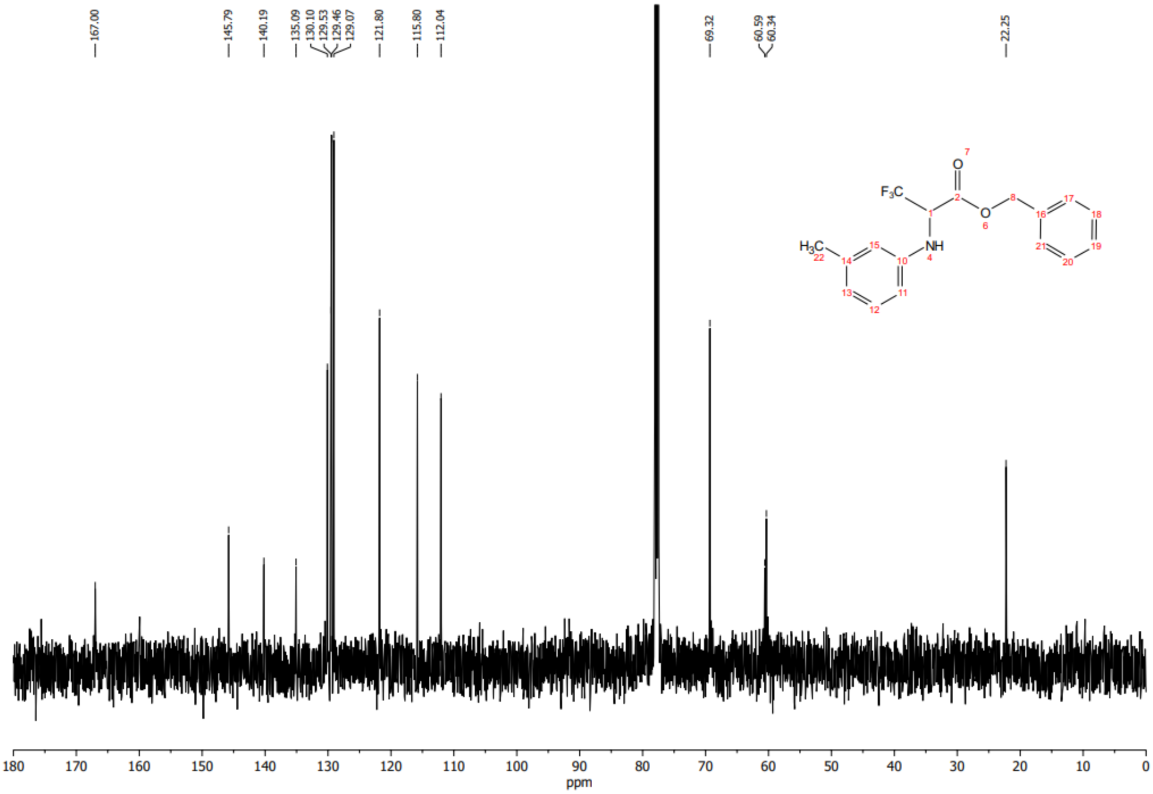

Benzyl 3,3,3-trifluoro-2-(*o*-tolylamino)propanoate (**4d**), ^{19}F NMR (376 MHz, $CDCl_3$):

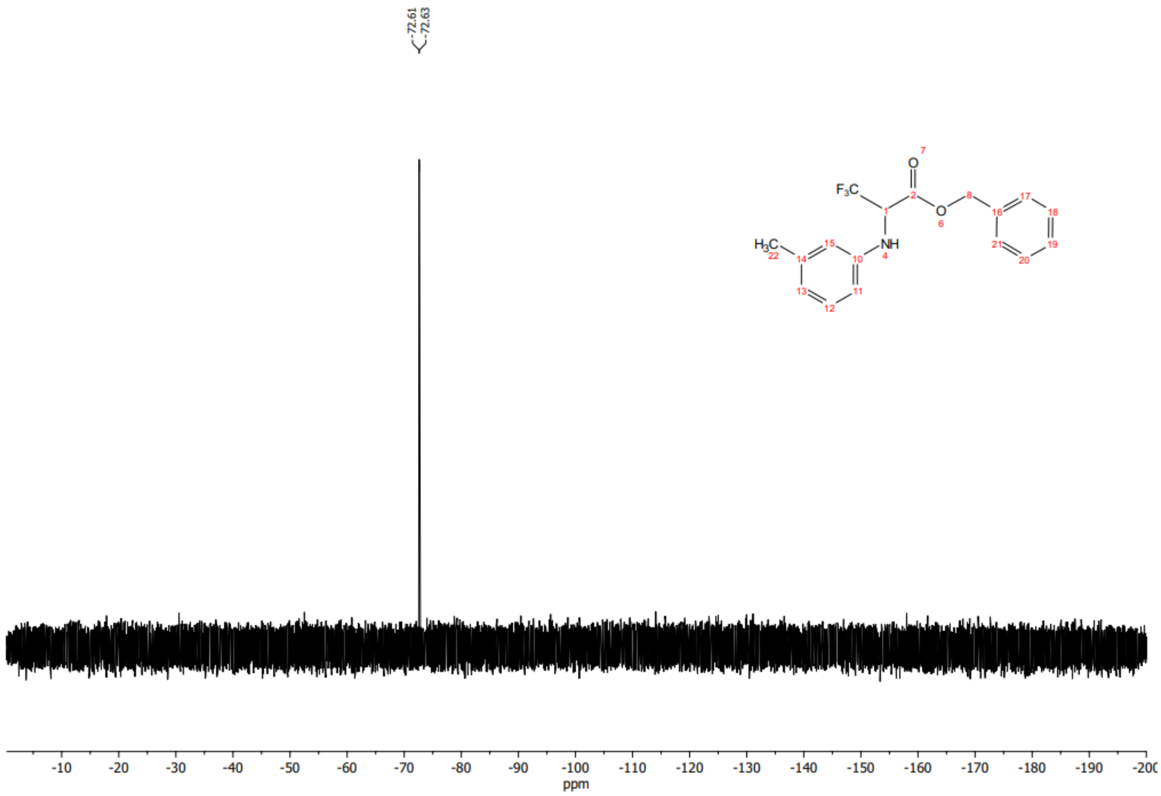

Benzyl 2-((4-chlorophenyl)amino)-3,3,3-trifluoropropanoate (**4e**), ^{1}H NMR (400 MHz, $CDCl_3$):

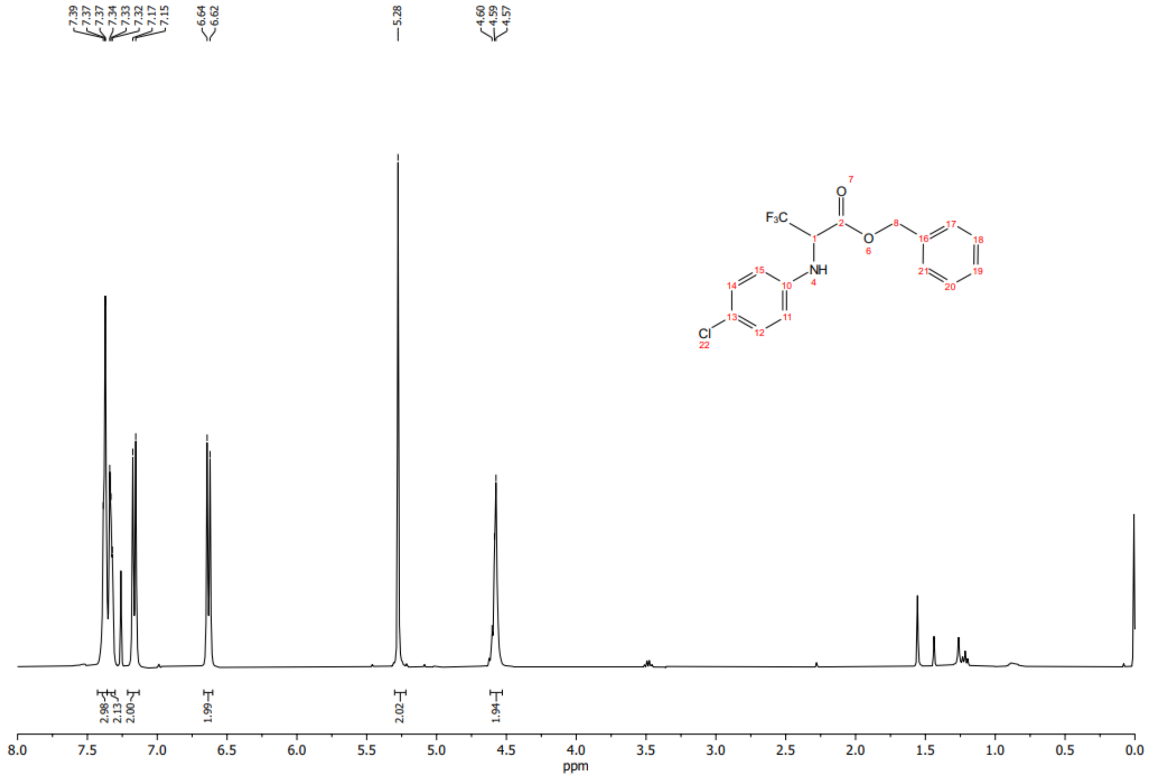

Benzyl 2-((4-chlorophenyl)amino)-3,3,3-trifluoropropanoate (**4e**), ^{13}C NMR (126 MHz, $CDCl_3$):

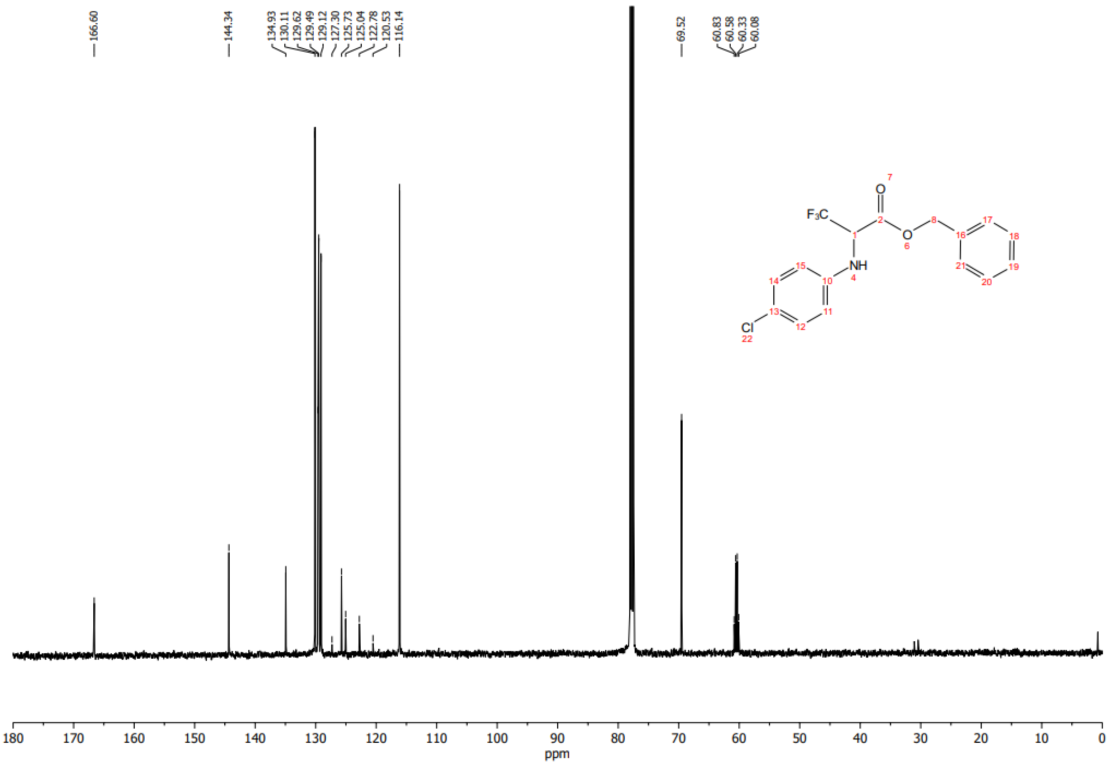

Benzyl 2-((4-chlorophenyl)amino)-3,3,3-trifluoropropanoate (**4e**), ^{19}F NMR (376 MHz, $CDCl_3$):

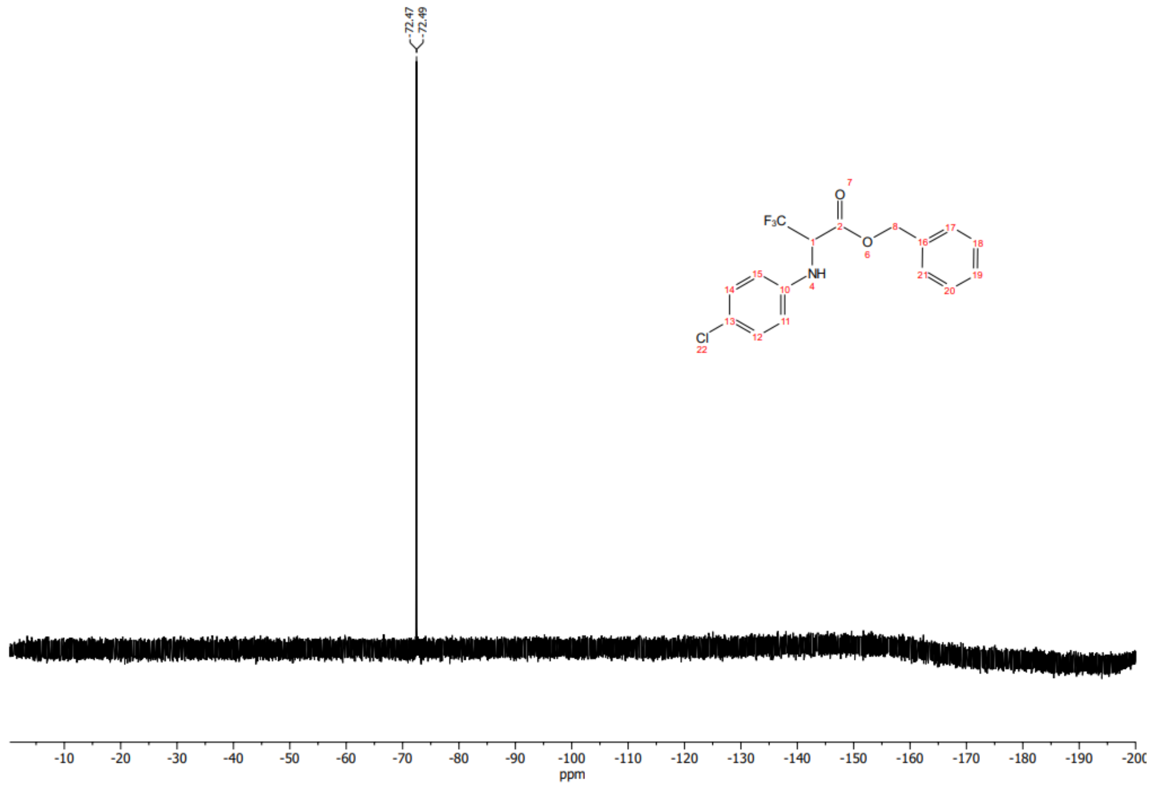

Benzyl 2-((4-bromophenyl)amino)-3,3,3-trifluoropropanoate (**4f**), ^{1}H NMR (400 MHz, $CDCl_3$):

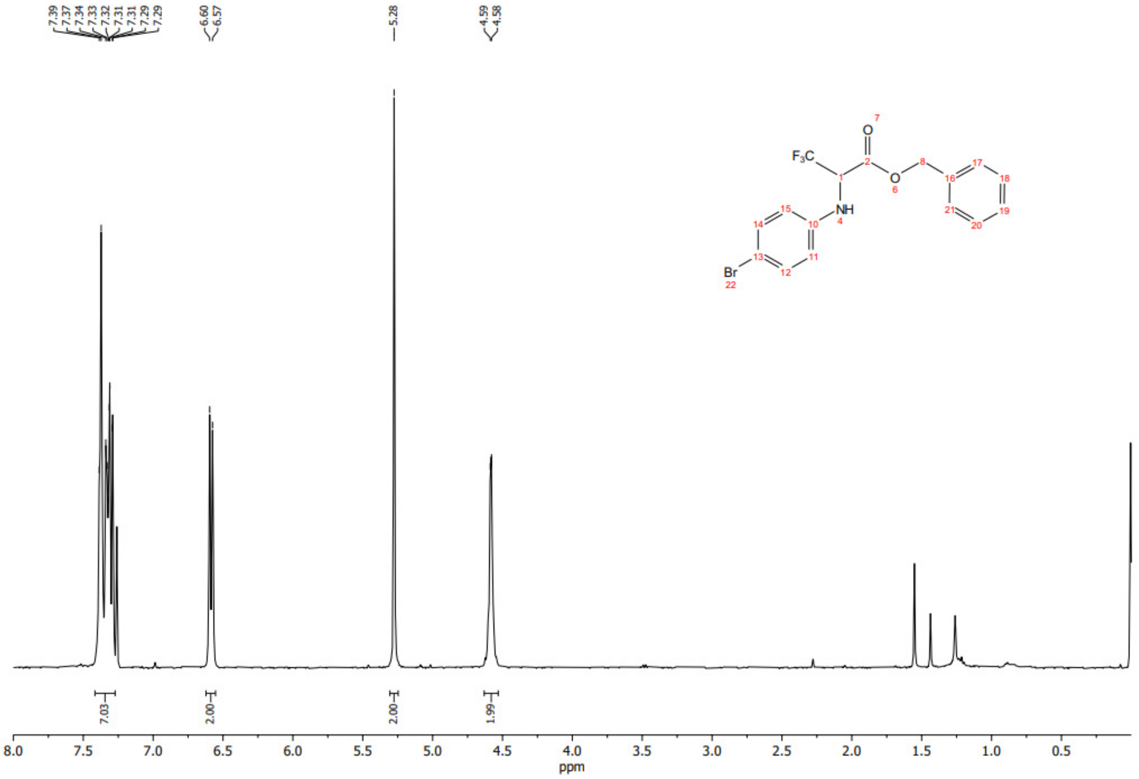

Benzyl 2-((4-bromophenyl)amino)-3,3,3-trifluoropropanoate (**4f**), ^{13}C NMR (126 MHz, $CDCl_3$):

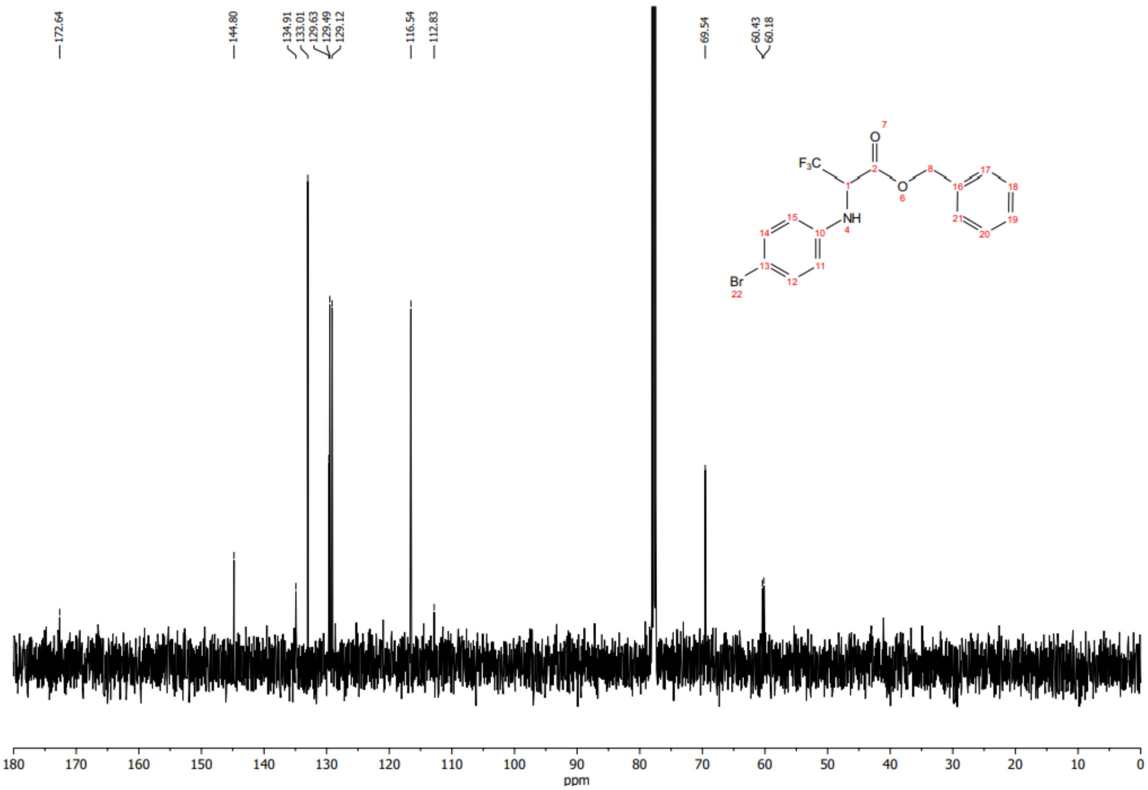

Benzyl 2-((4-bromophenyl)amino)-3,3,3-trifluoropropanoate (**4f**), ^{19}F NMR (376 MHz, $CDCl_3$):

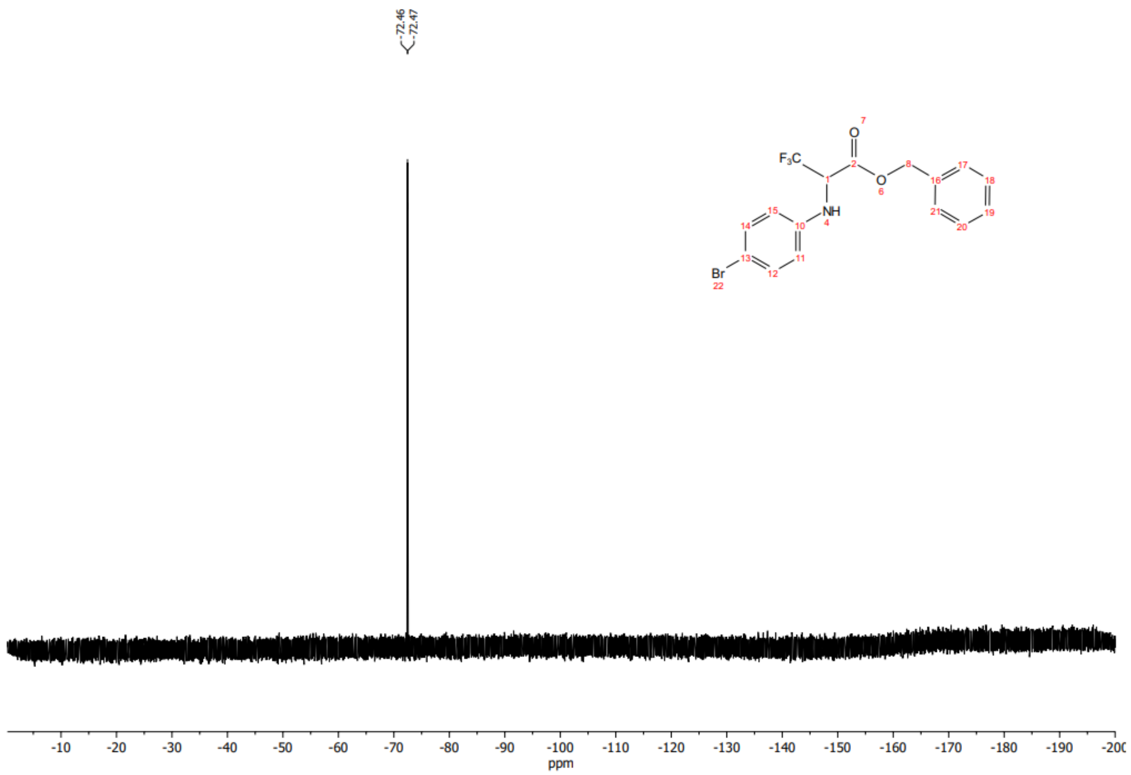

Benzyl 2-((3-chloro-4-fluorophenyl)amino)-3,3,3-trifluoropropanoate (**4g**), ^{1}H NMR (400 MHz, $CDCl_3$):

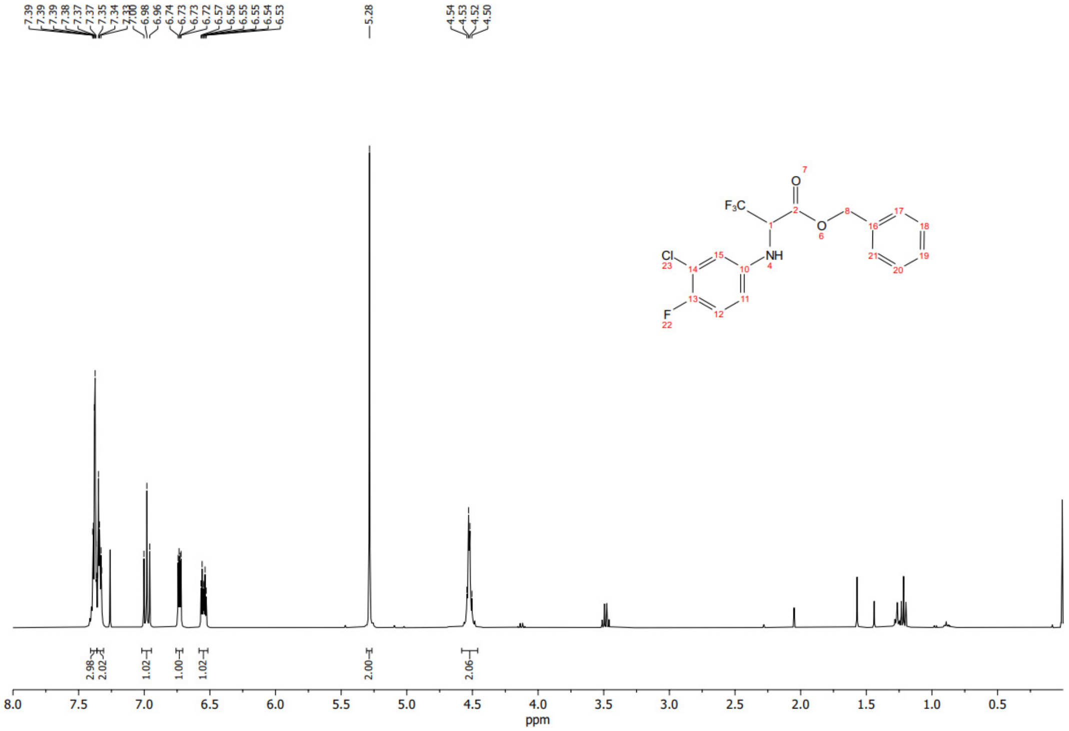

Benzyl 2-((3-chloro-4-fluorophenyl)amino)-3,3,3-trifluoropropanoate (**4g**), ^{13}C NMR (126 MHz, $CDCl_3$):

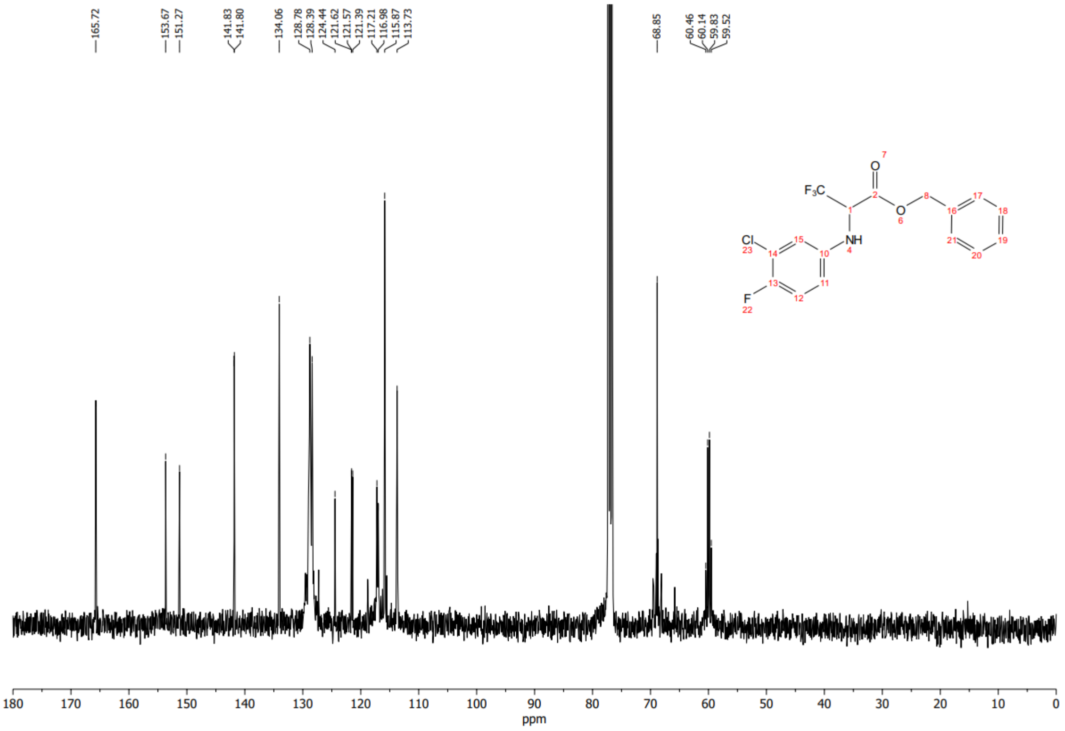

Benzyl 2-((3-chloro-4-fluorophenyl)amino)-3,3,3-trifluoropropanoate (**4g**), ^{19}F NMR (376 MHz, $CDCl_3$):

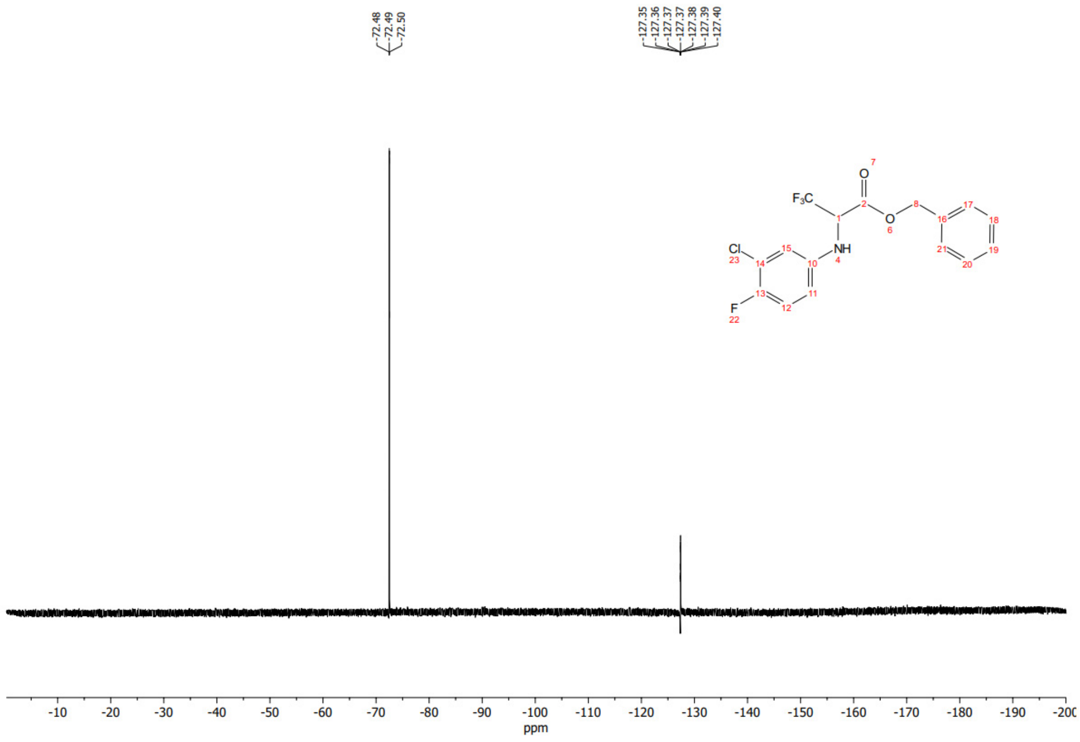

Benzyl 3,3,3-trifluoro-2-((4-isopropylphenyl)amino)propanoate (**4h**), ^{1}H NMR (400 MHz, $CDCl_3$):

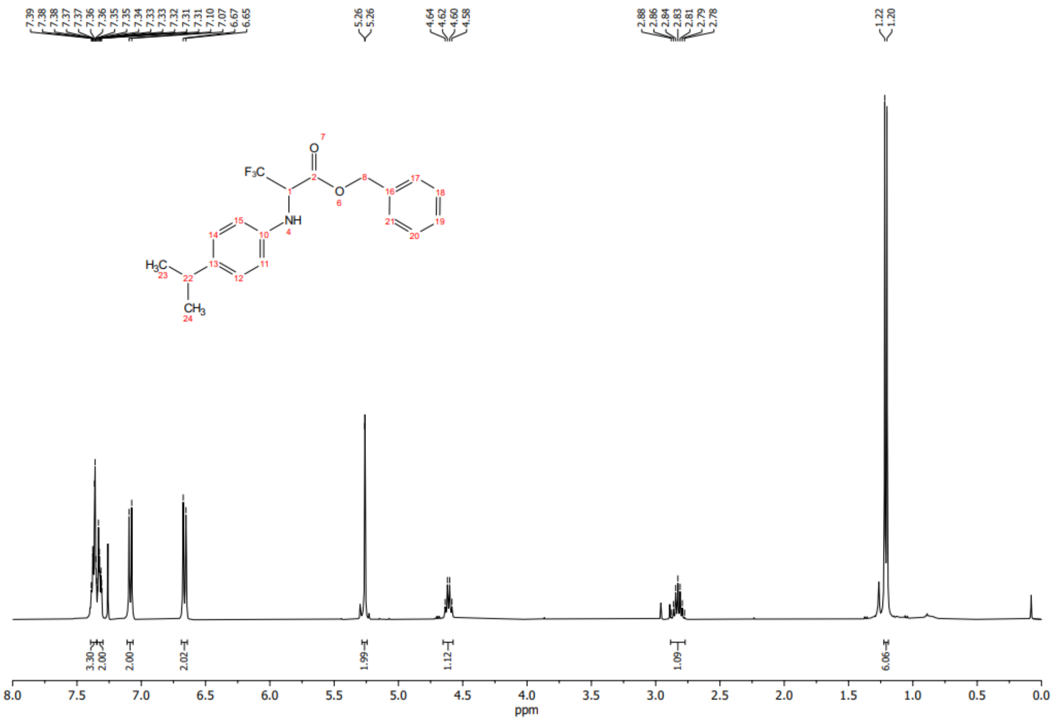

Benzyl 3,3,3-trifluoro-2-((4-isopropylphenyl)amino)propanoate (**4h**), ^{13}C NMR (126 MHz, $CDCl_3$):

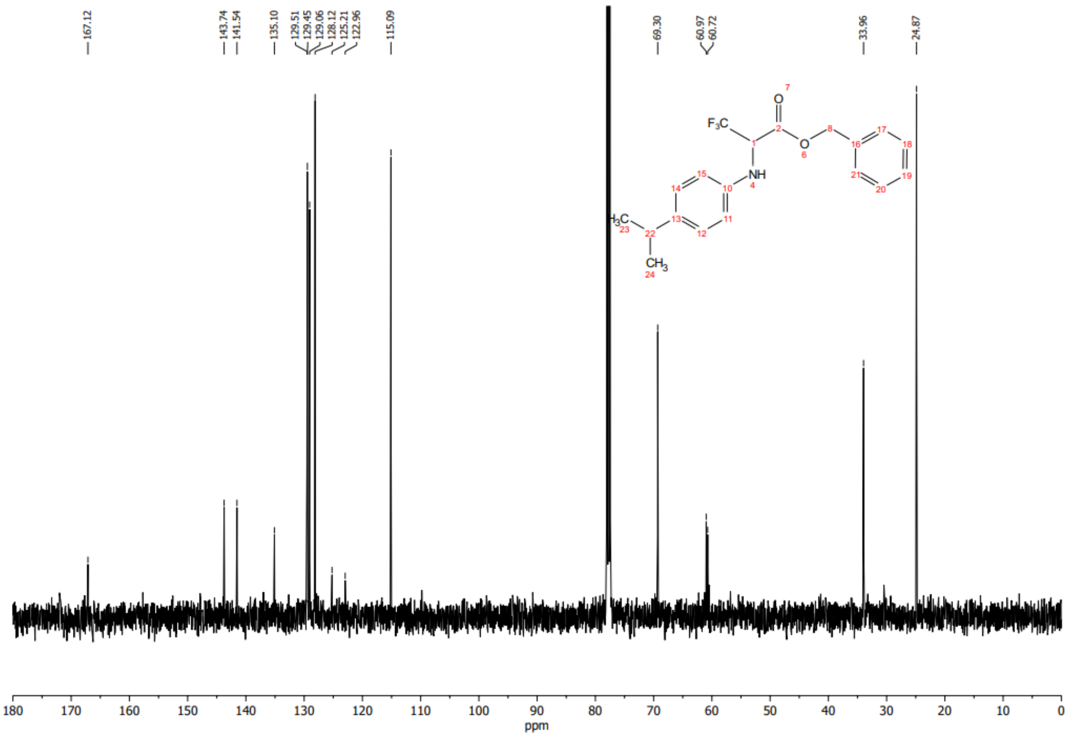

Benzyl 3,3,3-trifluoro-2-((4-isopropylphenyl)amino)propanoate (**4h**), ^{19}F NMR (376 MHz, $CDCl_3$):

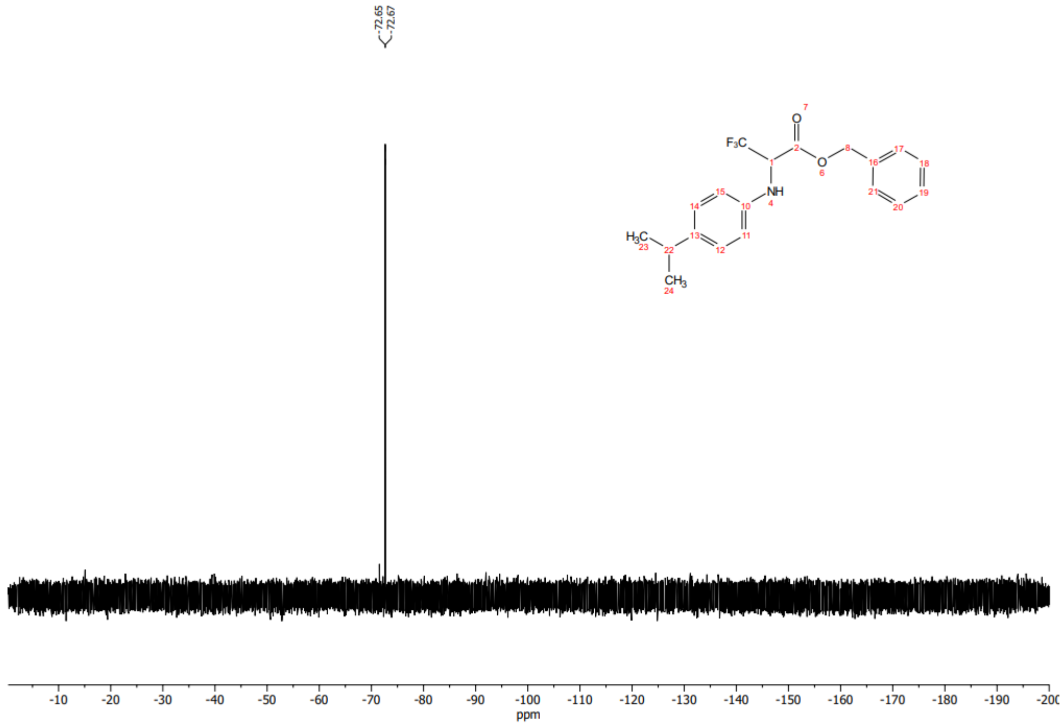

Benzyl 2-((4-(*tert*-butyl)phenyl)amino)-3,3,3-trifluoropropanoate (**4i**), ^{1}H NMR (400 MHz, $CDCl_3$):

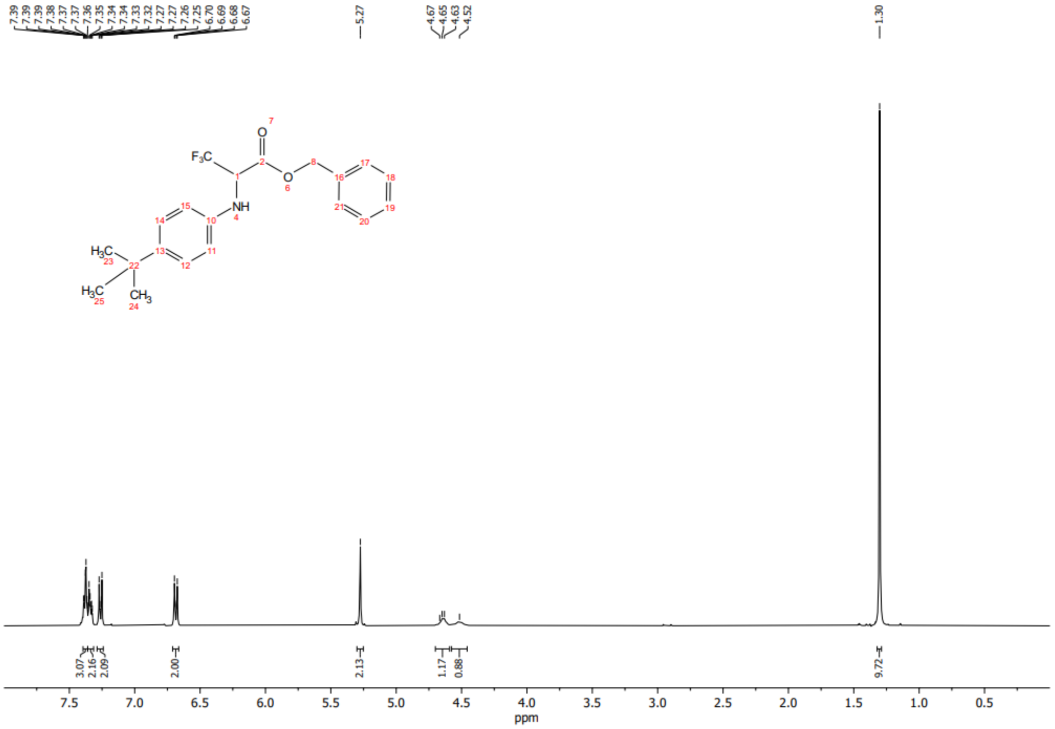

Benzyl 2-((4-(*tert*-butyl)phenyl)amino)-3,3,3-trifluoropropanoate (**4i**), ^{13}C NMR (126 MHz, $CDCl_3$):

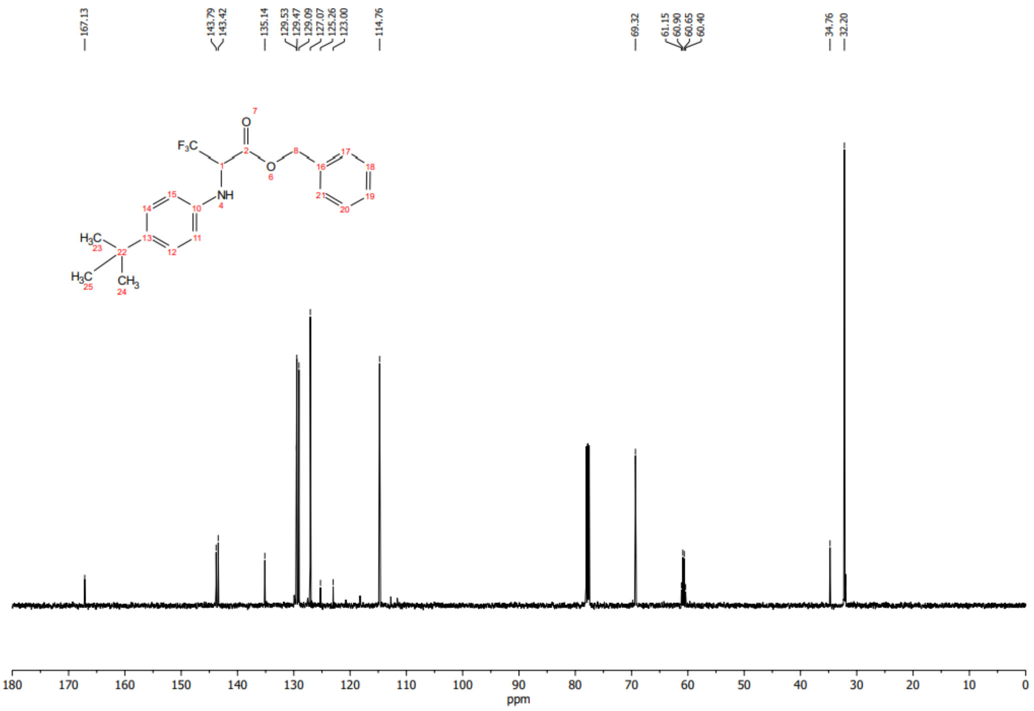

Benzyl 2-((4-(*tert*-butyl)phenyl)amino)-3,3,3-trifluoropropanoate (**4i**), ^{19}F NMR (376 MHz, $CDCl_3$):

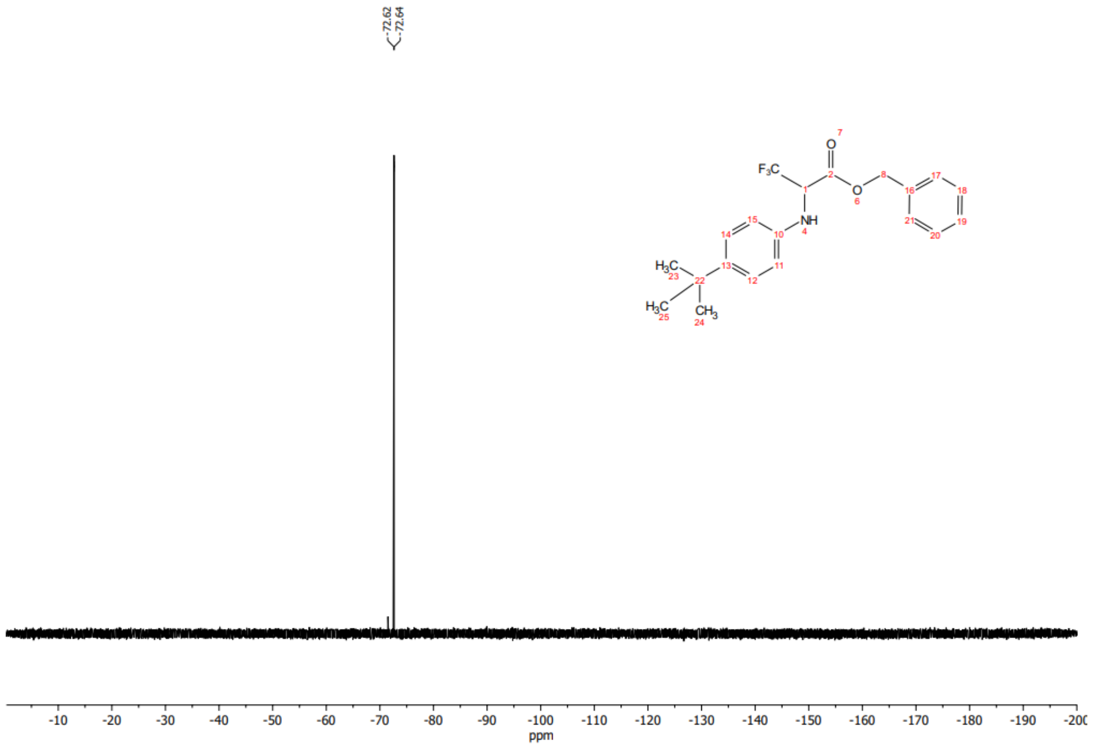

Benzyl 3,3,3-trifluoro-2-(naphthalen-2-ylamino)propanoate (**4j**), ^{1}H NMR (400 MHz, $CDCl_3$):

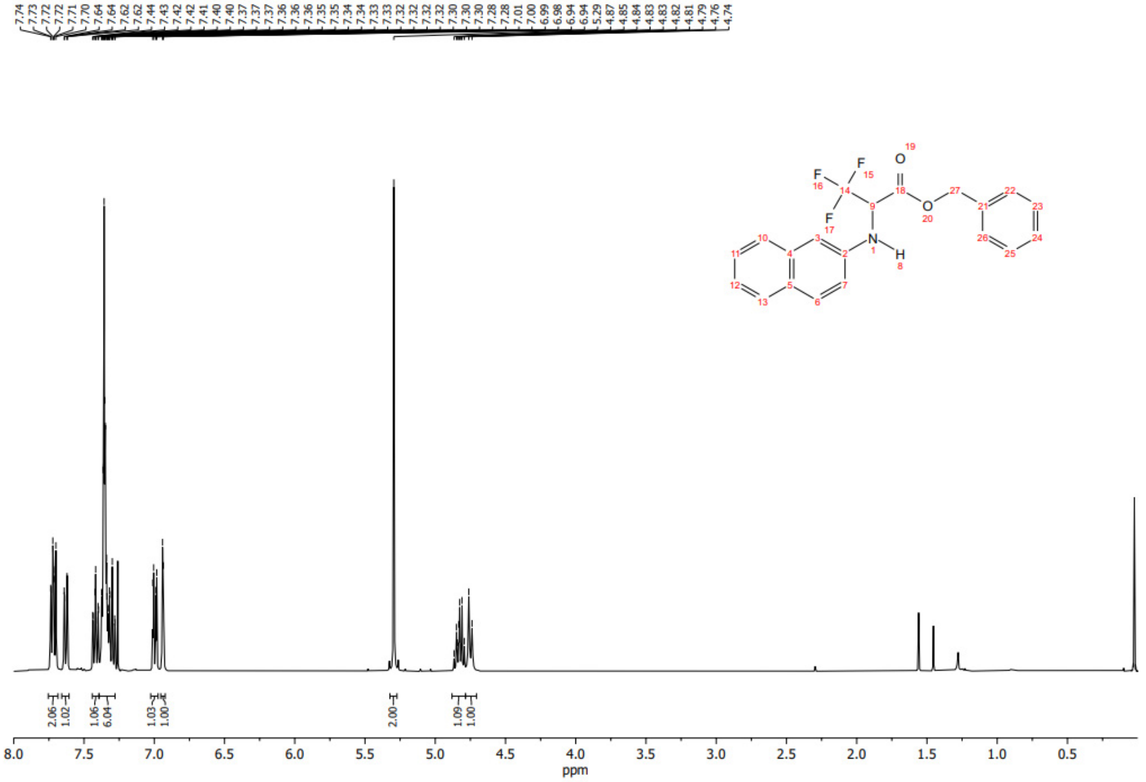

Benzyl 3,3,3-trifluoro-2-(naphthalen-2-ylamino)propanoate (**4j**), ^{13}C NMR (126 MHz, $CDCl_3$):

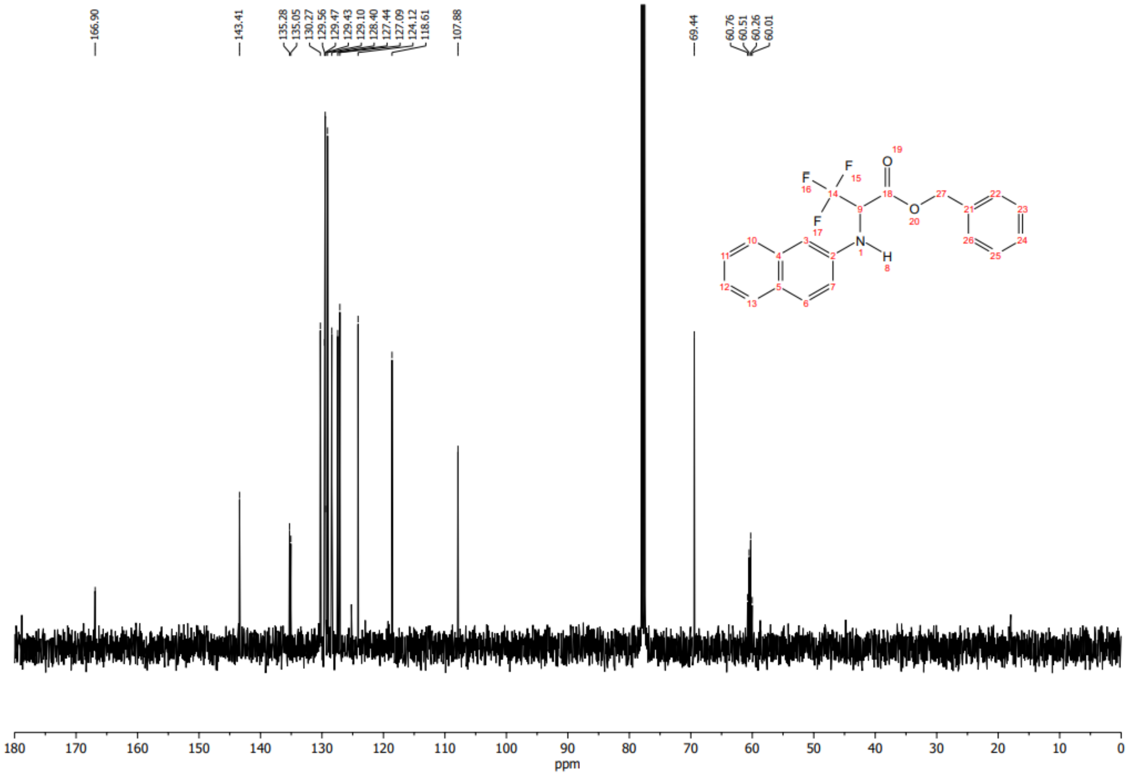

Benzyl 3,3,3-trifluoro-2-(naphthalen-2-ylamino)propanoate (**4j**), ^{19}F NMR (376 MHz, $CDCl_3$):

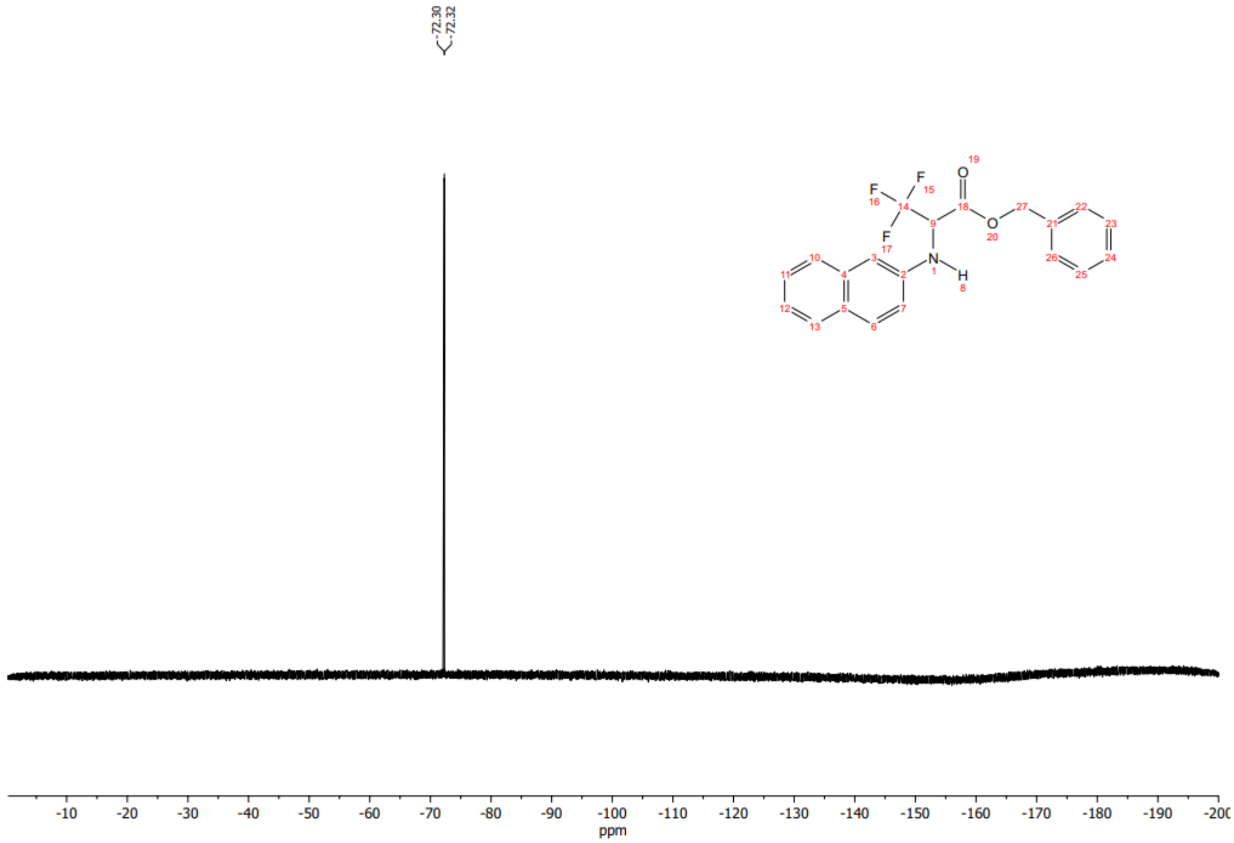

Benzyl 2-(benzo[*d*][1,3]dioxol-5-ylamino)-3,3,3-trifluoropropanoate (**4k**), ^{1}H NMR (400 MHz, $CDCl_3$):

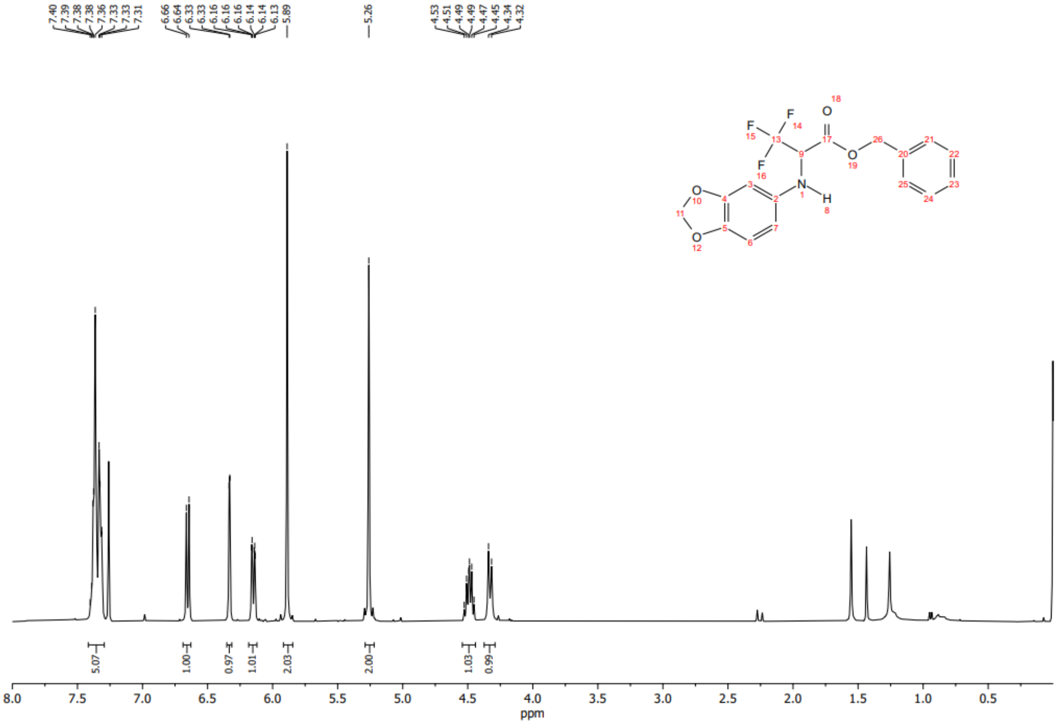

Benzyl 2-(benzo[*d*][1,3]dioxol-5-ylamino)-3,3,3-trifluoropropanoate (**4k**), ^{13}C NMR (126 MHz, $CDCl_3$):

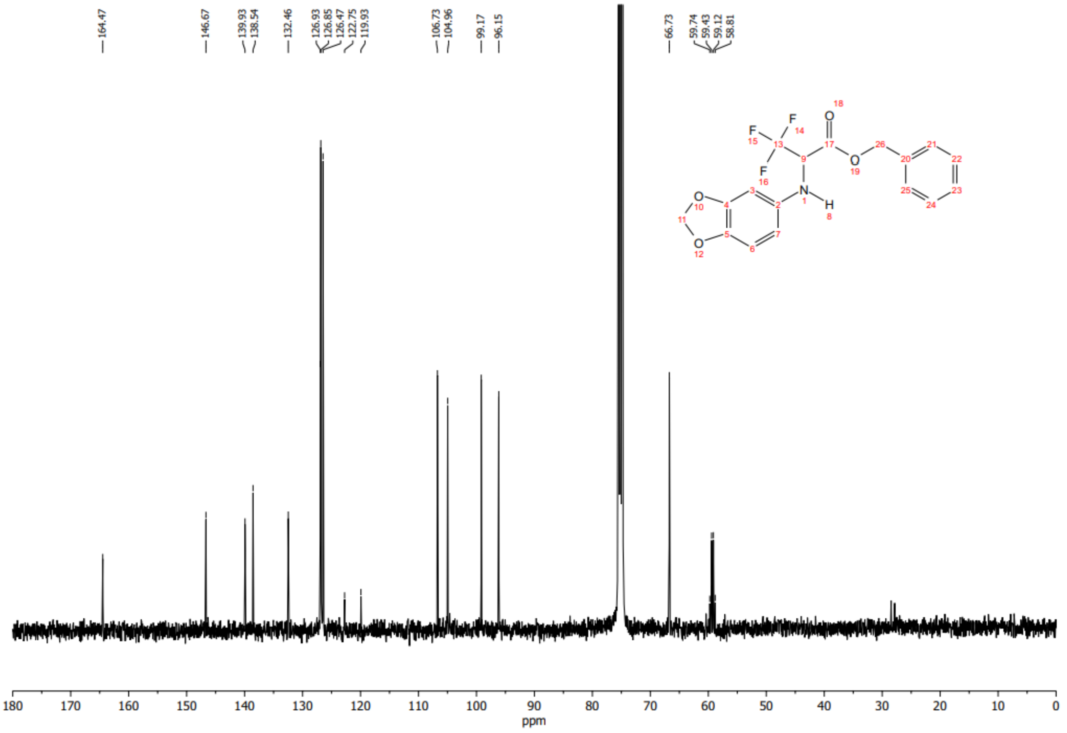

Benzyl 2-(benzo[*d*][1,3]dioxol-5-ylamino)-3,3,3-trifluoropropanoate (**4k**), ^{19}F NMR (376 MHz, $CDCl_3$):

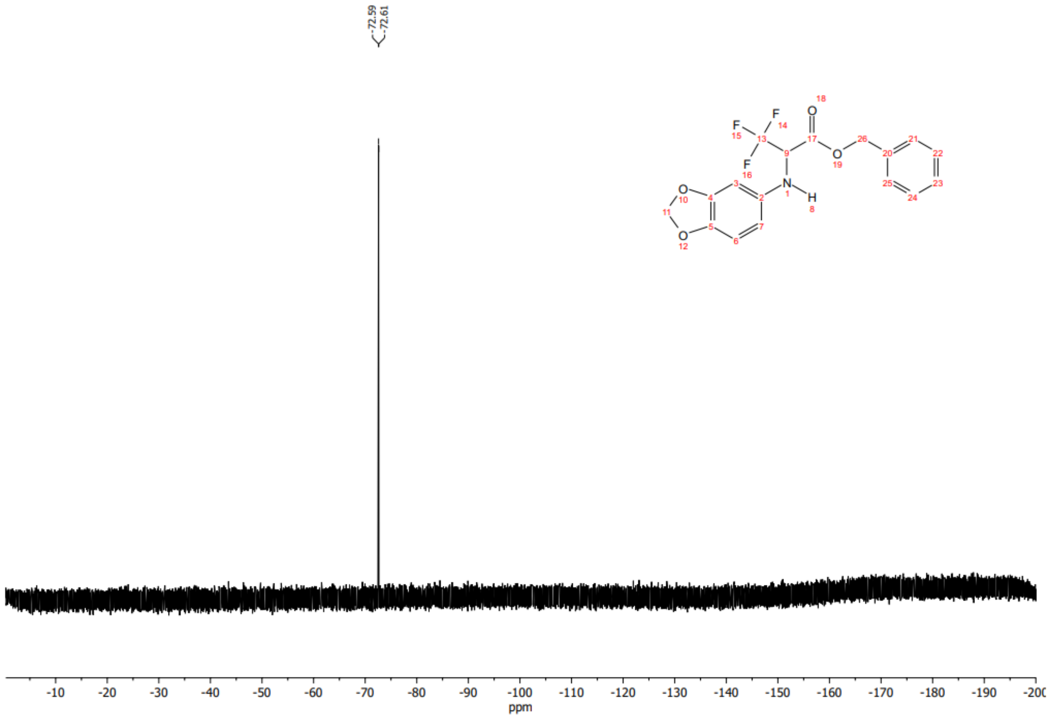

Benzyl 2-((4-acetylphenyl)amino)-3,3,3-trifluoropropanoate **(4l)**, ^{1}H NMR (400 MHz, $CDCl_3$):

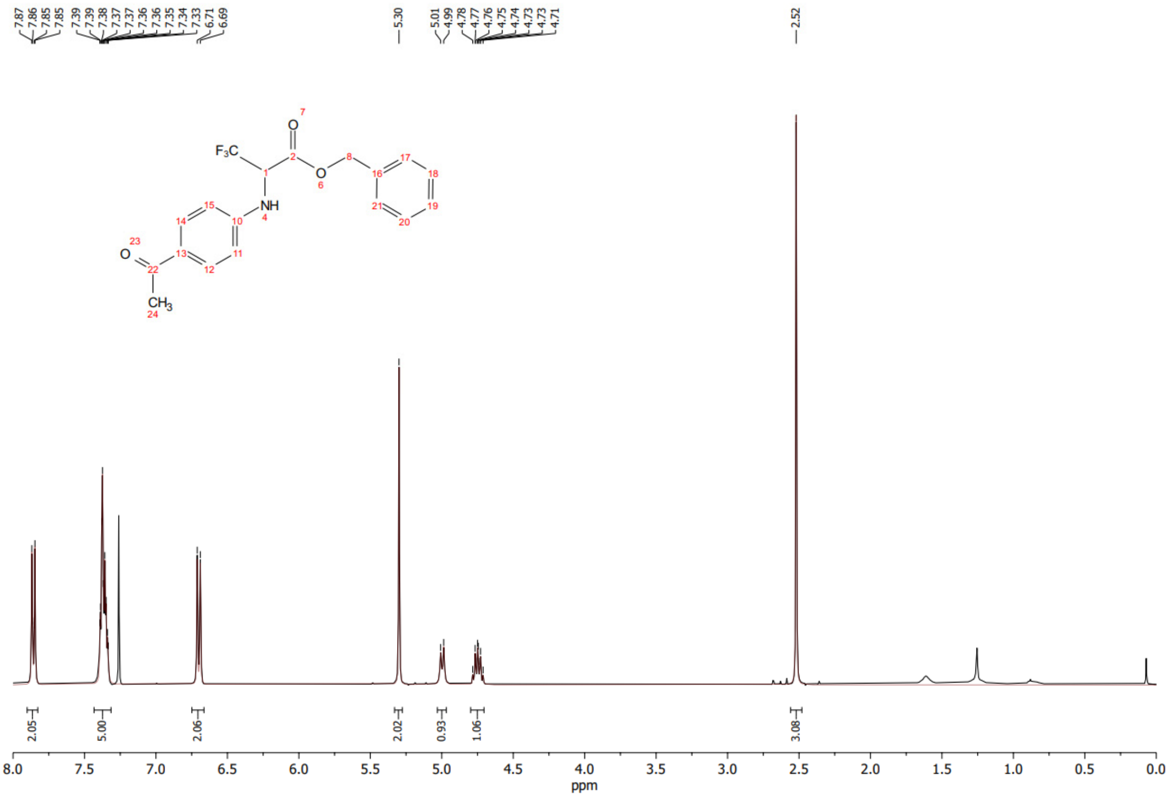

Benzyl 2-((4-acetylphenyl)amino)-3,3,3-trifluoropropanoate (**4l**), ^{13}C NMR (126 MHz, $CDCl_3$):

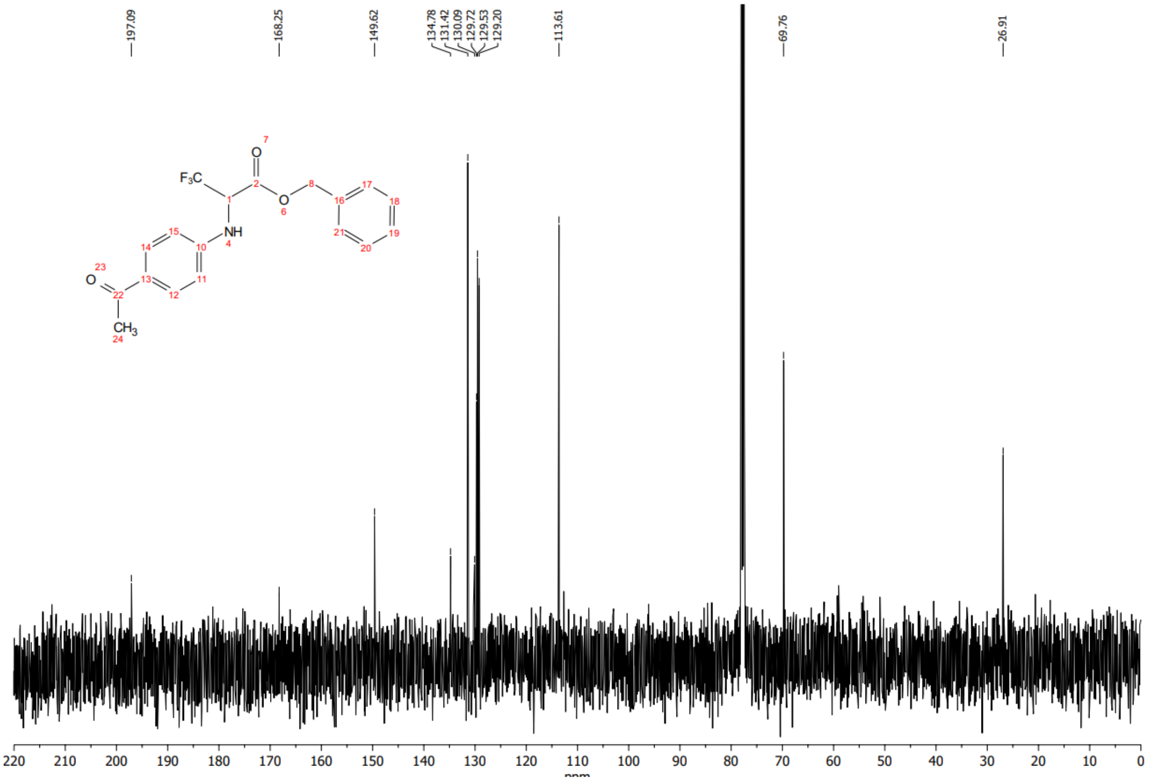

Benzyl 2-((4-acetylphenyl)amino)-3,3,3-trifluoropropanoate (**4l**), ^{19}F NMR (376 MHz, $CDCl_3$):

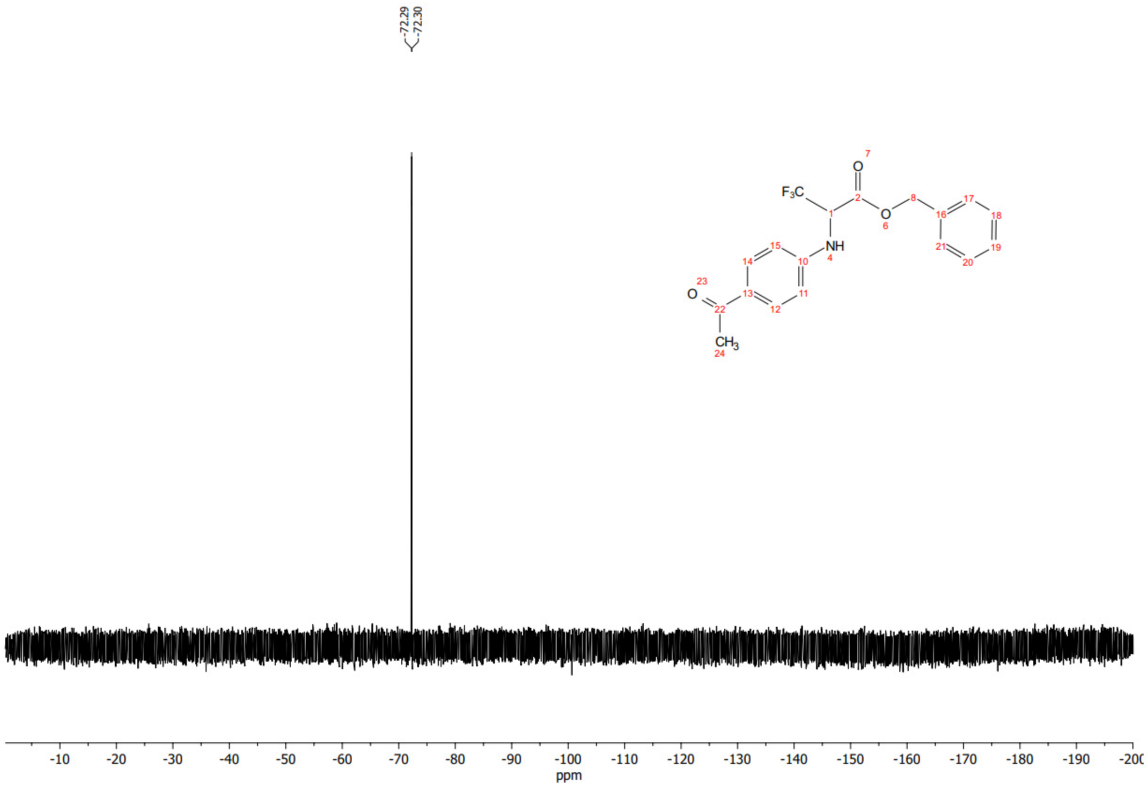

Benzyl 2-((4-cyanophenyl)amino)-3,3,3-trifluoropropanoate **(4m)**, ^{1}H NMR (400 MHz, $CDCl_3$):

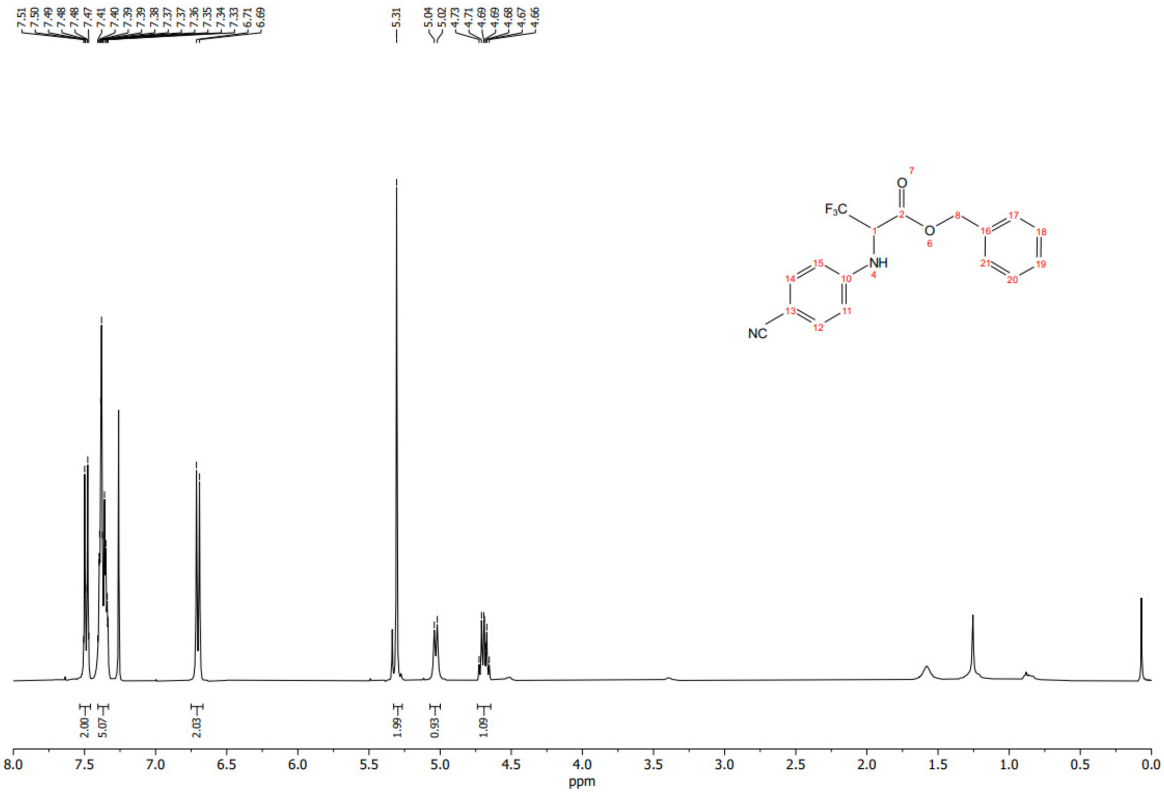

Benzyl 2-((4-cyanophenyl)amino)-3,3,3-trifluoropropanoate (**4m**), ^{13}C NMR (126 MHz, $CDCl_3$):

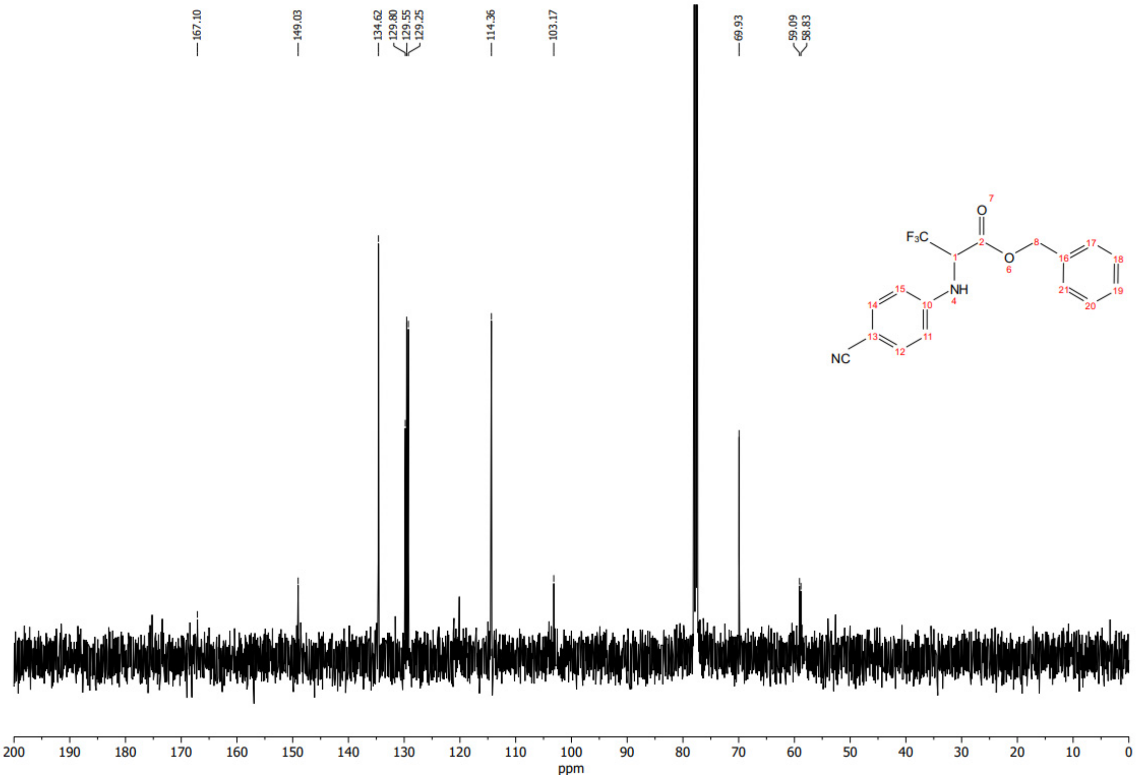

Benzyl 2-((4-cyanophenyl)amino)-3,3,3-trifluoropropanoate (**4m**), ^{19}F NMR (376 MHz, $CDCl_3$):

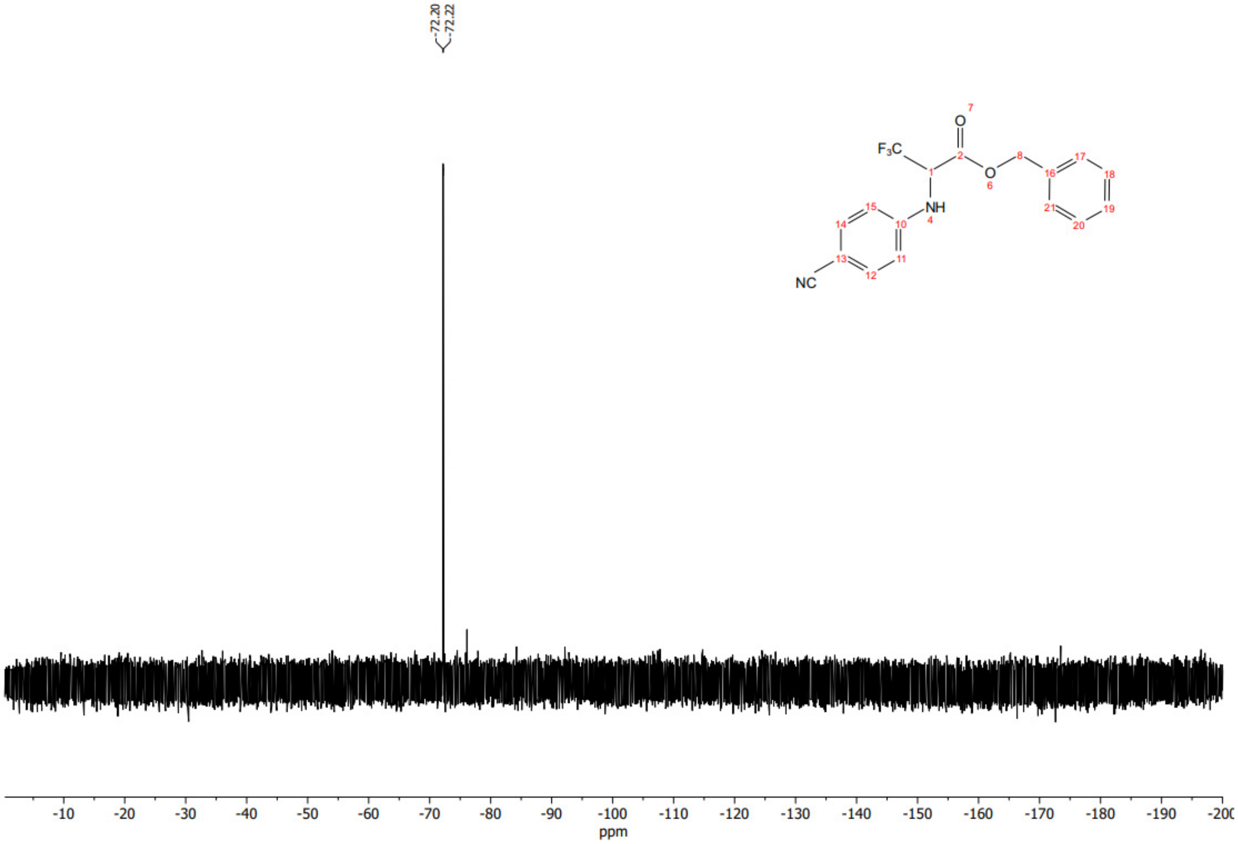

Benzyl 3,3,3-trifluoro-2-((3-(trifluoromethyl)phenyl)amino)propanoate (**4n**), ^{1}H NMR (400 MHz, $CDCl_3$):

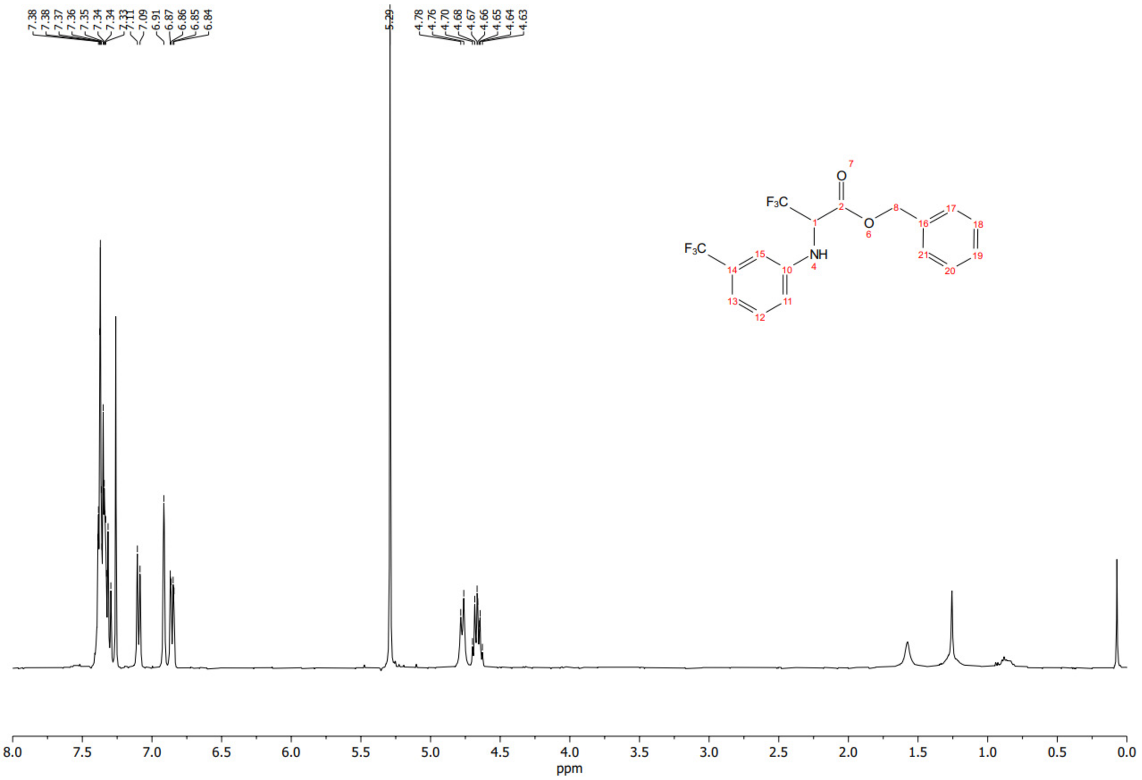

Benzyl 3,3,3-trifluoro-2-((3-(trifluoromethyl)phenyl)amino)propanoate (**4n**), ^{13}C NMR (126 MHz, $CDCl_3$):

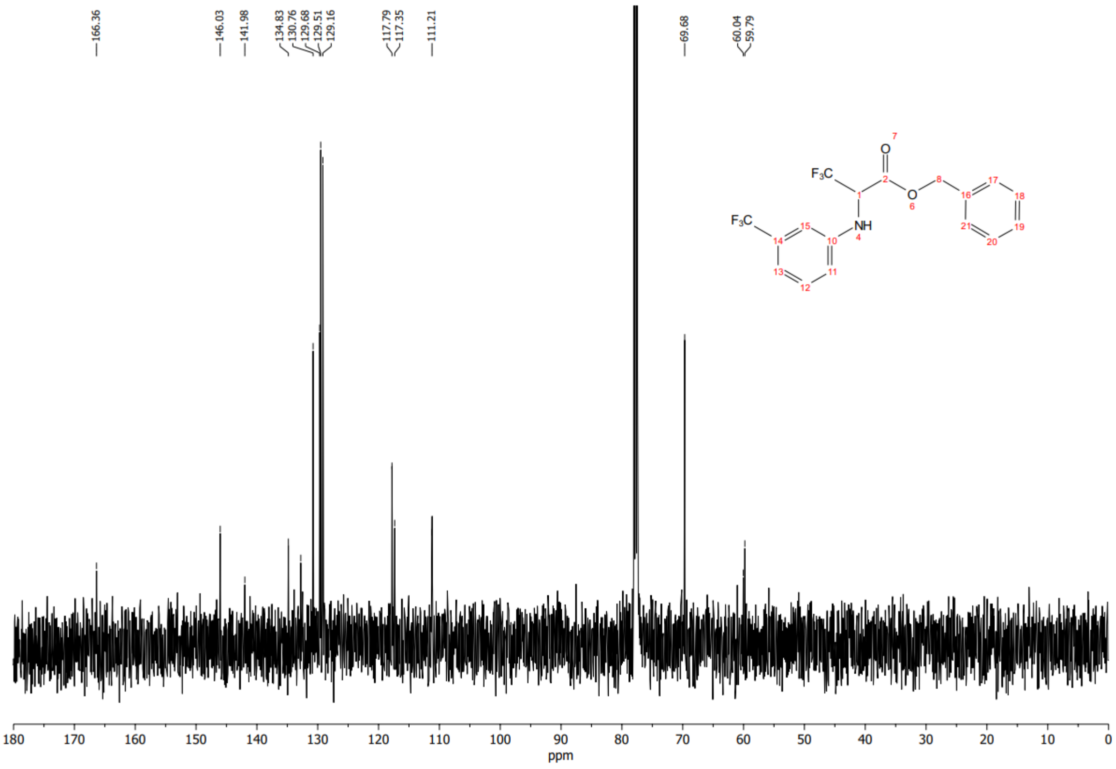

Benzyl 3,3,3-trifluoro-2-((3-(trifluoromethyl)phenyl)amino)propanoate (**4n**), ^{19}F NMR (376 MHz, $CDCl_3$):

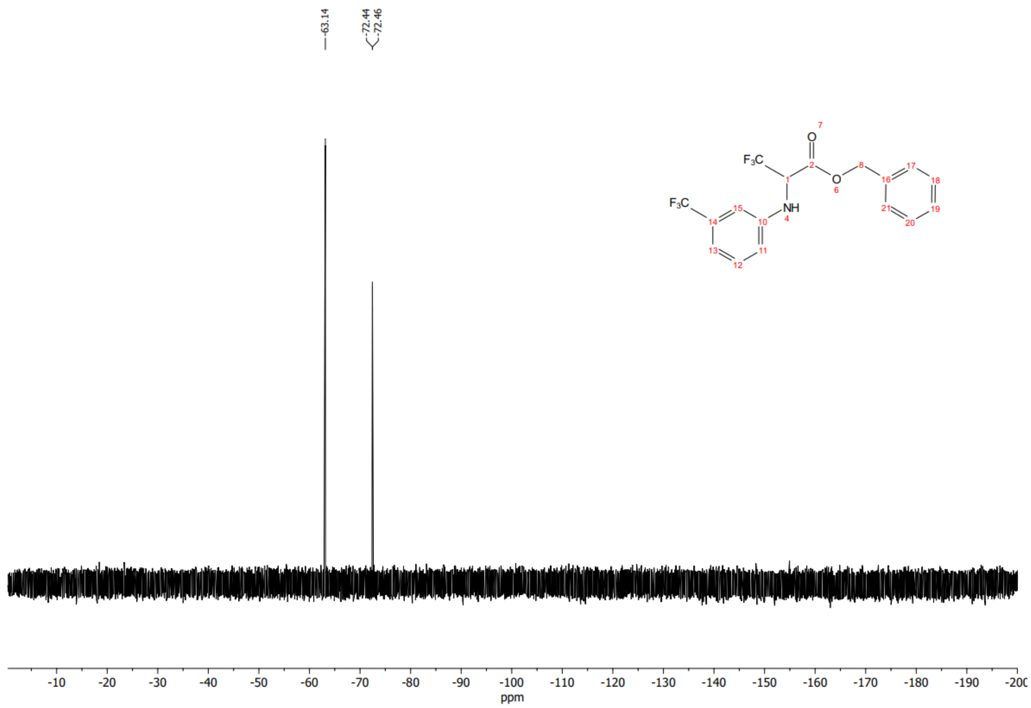

2,5-Dimethylbenzyl 3,3,3-trifluoro-2-(phenylamino)propanoate (**5a**), ^{1}H NMR (400 MHz, $CDCl_3$):

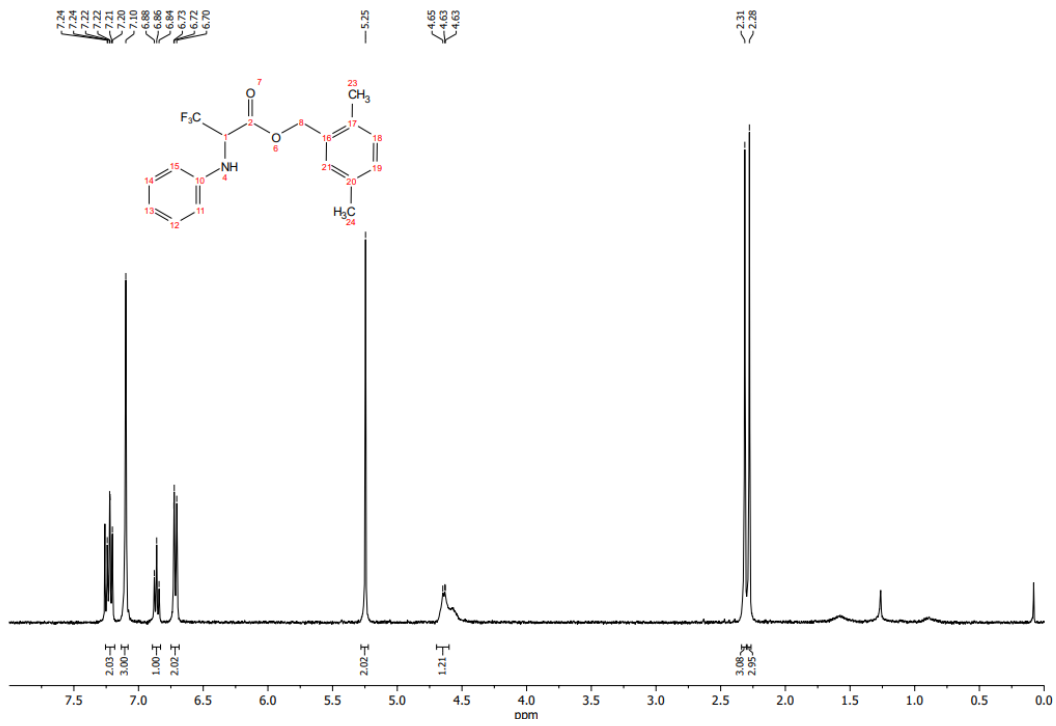

2,5-Dimethylbenzyl 3,3,3-trifluoro-2-(phenylamino)propanoate (**5a**), ^{13}C NMR (126 MHz, $CDCl_3$):

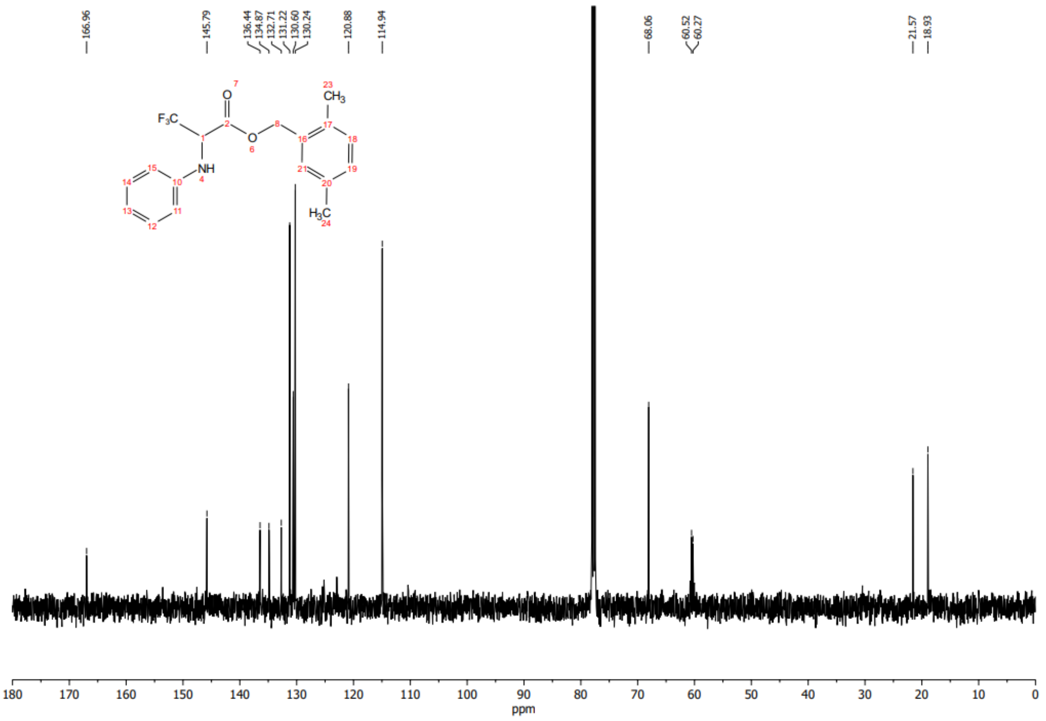

2,5-Dimethylbenzyl 3,3,3-trifluoro-2-(phenylamino)propanoate (**5a**), ^{19}F NMR (376 MHz, $CDCl_3$):

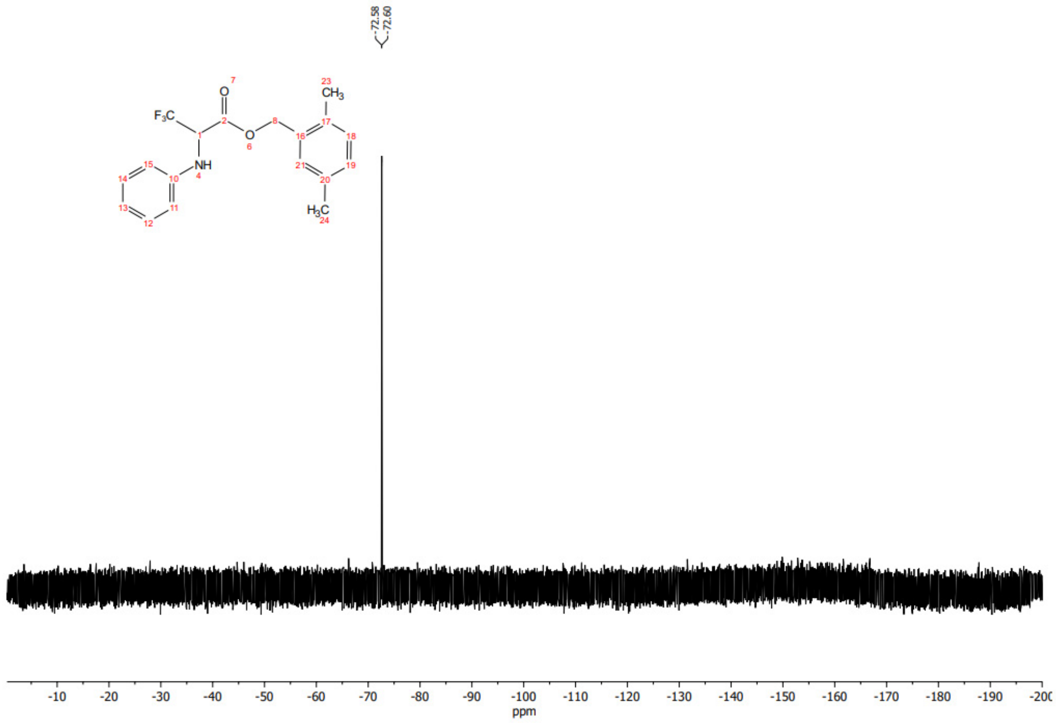

2,5-Dimethylbenzyl 3,3,3-trifluoro-2-(p-tolylamino)propanoate (**5c**), ^{1}H NMR (400 MHz, $CDCl_3$):

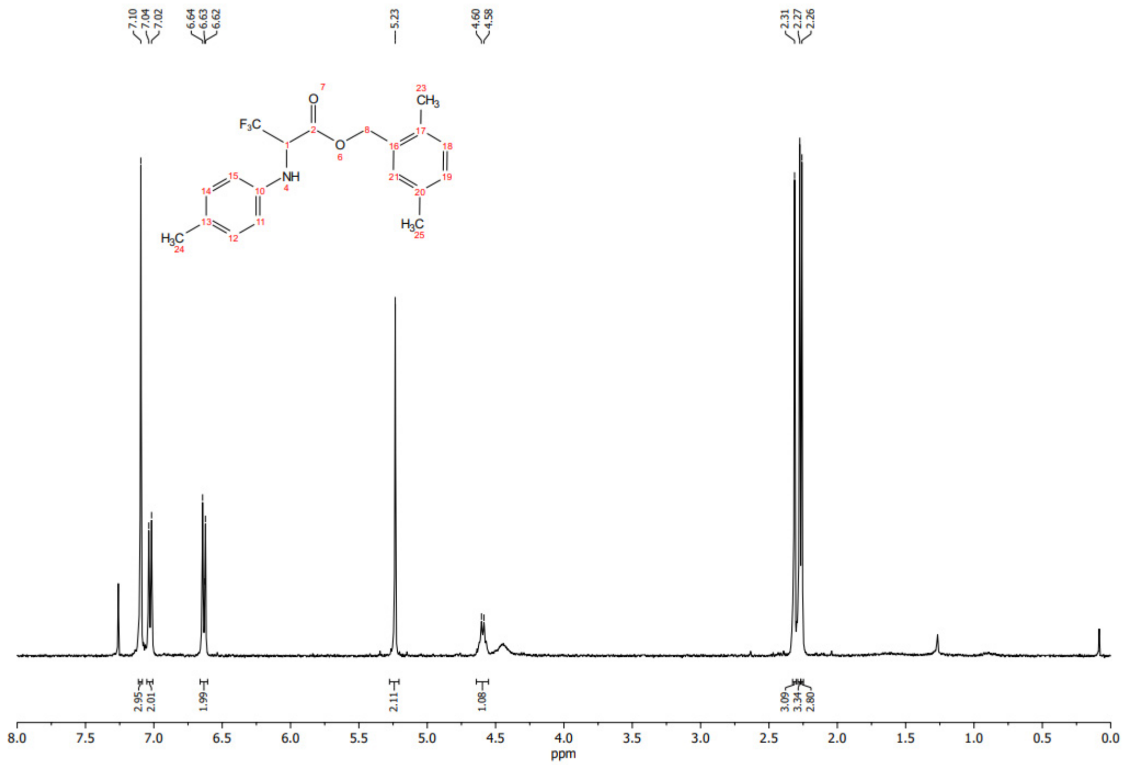

2,5-Dimethylbenzyl 3,3,3-trifluoro-2-(p-tolylamino)propanoate (**5c**), ^{13}C NMR (126 MHz, $CDCl_3$):

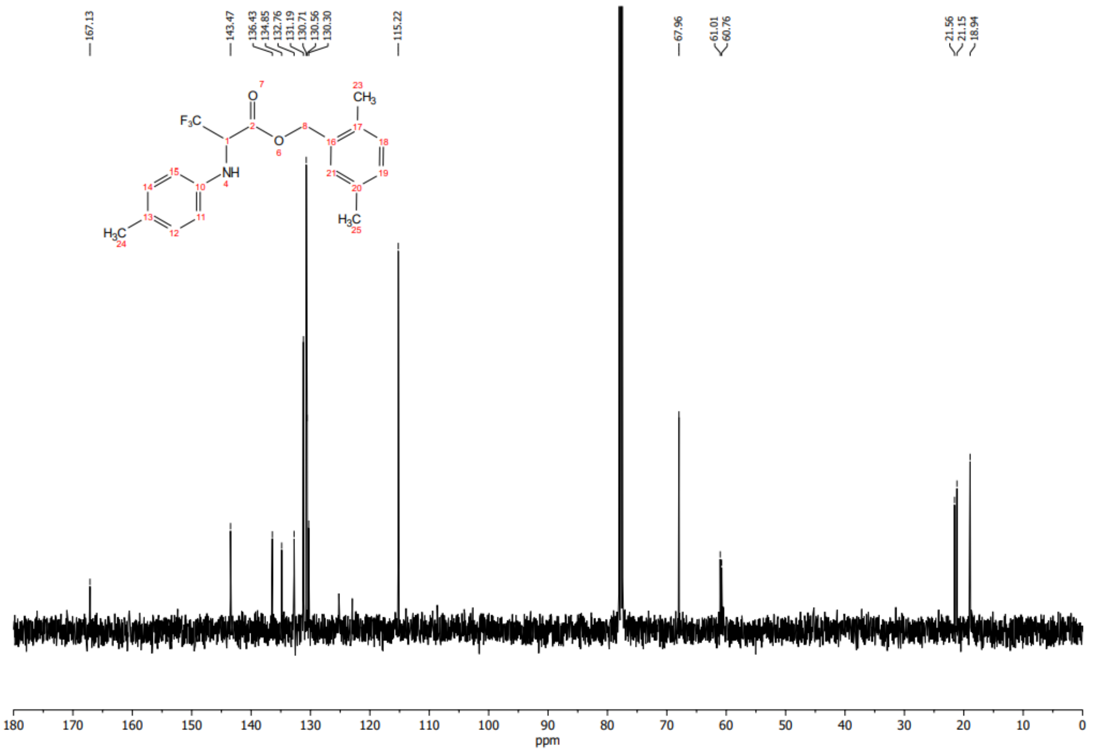

2,5-Dimethylbenzyl 3,3,3-trifluoro-2-(p-tolylamino)propanoate (**5c**), ^{19}F NMR (376 MHz, $CDCl_3$):

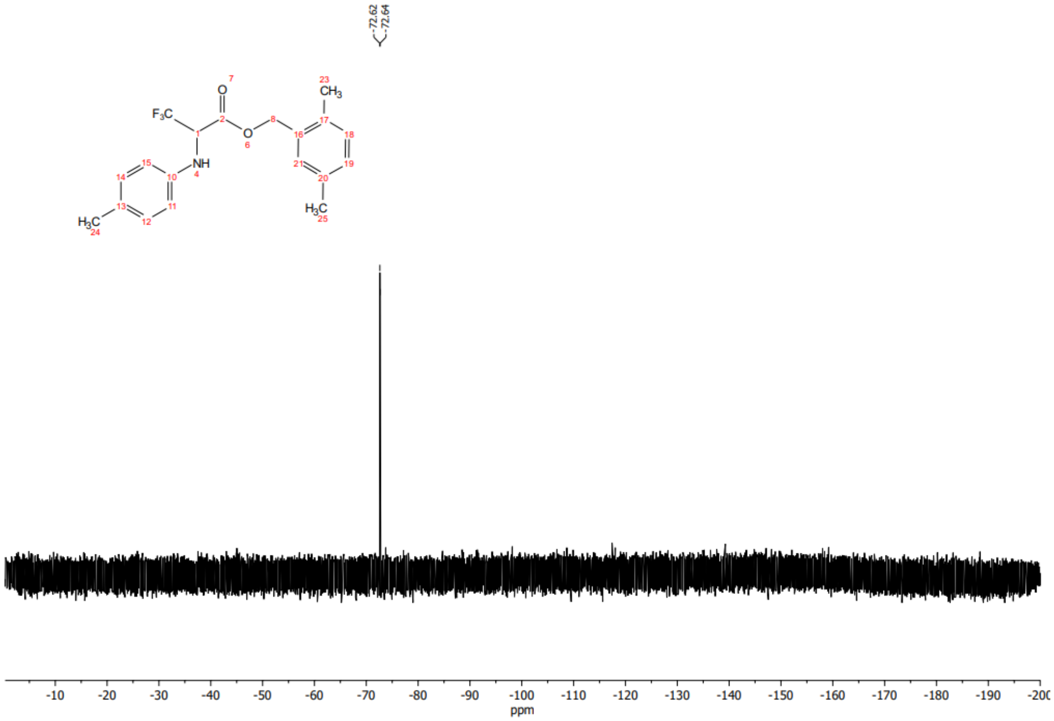

2,5-Dimethylbenzyl 3,3,3-trifluoro-2-(m-tolylamino)propanoate (**5d**), ^{1}H NMR (400 MHz, $CDCl_3$):

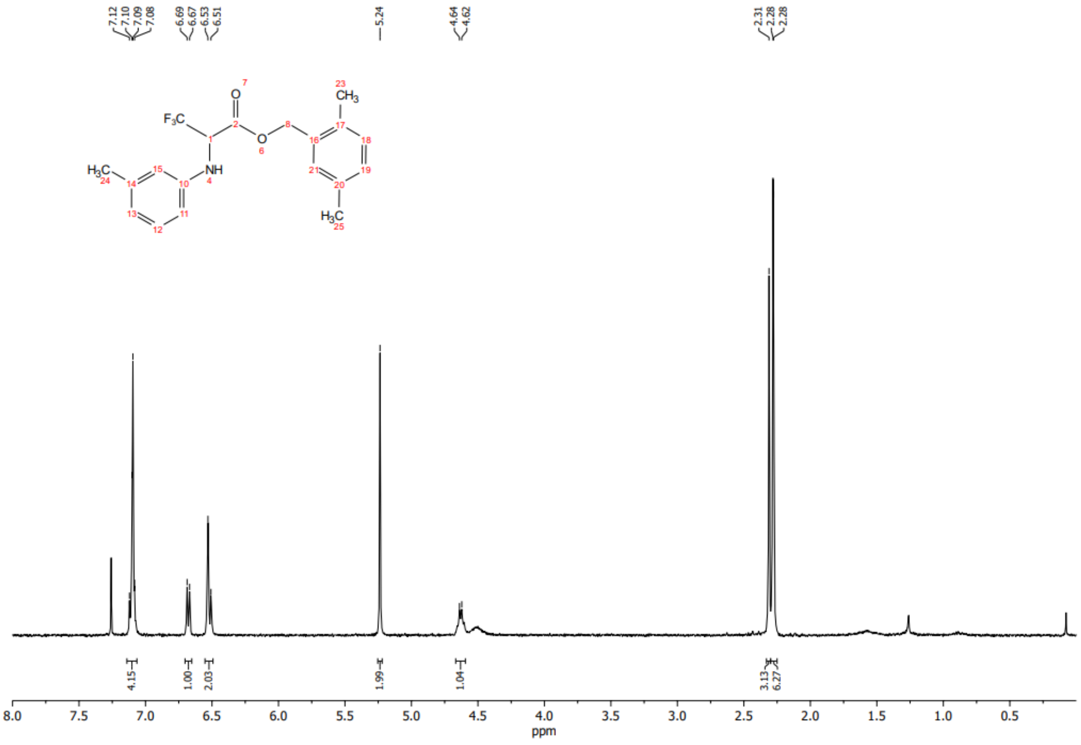

2,5-Dimethylbenzyl 3,3,3-trifluoro-2-(m-tolylamino)propanoate (**5d**), ^{13}C NMR (126 MHz, $CDCl_3$):

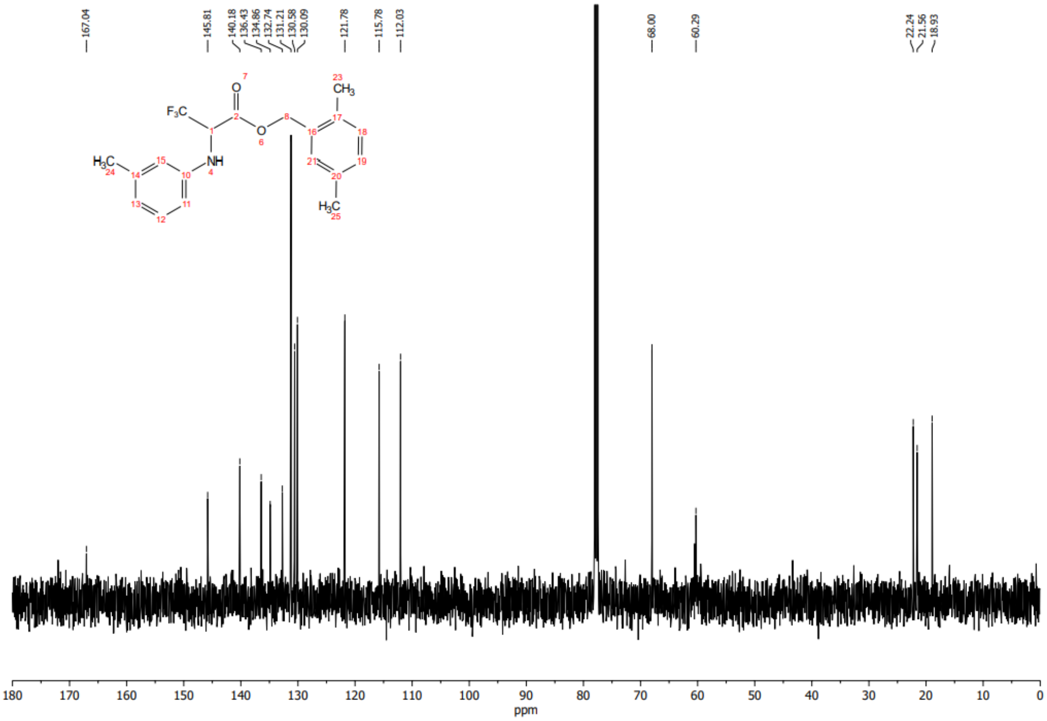

2,5-Dimethylbenzyl 3,3,3-trifluoro-2-(m-tolylamino)propanoate (**5d**), ^{19}F NMR (376 MHz, $CDCl_3$):

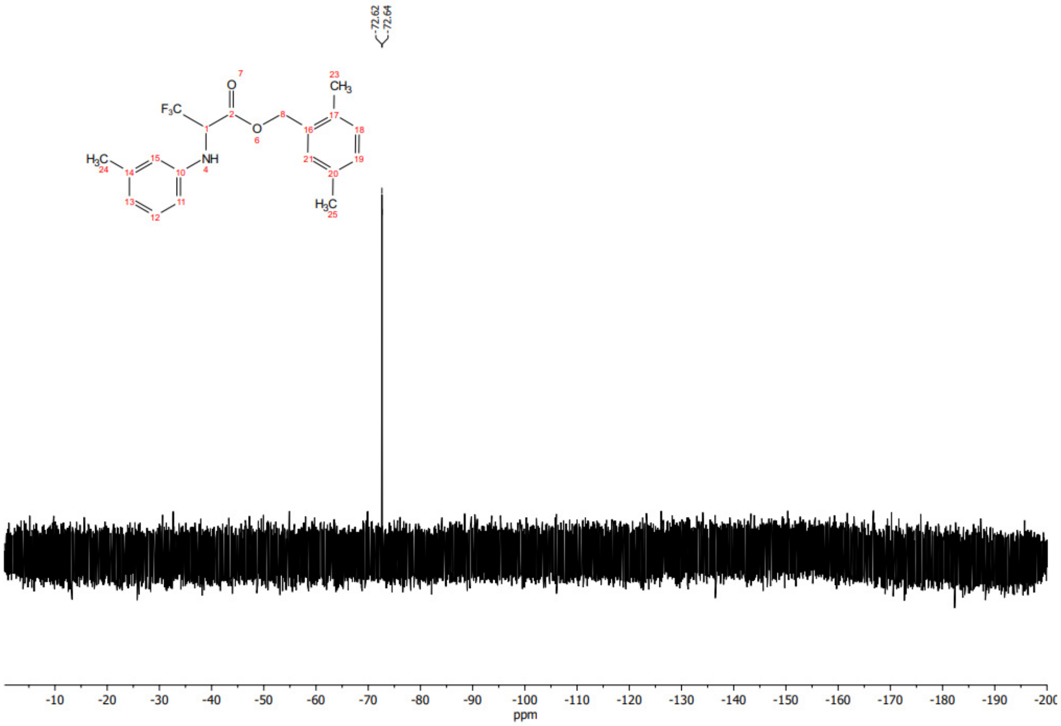

2,5-Dimethylbenzyl 3,3,3-trifluoro-2-(o-tolylamino)propanoate (**5e**), ^{1}H NMR (400 MHz, $CDCl_3$):

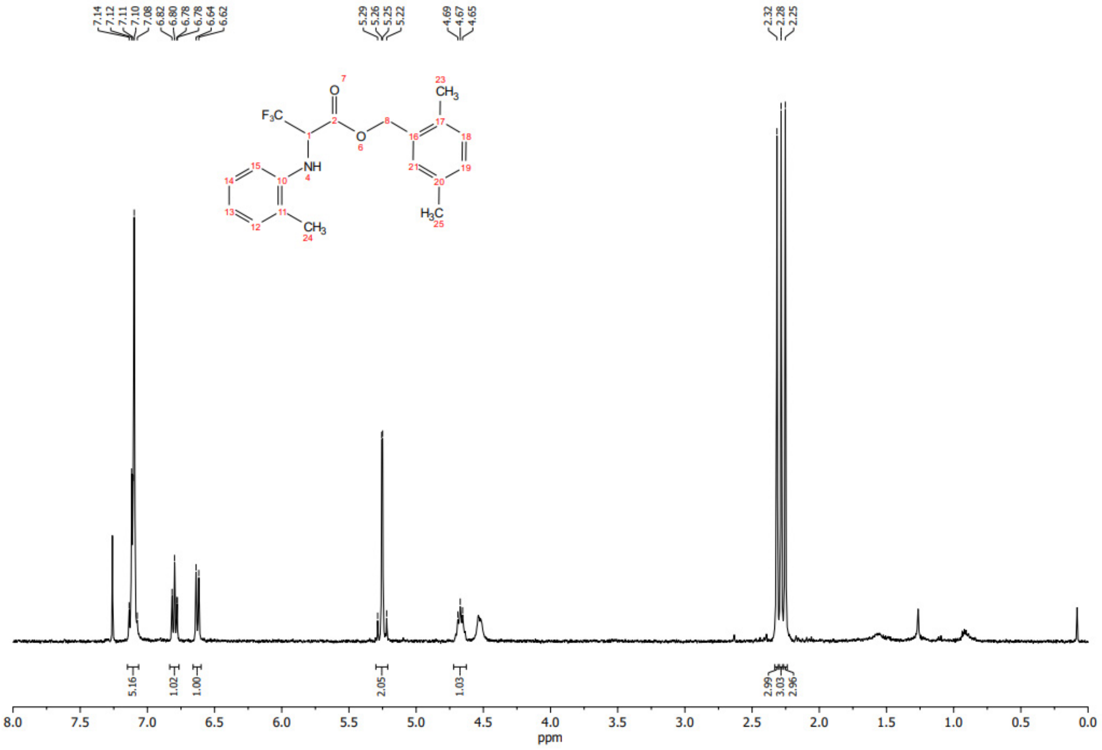

2,5-Dimethylbenzyl 3,3,3-trifluoro-2-(o-tolylamino)propanoate (**5e**), ^{13}C NMR (126 MHz, $CDCl_3$):

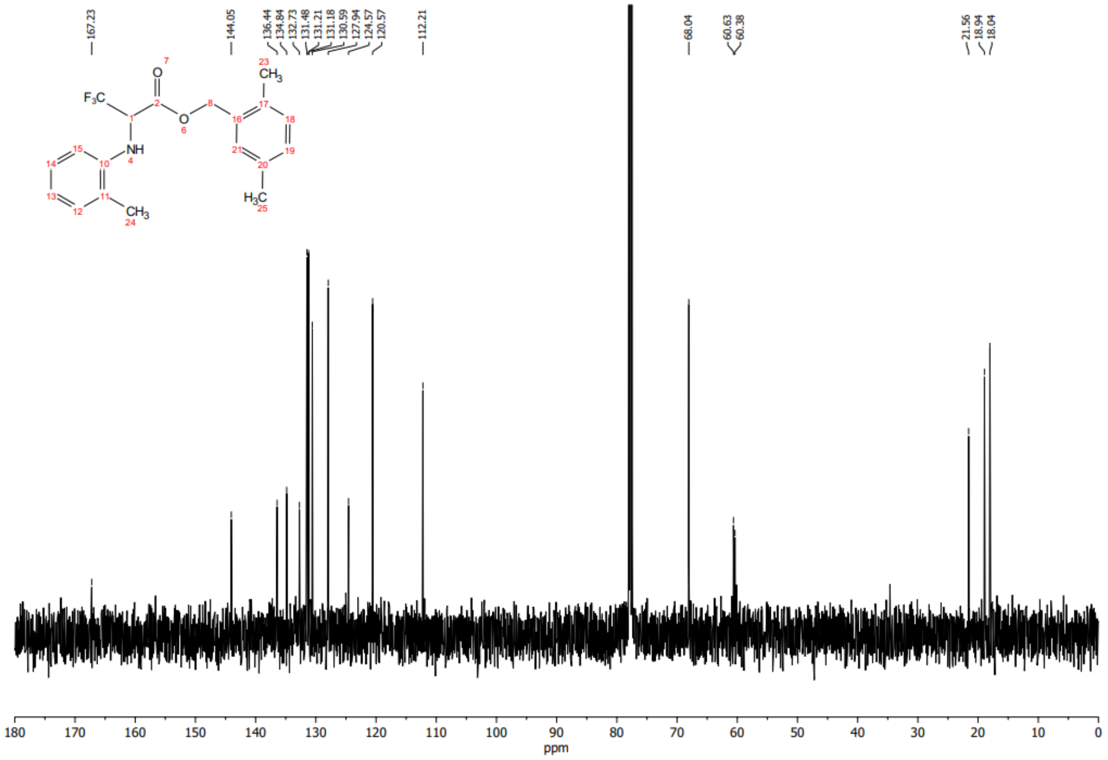

2,5-Dimethylbenzyl 3,3,3-trifluoro-2-(o-tolylamino)propanoate (**5e**), ^{19}F NMR (376 MHz, $CDCl_3$):

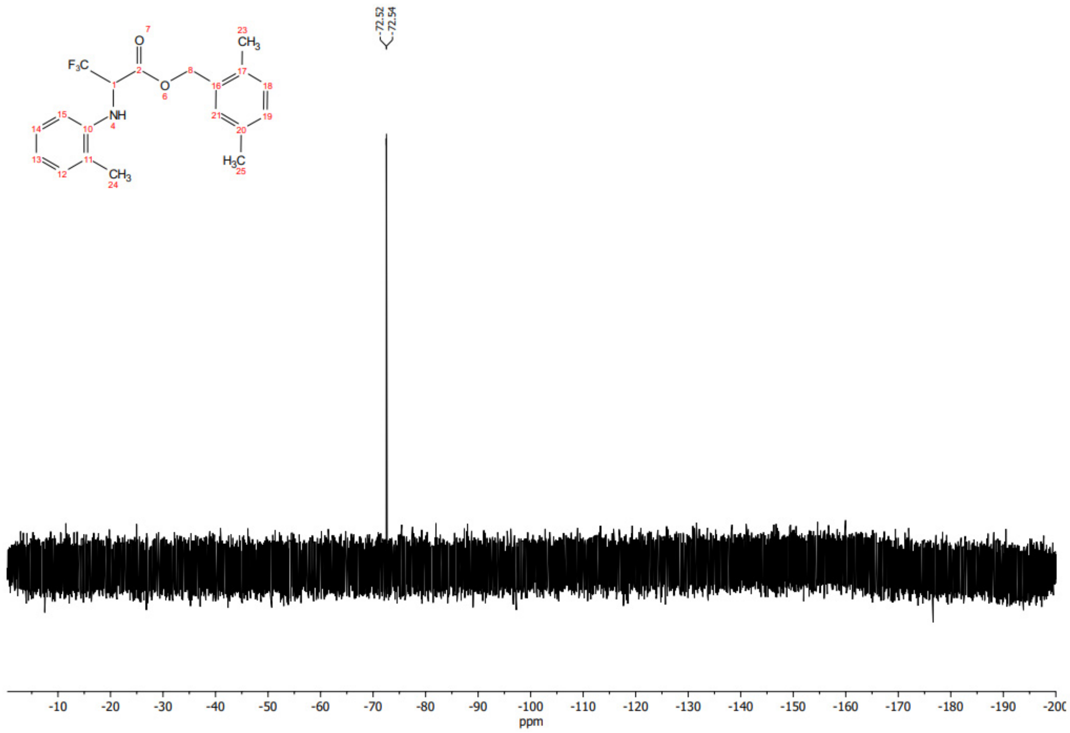

2,5-Dimethylbenzyl 2-((4-chlorophenyl)amino)-3,3,3-trifluoropropanoate (**5f**), ^{1}H NMR (400 MHz, $CDCl_3$):

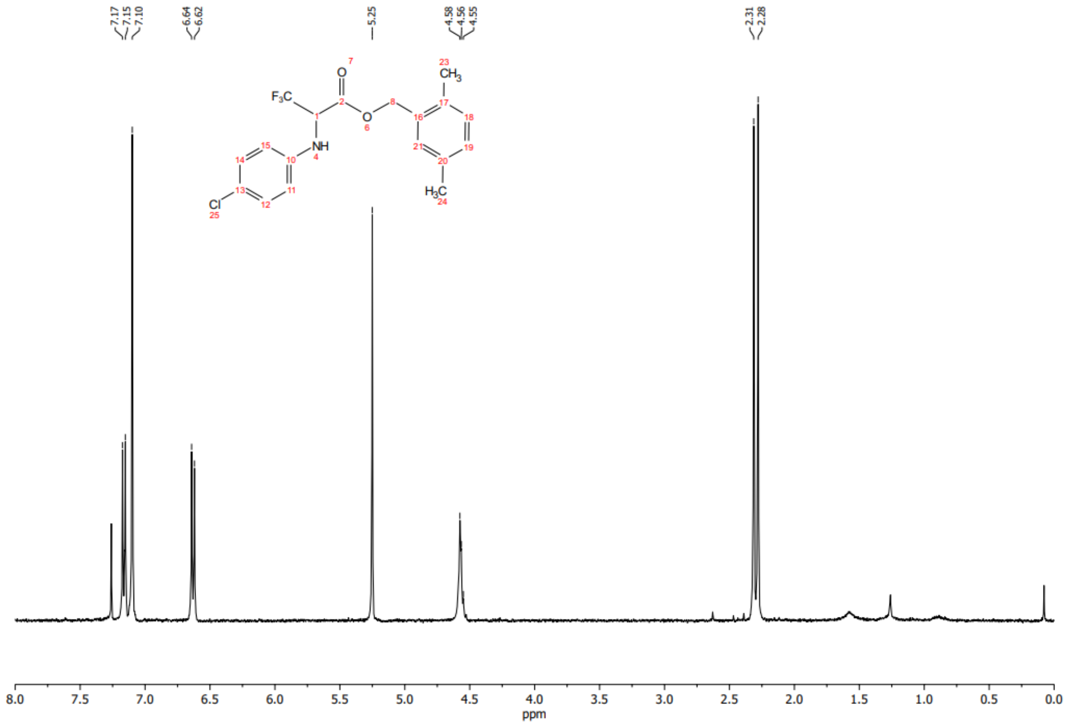

2,5-Dimethylbenzyl 2-((4-chlorophenyl)amino)-3,3,3-trifluoropropanoate (**5f**), ^{13}C NMR (126 MHz, $CDCl_3$):

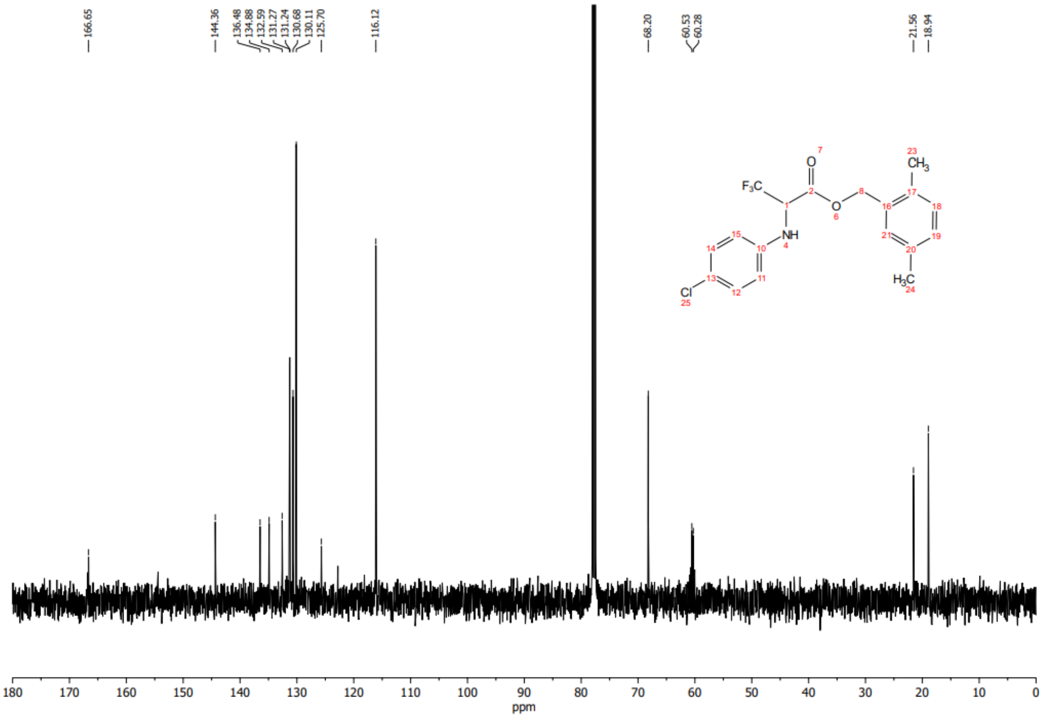

2,5-Dimethylbenzyl 2-((4-chlorophenyl)amino)-3,3,3-trifluoropropanoate (**5f**), ^{19}F NMR (376 MHz, $CDCl_3$):

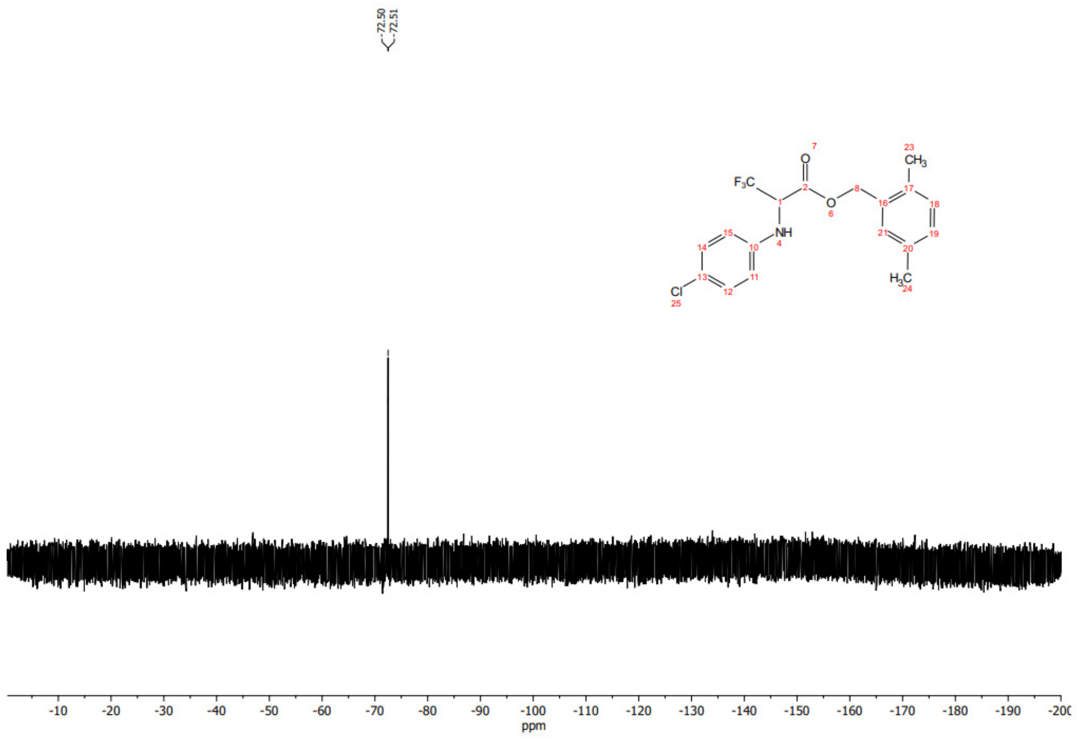

2,5-Dimethylbenzyl 2-((4-bromophenyl)amino)-3,3,3-trifluoropropanoate (**5g**), ^{1}H NMR (400 MHz, $CDCl_3$):

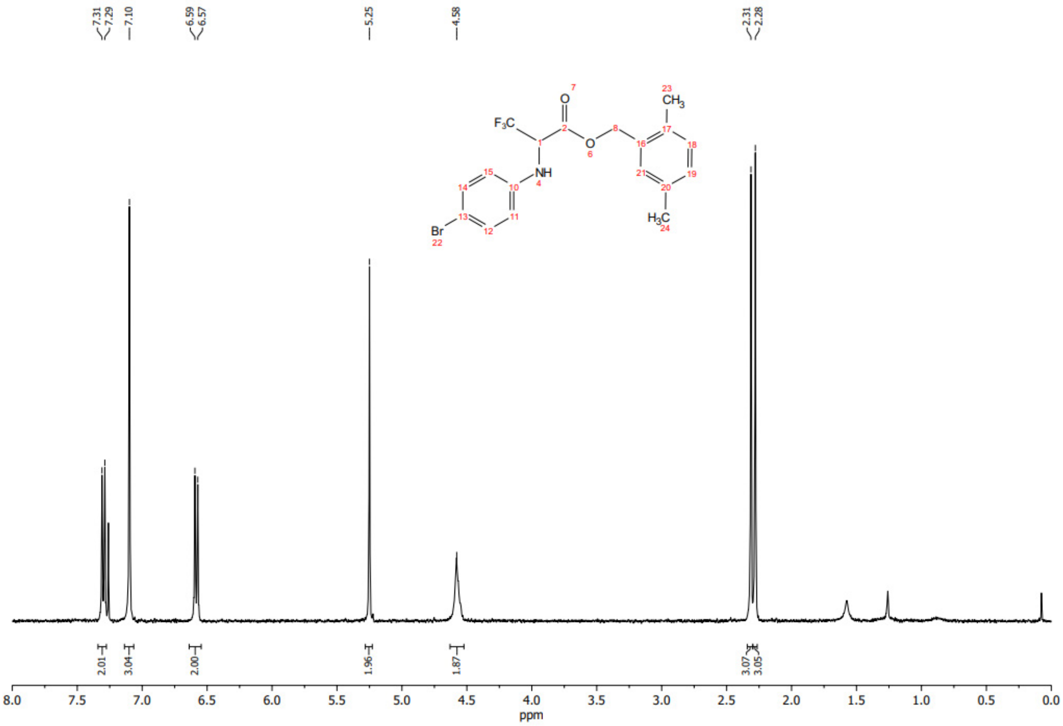

2,5-Dimethylbenzyl 2-((4-bromophenyl)amino)-3,3,3-trifluoropropanoate (**5g**), ^{13}C NMR (126 MHz, $CDCl_3$):

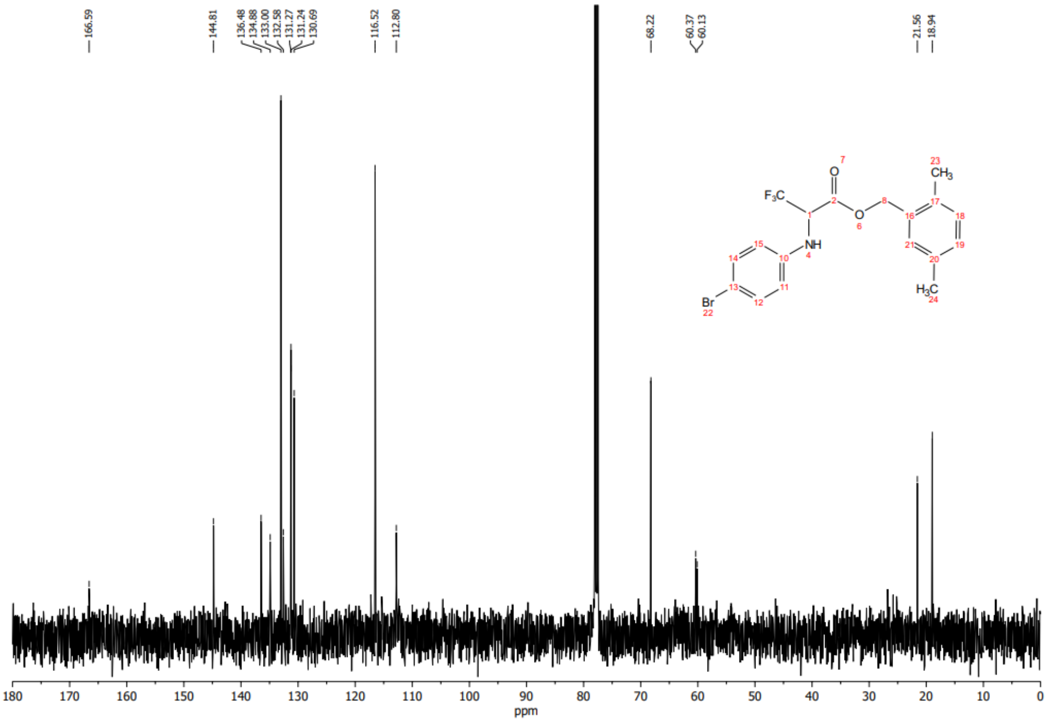

2,5-Dimethylbenzyl 2-((4-bromophenyl)amino)-3,3,3-trifluoropropanoate (**5g**), ^{19}F NMR (376 MHz, $CDCl_3$):

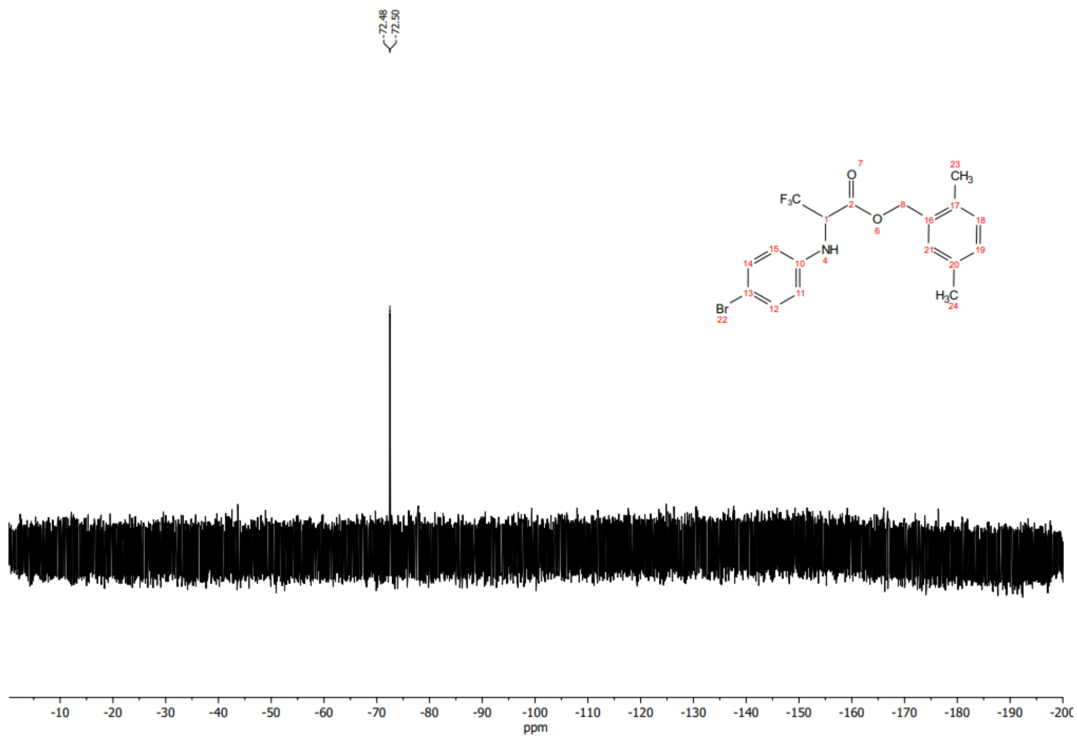

2,5-Dimethylbenzyl 2-((4-(tert-butyl)phenyl)amino)-3,3,3-trifluoropropanoate (**5h**), ^{1}H NMR (400 MHz, $CDCl_3$):

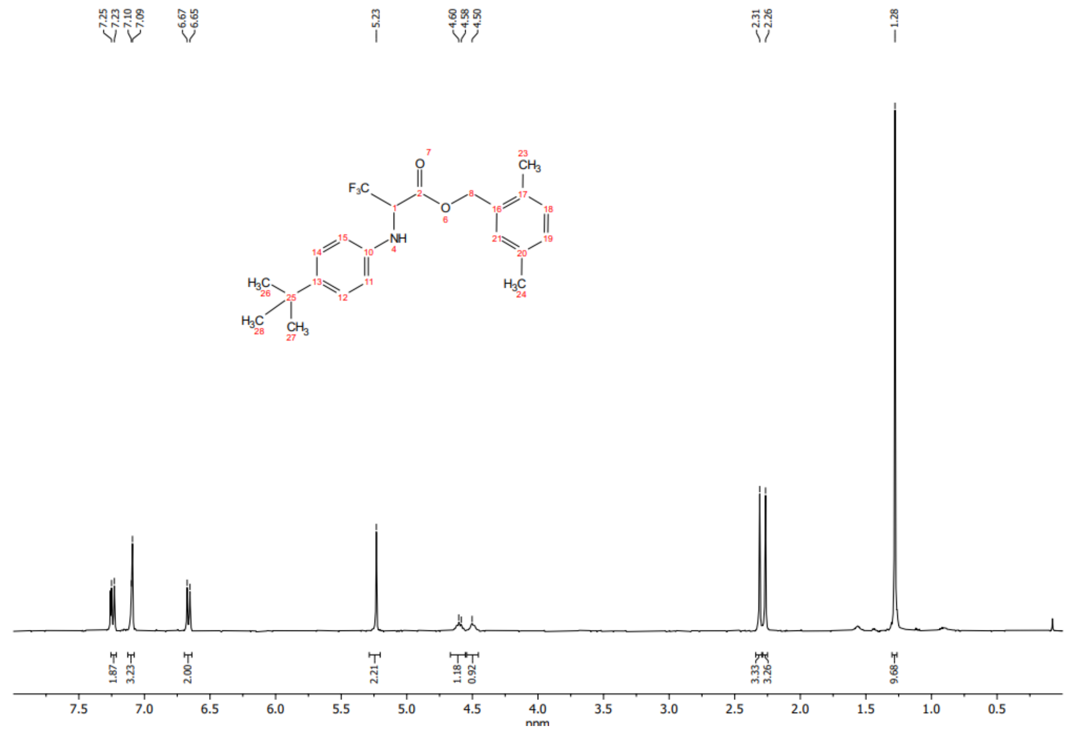

2,5-Dimethylbenzyl 2-((4-(tert-butyl)phenyl)amino)-3,3,3-trifluoropropanoate (**5h**), ^{13}C NMR (126 MHz, $CDCl_3$):

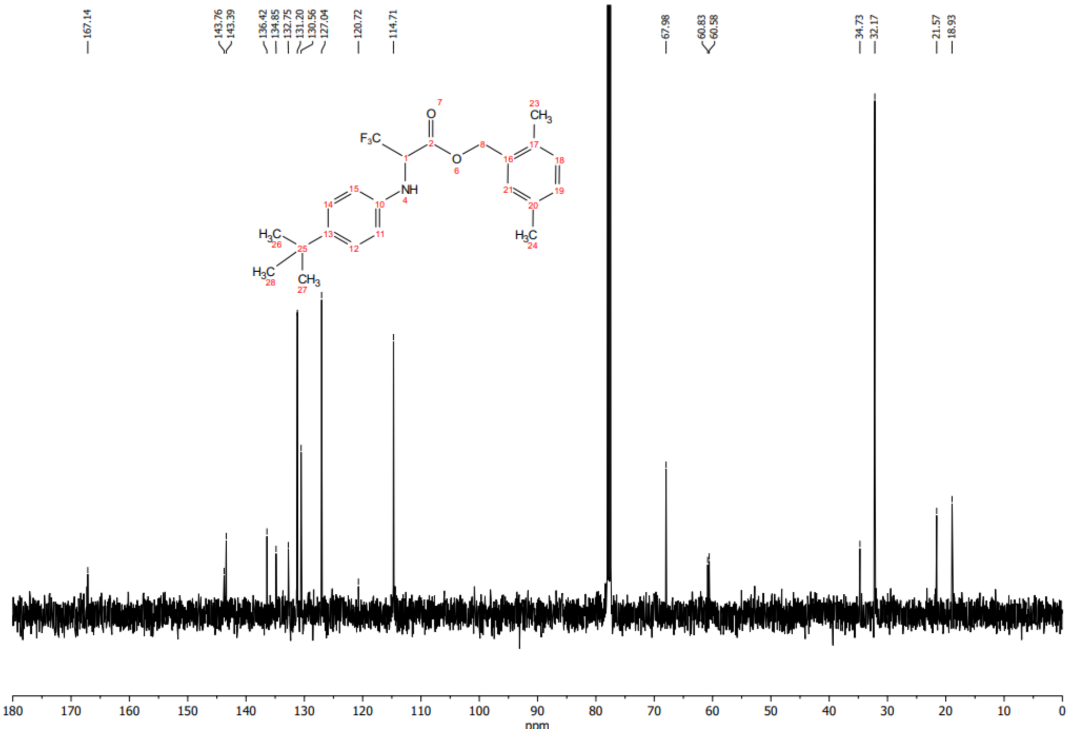

2,5-Dimethylbenzyl 2-((4-(tert-butyl)phenyl)amino)-3,3,3-trifluoropropanoate (**5h**), ^{19}F NMR (376 MHz, $CDCl_3$):

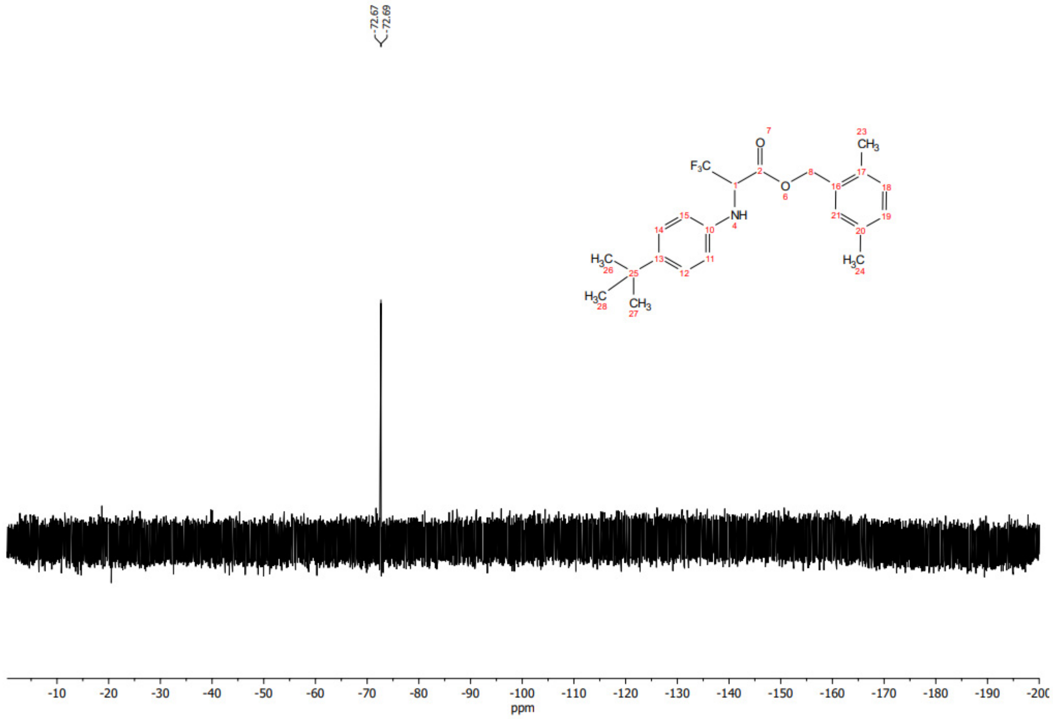

2,5-Dimethylbenzyl 2-((4-acetylphenyl)amino)-3,3,3-trifluoropropanoate (**5i**), ^{1}H NMR (400 MHz, $CDCl_3$):

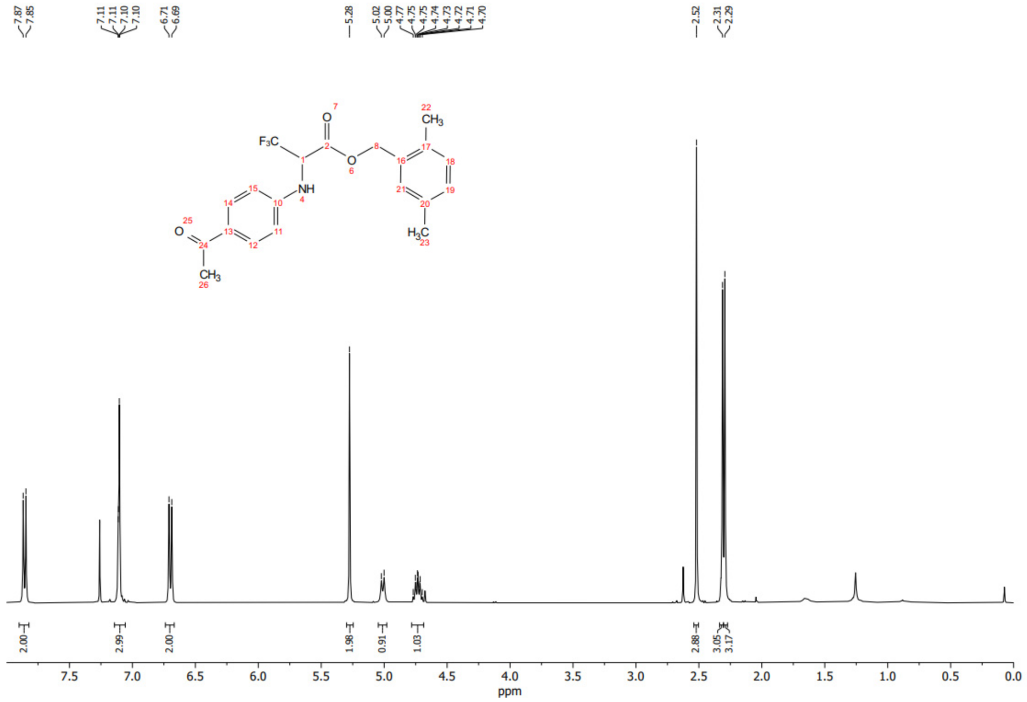

2,5-Dimethylbenzyl 2-((4-acetylphenyl)amino)-3,3,3-trifluoropropanoate (**5i**), ^{13}C NMR (126 MHz, $CDCl_3$):

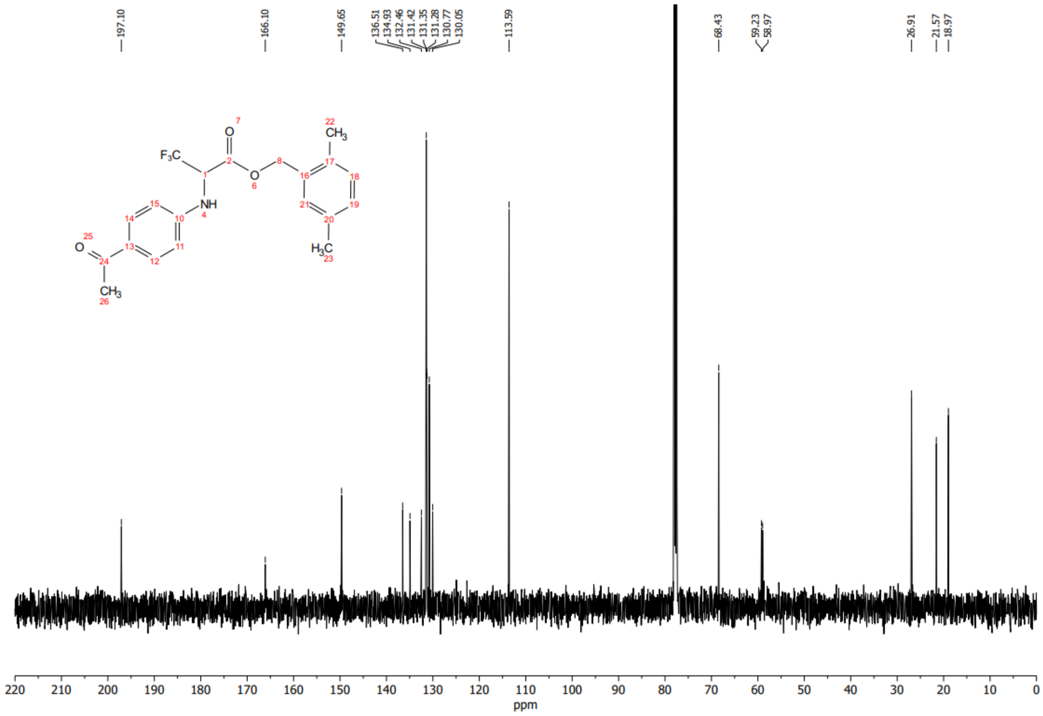

2,5-Dimethylbenzyl 2-((4-acetylphenyl)amino)-3,3,3-trifluoropropanoate (**5i**), ^{19}F NMR (376 MHz, $CDCl_3$):

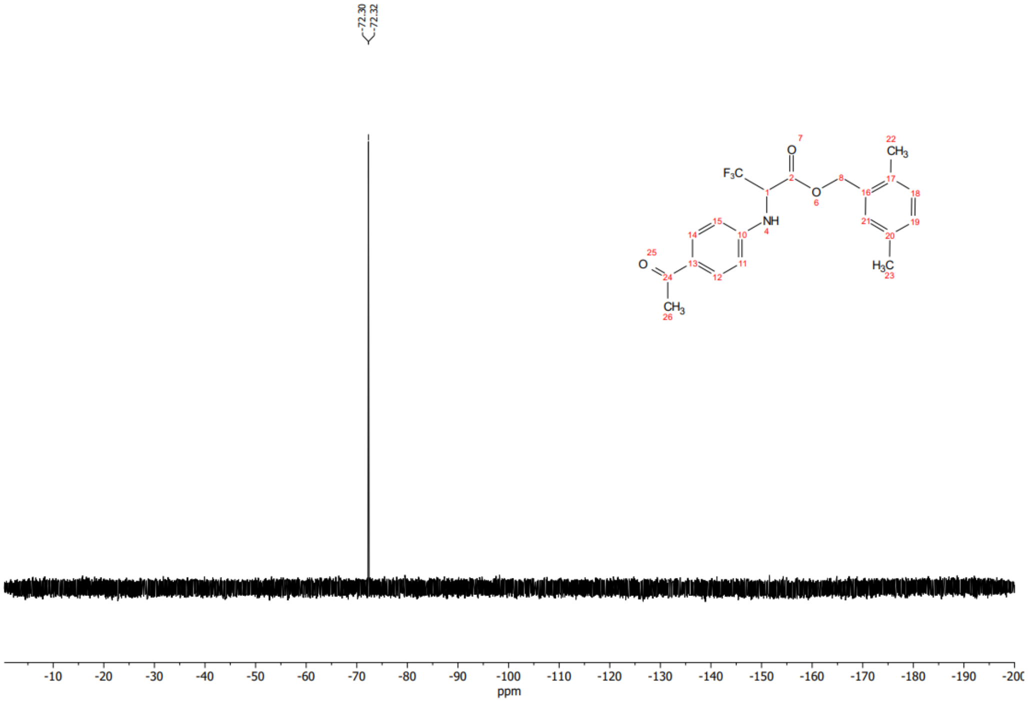

2,5-Dimethylbenzyl 2-((4-cyanophenyl)amino)-3,3,3-trifluoropropanoate (**5j**), ^{1}H NMR (400 MHz, $CDCl_3$):

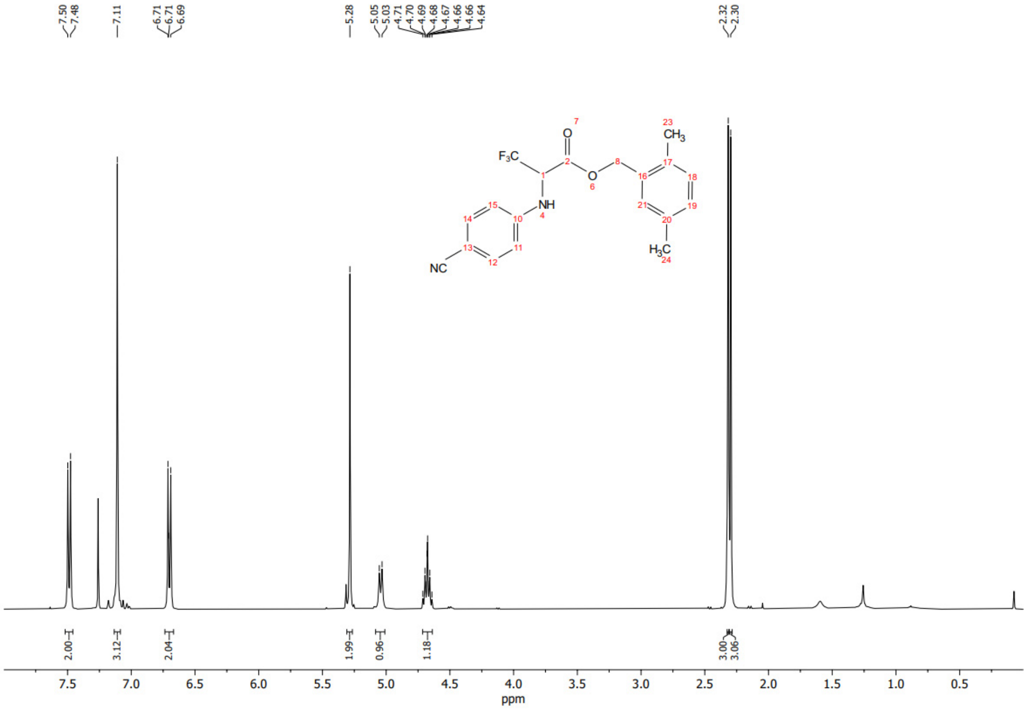

2,5-Dimethylbenzyl 2-((4-cyanophenyl)amino)-3,3,3-trifluoropropanoate (**5j**), ^{13}C NMR (126 MHz, $CDCl_3$):

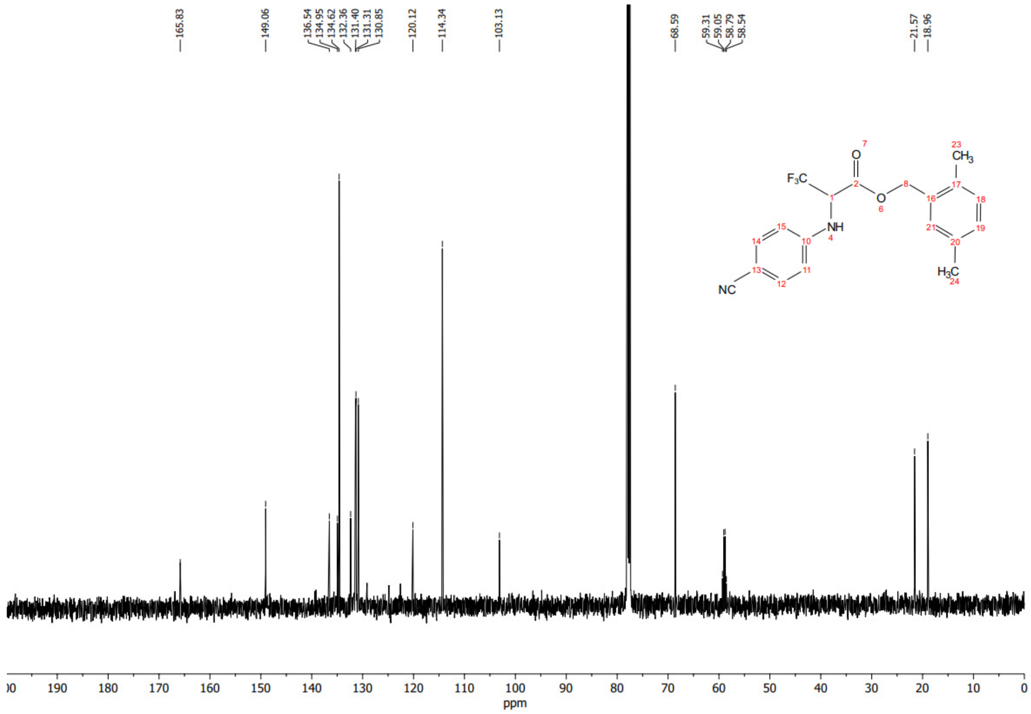

2,5-Dimethylbenzyl 2-((4-cyanophenyl)amino)-3,3,3-trifluoropropanoate (**5j**), ^{19}F NMR (376 MHz, $CDCl_3$):

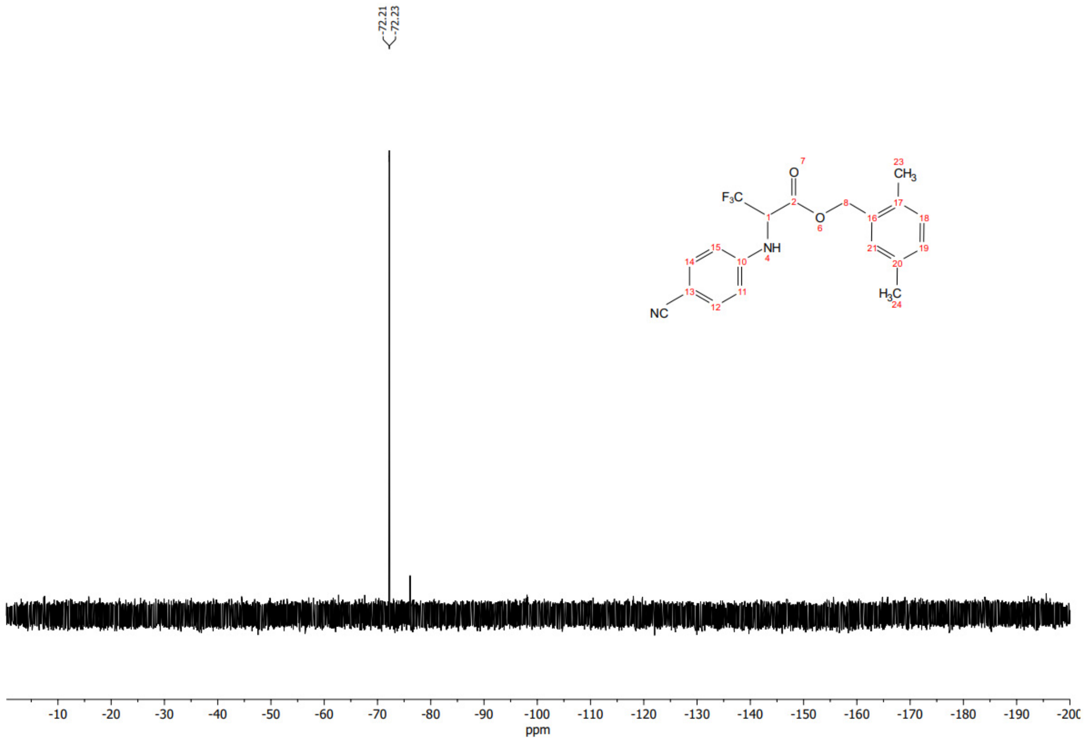

2,5-Dimethylbenzyl 3,3,3-trifluoro-2-((3-(trifluoromethyl)phenyl)amino)propanoate (**5k**), ^{1}H NMR (400 MHz, $CDCl_3$):

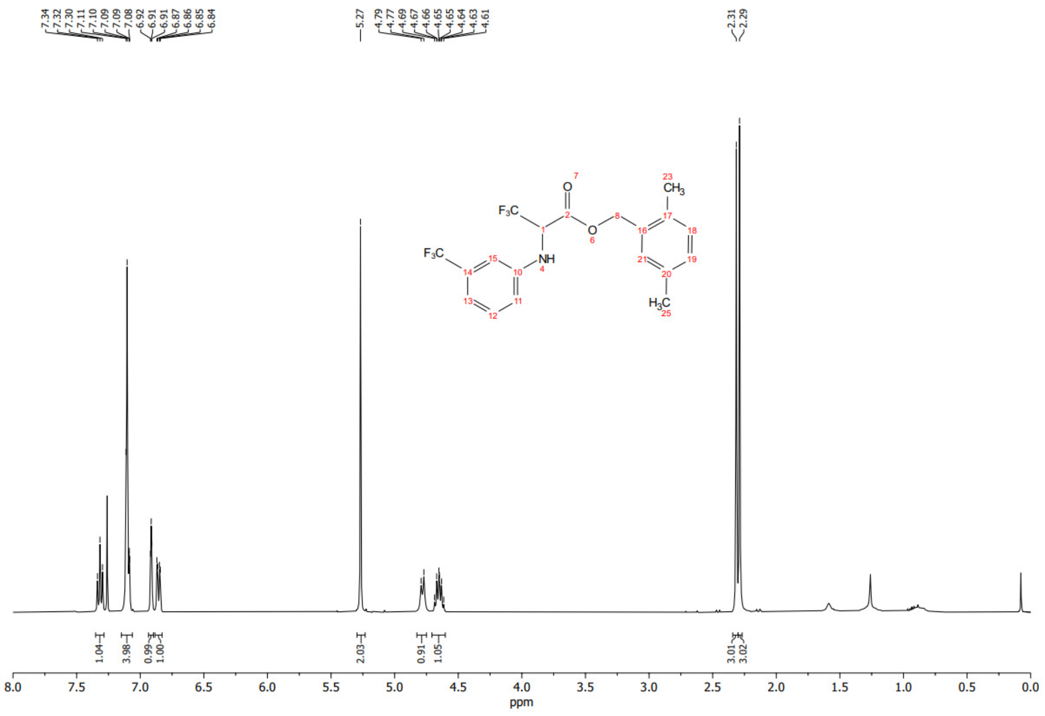

2,5-Dimethylbenzyl 3,3,3-trifluoro-2-((3-(trifluoromethyl)phenyl)amino)propanoate (**5k**), ^{13}C NMR (126 MHz, $CDCl_3$):

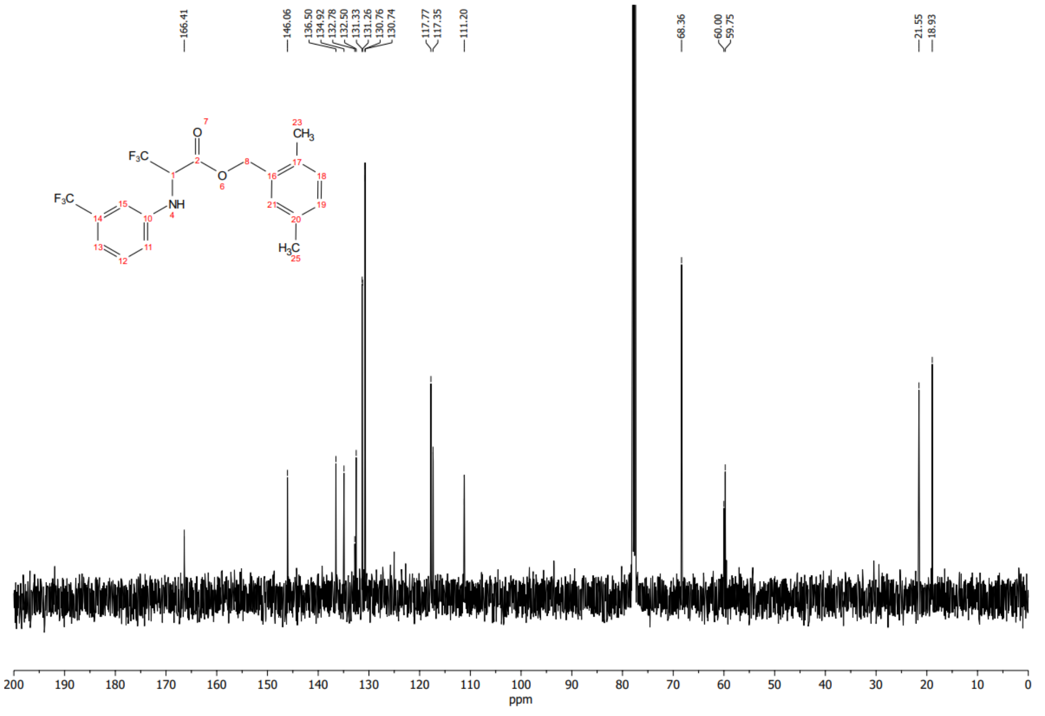

2,5-Dimethylbenzyl 3,3,3-trifluoro-2-((3-(trifluoromethyl)phenyl)amino)propanoate (**5k**), ^{19}F NMR (376 MHz, $CDCl_3$):

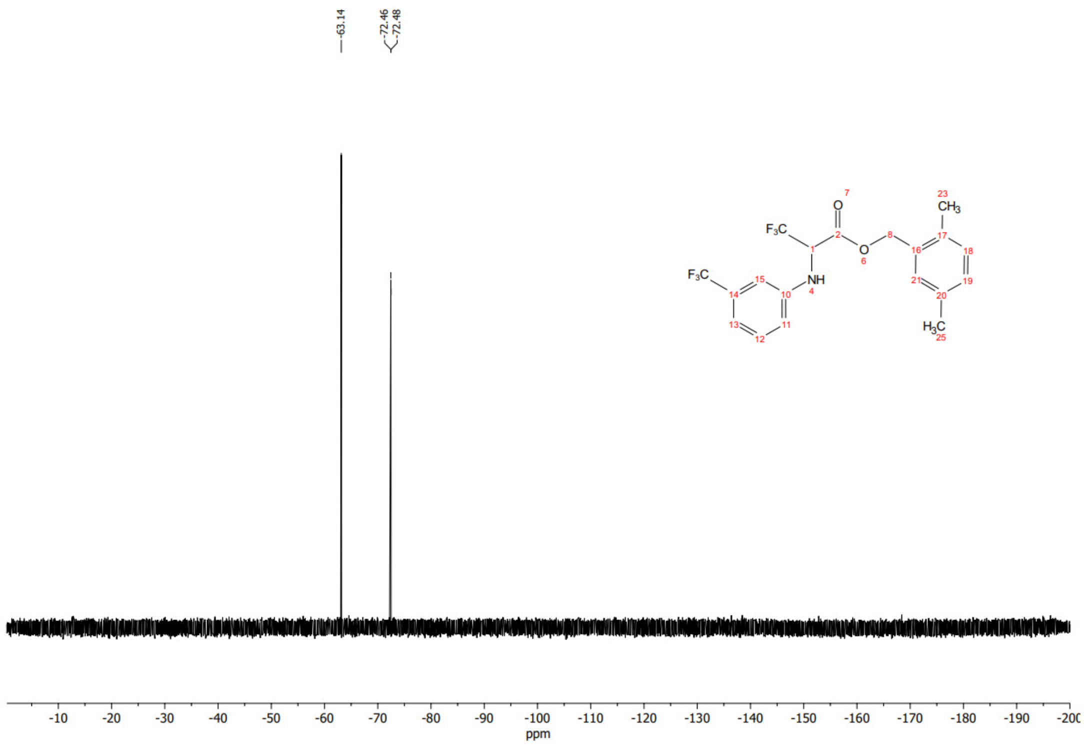

Benzyl 2-amino-3,3,3-trifluoropropanoate (**7**), ^{1}H NMR (400 MHz, $CDCl_3$):

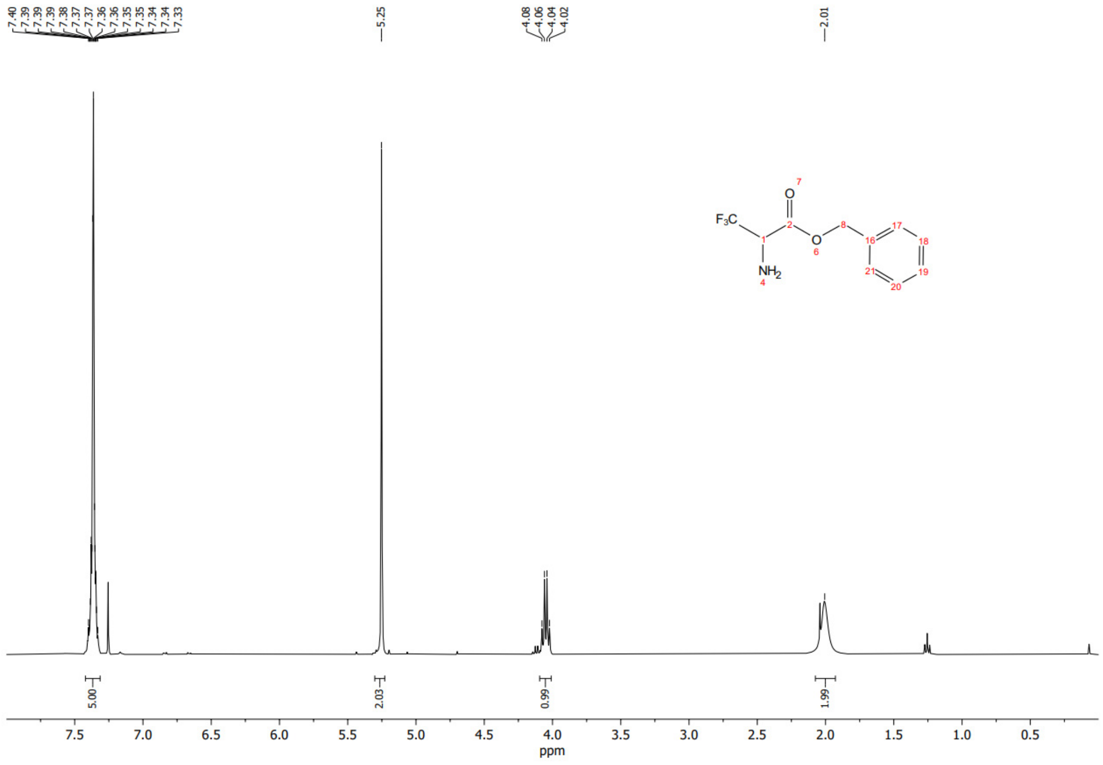

Benzyl 2-amino-3,3,3-trifluoropropanoate (**7**), ^{13}C NMR (126 MHz, CDCl$_3$):

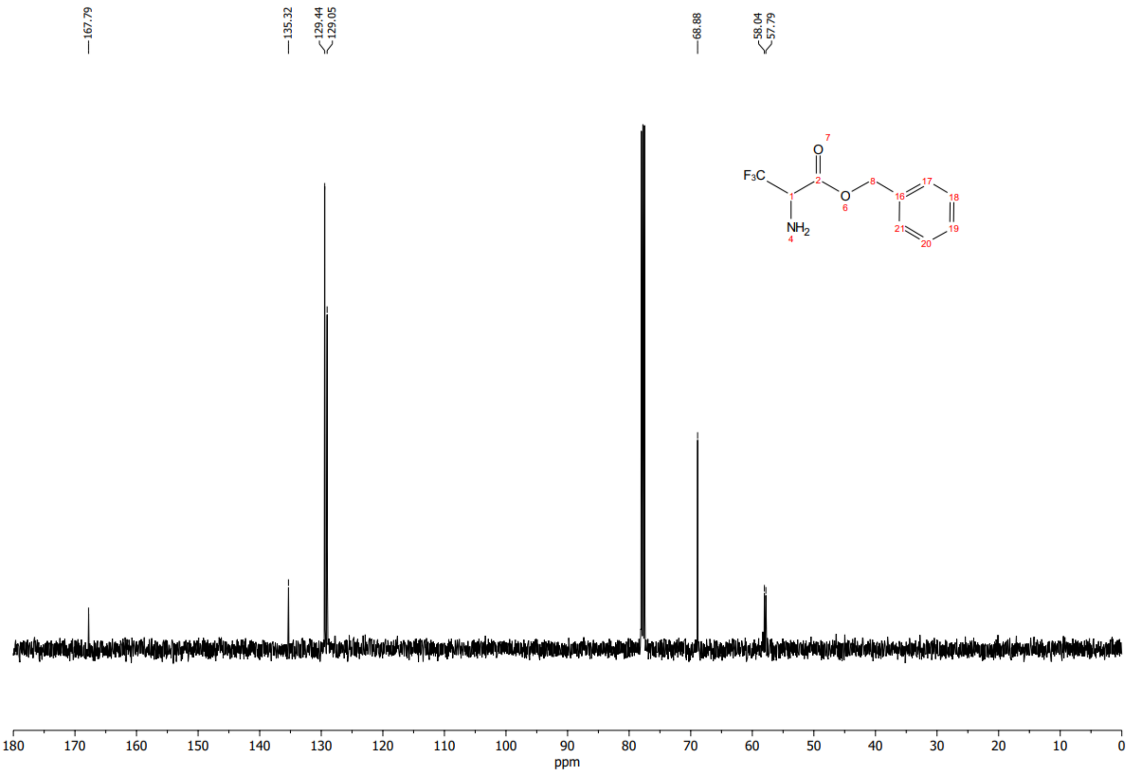

Benzyl 2-amino-3,3,3-trifluoropropanoate (**7**), ^{19}F NMR (376 MHz, CDCl$_3$):

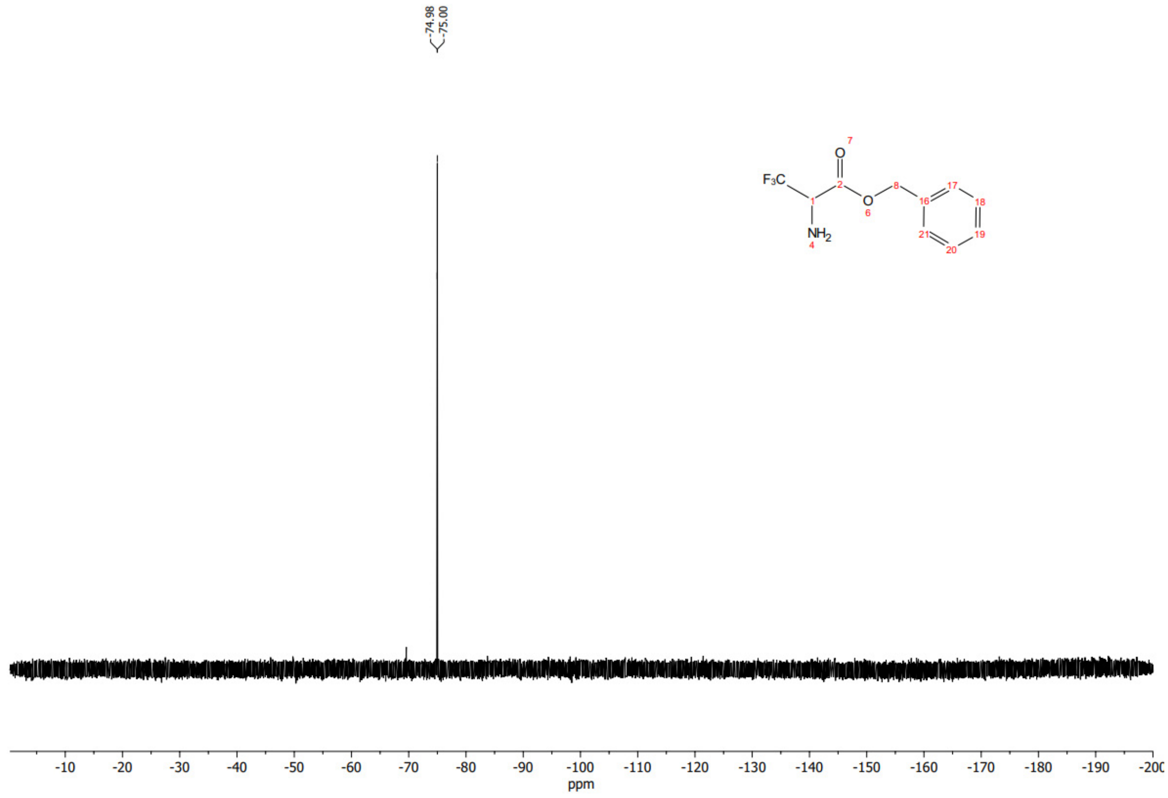

3,3,3-trifluoro-2-((4-methoxyphenyl)amino)propan-1-ol (**8**), ^{1}H NMR (400 MHz, $CDCl_3$):

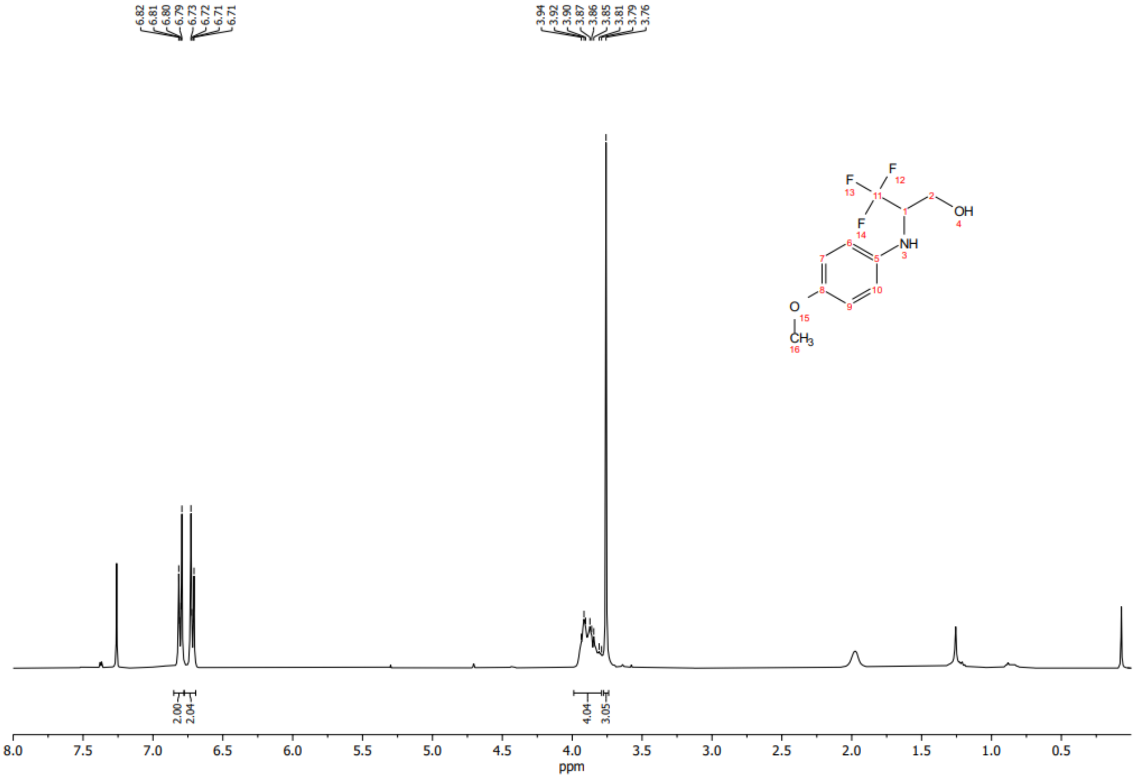

3,3,3-trifluoro-2-((4-methoxyphenyl)amino)propan-1-ol (**8**), ^{13}C NMR (126 MHz, $CDCl_3$):

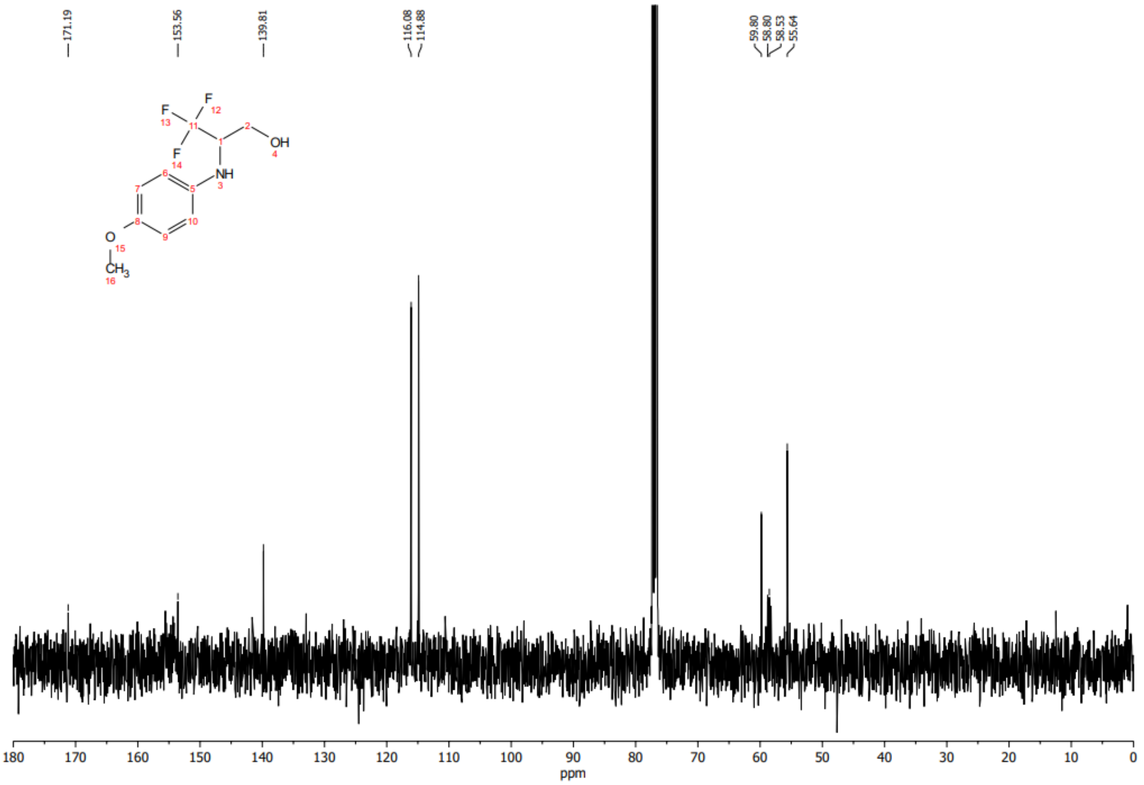

3,3,3-trifluoro-2-((4-methoxyphenyl)amino)propan-1-ol (**8**), ^{19}F NMR (376 MHz, $CDCl_3$):

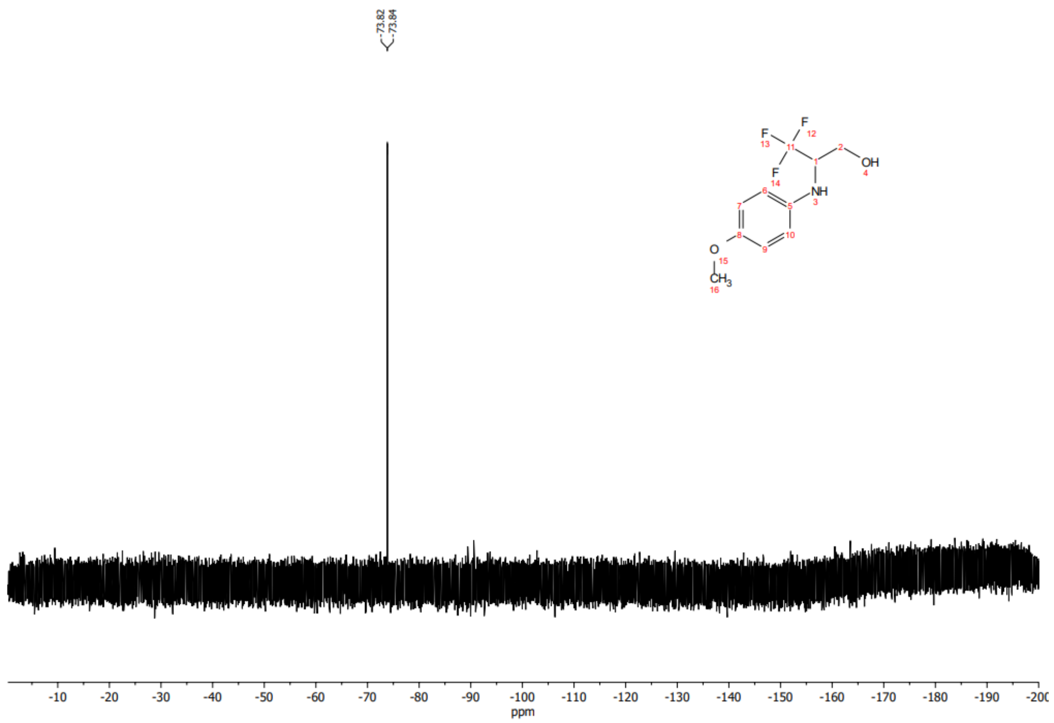

3,3,3-Trifluoro-2-((4-methoxyphenyl)amino)-1,1-diphenylpropan-1-ol (**9**), ^{1}H NMR (400 MHz, $CDCl_3$):

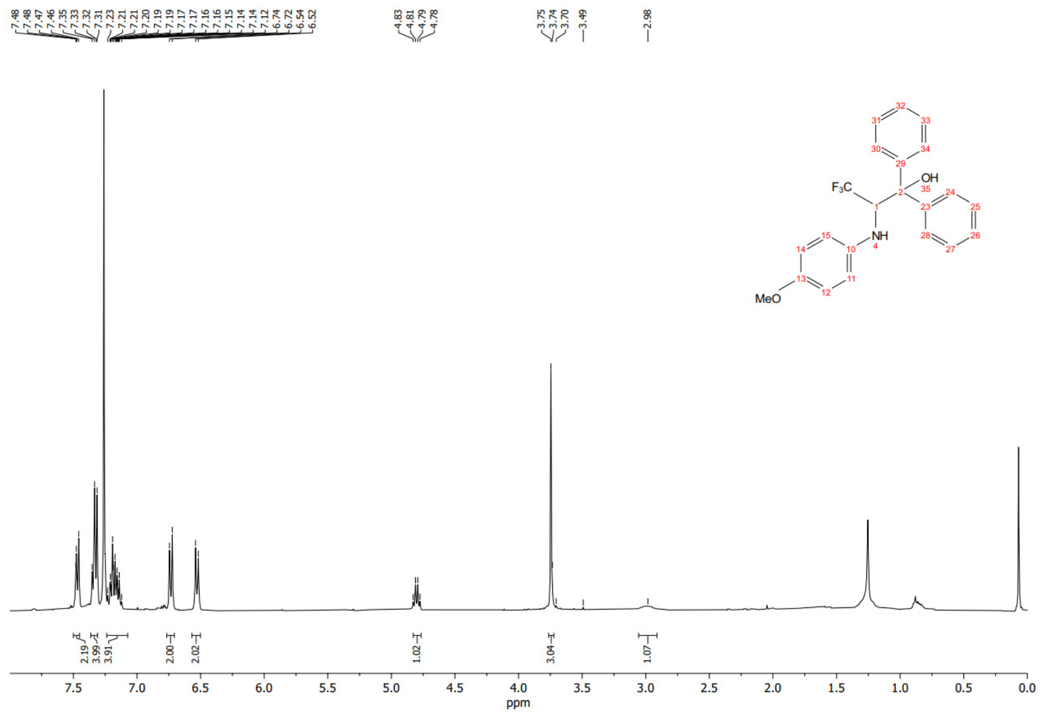

3,3,3-Trifluoro-2-((4-methoxyphenyl)amino)-1,1-diphenylpropan-1-ol (**9**), ^{13}C NMR (126 MHz, $CDCl_3$):

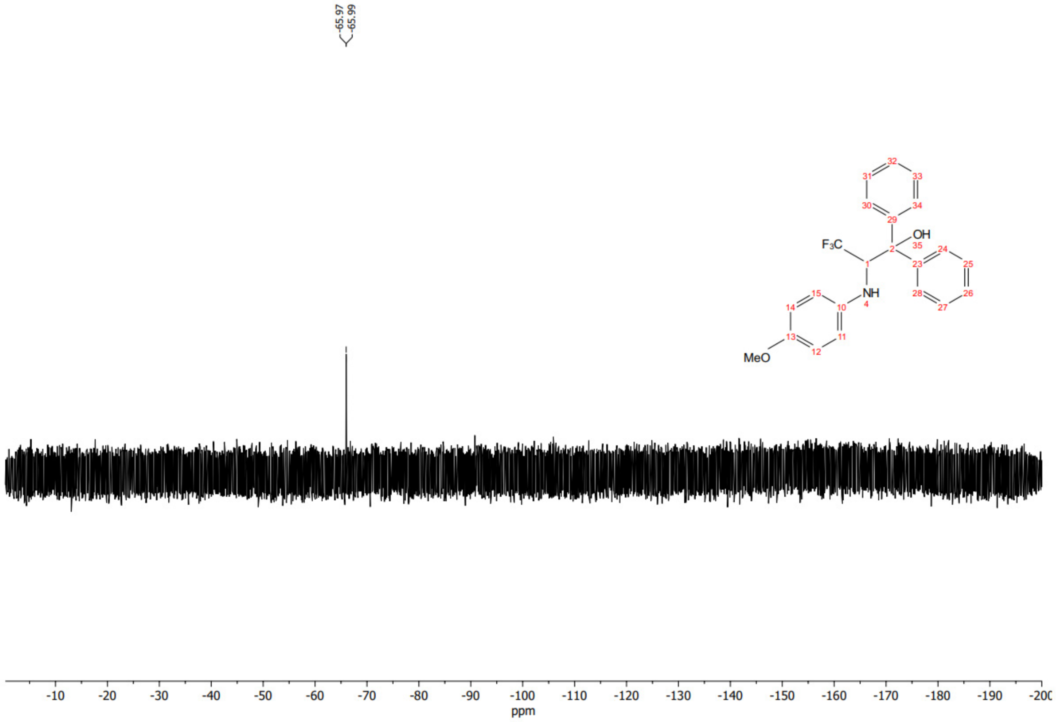

3,3,3-Trifluoro-2-((4-methoxyphenyl)amino)-1,1-diphenylpropan-1-ol (**9**), ^{19}F NMR (376 MHz, $CDCl_3$):

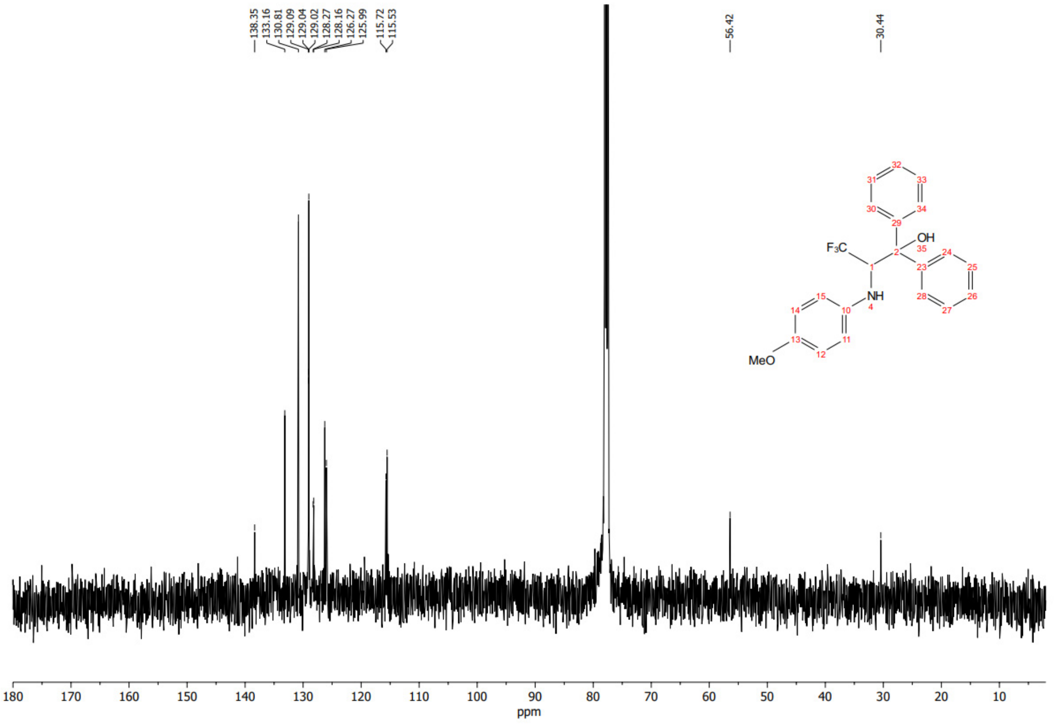

N-(3-Bromo-1,1,1-trifluoropropan-2-yl)-4-methoxyaniline (**10**), ^{1}H NMR (400 MHz, $CDCl_3$):

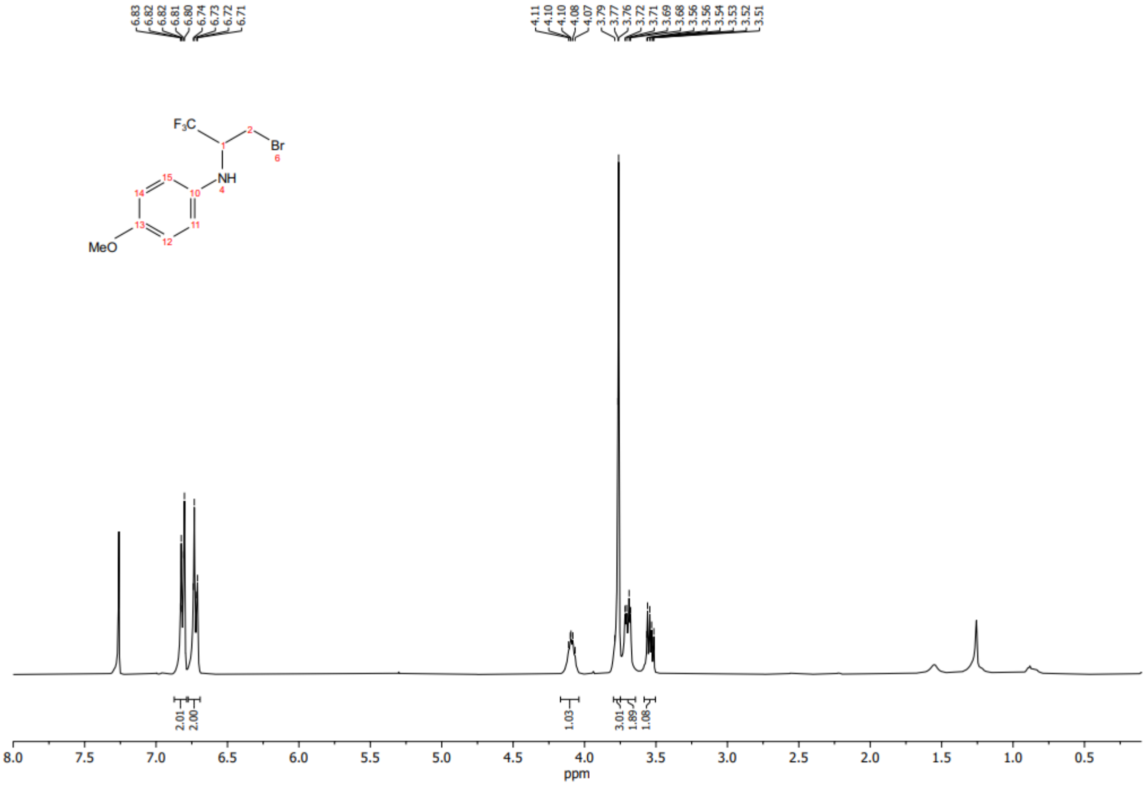

N-(3-Bromo-1,1,1-trifluoropropan-2-yl)-4-methoxyaniline (**10**), ^{13}C NMR (126 MHz, $CDCl_3$):

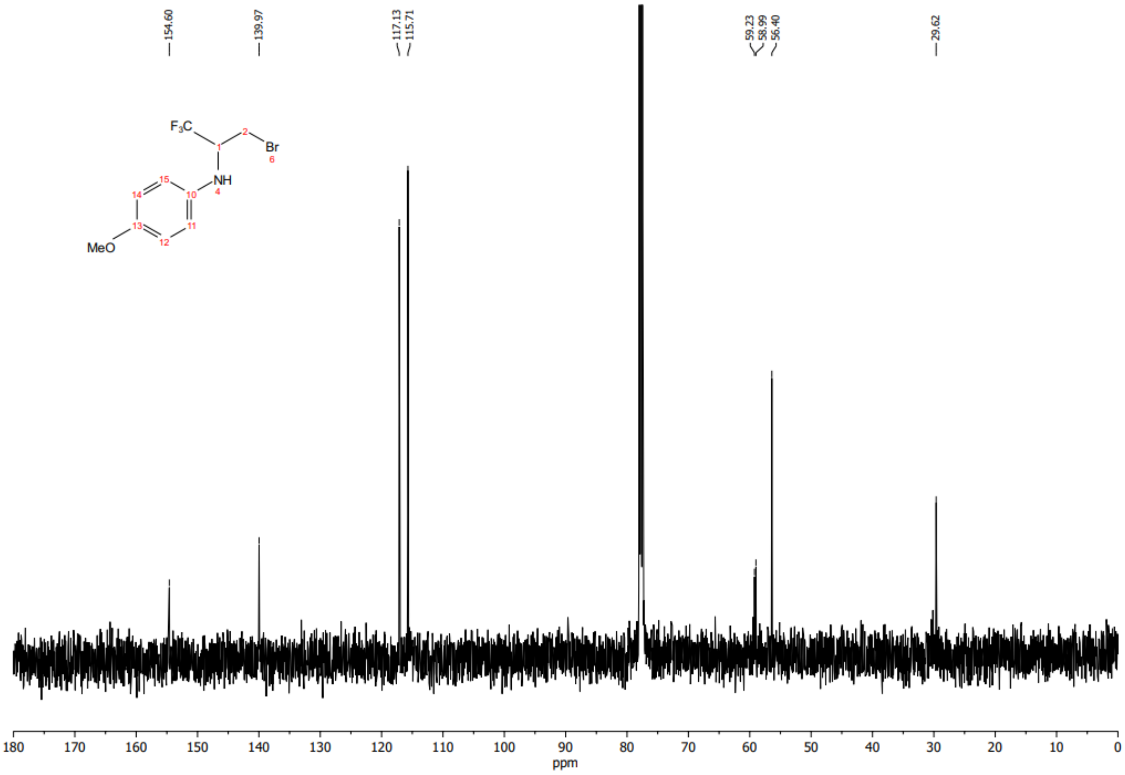

N-(3-Bromo-1,1,1-trifluoropropan-2-yl)-4-methoxyaniline (**10**), ^{19}F NMR (376 MHz, $CDCl_3$):

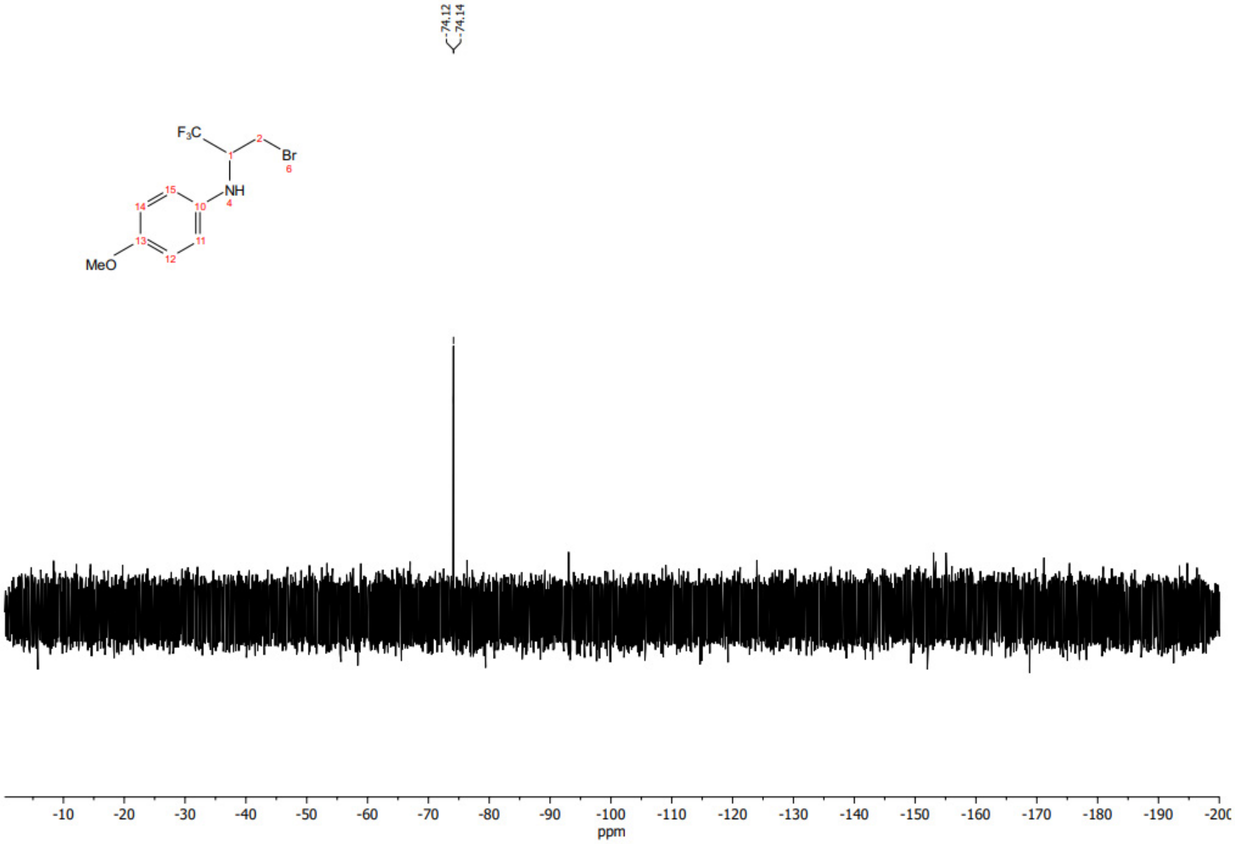

Atom coordinates of the DFT-generated Fe-ylide reaction intermediate models

R(+)

Fe	-0.461819	-1.315394	0.030739	H	0.078268	-4.346944	1.123234
N	0.159901	-1.569940	1.946379	H	-3.269685	-2.688337	-0.753262
N	-2.150992	-0.442874	0.674571	H	-3.795669	-5.122072	-0.269518
N	-1.100703	-1.165617	-1.863598	C	0.678327	0.719393	-0.063270
N	1.222729	-2.282016	-0.593682	C	0.901180	1.301180	-1.424818
C	-3.160594	0.049887	-0.091993	F	1.527118	2.518816	-1.417235
C	1.290498	-2.201061	2.384018	F	-0.220517	1.497554	-2.120643
C	-2.535368	-0.271361	1.967814	F	1.704886	0.505838	-2.165671
C	-0.549510	-1.246739	3.065125	C	0.024958	1.610403	0.899039
C	-2.248018	-0.576821	-2.295005	O	0.321704	1.667771	2.080296
C	2.211374	-2.832522	0.179140	O	-0.952936	2.335396	0.358658
C	-0.475437	-1.642086	-2.972012	C	-1.740316	3.129103	1.240337
C	1.517847	-2.617335	-1.883768	H	-1.137046	3.965675	1.607135
C	-4.216147	0.573507	0.743487	H	-2.040087	2.524299	2.099101
C	1.315028	-2.238734	3.827152	C	-2.951691	3.622042	0.495993
C	-3.832472	0.366040	2.023939	C	-3.980964	4.224078	1.217625
C	0.170558	-1.651353	4.248383	C	-3.079861	3.478948	-0.881630
C	-2.350255	-0.671885	-3.733625	C	-5.125959	4.673166	0.573738
C	3.183027	-3.494411	-0.654945	C	-4.228607	3.925812	-1.526261
C	-1.250885	-1.337939	-4.154415	C	-5.254747	4.522210	-0.803770
C	2.750265	-3.363613	-1.933178	H	-3.891500	4.329227	2.294779
H	-5.116120	1.046707	0.377592	H	-2.291966	2.991086	-1.440588
H	2.105556	-2.675688	4.420407	H	-5.921586	5.135067	1.147705
H	-4.356922	0.623352	2.933234	H	-4.320992	3.802296	-2.599818
H	-0.173787	-1.502812	5.261879	H	-6.150179	4.867243	-1.308480
H	-3.165755	-0.278084	-4.323513	N	1.982048	0.282857	0.535535
H	4.066146	-4.000868	-0.292506	H	2.165405	-0.646347	0.142470
H	-0.971383	-1.606073	-5.163372	H	1.736509	0.125088	1.520091
H	3.206308	-3.743740	-2.836278	C	3.206454	1.084743	0.452878
C	-3.203038	0.009415	-1.477870	C	4.219213	0.648892	-0.377768
C	2.259632	-2.773297	1.567528	C	3.346832	2.243908	1.207224
C	0.735101	-2.319828	-2.988465	C	5.399424	1.378157	-0.476987
C	-1.797414	-0.644918	3.081336	C	4.516132	2.970414	1.111296
H	-4.068805	0.450685	-1.959178	C	5.549363	2.547186	0.267031
H	3.107006	-3.241584	2.055138	H	4.096792	-0.258805	-0.959524
H	1.103695	-2.646726	-3.954601	H	2.544066	2.567905	1.857009
H	-2.236319	-0.447819	4.053199	H	6.181451	1.024348	-1.134373
C	-2.607009	-3.390510	-0.274607	H	4.654968	3.877913	1.685439
C	-0.908350	-4.292728	0.694822	O	6.647470	3.331977	0.245465
C	-1.850241	-5.270642	0.584595	C	7.720870	2.962259	-0.602265
N	-1.393258	-3.125699	0.154909	H	8.482099	3.728048	-0.470705
N	-2.923860	-4.678651	-0.034374	H	7.408068	2.938327	-1.651070
H	-1.854803	-6.304968	0.881648	H	8.134033	1.988635	-0.319198

R(−)

Fe	-1.510576	-0.262961	0.224698
N	-0.576922	1.188319	1.295597
N	-2.193672	1.098448	-1.072905
N	-2.501557	-1.704900	-0.751550
N	-0.855507	-1.623901	1.604019
C	-2.946669	0.866514	-2.181443
C	0.130644	1.055055	2.457753
C	-2.012301	2.443255	-1.007956
C	-0.641013	2.526908	1.045383
C	-3.231624	-1.565643	-1.889907
C	-0.126715	-1.386643	2.740014
C	-2.590406	-3.014444	-0.398699
C	-1.188099	-2.948992	1.635498
C	-3.236676	2.109628	-2.858647
C	0.564851	2.350078	2.929060
C	-2.660849	3.090629	-2.126367
C	0.082188	3.261929	2.054702
C	-3.798694	-2.835790	-2.281251
C	0.046134	-2.609004	3.482944
C	-3.403471	-3.735877	-1.352226
C	-0.615434	-3.575969	2.799373
H	-3.810764	2.198337	-3.769838
H	1.157433	2.517500	3.817078
H	-2.663258	4.156119	-2.307997
H	0.211845	4.334796	2.061199
H	-4.414930	-2.995709	-3.154502
H	0.590012	-2.695600	4.412709
H	-3.626236	-4.792144	-1.301013
H	-0.725934	-4.620104	3.055489
C	-3.425495	-0.374494	-2.574429
C	0.361749	-0.142270	3.124685
C	-1.988775	-3.596313	0.707907
C	-1.307255	3.113297	-0.017579
H	-4.024451	-0.412463	-3.477610
H	0.938789	-0.097327	4.041290
H	-2.159605	-4.656281	0.861113
H	-1.258686	4.194104	-0.089835
C	-4.398162	0.099725	0.932098
C	-3.230281	0.212665	2.738468
C	-4.529393	0.383182	3.111139
N	-3.163000	0.036647	1.377241
N	-5.257878	0.308818	1.949154
H	-4.989376	0.546266	4.070234
H	-2.344656	0.204097	3.351196
H	-4.699091	0.000415	-0.097890
H	-6.256666	0.394001	1.864616
C	0.440673	-0.592232	-1.044604
C	0.506594	-1.858092	-1.840809
F	1.656116	-1.999898	-2.571146
F	-0.481189	-1.987636	-2.723945
F	0.483589	-2.940914	-1.029699
C	0.649916	0.603441	-1.865418
O	0.277621	0.771178	-3.001826
O	1.310487	1.566620	-1.171972
C	1.480407	2.828116	-1.820528
H	0.523434	3.156373	-2.229108
H	2.179059	2.702675	-2.654107
C	2.005266	3.807994	-0.808483
C	1.519707	5.112462	-0.789590
C	2.979181	3.435644	0.115728
C	1.995775	6.031825	0.138145
C	3.451505	4.349534	1.048015
C	2.960774	5.650626	1.062954
H	0.752855	5.408264	-1.498480
H	3.358131	2.419683	0.119278
H	1.605383	7.043298	0.143994
H	4.202066	4.043842	1.768169
H	3.327601	6.363255	1.792927
N	1.403751	-0.664289	0.103187
H	0.919829	-1.235437	0.806877
H	1.397136	0.285069	0.490005
C	2.782299	-1.149299	-0.023503
C	3.128363	-2.294169	0.665971
C	3.719032	-0.471136	-0.794412
C	4.428449	-2.784120	0.597484
C	5.010459	-0.951679	-0.864832
C	5.376349	-2.111511	-0.171333
H	2.387237	-2.818171	1.261529
H	3.437508	0.418216	-1.341826
H	4.679732	-3.683789	1.141774
H	5.759990	-0.445292	-1.459868
O	6.662165	-2.495997	-0.309640
C	7.086997	-3.673266	0.355386
H	8.137588	-3.796013	0.101899
H	6.526256	-4.547944	0.010793
H	6.985444	-3.574797	1.441019

S(+)

Fe	1.529219	-0.223825	0.203584
N	2.190488	1.164587	-1.075025
N	2.532326	-1.657412	-0.774564
N	0.894278	-1.615782	1.564685
N	0.580536	1.215043	1.276234
C	2.590105	-2.982814	-0.473837
C	1.940269	2.499978	-1.043728
C	3.308382	-1.482121	-1.875786
C	2.995137	0.951592	-2.149100
C	1.188142	-2.951624	1.558441
C	0.564956	2.551184	1.004558
C	0.224617	-1.377861	2.736394
C	-0.061584	1.065743	2.473319
C	3.429079	-3.676481	-1.425453
C	2.599885	3.160084	-2.148584
C	3.875911	-2.743207	-2.296291
C	3.254456	2.197045	-2.835968
C	0.644402	-3.583344	2.732615
C	-0.148195	3.265712	2.035048
C	0.048574	-2.607888	3.464418
C	-0.534057	2.345860	2.948656
H	3.634577	-4.737374	-1.410923
H	2.556868	4.221031	-2.350113
H	4.525749	-2.875175	-3.149617
H	3.861873	2.298474	-3.723907
H	0.730787	-4.635529	2.963960
H	-0.333673	4.330402	2.030769
H	-0.456906	-2.696363	4.415431
H	-1.088875	2.498655	3.863331
C	1.961890	-3.593312	0.602657
C	1.185600	3.152830	-0.078499
C	-0.234671	-0.133427	3.151919
C	3.521036	-0.274838	-2.524530
H	2.107391	-4.661438	0.720486
H	1.081539	4.228096	-0.170888
H	-0.766538	-0.094247	4.095913
H	4.156865	-0.291538	-3.402920
C	3.935697	-0.848808	1.894916
C	3.706753	1.288274	1.751312
C	4.808817	1.072219	2.522257
N	3.172247	0.081977	1.367187
N	4.938807	-0.292782	2.603267
H	5.494203	1.745996	3.006298
H	3.262970	2.221030	1.446480
H	3.795195	-1.911750	1.786470
H	5.655401	-0.795292	3.099150
C	-0.426128	-0.560927	-1.056573
C	-0.475248	-1.809154	-1.881876
F	-1.619428	-1.944563	-2.622043
F	0.518877	-1.904895	-2.762824
F	-0.445482	-2.911275	-1.098834
C	-0.674249	0.640263	-1.859349
O	-0.294079	0.839310	-2.987861
O	-1.386315	1.563135	-1.162191
C	-1.604719	2.825495	-1.794316
H	-0.662531	3.192612	-2.203642
H	-2.303063	2.684788	-2.625691
C	-2.158793	3.773919	-0.767817
C	-1.728721	5.097674	-0.743368
C	-3.105140	3.353226	0.164309
C	-2.231877	5.988385	0.198020
C	-3.604665	4.238756	1.109757
C	-3.168710	5.559193	1.130688
H	-0.984578	5.432190	-1.459266
H	-3.441205	2.322309	0.163250
H	-1.884480	7.015377	0.208022
H	-4.333311	3.895768	1.835575
H	-3.556385	6.249605	1.871126
N	-1.374509	-0.672796	0.101669
H	-0.858546	-1.227859	0.796964
H	-1.399285	0.274992	0.491189
C	-2.734330	-1.210229	-0.010554
C	-3.025444	-2.372088	0.676088
C	-3.707412	-0.565087	-0.764445
C	-4.306216	-2.912026	0.622770
C	-4.980033	-1.095368	-0.819728
C	-5.290654	-2.272315	-0.128213
H	-2.255248	-2.870390	1.257021
H	-3.467824	0.337331	-1.310412
H	-4.513856	-3.824100	1.164752
H	-5.757042	-0.615326	-1.401187
O	-6.562535	-2.705576	-0.250708
C	-6.932445	-3.901402	0.413928
H	-7.980935	-4.063508	0.174191
H	-6.343123	-4.752159	0.057321
H	-6.819686	-3.804036	1.498575

S(−)

Fe	-0.459670	1.297541	0.043490
N	-2.164009	0.448873	0.677870
N	-1.072993	1.177811	-1.861407
N	1.241772	2.241547	-0.571261
N	0.138993	1.521488	1.970029
C	-0.403665	1.588185	-2.971878
C	-2.535042	0.202227	1.963430
C	-2.256255	0.668607	-2.294705
C	-3.200566	0.042487	-0.101888
C	1.591862	2.526446	-1.860579
C	-0.547073	1.137558	3.084607
C	2.188787	2.834203	0.221796
C	1.237761	2.200192	2.416365
C	-1.187086	1.318746	-4.157310
C	-3.851047	-0.397120	1.998978
C	-2.338614	0.748530	-3.735814
C	-4.262533	-0.498388	0.715081
C	2.821093	3.277911	-1.885964
C	0.159426	1.548500	4.273414
C	3.190447	3.471406	-0.595041
C	1.266129	2.209667	3.859749
H	-0.878352	1.545935	-5.167803
H	-4.369638	-0.698904	2.897859
H	-3.176694	0.407831	-4.327089
H	-5.185099	-0.912363	0.334076
H	3.316764	3.621784	-2.782736
H	-0.170166	1.357893	5.284771
H	4.052317	4.001276	-0.215202
H	2.035380	2.675024	4.459381
C	0.838524	2.207140	-2.981153
C	-1.782056	0.507564	3.087900
C	2.193742	2.806867	1.611232
C	-3.248791	0.138823	-1.484052
H	1.244433	2.488760	-3.946496
H	-2.208242	0.258330	4.053273
H	3.014955	3.307641	2.111535
H	-4.139976	-0.238692	-1.973283
C	-1.304338	4.078648	-0.728539
C	-2.126321	3.606063	1.205627
C	-2.541586	4.869684	0.911516
N	-1.355587	3.125061	0.174654
N	-2.008922	5.153739	-0.322230
H	-3.151241	5.572979	1.451668
H	-2.327201	3.011447	2.080845
H	-0.778780	4.026019	-1.667906
H	-2.122410	6.009913	-0.838059
C	0.668652	-0.746540	-0.054232
C	0.912283	-1.324221	-1.415116
F	1.503726	-2.559156	-1.398790
F	-0.196235	-1.485795	-2.141295
F	1.756219	-0.548731	-2.130398
C	-0.001025	-1.652906	0.883285
O	0.293301	-1.751724	2.062195
O	-0.990413	-2.342360	0.318378
C	-1.790386	-3.159215	1.166972
H	-1.205122	-4.028207	1.484593
H	-2.067477	-2.591618	2.057942
C	-3.020113	-3.583163	0.409938
C	-4.069357	-4.171878	1.113498
C	-3.144378	-3.391061	-0.962081
C	-5.229971	-4.559186	0.457658
C	-4.308916	-3.775323	-1.618510
C	-5.354809	-4.358403	-0.913811
H	-3.983136	-4.314671	2.186566
H	-2.340142	-2.915370	-1.507938
H	-6.041028	-5.010962	1.017932
H	-4.397976	-3.612873	-2.687128
H	-6.262607	-4.654247	-1.427569
N	1.962418	-0.318592	0.574255
H	2.142162	0.623063	0.208774
H	1.702979	-0.191884	1.559713
C	3.194868	-1.106926	0.483826
C	4.219072	-0.628989	-0.308890
C	3.332149	-2.293631	1.194402
C	5.408271	-1.342644	-0.413418
C	4.510380	-3.004842	1.093093
C	5.555441	-2.538805	0.286833
H	4.097993	0.300020	-0.856837
H	2.520195	-2.650602	1.814738
H	6.199313	-0.956041	-1.040844
H	4.647143	-3.933286	1.633205
O	6.661567	-3.311967	0.255696
C	7.746852	-2.899326	-0.556347
H	8.513172	-3.662527	-0.440258
H	7.453478	-2.837257	-1.609210
H	8.144821	-1.933730	-0.227666

Rosetta params files for pro*R* and pro*S* heme-ylide reaction intermediates

RBY.params (*R* enantioselectivity)

```
NAME SBY
IO_STRING SBY Z
TYPE LIGAND
AA UNK
ATOM Fe   VIRT  X   0
ATOM  N1  VIRT  X   0
ATOM  N2  VIRT  X   0
ATOM  N3  VIRT  X   0
ATOM  N4  VIRT  X   0
ATOM CARB CH1   X   -0.06
ATOM  CTF CH1   X   -0.06
ATOM  F1  F     X   -0.22
ATOM  F2  F     X   -0.22
ATOM  F3  F     X   -0.22
ATOM CEST COO   X   0.65
ATOM OCAR OOC   X   -0.73
ATOM OEST OH    X   -0.63
ATOM CMET CH2   X   -0.15
ATOM CBN1 aroC  X   -0.08
ATOM CBN2 aroC  X   -0.08
ATOM CBN4 aroC  X   -0.08
ATOM CBN6 aroC  X   -0.08
ATOM CBN5 aroC  X   -0.08
ATOM CBN3 aroC  X   -0.08
ATOM HBN3 Haro  X   0.15
ATOM HBN5 Haro  X   0.15
ATOM HBN6 Haro  X   0.15
ATOM HBN4 Haro  X   0.15
ATOM HBN2 Haro  X   0.15
ATOM HMT1 Hapo  X   0.13
ATOM HMT2 Hapo  X   0.13
ATOM NAME NH2O  X   -0.44
ATOM CAR1 aroC  X   -0.08
ATOM CAR2 aroC  X   -0.08
ATOM CAR4 aroC  X   -0.08
ATOM CAR6 aroC  X   -0.08
ATOM CAR5 aroC  X   -0.08
ATOM CAR3 aroC  X   -0.08
ATOM HAR3 Haro  X   0.15
ATOM HAR6 Haro  X   0.15
ATOM  OME OH    X   -0.63
ATOM  CME CH3   X   -0.24
```

```
ATOM HME1 Hapo  X   0.13
ATOM HME2 Hapo  X   0.13
ATOM HME3 Hapo  X   0.13
ATOM HAR5 Haro  X   0.15
ATOM HAR2 Haro  X   0.15
ATOM HAM1 Hpol  X   0.46
ATOM HAM2 Hpol  X   0.46
BOND_TYPE Fe    N1  1
BOND_TYPE Fe    N2  1
BOND_TYPE Fe    N3  1
BOND_TYPE Fe    N4  1
BOND_TYPE Fe   CARB 1
BOND_TYPE CARB  CTF 1
BOND_TYPE CARB CEST 1
BOND_TYPE CARB NAME 1
BOND_TYPE  CTF  F1  1
BOND_TYPE  CTF  F2  1
BOND_TYPE  CTF  F3  1
BOND_TYPE CEST OCAR 2
BOND_TYPE CEST OEST 1
BOND_TYPE OEST CMET 1
BOND_TYPE CMET HMT1 1
BOND_TYPE CMET HMT2 1
BOND_TYPE CMET CBN1 1
BOND_TYPE CBN1 CBN2 4
BOND_TYPE CBN1 CBN3 4
BOND_TYPE CBN2 CBN4 4
BOND_TYPE CBN2 HBN2 1
BOND_TYPE CBN3 CBN5 4
BOND_TYPE CBN3 HBN3 1
BOND_TYPE CBN4 CBN6 4
BOND_TYPE CBN4 HBN4 1
BOND_TYPE CBN5 CBN6 4
BOND_TYPE CBN5 HBN5 1
BOND_TYPE CBN6 HBN6 1
BOND_TYPE NAME HAM1 1
BOND_TYPE NAME HAM2 1
BOND_TYPE NAME CAR1 1
BOND_TYPE CAR1 CAR2 4
BOND_TYPE CAR1 CAR3 4
BOND_TYPE CAR2 CAR4 4
BOND_TYPE CAR2 HAR2 1
BOND_TYPE CAR3 CAR5 4
BOND_TYPE CAR3 HAR3 1
BOND_TYPE CAR4 CAR6 4
BOND_TYPE CAR4 HAR5 1
```

```
BOND_TYPE CAR5 CAR6 4
BOND_TYPE CAR5 HAR6 1
BOND_TYPE CAR6  OME 1
BOND_TYPE  OME  CME 1
BOND_TYPE  CME HME1 1
BOND_TYPE  CME HME2 1
BOND_TYPE  CME HME3 1
CHI 1  N1  Fe   CARB  CTF
CHI 2 Fe   CARB  CTF  F1
CHI 3 Fe   CARB CEST OCAR
#CHI 4 Fe   CARB NAME CAR1
CHI 4 CARB CEST OEST CMET
CHI 5 CEST OEST CMET CBN1
CHI 6 OEST CMET CBN1 CBN2
CHI 7 CARB NAME CAR1 CAR2
CHI 8 CAR4 CAR6  OME  CME
NBR_ATOM CARB
NBR_RADIUS 9.635646
ICOOR_INTERNAL   Fe     0.000000    0.000000    0.000000  Fe    N1    N2
ICOOR_INTERNAL    N1    0.000000  180.000000    2.028999  Fe    N1    N2
ICOOR_INTERNAL    N2    0.000001   89.310577    2.005510  Fe    N1    N2
ICOOR_INTERNAL    N3  -104.623260    3.213479    2.006300  Fe    N1    N2
ICOOR_INTERNAL    N4   -73.251698   91.443015    2.039252  Fe    N1    N3
ICOOR_INTERNAL   CARB  -90.481937   89.675852    2.344479  Fe    N1    N4
ICOOR_INTERNAL    CTF  163.774042   63.794427    1.496688  CARB  Fe    N1
ICOOR_INTERNAL    F1   179.079597   65.827355    1.369809   CTF  CARB  Fe
ICOOR_INTERNAL    F2  -120.415534   66.272205    1.331605   CTF  CARB   F1
ICOOR_INTERNAL    F3  -121.751017   69.081202    1.352368   CTF  CARB   F2
ICOOR_INTERNAL   CEST  127.475200   72.276488    1.466490  CARB  Fe    CTF
ICOOR_INTERNAL   OCAR  -94.653341   53.480625    1.207533  CEST  CARB  Fe
ICOOR_INTERNAL   OEST  178.667179   68.783012    1.356201  CEST  CARB  OCAR
ICOOR_INTERNAL   CMET -175.488429   63.076417    1.429093  OEST  CEST  CARB
ICOOR_INTERNAL   CBN1  170.860802   71.789041    1.503343  CMET  OEST  CEST
ICOOR_INTERNAL   CBN2 -138.027418   60.106506    1.391990  CBN1  CMET  OEST
ICOOR_INTERNAL   CBN4 -179.849573   59.378720    1.391023  CBN2  CBN1  CMET
ICOOR_INTERNAL   CBN6   -0.303389   59.971885    1.390212  CBN4  CBN2  CBN1
ICOOR_INTERNAL   CBN5    0.441167   60.375284    1.391295  CBN6  CBN4  CBN2
ICOOR_INTERNAL   CBN3   -0.087983   59.821281    1.389188  CBN5  CBN6  CBN4
ICOOR_INTERNAL   HBN3 -179.226385   60.413646    1.084693  CBN3  CBN5  CBN6
ICOOR_INTERNAL   HBN5 -179.750297   59.893926    1.085067  CBN5  CBN6  CBN3
ICOOR_INTERNAL   HBN6 -179.926595   59.784245    1.084772  CBN6  CBN4  CBN5
ICOOR_INTERNAL   HBN4 -179.377255   60.184774    1.085155  CBN4  CBN2  CBN6
ICOOR_INTERNAL   HBN2 -179.245068   60.351894    1.085895  CBN2  CBN1  CBN4
ICOOR_INTERNAL   HMT1 -121.376669   70.761558    1.090856  CMET  OEST  CBN1
ICOOR_INTERNAL   HMT2 -117.435524   71.410111    1.093975  CMET  OEST  HMT1
ICOOR_INTERNAL   NAME  116.095827   82.237049    1.500428  CARB  Fe    CEST
```

```
ICOOR_INTERNAL   CAR1  166.942196   56.738109    1.468968  NAME  CARB  Fe
ICOOR_INTERNAL   CAR2 -116.356184   62.143195    1.380312  CAR1  NAME  CARB
ICOOR_INTERNAL   CAR4 -179.233286   59.841628    1.391297  CAR2  CAR1  NAME
ICOOR_INTERNAL   CAR6    0.069998   60.443169    1.393481  CAR4  CAR2  CAR1
ICOOR_INTERNAL   CAR5   -0.042150   60.364792    1.399966  CAR6  CAR4  CAR2
ICOOR_INTERNAL   CAR3   -0.100289   59.467928    1.380451  CAR5  CAR6  CAR4
ICOOR_INTERNAL   HAR3 -179.170292   59.947332    1.082013  CAR3  CAR5  CAR6
ICOOR_INTERNAL   HAR6 -179.690361   61.260324    1.082989  CAR5  CAR6  CAR3
ICOOR_INTERNAL    OME -179.945905   55.401105    1.349454  CAR6  CAR4  CAR5
ICOOR_INTERNAL    CME    0.564051   61.647021    1.415948   OME  CAR6  CAR4
ICOOR_INTERNAL   HME1  179.597164   74.127743    1.087094   CME   OME  CAR6
ICOOR_INTERNAL   HME2 -118.729227   68.731224    1.094443   CME   OME  HME1
ICOOR_INTERNAL   HME3 -122.508066   68.742598    1.094725   CME   OME  HME2
ICOOR_INTERNAL   HAR5 -179.894983   61.045152    1.082055  CAR4  CAR2  CAR6
ICOOR_INTERNAL   HAR2 -179.704939   59.586433    1.085837  CAR2  CAR1  CAR4
ICOOR_INTERNAL   HAM1 -124.206737   75.312539    1.023892  NAME  CARB  CAR1
ICOOR_INTERNAL   HAM2 -109.468063   75.805555    1.022173  NAME  CARB  HAM1
```

SBY.params (*S* enantioselectivity)

```
NAME RBY
IO_STRING RBY Z
TYPE LIGAND
AA UNK
ATOM Fe   VIRT  X   0
ATOM  N1  VIRT  X   0
ATOM  N2  VIRT  X   0
ATOM  N3  VIRT  X   0
ATOM  N4  VIRT  X   0
ATOM CARB CH1   X   -0.06
ATOM  CTF CH1   X   -0.06
ATOM  F1  F     X   -0.22
ATOM  F2  F     X   -0.22
ATOM  F3  F     X   -0.22
ATOM CEST COO   X   0.65
ATOM OCAR OOC   X   -0.73
ATOM OEST OH    X   -0.63
ATOM CMET CH2   X   -0.15
ATOM CBN1 aroC  X   -0.08
ATOM CBN2 aroC  X   -0.08
ATOM CBN4 aroC  X   -0.08
ATOM CBN6 aroC  X   -0.08
ATOM CBN5 aroC  X   -0.08
ATOM CBN3 aroC  X   -0.08
ATOM HBN3 Haro  X   0.15
ATOM HBN5 Haro  X   0.15
```

```
ATOM HBN6 Haro  X   0.15
ATOM HBN4 Haro  X   0.15
ATOM HBN2 Haro  X   0.15
ATOM HMT1 Hapo  X   0.13
ATOM HMT2 Hapo  X   0.13
ATOM NAME NH2O  X   -0.44
ATOM CAR1 aroC  X   -0.08
ATOM CAR2 aroC  X   -0.08
ATOM CAR4 aroC  X   -0.08
ATOM CAR6 aroC  X   -0.08
ATOM CAR5 aroC  X   -0.08
ATOM CAR3 aroC  X   -0.08
ATOM HAR3 Haro  X   0.15
ATOM HAR6 Haro  X   0.15
ATOM  OME OH    X   -0.63
ATOM  CME CH3   X   -0.24
ATOM HME1 Hapo  X   0.13
ATOM HME2 Hapo  X   0.13
ATOM HME3 Hapo  X   0.13
ATOM HAR5 Haro  X   0.15
ATOM HAR2 Haro  X   0.15
ATOM HAM1 Hpol  X   0.46
ATOM HAM2 Hpol  X   0.46
BOND_TYPE Fe    N1  1
BOND_TYPE Fe    N2  1
BOND_TYPE Fe    N3  1
BOND_TYPE Fe    N4  1
BOND_TYPE Fe   CARB 1
BOND_TYPE CARB  CTF 1
BOND_TYPE CARB CEST 1
BOND_TYPE CARB NAME 1
BOND_TYPE  CTF  F1  1
BOND_TYPE  CTF  F2  1
BOND_TYPE  CTF  F3  1
BOND_TYPE CEST OCAR 2
BOND_TYPE CEST OEST 1
BOND_TYPE OEST CMET 1
BOND_TYPE CMET HMT1 1
BOND_TYPE CMET HMT2 1
BOND_TYPE CMET CBN1 1
BOND_TYPE CBN1 CBN2 4
BOND_TYPE CBN1 CBN3 4
BOND_TYPE CBN2 CBN4 4
BOND_TYPE CBN2 HBN2 1
BOND_TYPE CBN3 CBN5 4
BOND_TYPE CBN3 HBN3 1
```

```
BOND_TYPE CBN4 CBN6 4
BOND_TYPE CBN4 HBN4 1
BOND_TYPE CBN5 CBN6 4
BOND_TYPE CBN5 HBN5 1
BOND_TYPE CBN6 HBN6 1
BOND_TYPE NAME HAM1 1
BOND_TYPE NAME HAM2 1
BOND_TYPE NAME CAR1 1
BOND_TYPE CAR1 CAR2 4
BOND_TYPE CAR1 CAR3 4
BOND_TYPE CAR2 CAR4 4
BOND_TYPE CAR2 HAR2 1
BOND_TYPE CAR3 CAR5 4
BOND_TYPE CAR3 HAR3 1
BOND_TYPE CAR4 CAR6 4
BOND_TYPE CAR4 HAR5 1
BOND_TYPE CAR5 CAR6 4
BOND_TYPE CAR5 HAR6 1
BOND_TYPE CAR6  OME 1
BOND_TYPE  OME  CME 1
BOND_TYPE  CME HME1 1
BOND_TYPE  CME HME2 1
BOND_TYPE  CME HME3 1
CHI 1  N1  Fe   CARB  CTF
CHI 2 Fe   CARB  CTF  F1
CHI 3 Fe   CARB CEST OCAR
#CHI 4 Fe   CARB NAME CAR1
CHI 4 CARB CEST OEST CMET
CHI 5 CEST OEST CMET CBN1
CHI 6 OEST CMET CBN1 CBN2
CHI 7 CARB NAME CAR1 CAR2
CHI 8 CAR4 CAR6  OME  CME
NBR_ATOM CARB
NBR_RADIUS 9.635646
ICOOR_INTERNAL   Fe     0.000000    0.000000    0.000000  Fe    N1    N2
ICOOR_INTERNAL    N1    0.000000  179.999999    2.005510  Fe    N1    N2
ICOOR_INTERNAL    N2    0.000000   89.310577    2.028999  Fe    N1    N2
ICOOR_INTERNAL    N3  -70.453531    2.254346    2.039252  Fe    N1    N2
ICOOR_INTERNAL    N4 -106.437182   89.877524    2.006300  Fe    N1    N3
ICOOR_INTERNAL   CARB  -92.765254   88.360942    2.344479  Fe    N1    N4
ICOOR_INTERNAL    CTF  105.526966   63.794427    1.496688  CARB  Fe    N1
ICOOR_INTERNAL    F1 -179.079597   65.827355    1.369809   CTF  CARB  Fe
ICOOR_INTERNAL    F2  120.415534   66.272205    1.331605   CTF  CARB   F1
ICOOR_INTERNAL    F3  121.751017   69.081202    1.352368   CTF  CARB   F2
ICOOR_INTERNAL   CEST -127.475200   72.276488    1.466490  CARB  Fe    CTF
ICOOR_INTERNAL   OCAR   94.653341   53.480625    1.207533  CEST  CARB  Fe
```

```
ICOOR_INTERNAL   OEST -178.667179   68.783012    1.356201  CEST  CARB  OCAR
ICOOR_INTERNAL   CMET  175.488429   63.076417    1.429093  OEST  CEST  CARB
ICOOR_INTERNAL   CBN1 -170.860802   71.789041    1.503343  CMET  OEST  CEST
ICOOR_INTERNAL   CBN2  138.027418   60.106506    1.391990  CBN1  CMET  OEST
ICOOR_INTERNAL   CBN4  179.849573   59.378720    1.391023  CBN2  CBN1  CMET
ICOOR_INTERNAL   CBN6    0.303389   59.971885    1.390212  CBN4  CBN2  CBN1
ICOOR_INTERNAL   CBN5   -0.441167   60.375284    1.391295  CBN6  CBN4  CBN2
ICOOR_INTERNAL   CBN3    0.087983   59.821281    1.389188  CBN5  CBN6  CBN4
ICOOR_INTERNAL   HBN3  179.226385   60.413646    1.084693  CBN3  CBN5  CBN6
ICOOR_INTERNAL   HBN5  179.750297   59.893926    1.085067  CBN5  CBN6  CBN3
ICOOR_INTERNAL   HBN6  179.926595   59.784245    1.084772  CBN6  CBN4  CBN5
ICOOR_INTERNAL   HBN4  179.377255   60.184774    1.085155  CBN4  CBN2  CBN6
ICOOR_INTERNAL   HBN2  179.245068   60.351894    1.085895  CBN2  CBN1  CBN4
ICOOR_INTERNAL   HMT1  121.376669   70.761558    1.090856  CMET  OEST  CBN1
ICOOR_INTERNAL   HMT2  117.435524   71.410111    1.093975  CMET  OEST  HMT1
ICOOR_INTERNAL   NAME -116.095827   82.237049    1.500428  CARB  Fe    CEST
ICOOR_INTERNAL   CAR1 -166.942196   56.738109    1.468968  NAME  CARB  Fe
ICOOR_INTERNAL   CAR2  116.356184   62.143195    1.380312  CAR1  NAME  CARB
ICOOR_INTERNAL   CAR4  179.233286   59.841628    1.391297  CAR2  CAR1  NAME
ICOOR_INTERNAL   CAR6   -0.069998   60.443169    1.393481  CAR4  CAR2  CAR1
ICOOR_INTERNAL   CAR5    0.042150   60.364792    1.399966  CAR6  CAR4  CAR2
ICOOR_INTERNAL   CAR3    0.100289   59.467928    1.380451  CAR5  CAR6  CAR4
ICOOR_INTERNAL   HAR3  179.170292   59.947332    1.082013  CAR3  CAR5  CAR6
ICOOR_INTERNAL   HAR6  179.690361   61.260324    1.082989  CAR5  CAR6  CAR3
ICOOR_INTERNAL    OME  179.945905   55.401105    1.349454  CAR6  CAR4  CAR5
ICOOR_INTERNAL    CME   -0.564051   61.647021    1.415948   OME  CAR6  CAR4
ICOOR_INTERNAL   HME1 -179.597164   74.127743    1.087094   CME   OME  CAR6
ICOOR_INTERNAL   HME2  118.729227   68.731224    1.094443   CME   OME  HME1
ICOOR_INTERNAL   HME3  122.508066   68.742598    1.094725   CME   OME  HME2
ICOOR_INTERNAL   HAR5  179.894983   61.045152    1.082055  CAR4  CAR2  CAR6
ICOOR_INTERNAL   HAR2  179.704939   59.586433    1.085837  CAR2  CAR1  CAR4
ICOOR_INTERNAL   HAM1  124.206737   75.312539    1.023892  NAME  CARB  CAR1
ICOOR_INTERNAL   HAM2  109.468063   75.805555    1.022173  NAME  CARB  HAM1
```

Rosetta constraint files for pro*R* and pro*S* heme-ylide reaction intermediates

RBY.cst (*R* enantioselectivity)

```
Dihedral N1 82 Fe 82 CARB 82 CEST 82 CIRCULARHARMONIC 5.083 0.61
Dihedral OCAR 82 CEST 82 OEST 82 CMET 82 CIRCULARHARMONIC 0 0.38
Dihedral CARB 82 NAME 82 CAR1 82 CAR2 82 CIRCULARHARMONIC 4.252 0.69
AmbiguousConstraint
Dihedral CAR4 82 CAR6 82 OME 82 CME 82 CIRCULARHARMONIC 0 0.74
Dihedral CAR5 82 CAR6 82 OME 82 CME 82 CIRCULARHARMONIC 0 0.74
END
```

SBY.cst (*S* enantioselectivity)

```
Dihedral N1 82 Fe 82 CARB 82 CEST 82 CIRCULARHARMONIC 5.900 0.61
Dihedral OCAR 82 CEST 82 OEST 82 CMET 82 CIRCULARHARMONIC 0 0.38
Dihedral CARB 82 NAME 82 CAR1 82 CAR2 82 CIRCULARHARMONIC 2.031 0.69
AmbiguousConstraint
Dihedral CAR4 82 CAR6 82 OME 82 CME 82 CIRCULARHARMONIC 0 0.74
Dihedral CAR5 82 CAR6 82 OME 82 CME 82 CIRCULARHARMONIC 0 0.74
END
```

Input PDB link lines

```
LINK        SG  CYS A  10                 CAB  HEC A  81                 1.79
LINK        SG  CYS A  13                 CAC  HEC A  81                 2.17
LINK        FE  HEC A  81                CARB  RBY X   1                 2.33
or
LINK        FE  HEC A  81                CARB  SBY X   1                 2.33
```

Rosetta fold tree

1,80,-1 1,81,1 81,82,FE,N1

Starting conformations for the (+) and (−) carbene-borne ester

```
pose.set_chi(3, 82, 90) # For the (+) ester conformation.
or
pose.set_chi(3, 82, 270) # For the (−) ester conformation.
```

Additional Rosetta score function terms

{"fa_intra_atr_nonprotein": 1, "fa_intra_rep_nonprotein": 0.545}

References

1. Sheldrick, G. *Acta Crystallographica Section A* **2015,** *71*, 3-8.
2. Sheldrick, G. *Acta Crystallographica Section C* **2015,** *71*, 3-8.
3. Dolomanov, O. V.; Bourhis, L. J.; Gildea, R. J.; Howard, J. A. K.; Puschmann, H. *Journal of Applied Crystallography* **2009,** *42*, 339-341.
4. Xia, Y.; Chu, W.; Qi, Q.; Xun, L. *Nucleic Acids Research* **2014,** *43*, e12-e12.
5. Ubbink, M.; Van Beeumen, J.; Canters, G. W. *Journal of Bacteriology* **1992,** *174*, 3707-3714.
6. Tang, L.; Gao, H.; Zhu, X.; Wang, X.; Zhou, M.; Jiang, R. *BioTechniques* **2012,** *52*, 149-158.
7. Ishii, M.; Igarashi, Y.; Kodama, T. *Agricultural and Biological Chemistry* **1987,** *51*, 1695-1696.
